# Reference intervals for the urinary steroid metabolome: The impact of sex, age, day and night time on human adult steroidogenesis

**DOI:** 10.1371/journal.pone.0214549

**Published:** 2019-03-29

**Authors:** Daniel Ackermann, Michael Groessl, Menno Pruijm, Belen Ponte, Geneviève Escher, Claudia H. d’Uscio, Idris Guessous, Georg Ehret, Antoinette Pechère-Bertschi, Pierre-Yves Martin, Michel Burnier, Bernhard Dick, Bruno Vogt, Murielle Bochud, Valentin Rousson, Nasser A. Dhayat

**Affiliations:** 1 Department of Nephrology and Hypertension and Department of Clinical Research, Inselspital, Bern University Hospital, University of Bern, Bern, Switzerland; 2 Nephrology Service, University Hospital of Lausanne, Lausanne, Switzerland; 3 Nephrology Service, Department of Specialties of Internal Medicine, University Hospital of Geneva, Geneva, Switzerland; 4 Department of Community Medicine, Primary Care and Emergency Medicine, University Hospital of Geneva, Geneva, Switzerland; 5 Cardiology Service, Department of Specialties of Internal Medicine, University Hospital of Geneva, Geneva, Switzerland; 6 Endocrinology Service, Department of Internal Medicine Specialties, University Hospital of Geneva, Geneva, Switzerland; 7 Institute of Social and Preventive Medicine, University Hospital of Lausanne, Lausanne, Switzerland; International University of Health and Welfare, School of Medicine, JAPAN

## Abstract

**Objective:**

Urinary steroid metabolomics by GC-MS is an established method in both clinical and research settings to describe steroidogenic disorders. However, population-based reference intervals for adults do not exist.

**Methods:**

We measured daytime and night time urinary excretion of 40 steroid metabolites by GC-MS in 1128 adult participants of European ancestry, aged 18 to 90 years, within a large population-based, multicentric, cross-sectional study. Age and sex-related patterns in adjacent daytime and night time urine collections over 24 hours were modelled for each steroid metabolite by multivariable linear mixed regression. We compared our results with those obtained through a systematic literature review on reference intervals of urinary steroid excretion.

**Results:**

Flexible models were created for all urinary steroid metabolites thereby estimating sex- and age-related changes of the urinary steroid metabolome. Most urinary steroid metabolites showed an age-dependence with the exception of 6β-OH-cortisol, 18-OH-cortisol, and β-cortol. Reference intervals for all metabolites excreted during 24 hours were derived from the 2.5^th^ and 97.5^th^ percentile of modelled reference curves. The excretion rate per period of metabolites predominantly derived from the adrenals was mainly higher during the day than at night and the correlation between day and night time metabolite excretion was highly positive for most androgens and moderately positive for glucocorticoids.

**Conclusions:**

This study gives unprecedented new insights into sex- and age-specificity of the human adult steroid metabolome and provides further information on the day/night variation of urinary steroid hormone excretion. The population-based reference ranges for 40 GC-MS-measured metabolites will facilitate the interpretation of steroid profiles in clinical practice.

## Introduction

Steroid hormones mediate a wide variety of biological processes and analysis of these in serum and their metabolites in urine are used to detect steroid misuse and disorders of reproductive function, sexual development, electrolyte balance, blood pressure and stress response [1–7]. They are small hydrophobic molecules that are synthesized from cholesterol in the testis and ovary, in the adrenal cortex, the placenta and the brain. In humans, the small number of circulating sex steroids, glucocorticoids, corticosterones and mineralocorticoids are converted into a large number of metabolites predominantly excreted via the urine in which they are easily accessible for analysis [8]. Major milestones in the development of urinary steroid hormone analysis were built by the advances in transforming the steroids into suitable derivates for GC-MS analysis, e.g. by producing stable oxime-silyl-derivatives, and by the development of a derivative purification method based on lipophilic Sephadex in 1977 [9–11]. The long history of urinary steroid analysis and the current role of GC-MS in steroid analysis compared with other analytical techniques as LC-MS/MS were sufficiently addressed in detail elsewhere [12–15].

For the correct clinical interpretation of a urinary steroid profile a comparison with reference intervals is necessary. Early efforts have been made to generate such values for the 24-hour excretion of the adult urinary steroid metabolome as summarized by Cedric H. L. Shackleton in 1986 [16]. Since then, new data about urinary steroid excretion values measured by GC-MS in adults from the general population were published, however, these data are limited in several respects: 1) the completeness of characterisation of the reference population, 2) the number of analyzed individuals: it ranged from 13 to 120 women and 10 to 120 men per study with overall less than 400 women and less than 400 men in all studies on reference intervals together, 3) the number of analyzed urinary steroids, which remained below 20 in most studies, and 4) the comparability of the laboratory data if several laboratories with different analytical systems were involved in the same study. None of the published reference intervals come from a formal population-based study or was large enough to provide sex- and age-specific reference intervals according to published recommendations [17]. While recent decades have seen many new technical developments and enhancements in the field of steroid hormone diagnostics, the method of GC-MS has established itself more and more firmly and is still regarded as the gold-standard allowing a comprehensive analysis of the steroid hormone metabolome [15].

The human steroid metabolome is influenced by sex and age and this has been taken into account in the past by providing steroid excretion reference intervals for men and women and for different age groups [18–23]. Furthermore, the human steroid metabolome varies at least in part with sleep and / or with the time of day [24, 25], but data supporting this notion for the urinary steroid metabolome in humans are still sparse [26, 27].

This study aims to bridge some of these gaps. The study was conducted 1) to summarize the available data on reference intervals for the urinary steroid excretion from healthy adults of the general population measured by GC-MS during the last 30 years since 1986 and 2) to describe sex- and age-specificity and differences between day and night excretion of a large number of urinary steroid metabolites in a thoroughly characterized general population of European descent measured by GC-MS and 3) to create sex- and age-specific reference intervals for the 24-hour urinary steroid metabolome that can be used in routine clinical work.

## Subjects and methods

### Literature search for reference intervals of quantitative urinary steroid excretion

A literature search was conducted to find reference intervals for the urinary steroid metabolome from healthy adults of the general population measured by GC-MS that were published since 1986. Using the PubMed electronic database and Google and Google Scholar, the search included the terms “urine/urinary steroid metabolome”, “urine/urinary steroid profile/profiling”, “urine/urinary steroids”, “normative data”, “reference values/intervals”, “GC-MS/gas chromatography-mass spectrometry”, “steroidogenesis”, “steroid synthesis/metabolism” and the trivial and systematic names of the steroid compounds measured in this study.

### Study population

The Swiss Kidney Project on Genes in Hypertension (SKIPOGH) is a multicenter, family-based, cross-sectional study exploring the genetic and non-genetic determinants of blood pressure and renal function in the general adult population [28]. 1128 participants were recruited in the regions of Bern and Geneva and in the city of Lausanne in Switzerland from December 2009 to March 2013 by different strategies. In Geneva, a random sample from an index list provided by the population-based Bus Santé study [29] was selected, in the city of Lausanne a random sample of volunteers were taken from the population-based CoLaus study [30], and in Bern a random sample of participants was selected from the cantonal telephone registry. Inclusion criteria for participation in the study were as follows: 1) age ≥ 18 years; 2) European ancestry; 3) at least 1 and ideally 3 first-degree family members willing to participate. The SKIPOGH study adhered to the Declaration of Helsinki and was approved by the competent institutional ethics committees in Geneva, Lausanne and Bern. All participants provided written informed consent.

### Study visit

The family members were contacted separately and individual appointments for a study visit were made. All participants completed a comprehensive health questionnaire about current and past medical history, medication, nutrition and lifestyle habits. The health questionnaire was checked for completeness and accuracy during the study visit. Body weight was measured to the nearest 0.1 kg with an electronic scale in the morning after an overnight fast in light indoor clothing and height was measured to the nearest 0.5 cm with a wall-mounted stadiometer. Fasting blood venous samples were drawn and were analyzed by standard clinical laboratory methods at each center. The Chronic Kidney Disease Epidemiology Collaboration (CKD-EPI) 2009 equation was used to calculate the estimated glomerular filtration rate (eGFR)[31]. Diabetes was defined as reported, treated, or fasting glycemia ≥7 mmol/L. Hypertension was defined as either systolic blood pressure ≥140 mmHg, diastolic blood pressure ≥90 mmHg, or the use of antihypertensive medications.

### Urine collection procedure

A 24-hour urine sample was collected separately for day and night. Participants received two labelled sterile 3-L preservative-free polyethylene containers UriSet 24 (Sarstedt, Nümbrecht, Germany) and standardized written and precise oral instructions. Daytime urine collection started in the morning with the study visit after an overnight fast and ended before bedtime at the same day. Night time urine collection ended with the collection of the first morning urine after waking up. Participants were asked to eat and drink as usual. Containers with daytime and night time urine collection were separately mixed and weighed and aliquoted into 30 mL low density polyethylene Wide-Mouth Bottles (Thermo Scientific, Rochester, New York). Urine aliquots were immediately stored at -80°C and were sent to the steroid laboratory of the Department of Nephrology and Hypertension at the Bern University Hospital, Switzerland, for centralized steroid analysis by GC-MS. Completeness of 24-hour urine collection was assessed based on the amount of urinary creatinine excretion as recently published [32].

### Quantification of steroid compounds by GC-MS

**Fig 1:**
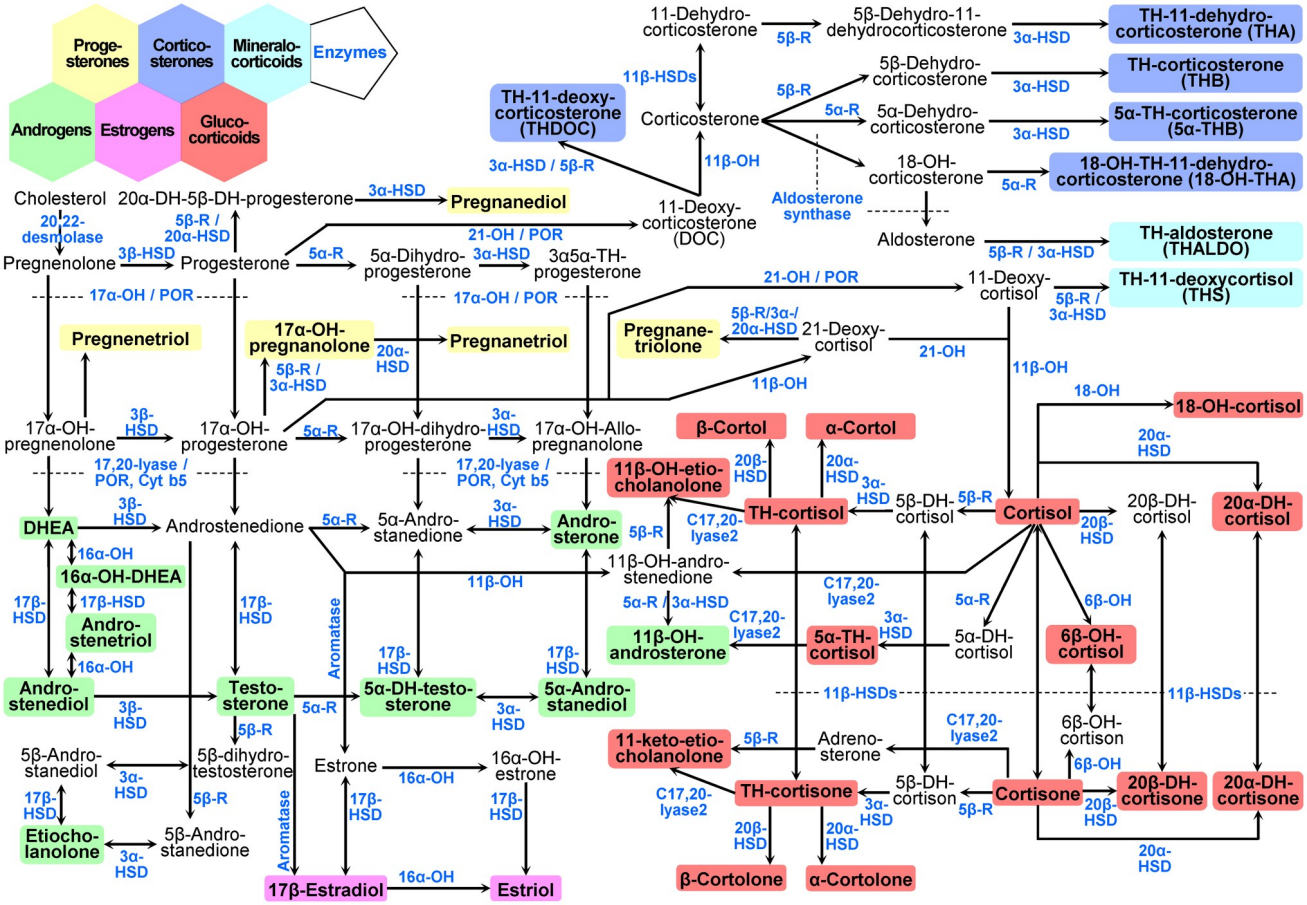
Pathways of human steroid hormone biosynthesis. Pathways from multiple steroid hormone producing cell types are combined to provide an overall view about the 40 urinary steroid metabolites measured in this study and their biosynthesis processes. Steroid groups are highlighted by colored background as indicated and enzyme activities are displayed in blue color. Abbreviations: DHEA: Dehydroepiandrosterone. The OH in enzyme names indicates a hydroxylase, e.g. 17α-OH: 17α-hydroxylase. The OH in steroid names indicates a hydroxyl group, e.g. 17-OH-pregnanolone: 17-hydroxy-pregnanolone. DH: dihydro; TH: tetrahydro; HSD: hydroxysteroid dehydrogenase; POR: P450 oxidoreductase; Cyt b5: Cytochrome b5; 5α-R: 5α-reductase; 5β-R: 5β-reductase.

Urinary excretion of 40 steroid hormone compounds listed in Table 1 and depicted in Fig 1 were quantified separately in μg/day and in μg/night time by an *in-house* adapted GC-MS method previously described [13, 33]. Table 1 further provides method-validation information for all steroid analytes. The corresponding calibration curves are given in S1 Fig and an example of a selected-ion monitoring chromatogram is given in S2 Fig.

**Table 1:** Steroid compounds measured in urine.

Trivial name	Systematic name	M	QIon	RT	LOD	LOQ	R^2^	Rec	CV1	CV2
**Progesterones**										
17α-OH-pregnanolone	3β,17-dihydroxy-5-pregnen-20-one	334.5	476	17.6	16	54	0.997	88	2.3	15.9
pregnanetriol	5β-pregnane-3α,17α,20α-triol	336.5	435	19.6	180	600	0.999	107	5.5	17.3
pregnenetriol	5-pregnene-3β,17α,20α-triol	334.5	433	23.0	22	72	0.997	101	5.5	29.9
pregnanetriolone	3α,17α,20α-trihydroxy-5β-pregnan-11-one	350.5	449	22.0	487	1622	0.994	81	10.5	23.3
pregnanediol	5β-pregnane-3α,20α-diol	316.5	269	19.0	92	307	0.995	108	8.6	20.2
**Androgens**										
dehydroepiandrosterone	3β-hydroxy-5-androsten-17-one	288.4	268	15.4	97	324	0.989	85	6.7	19
16α-OH-dehydroepiandrosterone	3β,16α-dihydroxy-5-androsten-17-one	304.4	266	18.3	614	2046	0.996	119	3.1	13.8
androstenediol	5-androstene-3β,17β-diol	290.4	239	15.9	109	364	0.997	83	6.5	19.1
androstenetriol	5-androstene-3β,16α,17β-triol	306.4	432	20.6	278	928	0.998	100	6.2	16.1
testosterone	17β-hydroxy-4-androsten-3-one	288.4	389	16.7	506	1686	0.993	85	13.4	31.2
5α-DH-testosterone	17β-hydroxy-5α-androstan-3-one	290.4	391	16.0	544	1812	0.990	85	5.3	22.8
androstanediol	5α-androstane-3α,17β-diol	292.4	331	14.6	258	860	0.993	95	5.9	16.1
androsterone	3α-hydroxy-5α-androstan-17-one	290.4	270	14.4	48	161	0.996	85	6	20.8
11β-OH-androsterone	3α,11β-dihydroxy-5α-androstan-17-one	306.4	268	17.6	204	680	0.994	85	6.2	16.2
etiocholanolone	3α-Hydroxy-5β-androstan-17-one	290.4	270	14.6	29	98	0.992	118	4.8	18.8
**Estrogens**										
17β-estradiol	1,3,5(10)-estratriene-3,17β-diol	272.4	416	16.5	205	682	0.999	81	17.4	37.3
estriol	1,3,5(10)-estratriene-3,16α,17β-triol	288.4	504	21.3	96	320	0.997	92	13.2	29
**Corticosterones**										
TH-11-deoxycorticosterone	3α,21-dihydroxy-5β-pregnan-20-one	334.5	476	21.2	516	1721	0.995	96	16.5	16.9
TH-11-dehydrocorticosterone	3α,21-dihydroxy-5β-pregnane-11,20-dione	348.5	490	23.6	105	349	0.996	88	21.2	26.9
18-OH-TH-11-dehydrocorticosterone	3α,18,21-trihydroxy-5β-pregnane-11,20-dione	364.5	457	26.0	7774	25913	0.999	92	24.5	30.5
TH-corticosterone	3α,11β,21-trihydroxy-5β-pregnan-20-one	350.5	564	24.1	266	888	0.995	81	5.4	10
5α-TH-corticosterone	3α,11β,21-trihydroxy-5α-pregnan-20-one	350.5	564	24.5	60	201	0.993	87	6.4	14.3
**Mineralocorticoids**										
TH-aldosterone	11β,18-epoxy-3α,18,21-trihydroxy-5β-pregnan-20-one	364.5	506	25.5	1438	4793	0.988	83	12.1	29
TH-11-deoxycortisol	3α,17,21-trihydroxy-5β-pregnan-20-one	350.5	564	21.0	388	1294	0.993	89	6.9	12.9
**Glucocorticoids**										
cortisol	11β,17,21-trihydroxy-4-pregnene-3,20-dione	362.5	605	30.8	336	1121	0.973	89	5.8	33.9
6β-OH-cortisol	6β,11β,17α,21-tetrahydroxy-4-pregnene-3,20-dione	378.5	513	32.0	1144	3814	0.989	81	6.5	40.3
18-OH-cortisol	11,17,18,21-tetrahydroxy-4-pregnene-3,20-dione	378.5	344	32.0	38669	128898	0.996	95	12.3	29.9
20α-DH-cortisol	11β,17,20α,21-tetrahydroxy-4-pregnen-3-one	364.5	296	32.8	3660	12199	0.994	97	5.2	19.8
TH-cortisol	3α,11β,17,21-tetrahydroxy-5β-pregnan-20-one	366.5	652	24.8	122	408	0.995	96	7.1	12.2
α-cortol	5β-pregnane-3α,11β,17α,20α,21-pentol	368.5	343	27.2	249	830	0.999	90	6.1	14
β-cortol	5β-pregnane-3α,11β,17α,20β,21-pentol	368.5	343	26.0	228	758	0.991	96	5.9	14.4
11β-OH-etiocholanolone	3α,11β-dihydroxy-5β-androstan-17-one	306.4	268	17.9	127	423	0.994	98	12.9	15.4
5α-TH-cortisol	3α,11β,17,21-tetrahydroxy-5α-pregnan-20-one	366.5	652	25.1	40	134	0.995	103	8.8	18.6
cortisone	17,21-dihydroxy-4-pregnene-3,11,20-trione	360.5	531	29.0	987	3290	0.973	88	10	25.5
20α-DH-cortisone	17α,20α,21-trihydroxy-4-pregnene-3,11-dione	362.5	402	31.6	523	1744	0.990	101	4.9	19.5
20β-DH-cortisone	17α,20β,21-trihydroxy-4-pregnene-3,11-dione	362.5	402	30.9	767	2556	0.990	98	5.1	18.4
TH-cortisone	3α,17,21-trihydroxy-5β-pregnan-11,20-dione	364.5	578	23.3	422	1408	0.996	96	8.1	11.1
α-cortolone	3α,17α,20α,21-tetrahydroxy-5β-pregnane-11-one	366.5	449	25.4	23	77	0.995	101	5.4	17.1
β-cortolone	3α,17α,20β,21-tetrahydroxy-5β-pregnane-11-one	366.5	449	26.1	49	162	0.984	106	5.5	15.3
11-keto-etiocholanolone	3α-hydroxy-5β-androstane-11,17-dione	304.4	269	16.1	435	1450	0.994	82	3.2	22

The nomenclature used for systematic names is in accordance with the recommendations of the IUPAC commission on the Nomenclature of Organic Chemistry published in 1969 [34] amended by the IUPAC–IUB Commission on Biochemical Nomenclature in 1971 [35] and again revised in 1989 [36]. M: molar mass [g/mol]. QIon: quantifier ion [m/z]. RT: retention time [min]. LOD: limit of detection [pg per sample]. LOQ: limits of quantitation [pg per sample]. R2: correlation coefficient of the linear calibration curve shown in S1 Fig. Rec: Recovery in %. CV1: intraassay coefficient of variation [%] (n = 58). CV2: interassay coefficient of variation [%] (n = 119). CV1 and CV2 were determined for a urine volume of 1.5 mL. OH: hydroxy, DH: dihydro, TH: tetrahydro.

In brief, urine sample preparation consisted of 1) pre-extraction on a Sep-Pak C18 column with the recovery standard medroxyprogesterone, 2) enzymatic hydrolysis with sulfatase and β-glucuronidase/arylsulfatase, 3) extraction of the free steroids from the hydrolysis mixture again on a Sep-Pak C18 cartridge, 4) derivatization with methoxyamine HCl 2% in pyridine at 60°C for one hour after adding the two standards Stigmasterol and 3β5β-TH-aldosterone and derivatization with Trimethylsilylimidazole (TMSI) at 100°C for 16 hours, 5) purification by gel filtration on a Lipidex 5000 column. The derivatized samples were analyzed by mass spectrometric analyses on a gas chromatograph 7890A coupled to a mass selective detector Hewlett-Packard 5975C (both from Agilent Technologies, La Jolla, California, USA) providing selected ion monitoring.

### Quality controls of the GC-MS method

The reproducibility of the applied GC-MS method is continuously monitored by an internal quality control. Urine samples of the same healthy volunteer are measured in parallel in all measurement series and the results are compared with the standard values derived from 15 measurements of this volunteer. The steroid laboratory participates monthly in an external quality control organized by the Foundation for Quality Medical Laboratory Diagnostics skml (Stichting Kwaliteitsbewaking Medische Laboratoriumdiagnostiek, Nijmegen, The Netherlands). Only analytes that fulfilled internal and external quality assessment requirements are included in steroid profiles. For internal quality control, quantitative results have to be within ±20% of the individual reference intervals. The external quality control is evaluated based on multiple of median (MOM) of reported steroids; results had to be better than ±30% of MOM of all participating laboratories for acceptance.

### Selection of reference sample group

Of the 1128 participants, 33 were excluded from the analyses due to missing data for urinary steroid excretion leaving 1095 participants. Additional participants were excluded from the reference sample group for the following reasons: urine under- or over-collection, pregnancy, self-reported bilateral oophorectomy or bilateral oophorectomy with hysterectomy, Addison’s disease (the presence of other hormone disorders was not reported in the cohort), self-reported diagnosis of active malignant disease, self-reported liver disease, estimated glomerular filtration rate less than 30 mL/min per 1.73 m^2^ calculated by CKD-EPI formula, missing data for serum creatinine or urinary creatinine, body mass index <16 kg/ m^2^ or > 40 kg/m^2^, >3 fold elevated γ-glutamyltransferase (>120 U/L for female and >180 U/L for male), aspartate or alanine aminotransferases (>105 U/L for female and > 150 U/L for male), intake of the following drugs (current intake or in the two previous weeks): hormonal contraceptives, hormones to suppress menstrual bleeding, hormonal menopause treatment, systemic glucocorticoids or mineralocorticoids, 5α-reductase inhibitors, aromatase inhibitors, antiepileptic agents, systemic azole derivatives.

### Statistical analysis

Baseline characteristics were expressed as numbers and frequency in % or as mean±SD or median;25^th^-75^th^ percentile. The 24-hour urinary steroid hormone metabolite excretion was calculated in μg/24h from day and night time urine collections by the formula (metabolite[μg/day]+metabolite[μg/night])/(collection time day[min]+collection time day[min]) × 1440 min. Sex-specific differences of baseline characteristics and 24-hour excretion of steroid hormone metabolites were assessed by chi squared test or Mann–Whitney U test, where appropriate, and a *p* value <0.05 was considered to be significant. Day and night time excretion rates of steroid analytes expressed in μg/hour were calculated by the formula metabolite[μg/day])/collection time day[min]×60 min and by the formula metabolite[μg/night time]/collection time night time[min]×60 min, respectively. Differences between day and night time excretion rates of steroid analytes were analyzed for each sex by Wilcoxon signed-rank test and visualized by boxplots and the correlation between night and day values was further assessed by calculating the Spearman’s rank correlation coefficient. Age- and sex-related changes of 24-hour excretion of steroid hormone metabolites were modelled by linear mixed regression models taking family and center effect into account as described in detail in the S1 Text and previously by V. Rousson [34]. Using the Akaike information criterion the best model for each steroid metabolite was separately selected for men and women and was plotted for different percentiles. Reference intervals based on the 2.5^th^-97.5^th^ percentiles for each metabolite were estimated from the described statistical models for men and women separately and for different age groups. All statistical analyses were conducted using the R software, version 3.3.3 [35].

## Results

### Literature search, measured steroid compounds and pathways of steroidogenesis

The literature search yielded twelve hits for studies that published reference intervals for the urinary steroid excretion from healthy adults of the general population measured by GC-MS since 1986 listed in S1 Table [16, 18–23, 36–40]. These studies combined included data on <800 participants, thereby precluding the detailed exploration of the relationship of steroid hormone metabolites with sex and age according to published recommendations [17] and none of them were reported to be population-based, i.e. coming from a true random sample of the general population on the strength of thoroughly described selection criteria. Studies including reference ranges for comparison purposes but not as the main objective of the publication were not considered in this list.

### Characteristics of the reference population

The number of individuals for these references was 838 (459 men, 54.8%). Women were older than men (age mean±SD: 51.2±16.1 versus 47.7±17.6 years, *p* = 0.0054) mainly because younger women using hormonal contraceptives were excluded from the analysis (n = 118). Smoking, hypertension and diabetes were more prevalent in men than in women. Serum electrolytes, parameters of kidney and liver function and parameters of inflammation and blood count were largely within the normal range. The baseline characteristics and laboratory values are shown in S2 Table.

### Sex- and age-related patterns of the urinary steroid metabolome during 24 hours

The 24-hour excretion of all urinary steroid hormone metabolites was right skewed distributed. The median metabolite excretion by sex compared by Mann–Whitney U test revealed a significant higher excretion for almost all metabolites in men than in women as shown in S3 Table, with the exception of pregnanediol (206 vs. 204 μg/24 hours, *p* = 0.57), of TH-aldosterone (456 vs. 378 μg/24 hours, *p* = 0.084), and of 18-OH-cortisol (180 vs. 173 μg/24 hours, *p* = 0.16). The excretion values were visualized by boxplots in S3(a) Fig. The relationship of 24-hour excretion of urinary steroid hormone metabolites to age was assessed using a nonparametric fit and revealed a different kind of relationship to age for men and for women for most metabolites as shown in S3(b) Fig. Therefore, the 24-hour excretion of urinary steroid hormone metabolites in function of age was modelled separately for men and for women. From the selected models, the 2.5^th^, 10^th^, 25^th^, 50^th^, 75^th^, 90^th^ and 97.5^th^ percentile for each metabolite was plotted on the transformed scale in function of age to get reference curves for men and women depicted in S4 Fig. No relationship to age was found for several metabolites, in particular for 11β-OH-androsterone, 18-OH-TH-11-dehydrocorticosterone, cortisol, 5α-TH-cortisol, TH-cortisone, α-cortolone, and β-cortolone in women, and for 20α-DH-cortisol and 20α-DH-cortisone in men, and for 6β-OH-cortisol, 18-OH-cortisol, and β-cortol in women and men. Lower and upper limits of sex- and age-specific reference intervals for the 24-hour excretion of urinary steroid hormone metabolites were derived from the 2.5^th^ and 97.5^th^ percentile for men in Table 2 and women in Table 3.

**Table 2:** Reference intervals for 40 steroid metabolites in 24-hour urine in men.

Metabolite, μg/24 hours	N	Age group, years							CF to nmol/24 hours
		18–29.9	30–39.9	40–49.9	50–59.9	60–69.9	70–79.9	80–85.7	
17α-OH-pregnanolone	451	75.3–584	65.8–535	57.4–489	49.8–446	43–406	37–369	31.7–335	×2.990
pregnanetriol	395	442–2045	385–1814	334–1607	289–1421	250–1255	215–1106	185–974	×2.972
pregnenetriol	448	54–1213	87.4–1580	47–1127	22.3–769	11.4–559	6.7–441	4.7–380	×2.990
pregnanetriolone	457	6–70.8	6.2–78	6.4–86.3	6.6–96	6.8–108	7–121	7.2–138	×2.853
pregnanediol	457	85.5–653	94.4–708	81.1–625	64–516	55.4–459	52.8–441	52.8–441	×3.160
dehydroepiandrosterone	439	36.3–8455	63.7–56319	46.1–17754	24.3–2776	15.3–907	11–445	8.9–287	×3.467
16α-OH-dehydroepiandrosterone	448	75.2–1413	155–2139	75.7–1418	30.6–874	13.4–582	6.8–428	4.4–356	×3.285
androstenediol	453	35.4–1036	53.9–1878	42.9–1359	26–673	17–377	12–234	9–160	×3.444
androstenetriol	456	176–1652	214–1927	158–1518	110–1137	77–857	54.2–652	38.6–500	×3.264
testosterone	451	13.1–177	16.6–204	14.3–187	10.1–152	7.2–126	5.3–105	4–90.1	×3.467
5α-DH-testosterone	456	6–91.6	5.4–86.6	4.9–81.8	4.4–77.3	4–72.9	3.6–68.8	3.2–64.8	×3.444
androstanediol	445	44.1–228	49.6–252	42.4–221	34.6–186	28.1–156	22.7–130	18.2–108	×3.420
androsterone	381	1450–5821	1456–5839	1073–4644	759–3594	541–2810	390–2224	285–1786	×3.444
11β-OH-androsterone	447	357–1724	373–1771	376–1780	367–1752	345–1687	313–1589	273–1462	×3.264
etiocholanolone	390	883–5047	794–4743	673–4308	531–3770	385–3165	249–2531	138–1910	×3.444
17β-estradiol	457	0.7–4.5	0.9–5.5	1–6.1	1.1–6.3	1–5.9	0.9–5.6	0.9–5.6	×3.671
estriol	456	1.8–13.8	1.9–14.5	2–15.1	2.2–15.8	2.3–16.5	2.4–17.3	2.5–18.1	×3.467
TH-11-deoxycorticosterone	455	3.2–23.3	3.1–22.7	2.9–21.4	2.7–19.5	2.4–17.3	2.2–16.2	2.2–16.2	×2.990
TH-11-dehydrocorticosterone	452	49.1–277	49.5–278	46.6–267	40.7–242	32.8–209	28.8–191	28.8–191	×2.869
18-OH-TH-11-dehydrocorticosterone	433	14–259	15.1–297	15.7–318	15.7–318	15.1–297	14–259	12.5–212	×2.743
TH-corticosterone	457	52.6–326	58.3–350	59.8–356	57.1–345	50.5–317	48.6–309	60.1–357	×2.853
5α-TH-corticosterone	457	155–891	170–976	147–844	128–738	119–681	115–663	115–663	×2.853
TH-aldosterone	456	5.1–79.2	5.4–84	5.5–85.6	5.4–83.9	5.1–79.1	4.6–71.7	4–62.5	×2.743
TH-11-deoxycortisol	457	27.5–145	28.4–152	29.4–160	30.4–168	31.5–176	32.6–185	33.8–194	×2.853
cortisol	457	41.6–243	47.1–282	50–302	49.7–300	47.3–282	48.4–291	54.5–335	×2.759
6β-OH-cortisol	457	35.3–303	35.3–303	35.3–303	35.3–303	35.3–303	35.3–303	35.3–303	×2.642
18-OH-cortisol	424	40.7–638	40.7–638	40.7–638	40.7–638	40.7–638	40.7–638	40.7–638	×2.642
20α-DH-cortisol	457	19.2–136	19.2–136	19.2–136	19.2–136	19.2–136	19.2–136	19.2–136	×2.743
TH-cortisol	371	714–2724	866–3301	966–3682	992–3781	938–3575	918–3501	1047–3992	×2.729
α-cortol	452	127–519	157–638	161–658	164–668	169–688	176–717	186–757	×2.714
β-cortol	453	213–1171	213–1171	213–1171	213–1171	213–1171	213–1171	213–1171	×2.714
11β-OH-etiocholanolone	455	29.7–955	15.1–861	27.7–944	43.7–1029	55–1081	59.1–1099	59.1–1099	×3.264
5α-TH-cortisol	381	524–3424	631–3992	578–3715	527–3436	497–3278	488–3226	488–3226	×2.729
cortisone	456	69.2–378	74–404	77–420	77.9–425	76.6–418	73.4–401	68.3–373	×2.774
20α-DH-cortisone	457	11.1–63.3	11.1–63.3	11.1–63.3	11.1–63.3	11.1–63.3	11.1–63.3	11.1–63.3	×2.759
20β-DH-cortisone	457	21.8–127	22.7–131	23.6–135	24.5–140	25.5–145	26.5–149	27.5–154	×2.759
TH-cortisone	407	1543–6080	1643–6378	1667–6449	1611–6285	1483–5902	1297–5332	1072–4623	×2.743
α-cortolone	426	552–2214	587–2338	611–2420	622–2456	618–2443	600–2382	570–2277	×2.729
β-cortolone	427	366–1387	349–1328	332–1271	315–1216	300–1164	285–1114	271–1065	×2.729
11-keto-etiocholanolone	455	97.7–1055	67.3–884	85–986	103–1082	109–1113	102–1076	83–975	×3.285

Reference intervals have been estimated by the described statistical models and are given as 2.5^th^-97.5^th^ percentiles in the unit μg/24 hours for different age groups. N represents the sample number per analyte included in the statistical model. CF: Conversion Factor.

**Table 3:** Reference intervals for 40 steroid metabolites in 24-hour urine in women.

Metabolite, μg/24 hours	N	Age group, years							CF to nmol/24 hours
		18–29.9	30–39.9	40–49.9	50–59.9	60–69.9	70–79.9	80–90.0	
17α-OH-pregnanolone	375	18.2–383	28.4–704	27.5–672	16.5–337	9.7–166	8.2–132	8.2–132	×2.990
pregnanetriol	360	173–1156	216–1346	200–1275	135–975	80.9–696	56.8–554	48.3–501	×2.972
pregnenetriol	378	18.7–810	12–661	7.4–535	4.3–429	2.4–341	1.2–268	0.5–207	×2.990
pregnanetriolone	379	4.1–95.7	3–54.5	3.1–57.9	3.4–67	3.6–77.4	3.9–89.4	4.2–103	×2.853
pregnanediol	376	62.9–2180	95.2–5269	94.6–5192	61.7–2101	38.2–830	29.5–520	27.5–460	×3.160
dehydroepiandrosterone	377	13.8–1623	15.6–1989	13.6–1578	9.1–834	5.7–409	4.1–242	3.2–168	×3.467
16α-OH-dehydroepiandrosterone	379	37.7–1742	33.9–1565	25–1157	15.2–704	8.9–411	5.8–270	4.3–199	×3.285
androstenediol	378	22.5–873	16.8–571	12.6–380	9.5–257	7.2–177	5.6–123	4.3–86.5	×3.444
androstenetriol	378	99.7–1351	72–1051	51.4–813	36.2–625	25.2–476	17.3–361	11.7–271	×3.264
testosterone	367	2.4–55.1	2.7–66.1	2.6–61.8	2.1–45.3	1.6–30.9	1.2–22.8	1.1–18.3	×3.467
5α-DH-testosterone	377	2.9–75.6	2.5–66.9	2.2–59.1	2–52.3	1.8–46.3	1.5–41	1.4–36.2	×3.444
androstanediol	372	8.9–106	10.5–124	9.7–114	7–83.1	4.9–57.7	4–46.7	3.7–44	×3.420
androsterone	349	480–4482	414–3978	307–3138	194–2181	116–1462	75.6–1051	54.3–816	×3.444
11β-OH-androsterone	376	192–1183	192–1183	192–1183	192–1183	192–1183	192–1183	192–1183	×3.264
etiocholanolone	351	543–3870	448–3483	345–3031	243–2540	153–2037	82.1–1551	33.9–1106	×3.444
17β-estradiol	377	0.3–7.6	0.7–26.6	0.9–35.8	0.5–17	0.3–7.1	0.2–5.4	0.2–5.4	×3.671
estriol	374	0.8–36.3	1.6–91.8	1.7–99.5	1–45.3	0.6–19.5	0.5–15	0.5–15	×3.467
TH-11-deoxycorticosterone	378	1.8–34.2	2.5–66.1	2.6–69.5	1.9–39	1.4–21.5	1.2–17.9	1.2–17.9	×2.990
TH-11-dehydrocorticosterone	379	31.5–240	29.5–228	27.6–216	25.9–205	24.3–194	22.7–184	21.2–174	×2.869
18-OH-TH-11-dehydrocorticosterone	342	10–262	10–262	10–262	10–262	10–262	10–262	10–262	×2.743
TH-corticosterone	379	28–226	34.8–261	39.7–285	41.7–294	40.4–288	36.2–268	29.7–235	×2.853
5α-TH-corticosterone	379	60.6–556	57–535	53.5–514	50.2–493	47–474	44.1–454	41.2–436	×2.853
TH-aldosterone	378	5.3–83	5.6–86.3	5.5–84.9	5.1–78.8	4.5–69.1	3.7–57.3	2.9–44.9	×2.743
TH-11-deoxycortisol	379	15–97.3	17.6–114	19.8–128	21.4–138	22.2–144	22.1–143	21.1–137	×2.853
cortisol	379	33.7–250	33.7–250	33.7–250	33.7–250	33.7–250	33.7–250	33.7–250	×2.759
6β-OH-cortisol	378	25.6–282	25.6–282	25.6–282	25.6–282	25.6–282	25.6–282	25.6–282	×2.642
18-OH-cortisol	344	32.4–516	32.4–516	32.4–516	32.4–516	32.4–516	32.4–516	32.4–516	×2.642
20α-DH-cortisol	379	16.7–176	16.2–169	15.6–163	15.2–156	14.7–150	14.2–144	13.8–138	×2.743
TH-cortisol	340	336–1677	416–2015	490–2319	550–2560	589–2714	603–2766	588–2711	×2.729
α-cortol	379	92.5–464	95.1–479	97.8–495	101–511	103–528	106–546	109–564	×2.714
β-cortol	378	125–751	125–751	125–751	125–751	125–751	125–751	125–751	×2.714
11β-OH-etiocholanolone	378	0.5–612	12.2–756	28.4–860	39.1–916	39.3–917	28.9–863	12.7–760	×3.264
5α-TH-cortisol	362	177–1982	177–1982	177–1982	177–1982	177–1982	177–1982	177–1982	×2.729
cortisone	379	48.3–308	56.3–360	59.4–379	56.7–362	50.2–321	47.9–306	47.9–306	×2.774
20α-DH-cortisone	379	8.6–52.3	8.7–53.1	8.3–49.9	7.4–43.7	6.4–36.5	6–34.2	6–34.2	×2.759
20β-DH-cortisone	379	23.9–145	22.5–136	21.2–129	20–121	18.8–114	17.8–107	16.7–101	×2.759
TH-cortisone	360	900–4654	900–4654	900–4654	900–4654	900–4654	900–4654	900–4654	×2.743
α-cortolone	362	405–1932	405–1932	405–1932	405–1932	405–1932	405–1932	405–1932	×2.729
β-cortolone	369	164–854	164–854	164–854	164–854	164–854	164–854	164–854	×2.729
11-keto-etiocholanolone	379	23.6–652	43.4–778	60.8–870	70.9–918	71–919	61–871	43.6–779	×3.285

Reference intervals have been estimated by the described statistical models and are given as 2.5^th^-97.5^th^ percentiles in the unit μg/24 hours for different age groups. N represents the sample number per analyte included in the statistical model. CF: Conversion Factor.

### Sex-related differences in the urinary steroid metabolome at day and night

**Fig 2:**
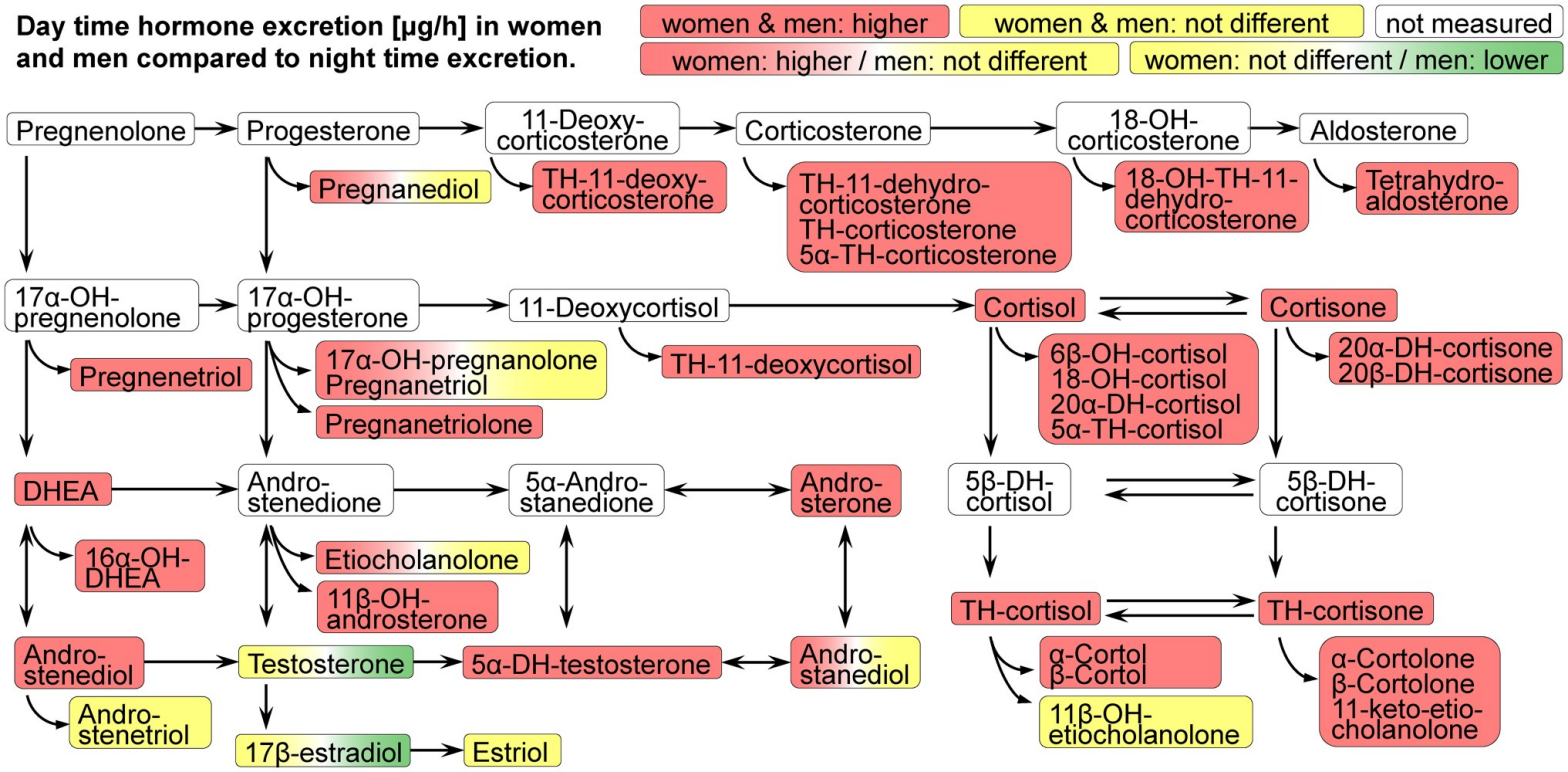
Overview about day and nighttime steroid hormone metabolite excretion in women and men. Day and night time excretion values of urinary steroid metabolites in μg/hour were compared by Wilcoxon signed-rank test separately for men and women. Test results are indicated by coloured boxes: a red colour indicates higher excretion values during the day compared to night time, a green colour indicates lower excretion values during the day compared to night time and a yellow colour indicates no statistically significant difference. A white colour indicates that the metabolite has not been measured in the study. The left half of each box represents the test result indicated by colour for women and the right half for men as shown in the legend. Abbreviations: DHEA: Dehydroepiandrosterone. The OH in steroid names indicates a hydroxyl group, e.g. 17-OH-pregnanolone: 17-hydroxy-pregnanolone. DH: dihydro; TH: tetrahydro.

Day and night time excretion values of urinary steroid metabolites were also right skewed distributed and were compared by Wilcoxon signed-rank test. The median excreted amount in μg/hour was significantly higher during the day than at night for 31 metabolites in men and for 35 metabolites in women as shown in S4 Table. Higher urinary excretion values during the day compared to night time have been found for cortisol and cortisone and for almost all glucocorticoid metabolites in both sexes. During the day higher excretion rates for both sexes have been also found for the most abundant circulating steroid hormone dehydroepiandrosterone (DHEA) and its two metabolites 16α-OH-DHEA and androstenediol, for the most potent androgen 5α-DH-testosterone and also for androsterone, the most abundant androgen metabolite in urine. For etiocholanolone, which is the second most abundant androgen metabolite in urine, a higher night time excretion rate has been revealed in women, but not in men. Excretion rates for testosterone and 17β-estradiol have not been found to be different in women but to be lower at night time in men. The results were visualized by boxplots in S3(e) and S3(f) Fig and summarized in a comprehensive overview in Fig 2. The correlation between day and night time excretion values assessed by Spearman’s rank correlation coefficient in S3(g) and S3(h) Figs and in S5 Table revealed a moderate to high positive correlation among most of androgen and progesterone metabolites and a low to moderate positive correlation among most of glucocorticoid metabolites in men and women. Sex specific differences were found for estrogen metabolites, which were much higher positively correlated in women than in men.

## Discussion

We quantified 40 urinary steroid metabolites by GC-MS in a large number of thoroughly characterized adults of European descent and described the impact of sex and age on each steroid compound and sex-specific differences in day and night time excretions. Furthermore, we created sex- and age-specific reference intervals for these 40 compounds that can be used in routine clinical work as diagnostic tools. According to our literature search of the hitherto available reference intervals for the urinary steroid excretion from adults of the general population measured by GC-MS during the last 30 years since 1986, this is, to our knowledge, the largest study of this kind with such a detailed phenotype and the only one large enough to explore the relationship with age, covering a broad age range in both men and women.

Due to methodological differences, a direct comparison of absolute urinary steroid excretion values obtained by our in-house adapted GC-MS method with values published by other laboratories is possible to only a limited extent. Transferability of reference ranges for the urinary steroid profile could only be guaranteed if our data could be calibrated against a certified reference material which is lacking. For the time being, approximate correction factors could be calculated based on the results of an external quality assessment scheme (available through the Foundation for Quality Medical Laboratory Diagnostics skml) in which our steroid laboratory participates.

However, an inter-methodological comparison of sex- and age-related changes of the urinary steroid metabolome is possible and the studies published by Weykamp et al. and de Jong et al. are suitable for that purpose as both studies provide sex- and age-related reference intervals including 24 or 20 individuals, respectively, per sex- and age-group summarized in S1 Table (15, 19). In line with both studies we found an age-related decline of the 24-hour excretion of most progesterones and of androgens. In addition, we obtained similar results for some glucocorticoids with an age-related increase of TH-cortisol and a slight age-related decrease of allo-TH-cortisol with higher values in men for both steroid hormones. In contrast to the study by Weykamp et al. and similarly to the study by de Jong et al, we found a moderate age-related increase for the 24-hour excretion of the 11-deoxycortisol metabolite TH-11-deoxycortisol in men whereas the results in women were more similar in all three studies with regard to age-related changes. Also for 11β-OH-etiocholanolone, which derives mainly from the metabolism of cortisol [41], our results suggest a different age-related behaviour for men and women.

Our study reveals higher urinary excretion values for almost all glucocorticoids during the day compared to night time in both sexes whereas the excretion rates of sex steroid hormones present a more heterogeneous picture. A previous study on 10 men and 10 women has shown highest excretion rates for the sum of urinary cortisol metabolites between 12:00 and 15:00 for men and women and lowest excretion rates between 24:00 and 03:00 for men and between 03:00 and 06:00 for women and suggested a delay between serum cortisol levels and the excretion of urinary cortisol metabolites of about 4–5 hours [27]. For the 3-hour urinary excretion values of the sum of androsterone and etiocholanolone the same study found a peak after noon and a trough around midnight for men and women. The results of the study are compatible to our results with regard to the circadian excretion of urinary glucocorticoids and of the main urinary androgen androsterone. In addition, our results suggest different circadian excretory behaviours of androgen metabolites, probably also because they are cleared at different rates from the plasma. It has been shown, that after injection of unconjugated radiolabelded [4-^14^C]-androsterone and [4-^14^C]-etiocholanolone both are cleared to urine very rapidly and at nearly identical rates with a half life of about 20 minutes, whereas the corresponding conjugated steroid glucuronides are excreted much less rapidly and at different rates [42].

The observed differences between day and night time steroid excretion values in our study clearly support the presence of a robust circadian rhythm of glucocorticoid synthesis in the adrenal glands [43]. Circadian rhythm refers to evolutionarily conserved biological oscillations following a roughly 24-hour cycle and comes from a genetically operated timekeeping system called the “biological clock” [44, 45]. The suprachiasmatic nucleus of the hypothalamus is considered to act as the master clock in the mammalian organism [46], Adrenal glands show a circadian rhythm of glucocorticoid synthesis [47]. We found a clear separation between day and night time urine steroid excretion, not only for glucocorticoids, but also for most other steroid hormone metabolites that are assumed to be predominantly synthesized in the adrenal glands. In contrast, no tissue-specific circadian rhythm has been so far observed in the mammalian testis [48–50], whereas a circadian clock may play a role in steroidogenesis in the mammalian ovary [51]. These tissue-specific differences may contribute to the observed heterogeneous picture of urinary androgen and estrogen excretion at day and night time.

In view of methodological aspects our study has several strengths: 1) the analyses of all urine samples in the same laboratory, ensuring comparability of the results from different participants, 2) the confirmation of gas chromatography data by MS, and 3) the high number of subjects that permits a high standard in the creation of the lower and upper limit of reference intervals [17]. Moreover, we have been recently able to validate our GC-MS method by multidimensional gas chromatography-time of flight mass spectrometry (GCxGC-TOF MS), a high-resolution method newly developed by our laboratory [52, 53]. Thereby, we are able to exclude for all 40 steroid metabolites analytical problems that may occur due to the effect of matrix interferences in urine or due to the very high chemical similarity of the measured compounds.

The collection completeness is of critical importance especially if urinary excretion is reported as absolute value per collection period (μg/24h). We have made major efforts to achieve a high level of completeness by standardising the collection procedures in all three study centres and procedures were reviewed with each participant subsequently. However, it is not possible to entirely avoid incorrect urine collections and therefore, urinary creatinine excretion was used as a criterion for collection completeness. By the detection of under- and over-collected urine samples on the basis of the lower and upper limit of the 95% CI of the 24-hour creatinine excretion at least extreme collection errors could be excluded from the analyses. The applied regression model to predict 24-hour urinary creatinine excretion was recently developed with data from more than 1000 adults of European descent from the Swiss Survey on Salt and was validated with the participants of SKIPOGH providing a good fit [32]. This comparability of the SKIPOGH population to another independent and large Swiss cross-sectional population sample group underlines the reproducibility of our reference sample group and strengthens the level of confidence in urine collection completeness in this study.

Our study has several limitations. The study was restricted to participants of European descent. Due to the cross-sectional design of the study, urinary steroid hormone excretion data from the same participants at different ages are not available. Information about the phase of menstrual cycle were not collected and therefore reference ranges established for premenopausal women cover the whole period of the menstrual cycle. However, in a study including ten healthy white women of age 20–40 years with regular endogenous menstrual cycles between 24–34 days, the range of 24-hour urinary excretion of androgens and glucocorticoids was in a similar range as measured in our study and no differences were found between menstrual, follicular and luteal phase of the menstrual cycle for the urinary excretion of androgens and of the five glucocorticoids cortisol, cortisone, TH-cortisol, allo-TH-cortisol and TH-cortisone [21]. Moreover, a study comparing salivary cortisol after awaking revealed no differences between 11 women in the luteal phase and 12 women in the follicular phase of the menstrual cycle [54] and in another study it was consciously avoided to record the phase of menstrual cycle because previous unpublished analyses did not reveal an impact on urinary glucocorticoid and androgen metabolites [27]. In contrast, levels of plasma estrogens are associated with menstrual characteristics [55] and therefore the urinary excretion of estrogens would be expected to be influenced by the menstrual cycle phase as suggested by a study on six premenopausal women with highest values during the periovulatory phase and lowest values during the early follicular phase [56]. Further studies are necessary to confirm these results and to light up the impact of menstrual cycle also on the urinary excretion of progesterones, estrogens, corticosterones, and mineralocorticoids. Additionally, it would also be preferable to use isotopically labelled internal standards for more robust and accurate quantification (e.g. one standard per compound class).

Despite these limitations, our study clearly expands the current knowledge on the urinary steroid hormone metabolome in adults from the general population. The study introduces new reference ranges for a large number of urinary steroid hormones and can serve as an important tool in clinical practice.

## Supporting information

S1 Fig Calibration curves. Calibration curves are shown for all analytes injected on column in the range from 39 to 20000 fmol. Crosses indicate data points, the solid line represents the linear regression curve and the dotted lines the 95% confidence interval. Axes are plotted in logarithmic scale (base 10).
(PDF)

S2 Fig Example of a selected-ion monitoring chromatogram. A plot of the sum of ion abundances for the selected compound-specific ions on the vertical axis versus the retention time of each characteristic ion on the horizontal axis simultaneously obtained by a GC-MS analysis of urinary steroid derivatives is shown. Multiple chromatographic peaks indicate the elution of numerous individual steroid compounds. By knowing the retention time for a given steroid indicated in the table, the members of the urinary steroid profile can be distinguished. Some steroid compounds are labeled. QIon: quantifier ion [m/z]. RT: retention time [min].
(PDF)

S3 Fig Descriptive analyses of steroid compounds. A descriptive analysis of the 40 steroid compounds measured in urine is shown including one steroid compound per page. Panel (a): boxplots, steroid (log-scale) by sex; Panel (b): Gasser-Müller nonparametric fits and scatter plots, steroid (log-scale) by age and by sex; Panels (c) and (d): boxplots, transformed steroid for men and women according to optimal power transformation; outliers are plotted as black dots; Panels (e) and (f): steroid (log-scale) by day and night time for men and women; Panels (g) and (h): Spearman correlation of transformed steroid night vs day. Abbreviations used: M = men; W = women; D = day; N = night; TR = optimal power transformation; nout = number of outliers; sk = skewness; ku = kurtosis; delta = robust estimate of mean difference expressed in standard deviations; rho = Spearman correlation coefficient; n = sample size.
(PDF)

S4 Fig Reference curves of steroid compounds. Reference curves of the 40 steroid compounds measured in urine are shown including one steroid compound per page. The percentiles 2.5, 10, 25, 50, 75, 90 and 97.5 of the steroid compounds in function of age and sex are shown on a log-scale. To improve comparison the same scale has been used for men and women.
(PDF)

S1 Table Published urinary steroid excretion reference values. Quantitative urinary steroid excretion values measured by GC-MS and published since 1986 in adults are shown. Abbreviations: GC: gas chromatography, MS: mass spectrometry, F: female, M: male, d: days, y: years.
(PDF)

S2 Table Baseline characteristics of the reference sample group. The number of participants is indicated for each characteristic and sex group. Categorical variables are described by % and continuous variables by their mean±standard deviation or by their median;25^th^-75^th^ percentiles. Sex-specific differences were determined by chi squared test or Mann–Whitney U test, and the corresponding *p* values are indicated.
(PDF)

S3 Table Sex specific differences in 24 hours urinary excretion of steroid hormone metabolites. The available number of participants is indicated for each metabolite stratified for sex. Metabolites in the unit μg/24 hours are described by their median;25^th^-75^th^ percentile. Between-group differences were determined by Mann–Whitney U test, and the corresponding *p* values are indicated.
(PDF)

S4 Table Day and nighttime specific differences in urinary excretion of steroid hormone metabolites in men and women. The available number of participants is indicated for each metabolite stratified for sex. Metabolites in the unit μg/hour are described by their median;25^th^-75^th^ percentile. Within-sex differences were determined by Wilcoxon signed-rank test, and the corresponding *p* values are indicated.
(PDF)

S5 Table Correlation between day and nighttime urinary excretion (μg/hour) of steroid hormone metabolites in men and in women. The available number of participants is indicated for each metabolite stratified for sex. The correlation between day and nighttime excretion values was assessed by Spearman’s rank correlation coefficient ρ (rho). Rho values were ranked in ascending order within each sex group. Metabolites were colored by steroid groups as indicated.
(PDF)

S1 Text Statistical methods. The statistical methods applied are described in detail.
(PDF)
