# Correction: Reference intervals for the urinary steroid metabolome: The impact of sex, age, day and night time on human adult steroidogenesis

**DOI:** 10.1371/journal.pone.0253678

**Published:** 2021-06-17

**Authors:** Daniel Ackermann, Michael Groessl, Menno Pruijm, Belen Ponte, Geneviève Escher, Claudia H. d’Uscio, Idris Guessous, Georg Ehret, Antoinette Pechère-Bertschi, Pierre-Yves Martin, Michel Burnier, Bernhard Dick, Bruno Vogt, Murielle Bochud, Valentin Rousson, Nasser A. Dhayat

In Tables 2 and 3, the heading “Age group, years” should be “Age, years”. The values should be 20, 30, 40, 50, 60, 70, 80. Please see the correct Tables 2 and 3 here.

**Table 2:** Reference intervals for 40 steroid metabolites in 24-hour urine in men.

Metabolite, μg/24 hours	N	Age, years							CF to nmol/ 24 hours
		20	30	40	50	60	70	80	
17α-OH-pregnanolone	451	75.3–584	65.8–535	57.4–489	49.8–446	43–406	37–369	31.7–335	×2.990
pregnanetriol	395	442–2045	385–1814	334–1607	289–1421	250–1255	215–1106	185–974	×2.972
pregnenetriol	448	54–1213	87.4–1580	47–1127	22.3–769	11.4–559	6.7–441	4.7–380	×2.990
pregnanetriolone	457	6–70.8	6.2–78	6.4–86.3	6.6–96	6.8–108	7–121	7.2–138	×2.853
pregnanediol	457	85.5–653	94.4–708	81.1–625	64–516	55.4–459	52.8–441	52.8–441	×3.160
dehydroepiandrosterone	439	36.3–8455	63.7–56319	46.1–17754	24.3–2776	15.3–907	11–445	8.9–287	×3.467
16α-OH-dehydroepiandrosterone	448	75.2–1413	155–2139	75.7–1418	30.6–874	13.4–582	6.8–428	4.4–356	×3.285
androstenediol	453	35.4–1036	53.9–1878	42.9–1359	26–673	17–377	12–234	9–160	×3.444
androstenetriol	456	176–1652	214–1927	158–1518	110–1137	77–857	54.2–652	38.6–500	×3.264
testosterone	451	13.1–177	16.6–204	14.3–187	10.1–152	7.2–126	5.3–105	4–90.1	×3.467
5α-DH-testosterone	456	6–91.6	5.4–86.6	4.9–81.8	4.4–77.3	4–72.9	3.6–68.8	3.2–64.8	×3.444
androstanediol	445	44.1–228	49.6–252	42.4–221	34.6–186	28.1–156	22.7–130	18.2–108	×3.420
androsterone	381	1450–5821	1456–5839	1073–4644	759–3594	541–2810	390–2224	285–1786	×3.444
11β-OH-androsterone	447	357–1724	373–1771	376–1780	367–1752	345–1687	313–1589	273–1462	×3.264
etiocholanolone	390	883–5047	794–4743	673–4308	531–3770	385–3165	249–2531	138–1910	×3.444
17β-estradiol	457	0.7–4.5	0.9–5.5	1–6.1	1.1–6.3	1–5.9	0.9–5.6	0.9–5.6	×3.671
estriol	456	1.8–13.8	1.9–14.5	2–15.1	2.2–15.8	2.3–16.5	2.4–17.3	2.5–18.1	×3.467
TH-11-deoxycorticosterone	455	3.2–23.3	3.1–22.7	2.9–21.4	2.7–19.5	2.4–17.3	2.2–16.2	2.2–16.2	×2.990
TH-11-dehydrocorticosterone	452	49.1–277	49.5–278	46.6–267	40.7–242	32.8–209	28.8–191	28.8–191	×2.869
18-OH-TH-11-dehydrocorticosterone	433	14–259	15.1–297	15.7–318	15.7–318	15.1–297	14–259	12.5–212	×2.743
TH-corticosterone	457	52.6–326	58.3–350	59.8–356	57.1–345	50.5–317	48.6–309	60.1–357	×2.853
5α-TH-corticosterone	457	155–891	170–976	147–844	128–738	119–681	115–663	115–663	×2.853
TH-aldosterone	456	5.1–79.2	5.4–84	5.5–85.6	5.4–83.9	5.1–79.1	4.6–71.7	4–62.5	×2.743
TH-11-deoxycortisol	457	27.5–145	28.4–152	29.4–160	30.4–168	31.5–176	32.6–185	33.8–194	×2.853
cortisol	457	41.6–243	47.1–282	50–302	49.7–300	47.3–282	48.4–291	54.5–335	×2.759
6β-OH-cortisol	457	35.3–303	35.3–303	35.3–303	35.3–303	35.3–303	35.3–303	35.3–303	×2.642
18-OH-cortisol	424	40.7–638	40.7–638	40.7–638	40.7–638	40.7–638	40.7–638	40.7–638	×2.642
20α-DH-cortisol	457	19.2–136	19.2–136	19.2–136	19.2–136	19.2–136	19.2–136	19.2–136	×2.743
TH-cortisol	371	714–2724	866–3301	966–3682	992–3781	938–3575	918–3501	1047–3992	×2.729
α-cortol	452	127–519	157–638	161–658	164–668	169–688	176–717	186–757	×2.714
β-cortol	453	213–1171	213–1171	213–1171	213–1171	213–1171	213–1171	213–1171	×2.714
11β-OH-etiocholanolone	455	29.7–955	15.1–861	27.7–944	43.7–1029	55–1081	59.1–1099	59.1–1099	×3.264
5α-TH-cortisol	381	524–3424	631–3992	578–3715	527–3436	497–3278	488–3226	488–3226	×2.729
cortisone	456	69.2–378	74–404	77–420	77.9–425	76.6–418	73.4–401	68.3–373	×2.774
20α-DH-cortisone	457	11.1–63.3	11.1–63.3	11.1–63.3	11.1–63.3	11.1–63.3	11.1–63.3	11.1–63.3	×2.759
20β-DH-cortisone	457	21.8–127	22.7–131	23.6–135	24.5–140	25.5–145	26.5–149	27.5–154	×2.759
TH-cortisone	407	1543–6080	1643–6378	1667–6449	1611–6285	1483–5902	1297–5332	1072–4623	×2.743
α-cortolone	426	552–2214	587–2338	611–2420	622–2456	618–2443	600–2382	570–2277	×2.729
β-cortolone	427	366–1387	349–1328	332–1271	315–1216	300–1164	285–1114	271–1065	×2.729
11-keto-etiocholanolone	455	97.7–1055	67.3–884	85–986	103–1082	109–1113	102–1076	83–975	×3.285

Reference intervals have been estimated by the described statistical models and are given as 2.5^th^-97.5^th^ percentiles in the unit μg/24 hours for different ages. N represents the sample number per analyte included in the statistical model. CF: Conversion Factor.

**Table 3:** Reference intervals for 40 steroid metabolites in 24-hour urine in women.

Metabolite, μg/24 hours	N	Age, years							CF to nmol/ 24 hours
		20	30	40	50	60	70	80	
17α-OH-pregnanolone	375	18.2–383	28.4–704	27.5–672	16.5–337	9.7–166	8.2–132	8.2–132	×2.990
pregnanetriol	360	173–1156	216–1346	200–1275	135–975	80.9–696	56.8–554	48.3–501	×2.972
pregnenetriol	378	18.7–810	12–661	7.4–535	4.3–429	2.4–341	1.2–268	0.5–207	×2.990
pregnanetriolone	379	4.1–95.7	3–54.5	3.1–57.9	3.4–67	3.6–77.4	3.9–89.4	4.2–103	×2.853
pregnanediol	376	62.9–2180	95.2–5269	94.6–5192	61.7–2101	38.2–830	29.5–520	27.5–460	×3.160
dehydroepiandrosterone	377	13.8–1623	15.6–1989	13.6–1578	9.1–834	5.7–409	4.1–242	3.2–168	×3.467
16α-OH-dehydroepiandrosterone	379	37.7–1742	33.9–1565	25–1157	15.2–704	8.9–411	5.8–270	4.3–199	×3.285
androstenediol	378	22.5–873	16.8–571	12.6–380	9.5–257	7.2–177	5.6–123	4.3–86.5	×3.444
androstenetriol	378	99.7–1351	72–1051	51.4–813	36.2–625	25.2–476	17.3–361	11.7–271	×3.264
testosterone	367	2.4–55.1	2.7–66.1	2.6–61.8	2.1–45.3	1.6–30.9	1.2–22.8	1.1–18.3	×3.467
5α-DH-testosterone	377	2.9–75.6	2.5–66.9	2.2–59.1	2–52.3	1.8–46.3	1.5–41	1.4–36.2	×3.444
androstanediol	372	8.9–106	10.5–124	9.7–114	7–83.1	4.9–57.7	4–46.7	3.7–44	×3.420
androsterone	349	480–4482	414–3978	307–3138	194–2181	116–1462	75.6–1051	54.3–816	×3.444
11β-OH-androsterone	376	192–1183	192–1183	192–1183	192–1183	192–1183	192–1183	192–1183	×3.264
etiocholanolone	351	543–3870	448–3483	345–3031	243–2540	153–2037	82.1–1551	33.9–1106	×3.444
17β-estradiol	377	0.3–7.6	0.7–26.6	0.9–35.8	0.5–17	0.3–7.1	0.2–5.4	0.2–5.4	×3.671
estriol	374	0.8–36.3	1.6–91.8	1.7–99.5	1–45.3	0.6–19.5	0.5–15	0.5–15	×3.467
TH-11-deoxycorticosterone	378	1.8–34.2	2.5–66.1	2.6–69.5	1.9–39	1.4–21.5	1.2–17.9	1.2–17.9	×2.990
TH-11-dehydrocorticosterone	379	31.5–240	29.5–228	27.6–216	25.9–205	24.3–194	22.7–184	21.2–174	×2.869
18-OH-TH-11-dehydrocorticosterone	342	10–262	10–262	10–262	10–262	10–262	10–262	10–262	×2.743
TH-corticosterone	379	28–226	34.8–261	39.7–285	41.7–294	40.4–288	36.2–268	29.7–235	×2.853
5α-TH-corticosterone	379	60.6–556	57–535	53.5–514	50.2–493	47–474	44.1–454	41.2–436	×2.853
TH-aldosterone	378	5.3–83	5.6–86.3	5.5–84.9	5.1–78.8	4.5–69.1	3.7–57.3	2.9–44.9	×2.743
TH-11-deoxycortisol	379	15–97.3	17.6–114	19.8–128	21.4–138	22.2–144	22.1–143	21.1–137	×2.853
cortisol	379	33.7–250	33.7–250	33.7–250	33.7–250	33.7–250	33.7–250	33.7–250	×2.759
6β-OH-cortisol	378	25.6–282	25.6–282	25.6–282	25.6–282	25.6–282	25.6–282	25.6–282	×2.642
18-OH-cortisol	344	32.4–516	32.4–516	32.4–516	32.4–516	32.4–516	32.4–516	32.4–516	×2.642
20α-DH-cortisol	379	16.7–176	16.2–169	15.6–163	15.2–156	14.7–150	14.2–144	13.8–138	×2.743
TH-cortisol	340	336–1677	416–2015	490–2319	550–2560	589–2714	603–2766	588–2711	×2.729
α-cortol	379	92.5–464	95.1–479	97.8–495	101–511	103–528	106–546	109–564	×2.714
β-cortol	378	125–751	125–751	125–751	125–751	125–751	125–751	125–751	×2.714
11β-OH-etiocholanolone	378	0.5–612	12.2–756	28.4–860	39.1–916	39.3–917	28.9–863	12.7–760	×3.264
5α-TH-cortisol	362	177–1982	177–1982	177–1982	177–1982	177–1982	177–1982	177–1982	×2.729
cortisone	379	48.3–308	56.3–360	59.4–379	56.7–362	50.2–321	47.9–306	47.9–306	×2.774
20α-DH-cortisone	379	8.6–52.3	8.7–53.1	8.3–49.9	7.4–43.7	6.4–36.5	6–34.2	6–34.2	×2.759
20β-DH-cortisone	379	23.9–145	22.5–136	21.2–129	20–121	18.8–114	17.8–107	16.7–101	×2.759
TH-cortisone	360	900–4654	900–4654	900–4654	900–4654	900–4654	900–4654	900–4654	×2.743
α-cortolone	362	405–1932	405–1932	405–1932	405–1932	405–1932	405–1932	405–1932	×2.729
β-cortolone	369	164–854	164–854	164–854	164–854	164–854	164–854	164–854	×2.729
11-keto-etiocholanolone	379	23.6–652	43.4–778	60.8–870	70.9–918	71–919	61–871	43.6–779	×3.285

Reference intervals have been estimated by the described statistical models and are given as 2.5^th^-97.5^th^ percentiles in the unit μg/24 hours for different ages. N represents the sample number per analyte included in the statistical model. CF: Conversion Factor.
