# Supplementary material for: Reference intervals for the urinary steroid metabolome: The impact of sex, age, day and night time on human adult steroidogenesis
Source: PLoS One. 2019 Mar 29;14(3):e0214549. doi: 10.1371/journal.pone.0214549 (PMC6440635; doi:10.1371/journal.pone.0214549)
Supplement: S1 Fig — Calibration curves are shown for all analytes injected on column in the range from 39 to 20000 fmol. Crosses indicate data points, the solid line represents the linear regression curve and the dotted lines the 95% confidence interval. Axes are plotted in logarithmic scale (base 10). (PDF) [file pone.0214549.s001.pdf]

Supporting Figure 1. Calibration curves. Calibration curves are shown for all analytes injected on column in the range from 39 to 20000 fmol. Crosses indicate data points, the solid line represents the linear regression curve and the dotted lines the 95% confidence interval. Axes are plotted in logarithmic scale (base 10).

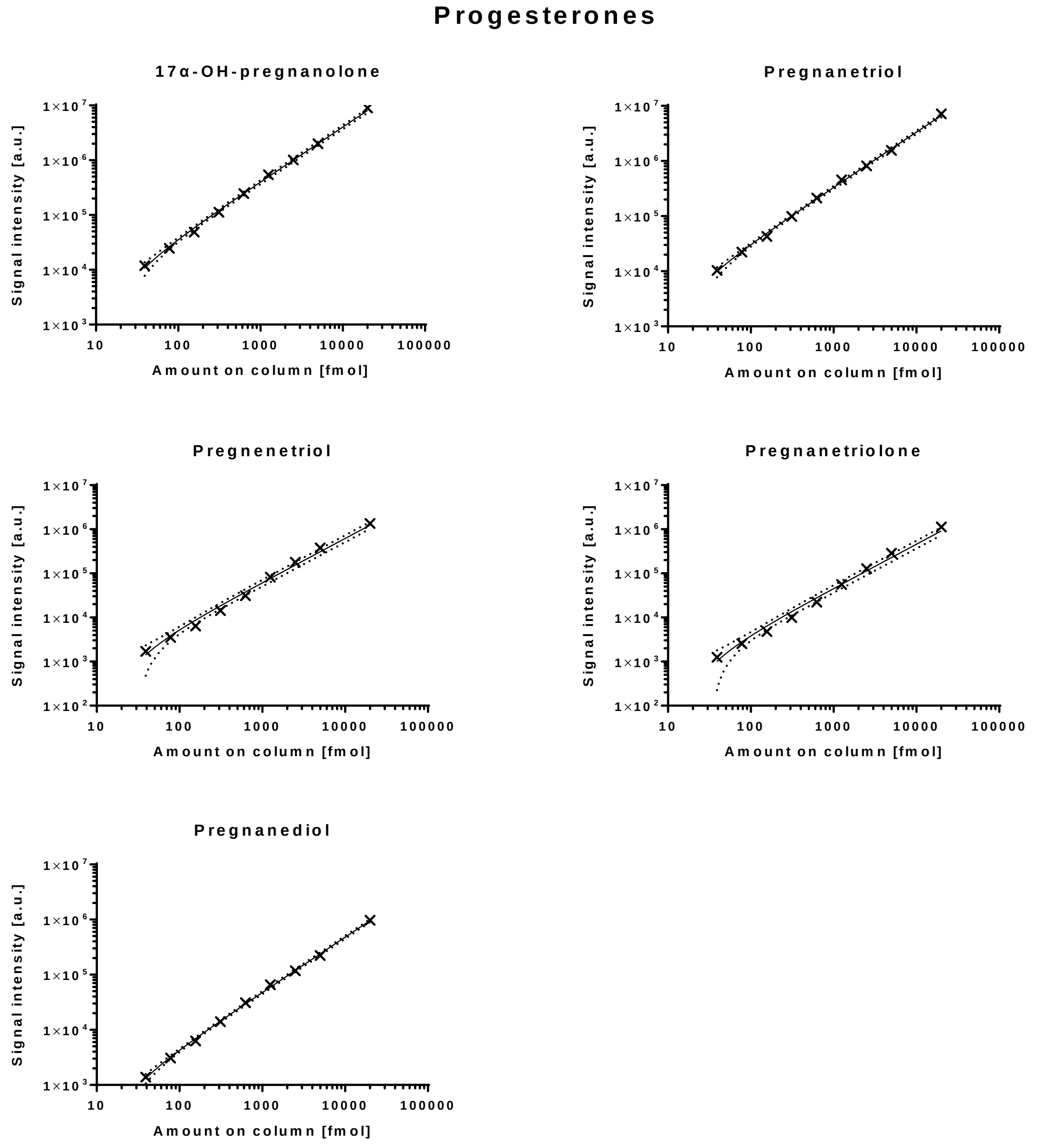

Androgens

Dehydroepiandrosterone

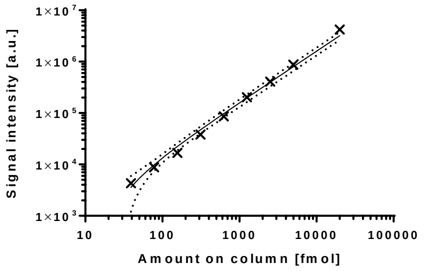

16α-OH-dehydroepiandrosterone

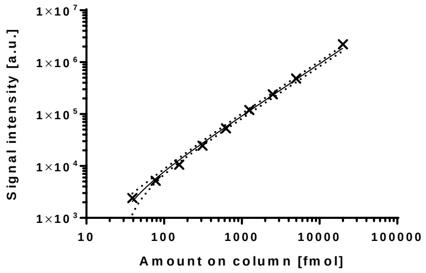

Androstenediol

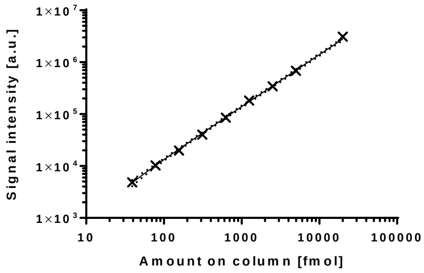

Androstenetriol

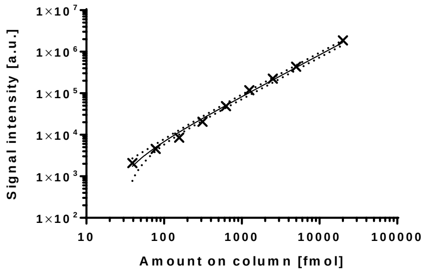

Testosterone

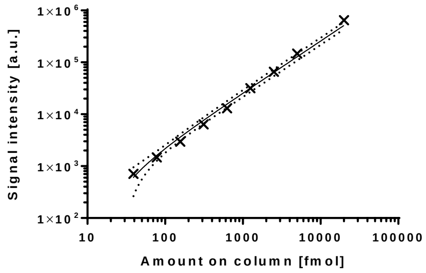

5α-DH-testosterone

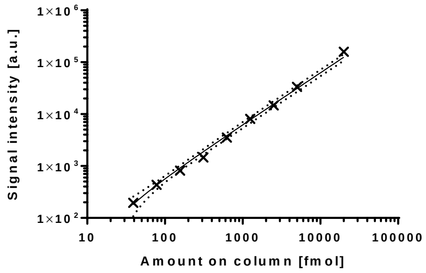

Androstanediol

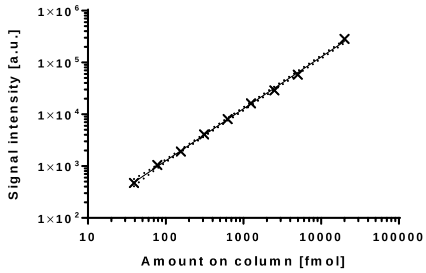

Androsterone

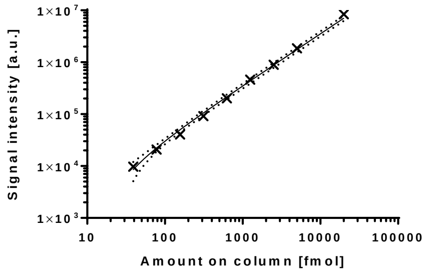

11β-OH-androsterone

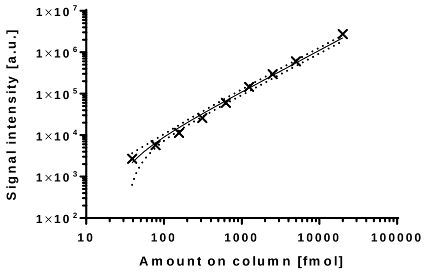

Etiocholanolone

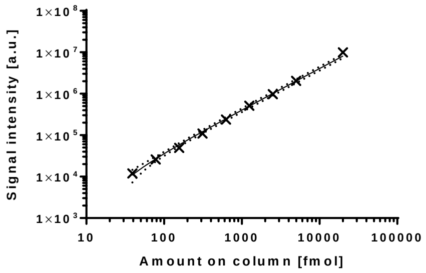

Estrogens

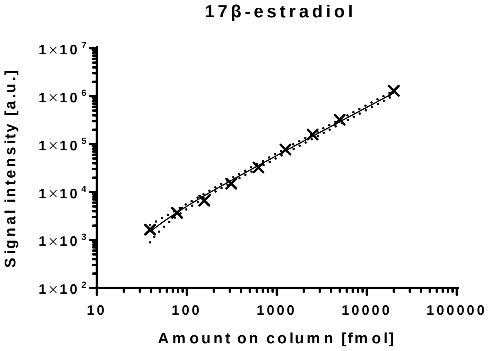
17β-estradiol
Signal intensity [a.u.]
1×10^7
1×10^6
1×10^5
1×10^4
1×10^3
1×10^2
10
100
1000
10000
100000
Amount on column [fmol]

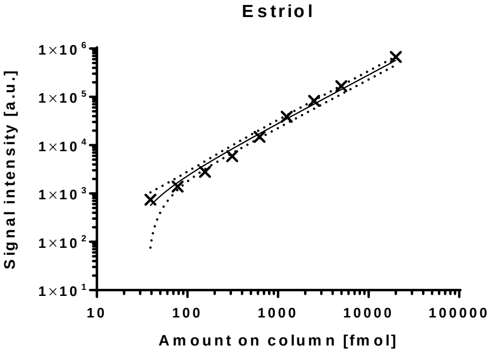
Estriol
Signal intensity [a.u.]
1×10^6
1×10^5
1×10^4
1×10^3
1×10^2
1×10^1
10
100
1000
10000
100000
Amount on column [fmol]

Corticosterones

TH-11-deoxycorticosterone

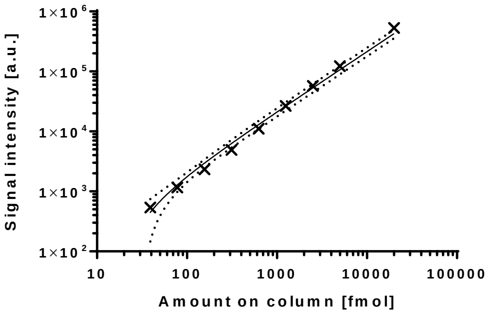

TH-11-dehydrocorticosterone

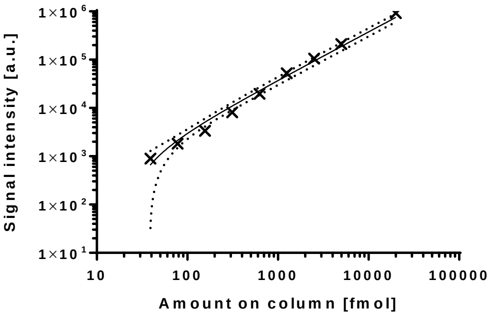

18-OH-TH-11-dehydrocorticosterone

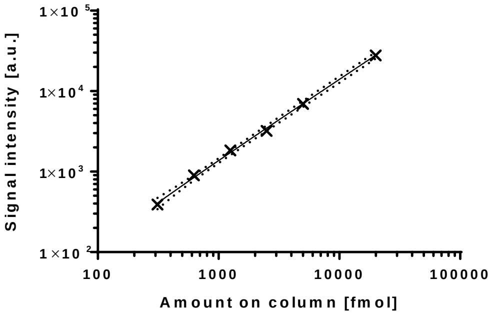

TH-corticosterone

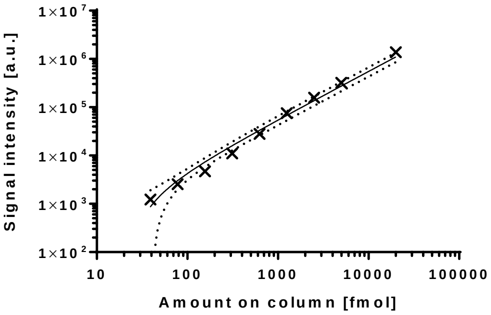

5α-TH-corticosterone

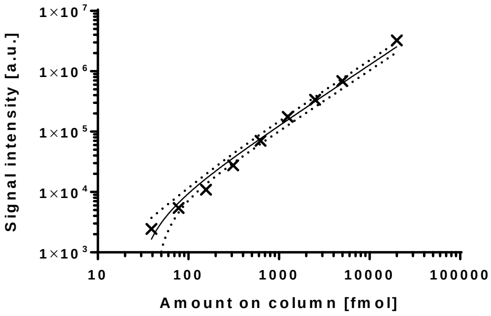

Mineralocorticoids

TH-aldosterone

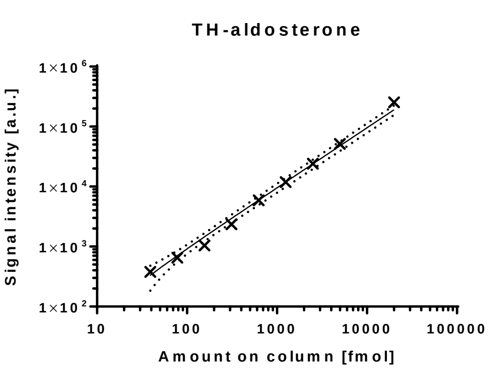

TH-11-deoxycortisol

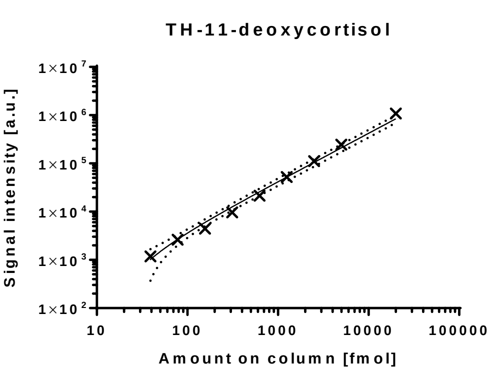

Glucocorticoids

Cortisol

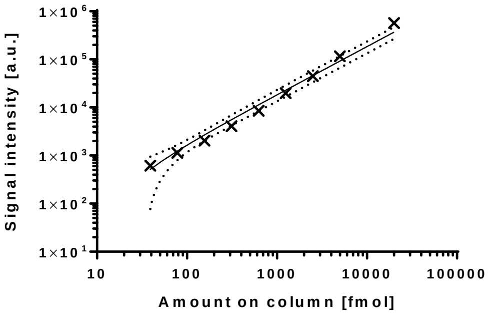

6β-OH-cortisol

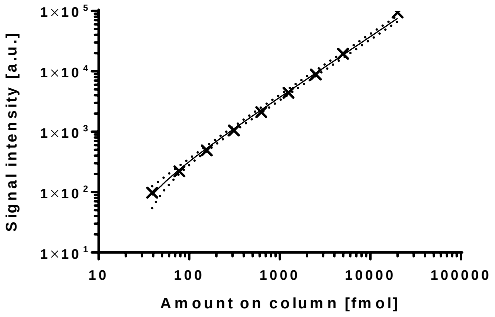

18-OH-cortisol

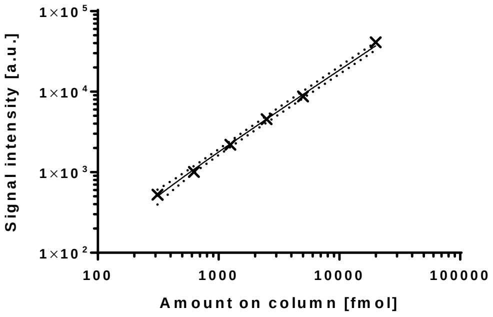

20α-DH-cortisol

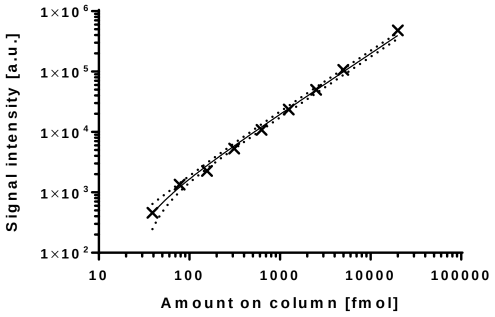

TH-cortisol

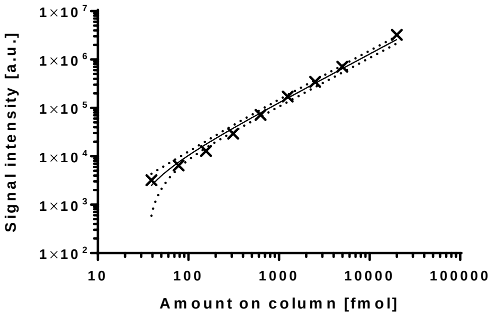

α-cortol

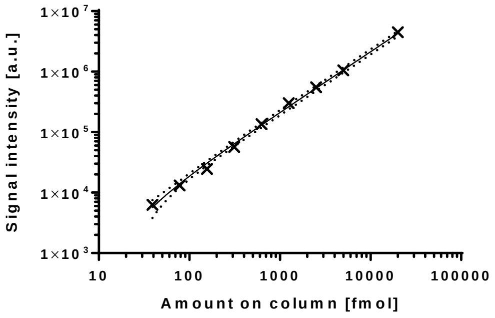

β-cortol

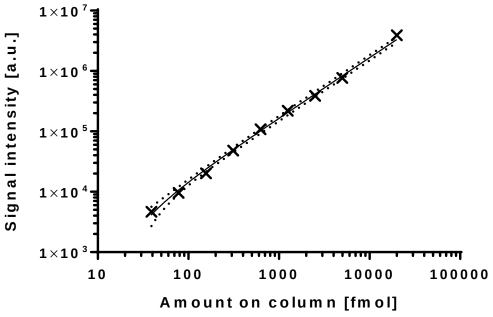

11β-OH-etiocholanolone

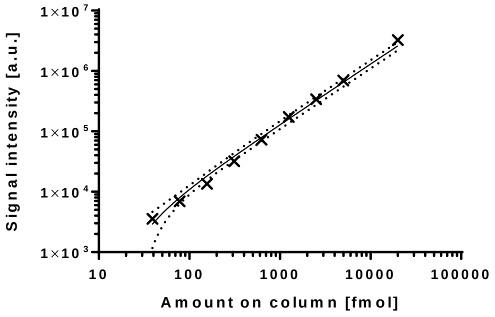

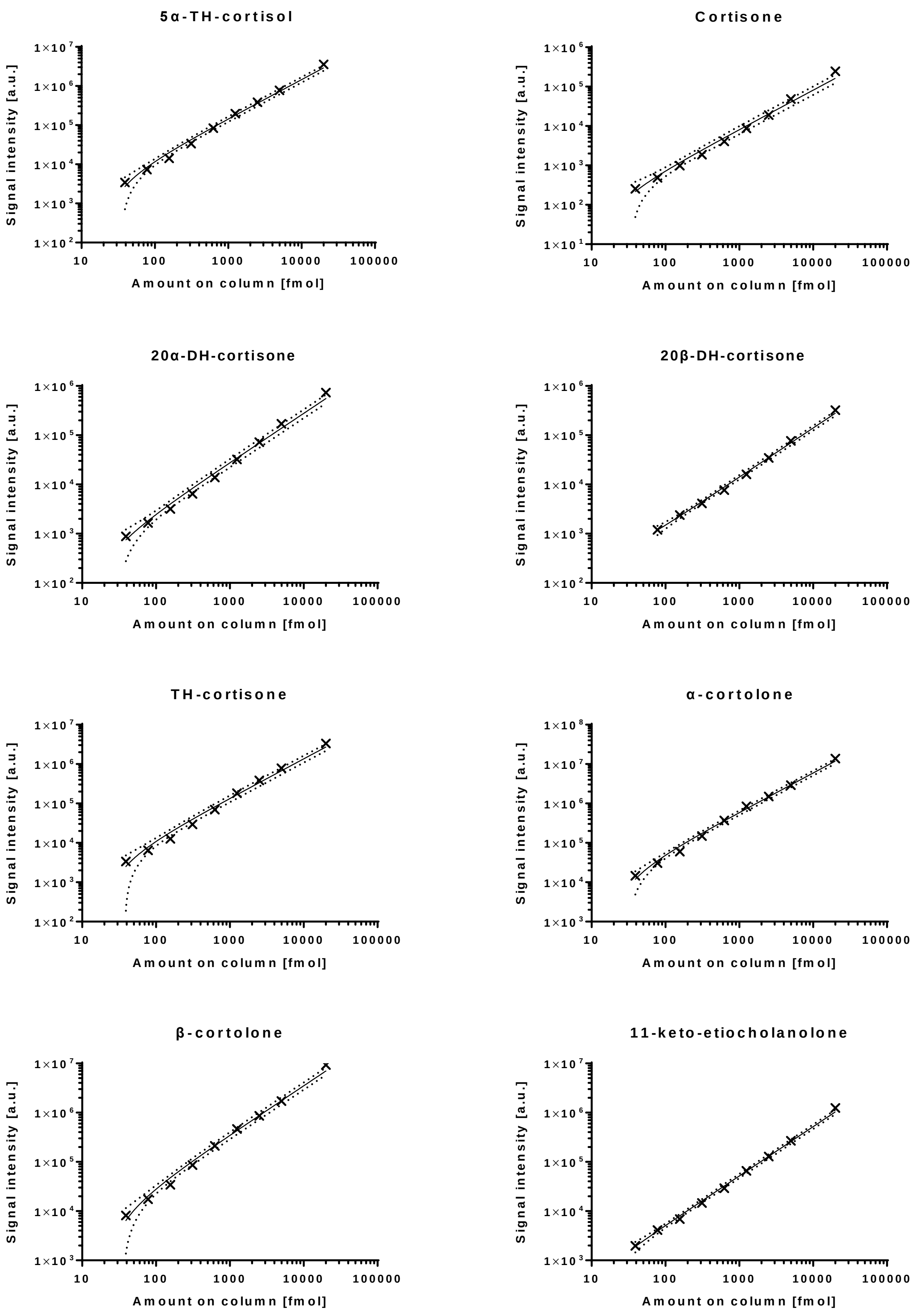
5α-TH-cortisol
Cortisone
20α-DH-cortisone
20β-DH-cortisone
TH-cortisone
α-cortolone
β-cortolone
11-keto-etiocholanolone
Signal intensity [a.u.]
Amount on column [fmol]
10
100
1000
10000
100000
