# Supplementary material for: Reference intervals for the urinary steroid metabolome: The impact of sex, age, day and night time on human adult steroidogenesis
Source: PLoS One. 2019 Mar 29;14(3):e0214549. doi: 10.1371/journal.pone.0214549 (PMC6440635; doi:10.1371/journal.pone.0214549)
Supplement: S2 Fig — A plot of the sum of ion abundances for the selected compound-specific ions on the vertical axis versus the retention time of each characteristic ion on the horizontal axis simultaneously obtained by a GC-MS analysis of urinary steroid derivatives is shown. Multiple chromatographic peaks indicate the elution of numerous individual steroid compounds. By knowing the retention time for a given steroid indicated in the table, the members of the urinary steroid profile can be distinguished. Some steroid compounds are labeled. QIon: quantifier ion [m/z]. RT: retention time [min]. (PDF) [file pone.0214549.s002.pdf]

Supporting Figure 2. Example of a selected-ion monitoring chromatogram. A plot of the sum of ion abundances for the selected compound-specific ions on the vertical axis versus the retention time of each characteristic ion on the horizontal axis simultaneously obtained by a GC-MS analysis of urinary steroid derivatives is shown. Multiple chromatographic peaks indicate the elution of numerous individual steroid compounds. By knowing the retention time for a given steroid indicated in the table, the members of the urinary steroid profile can be distinguished. Some steroid compounds are labeled. QIon: quantifier ion [m/z]. RT: retention time [min].

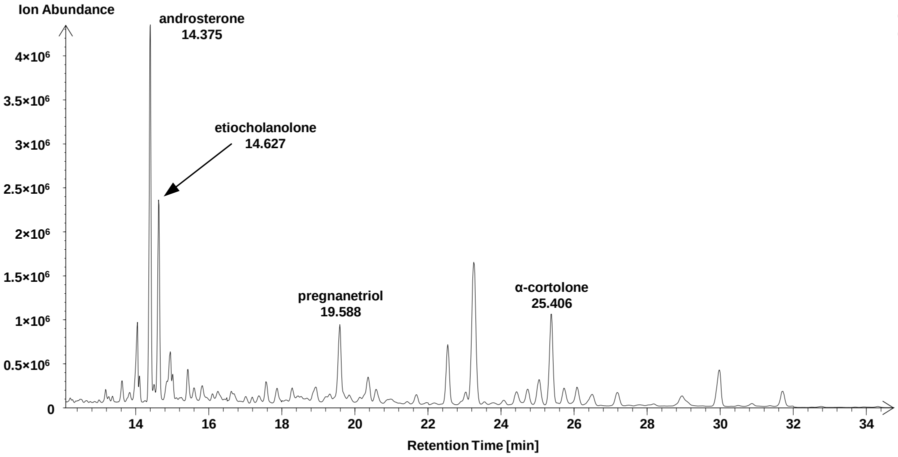

Compound Name	QIon	RT	Compound Name	QIon	RT
androsterone	270	14.375	pregnenetriol	433	23.017
androstanediol	331	14.645	TH-cortisone	578	23.315
etiocholanolone	270	14.627	TH-11-dehydrocorticosterone	490	23.586
dehydroepiandrosterone	268	15.429	TH-corticosterone	564	24.139
androstenediol	239	15.859	5α-TH-corticosterone	564	24.491
5α-DH-testosterone	391	16.013	TH-cortisol	652	24.766
11-keto-etiocholanolone	269	16.107	5α-TH-cortisol	652	25.056
17β-estradiol	416	16.521	α-cortolone	449	25.406
testosterone	389	16.719	TH-aldosterone	506	25.515
17α-OH-pregnanolone	476	17.580	18-OH-TH-11-dehydrocorticosterone	457	26.036
11β-OH-androsterone	268	17.580	β-cortol	343	26.046
11β-OH-etiocholanolone	268	17.896	β-cortolone	449	26.056
16α-OH-dehydroepiandrosterone	266	18.311	α-cortol	343	27.202
pregnanediol	269	18.972	cortisone	531	29.022
pregnanetriol	435	19.588	cortisol	605	30.763
androstenetriol	432	20.589	20β-DH-cortisone	402	30.857
TH-11-deoxycortisol	564	20.972	20α-DH-cortisone	402	31.560
TH-11-deoxycorticosterone	476	21.234	6β-OH-cortisol	513	31.998
estriol	504	21.285	18-OH-cortisol	344	31.975
pregnanetriolone	449	21.971	20α-DH-cortisol	296	32.770
