# Supplementary material for: Reference intervals for the urinary steroid metabolome: The impact of sex, age, day and night time on human adult steroidogenesis
Source: PLoS One. 2019 Mar 29;14(3):e0214549. doi: 10.1371/journal.pone.0214549 (PMC6440635; doi:10.1371/journal.pone.0214549)
Supplement: S3 Fig — A descriptive analysis of the 40 steroid compounds measured in urine is shown including one steroid compound per page. Panel (a): boxplots, steroid (log-scale) by sex; Panel (b): Gasser-Müller nonparametric fits and scatter plots, steroid (log-scale) by age and by sex; Panels (c) and (d): boxplots, transformed steroid for men and women according to optimal power transformation; outliers are plotted as black dots; Panels (e) and (f): steroid (log-scale) by day and night time for men and women; Panels (g) and (h): Spearman correlation of transformed steroid night vs day. Abbreviations used: M = men; W = women; D = day; N = night; TR = optimal power transformation; nout = number of outliers; sk = skewness; ku = kurtosis; delta = robust estimate of mean difference expressed in standard deviations; rho = Spearman correlation coefficient; n = sample size. (PDF) [file pone.0214549.s003.pdf]

Supporting Figure 3. Descriptive analysis of steroid steroid compounds. A descriptive analysis of the 40 steroid compounds measured in urine is shown including one steroid compound per page.

Panel (a): boxplots, steroid (log-scale) by sex;

Panel (b): Gasser-Müller nonparametric fits and scatter plots, steroid (log-scale) by age and by sex;

Panels (c) and (d): boxplots, transformed steroid for men and women according to optimal power transformation; outliers are plotted as black dots;

Panels (e) and (f): steroid (log-scale) by day and night time for men and women;

Panels (g) and (h): Spearman correlation of transformed steroid night vs day.

Abbreviations used:

M=men; W=women; D=day; N=night; TR=optimal power transformation; nout=number of outliers; sk=skewness; ku=kurtosis; delta=robust estimate of mean difference expressed in standard deviations; rho=Spearman correlation coefficient; n=sample size.

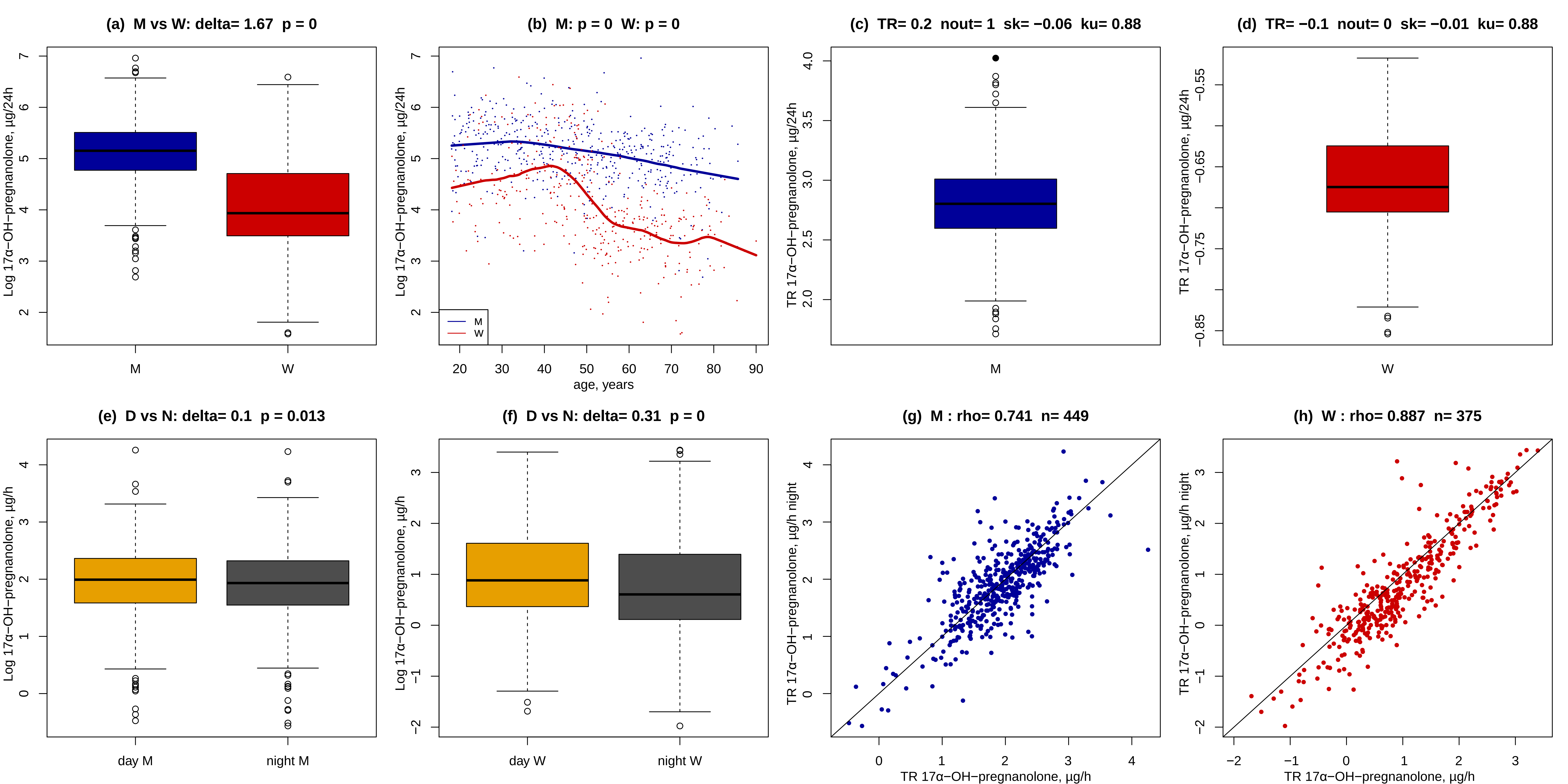

(a) M vs W: delta= 1.67 p = 0
Log 17α−OH−pregnanolone, µg/24h
M
W
(b) M: p = 0 W: p = 0
Log 17α−OH−pregnanolone, µg/24h
M
W
age, years
(c) TR= 0.2 nout= 1 sk= −0.06 ku= 0.88
TR 17α−OH−pregnanolone, µg/24h
M
(d) TR= −0.1 nout= 0 sk= −0.01 ku= 0.88
TR 17α−OH−pregnanolone, µg/24h
W
(e) D vs N: delta= 0.1 p = 0.013
Log 17α−OH−pregnanolone, µg/h
day M
night M
(f) D vs N: delta= 0.31 p = 0
Log 17α−OH−pregnanolone, µg/h
day W
night W
(g) M : rho= 0.741 n= 449
TR 17α−OH−pregnanolone, µg/h night
TR 17α−OH−pregnanolone, µg/h
(h) W : rho= 0.887 n= 375
TR 17α−OH−pregnanolone, µg/h night
TR 17α−OH−pregnanolone, µg/h

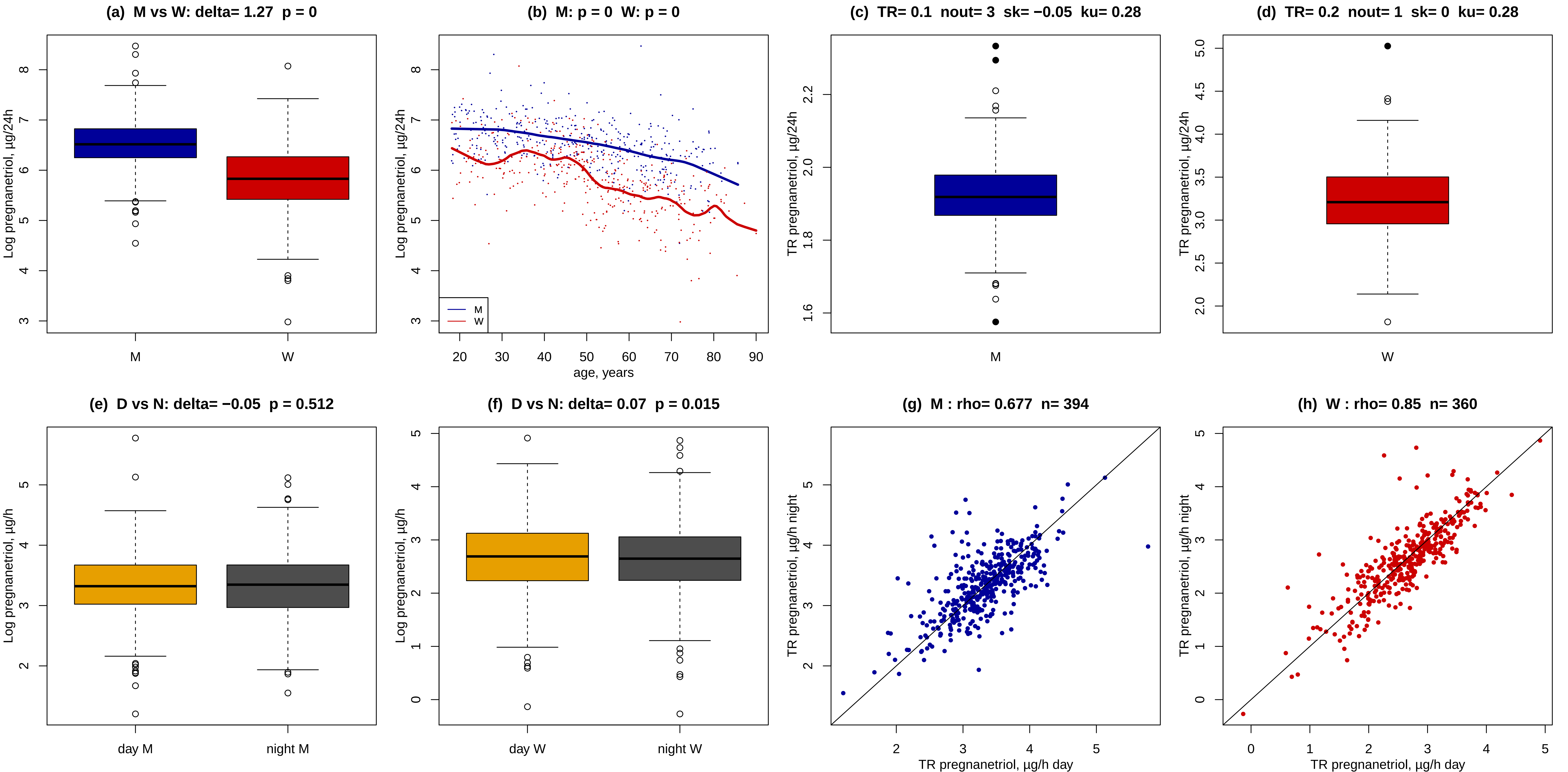
(a) M vs W: delta= 1.27 p = 0
(b) M: p = 0 W: p = 0
(c) TR= 0.1 nout= 3 sk= −0.05 ku= 0.28
(d) TR= 0.2 nout= 1 sk= 0 ku= 0.28
(e) D vs N: delta= −0.05 p = 0.512
(f) D vs N: delta= 0.07 p = 0.015
(g) M : rho= 0.677 n= 394
(h) W : rho= 0.85 n= 360
Log pregnanetriol, µg/24h
TR pregnanetriol, µg/24h
Log pregnanetriol, µg/h
TR pregnanetriol, µg/h night
TR pregnanetriol, µg/h day
age, years
M
W
day M
night M
day W
night W

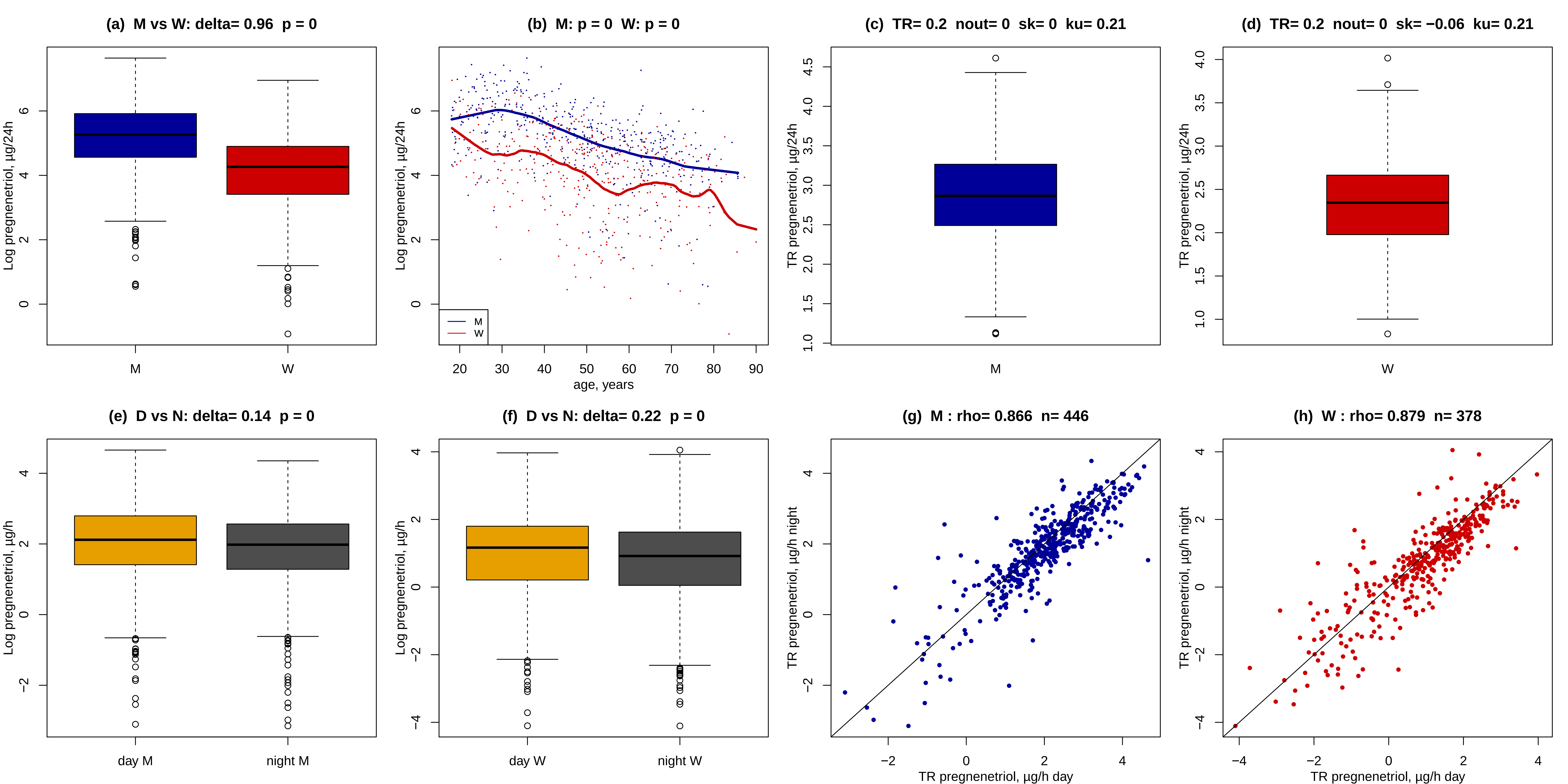
(a) M vs W: delta= 0.96 p = 0
Log pregnenetriol, µg/24h
M
W
(b) M: p = 0 W: p = 0
Log pregnenetriol, µg/24h
age, years
M
W
(c) TR= 0.2 nout= 0 sk= 0 ku= 0.21
TR pregnenetriol, µg/24h
M
(d) TR= 0.2 nout= 0 sk= −0.06 ku= 0.21
TR pregnenetriol, µg/24h
W
(e) D vs N: delta= 0.14 p = 0
Log pregnenetriol, µg/h
day M
night M
(f) D vs N: delta= 0.22 p = 0
Log pregnenetriol, µg/h
day W
night W
(g) M : rho= 0.866 n= 446
TR pregnenetriol, µg/h night
TR pregnenetriol, µg/h day
(h) W : rho= 0.879 n= 378
TR pregnenetriol, µg/h night
TR pregnenetriol, µg/h day

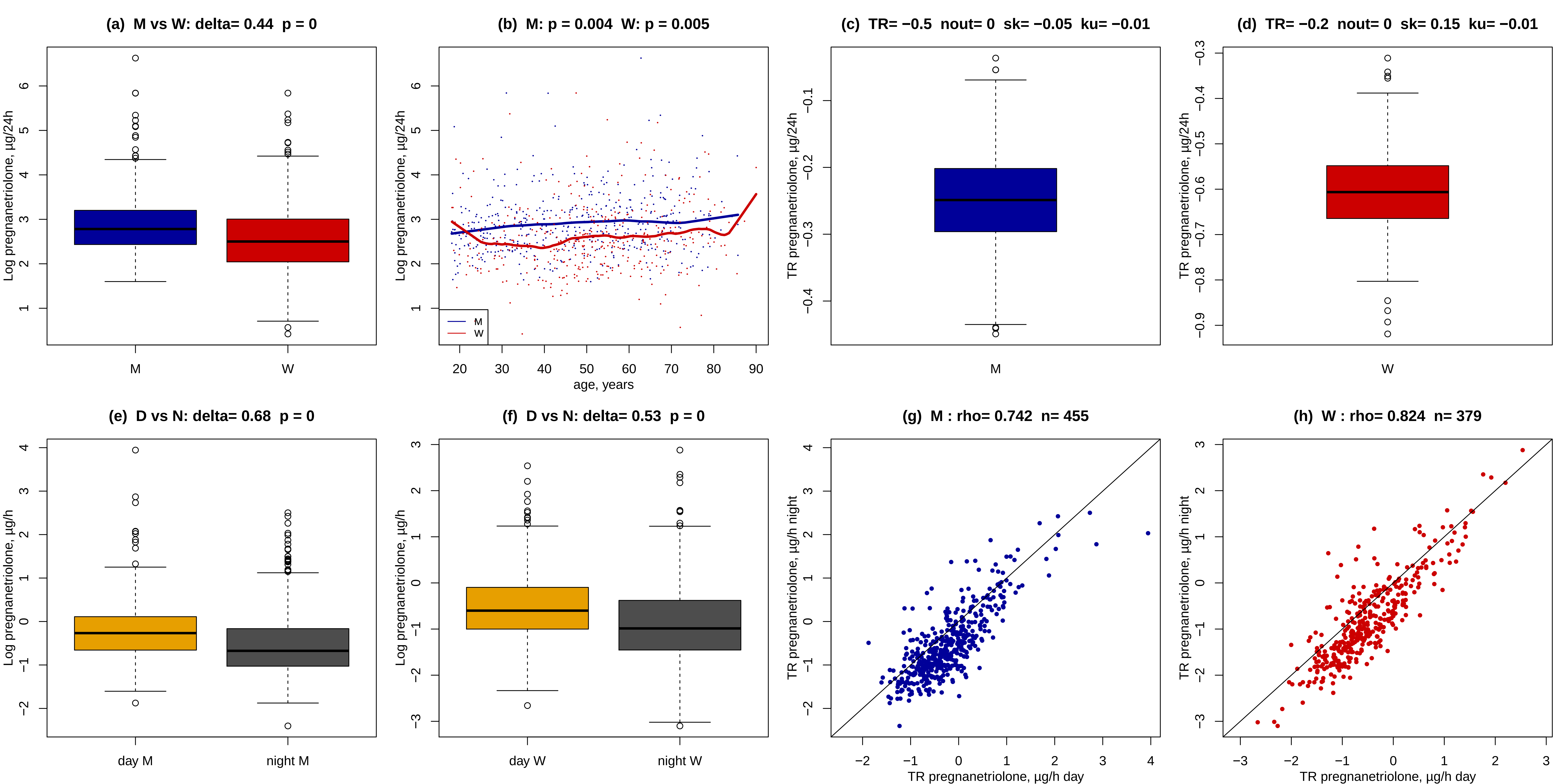
(a) M vs W: delta= 0.44 p = 0
Log pregnanetriolone, µg/24h
M
W
(b) M: p = 0.004 W: p = 0.005
Log pregnanetriolone, µg/24h
age, years
M
W
(c) TR= −0.5 nout= 0 sk= −0.05 ku= −0.01
TR pregnanetriolone, µg/24h
M
(d) TR= −0.2 nout= 0 sk= 0.15 ku= −0.01
TR pregnanetriolone, µg/24h
W
(e) D vs N: delta= 0.68 p = 0
Log pregnanetriolone, µg/h
day M
night M
(f) D vs N: delta= 0.53 p = 0
Log pregnanetriolone, µg/h
day W
night W
(g) M : rho= 0.742 n= 455
TR pregnanetriolone, µg/h night
TR pregnanetriolone, µg/h day
(h) W : rho= 0.824 n= 379
TR pregnanetriolone, µg/h night
TR pregnanetriolone, µg/h day

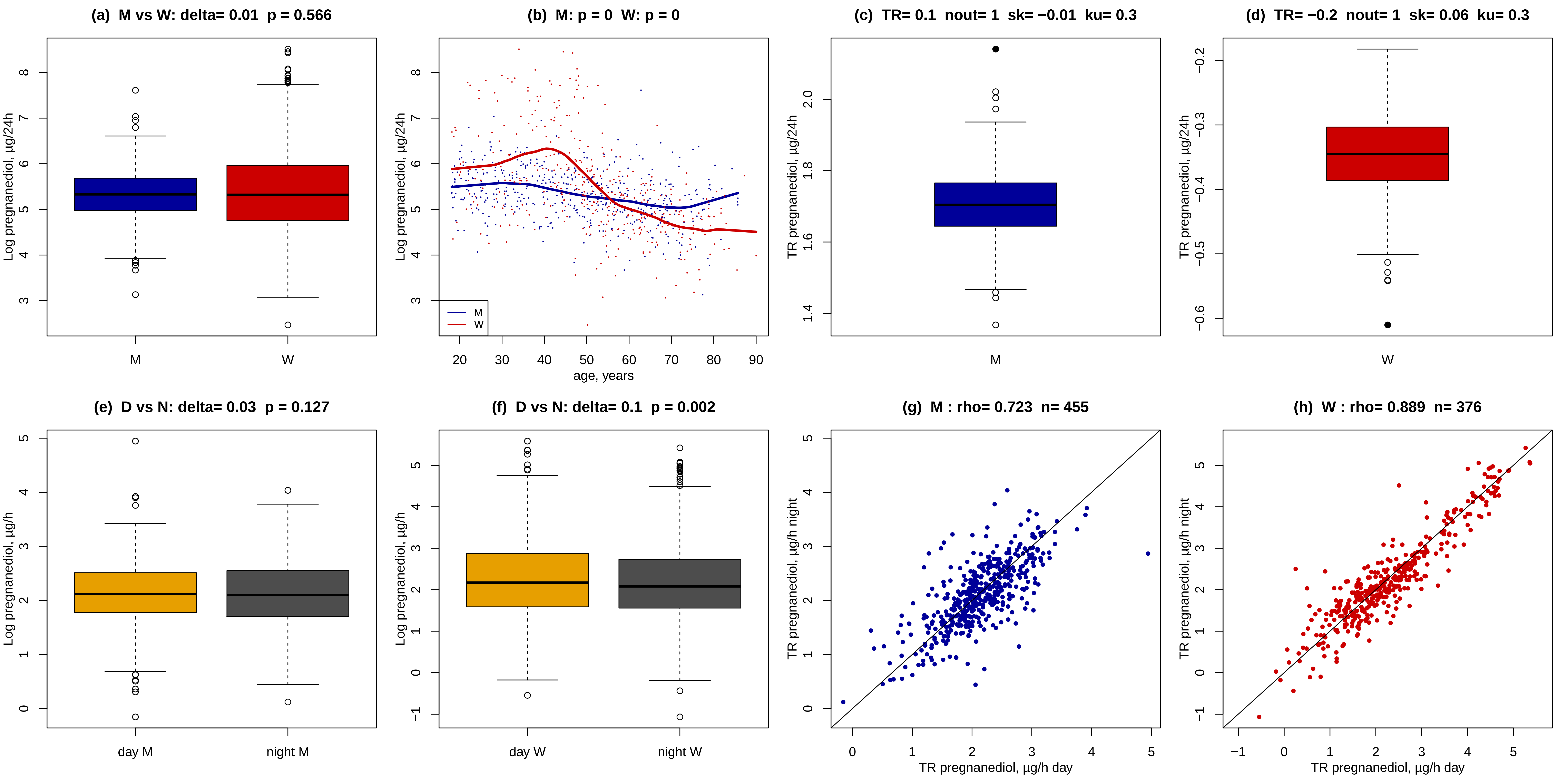
(a) M vs W: delta= 0.01 p = 0.566
Log pregnanediol, µg/24h
M
W
(b) M: p = 0 W: p = 0
Log pregnanediol, µg/24h
age, years
M
W
(c) TR= 0.1 nout= 1 sk= −0.01 ku= 0.3
TR pregnanediol, µg/24h
M
(d) TR= −0.2 nout= 1 sk= 0.06 ku= 0.3
TR pregnanediol, µg/24h
W
(e) D vs N: delta= 0.03 p = 0.127
Log pregnanediol, µg/h
day M
night M
(f) D vs N: delta= 0.1 p = 0.002
Log pregnanediol, µg/h
day W
night W
(g) M : rho= 0.723 n= 455
TR pregnanediol, µg/h night
TR pregnanediol, µg/h day
(h) W : rho= 0.889 n= 376
TR pregnanediol, µg/h night
TR pregnanediol, µg/h day

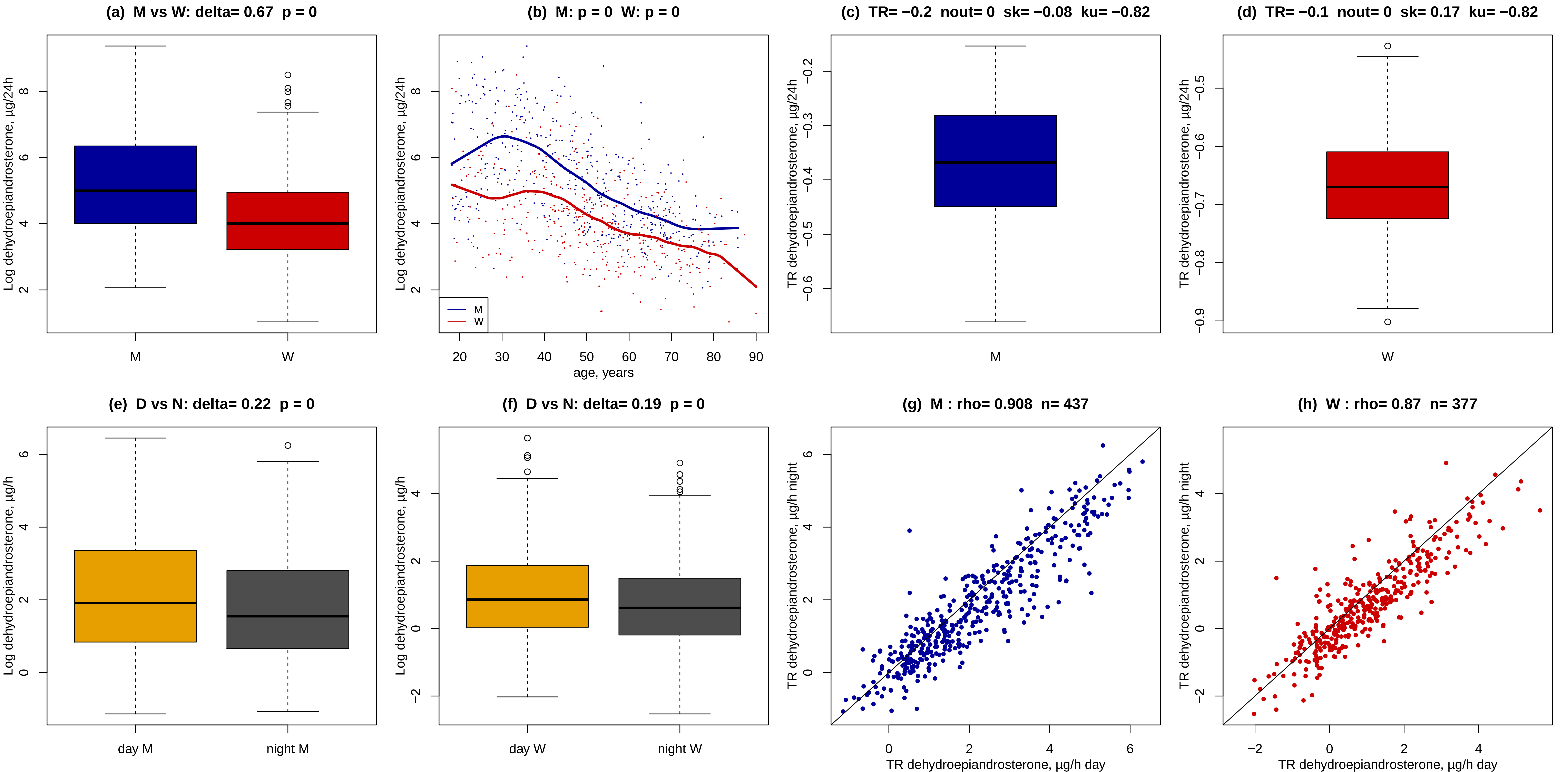

(a) M vs W: delta= 0.67 p = 0
(b) M: p = 0 W: p = 0
(c) TR= −0.2 nout= 0 sk= −0.08 ku= −0.82
(d) TR= −0.1 nout= 0 sk= 0.17 ku= −0.82
(e) D vs N: delta= 0.22 p = 0
(f) D vs N: delta= 0.19 p = 0
(g) M : rho= 0.908 n= 437
(h) W : rho= 0.87 n= 377
Log dehydroepiandrosterone, µg/24h
TR dehydroepiandrosterone, µg/24h
Log dehydroepiandrosterone, µg/h
TR dehydroepiandrosterone, µg/h night
TR dehydroepiandrosterone, µg/h day
age, years
M
W
day M
night M
day W
night W

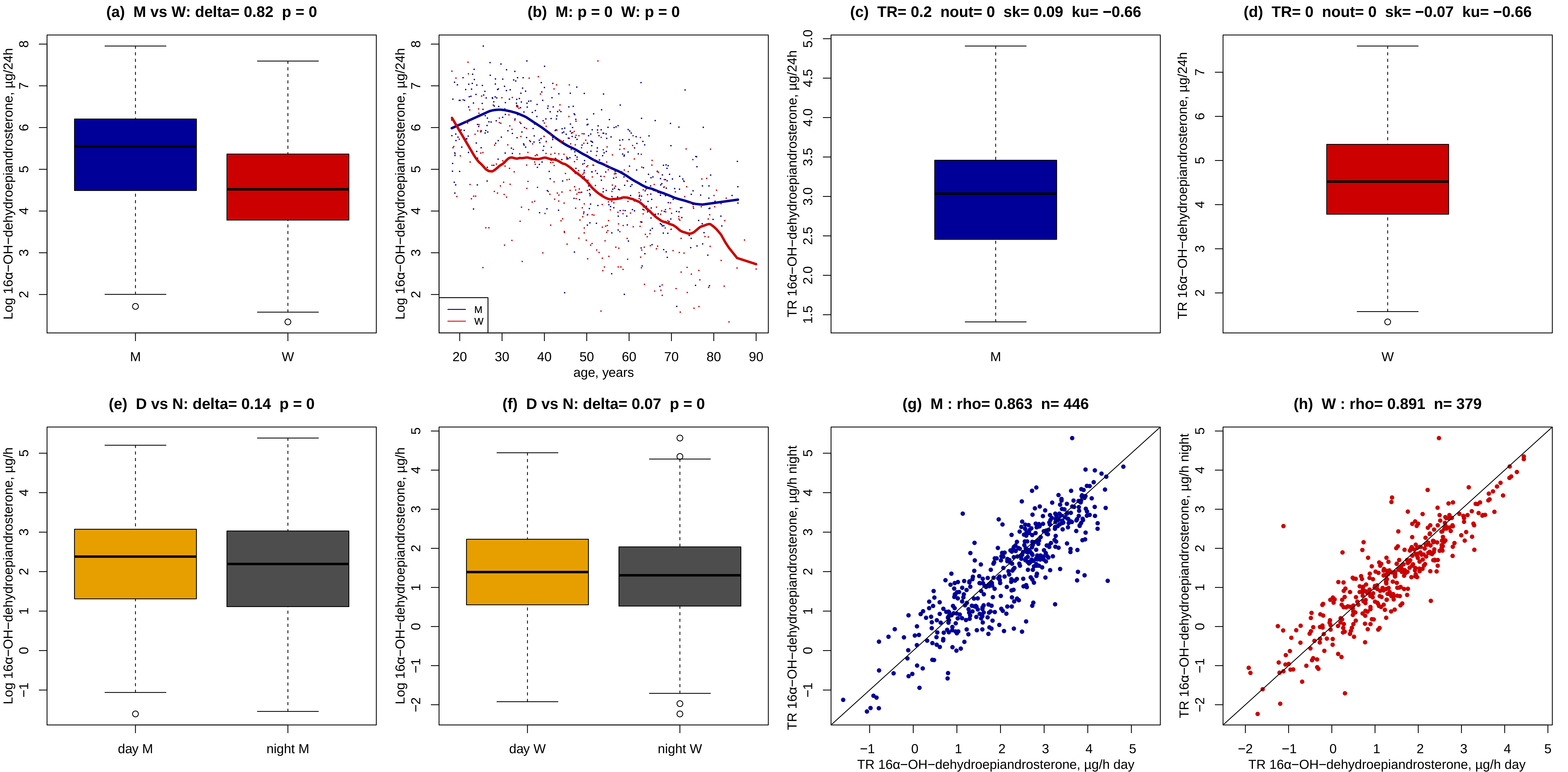

(a) M vs W: delta= 0.82 p = 0
Log 16α−OH−dehydroepiandrosterone, µg/24h
M
W
(b) M: p = 0 W: p = 0
Log 16α−OH−dehydroepiandrosterone, µg/24h
age, years
M
W
(c) TR= 0.2 nout= 0 sk= 0.09 ku= −0.66
TR 16α−OH−dehydroepiandrosterone, µg/24h
M
(d) TR= 0 nout= 0 sk= −0.07 ku= −0.66
TR 16α−OH−dehydroepiandrosterone, µg/24h
W
(e) D vs N: delta= 0.14 p = 0
Log 16α−OH−dehydroepiandrosterone, µg/h
day M
night M
(f) D vs N: delta= 0.07 p = 0
Log 16α−OH−dehydroepiandrosterone, µg/h
day W
night W
(g) M : rho= 0.863 n= 446
TR 16α−OH−dehydroepiandrosterone, µg/h night
TR 16α−OH−dehydroepiandrosterone, µg/h day
(h) W : rho= 0.891 n= 379
TR 16α−OH−dehydroepiandrosterone, µg/h night
TR 16α−OH−dehydroepiandrosterone, µg/h day

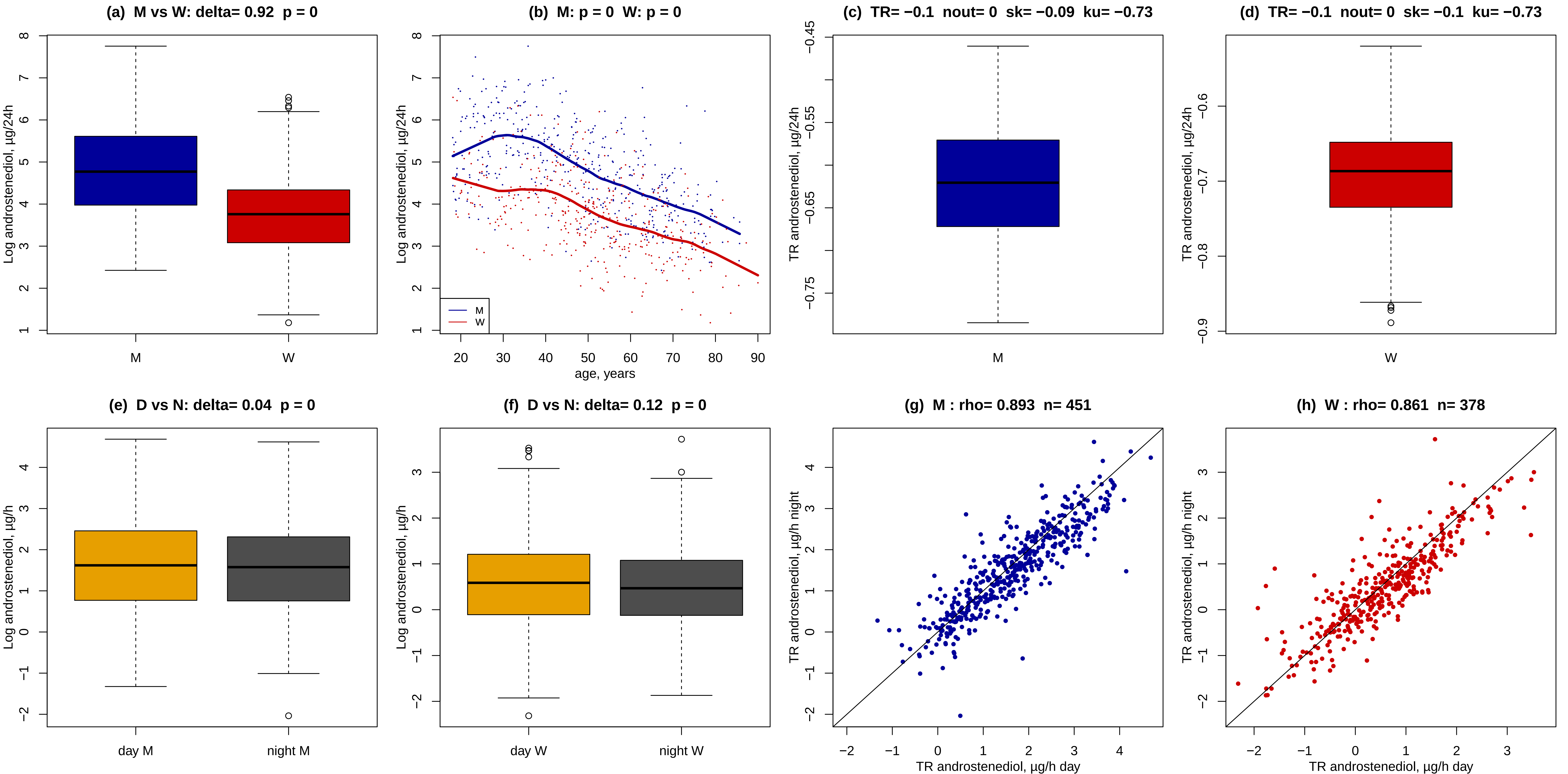

(a) M vs W: delta= 0.92 p = 0
Log androstenediol, µg/24h
M
W
(b) M: p = 0 W: p = 0
Log androstenediol, µg/24h
age, years
M
W
(c) TR= −0.1 nout= 0 sk= −0.09 ku= −0.73
TR androstenediol, µg/24h
M
(d) TR= −0.1 nout= 0 sk= −0.1 ku= −0.73
TR androstenediol, µg/24h
W
(e) D vs N: delta= 0.04 p = 0
Log androstenediol, µg/h
day M
night M
(f) D vs N: delta= 0.12 p = 0
Log androstenediol, µg/h
day W
night W
(g) M : rho= 0.893 n= 451
TR androstenediol, µg/h night
TR androstenediol, µg/h day
(h) W : rho= 0.861 n= 378
TR androstenediol, µg/h night
TR androstenediol, µg/h day

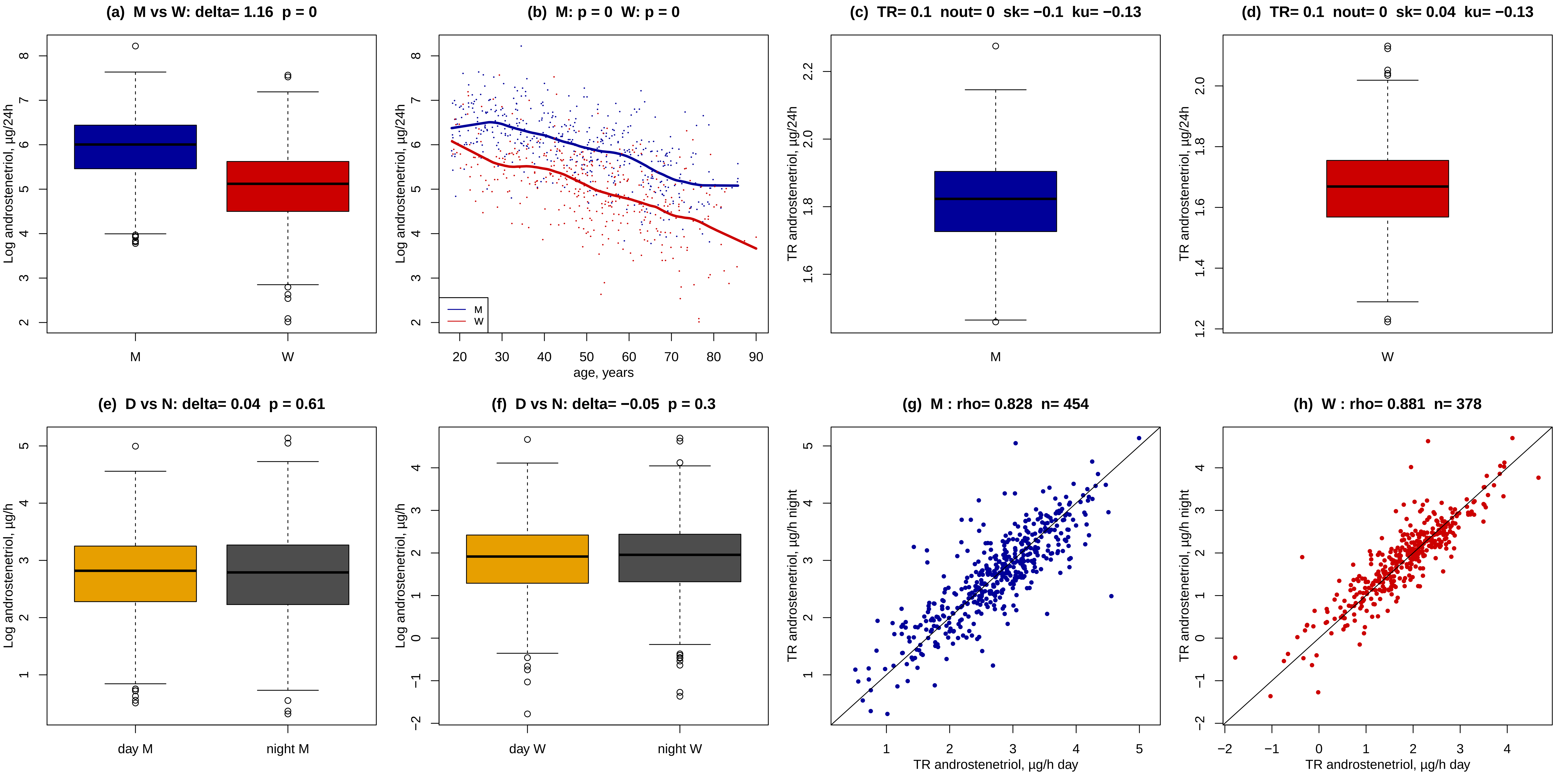

(a) M vs W: delta= 1.16 p = 0
(b) M: p = 0 W: p = 0
(c) TR= 0.1 nout= 0 sk= −0.1 ku= −0.13
(d) TR= 0.1 nout= 0 sk= 0.04 ku= −0.13
Log androstenetriol, µg/24h
TR androstenetriol, µg/24h
age, years
M
W
(e) D vs N: delta= 0.04 p = 0.61
(f) D vs N: delta= −0.05 p = 0.3
(g) M : rho= 0.828 n= 454
(h) W : rho= 0.881 n= 378
Log androstenetriol, µg/h
day M
night M
day W
night W
TR androstenetriol, µg/h night
TR androstenetriol, µg/h day

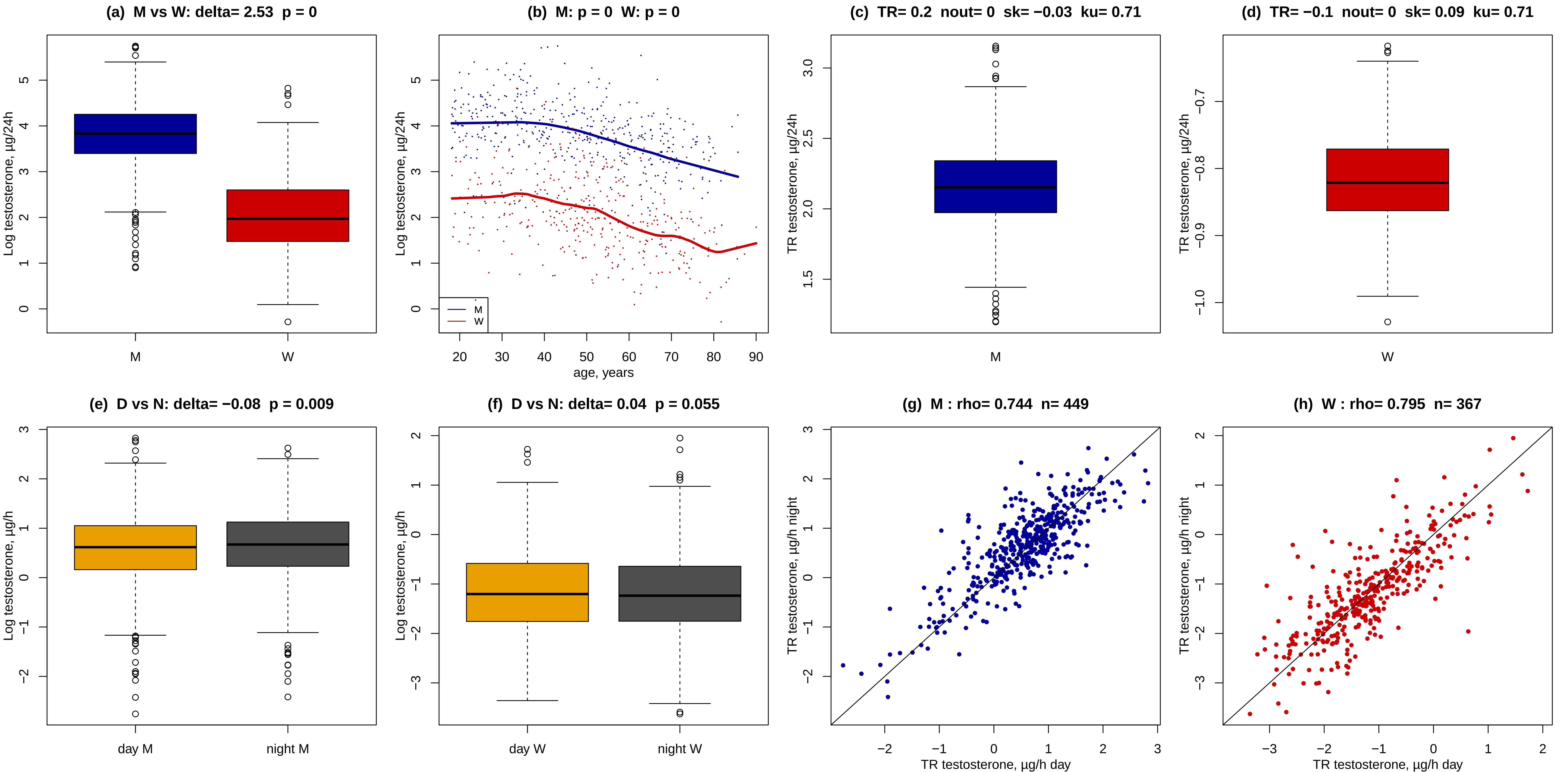

(a) M vs W: delta= 2.53 p = 0
(b) M: p = 0 W: p = 0
(c) TR= 0.2 nout= 0 sk= −0.03 ku= 0.71
(d) TR= −0.1 nout= 0 sk= 0.09 ku= 0.71
Log testosterone, µg/24h
TR testosterone, µg/24h
M
W
age, years
(e) D vs N: delta= −0.08 p = 0.009
(f) D vs N: delta= 0.04 p = 0.055
(g) M : rho= 0.744 n= 449
(h) W : rho= 0.795 n= 367
Log testosterone, µg/h
day M
night M
day W
night W
TR testosterone, µg/h night
TR testosterone, µg/h day

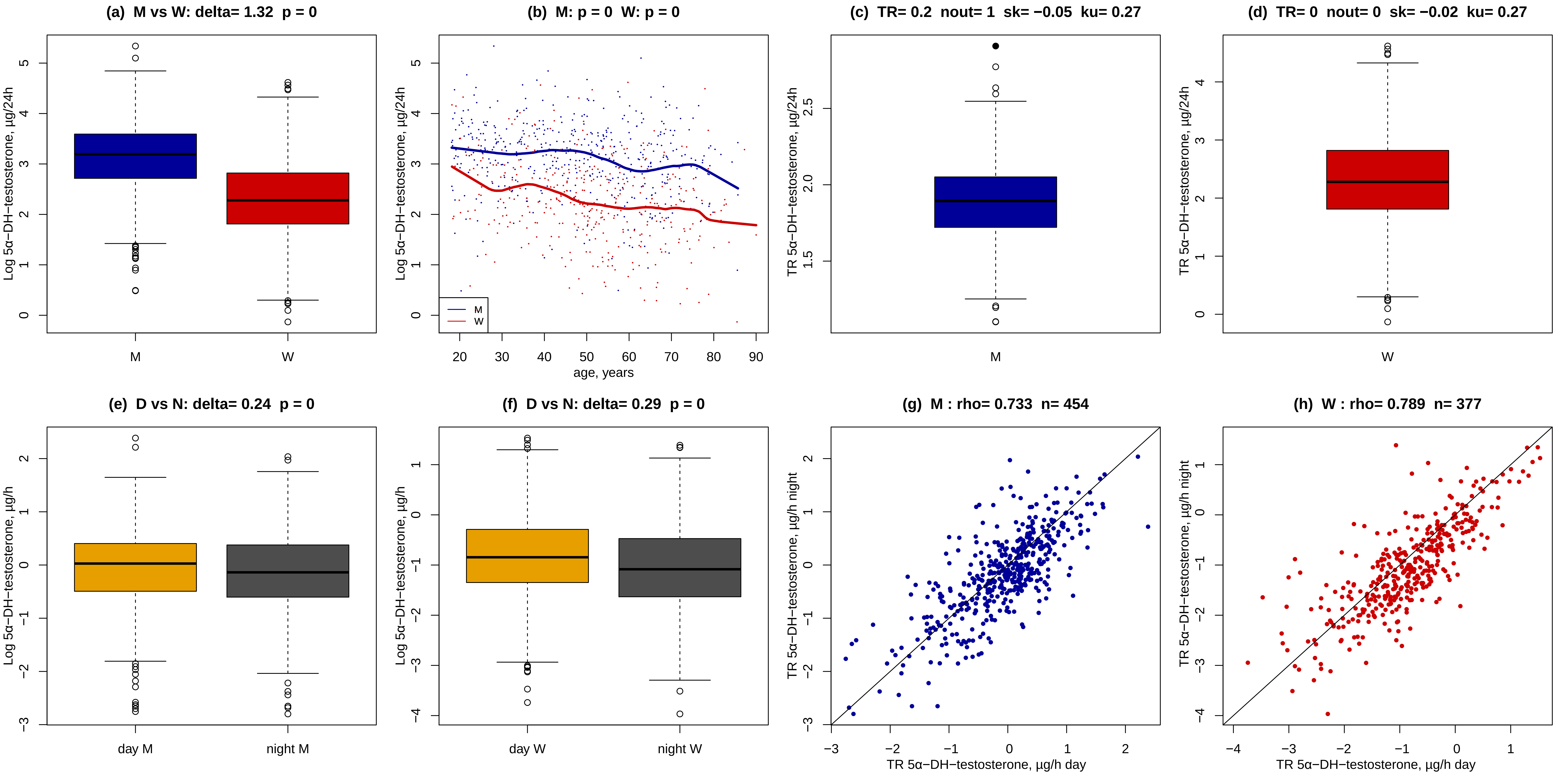

(a) M vs W: delta= 1.32 p = 0
Log 5α−DH−testosterone, μg/24h
M
W
(b) M: p = 0 W: p = 0
Log 5α−DH−testosterone, μg/24h
M
W
age, years
(c) TR= 0.2 nout= 1 sk= −0.05 ku= 0.27
TR 5α−DH−testosterone, μg/24h
M
(d) TR= 0 nout= 0 sk= −0.02 ku= 0.27
TR 5α−DH−testosterone, μg/24h
W
(e) D vs N: delta= 0.24 p = 0
Log 5α−DH−testosterone, μg/h
day M
night M
(f) D vs N: delta= 0.29 p = 0
Log 5α−DH−testosterone, μg/h
day W
night W
(g) M : rho= 0.733 n= 454
TR 5α−DH−testosterone, μg/h night
TR 5α−DH−testosterone, μg/h day
(h) W : rho= 0.789 n= 377
TR 5α−DH−testosterone, μg/h night
TR 5α−DH−testosterone, μg/h day

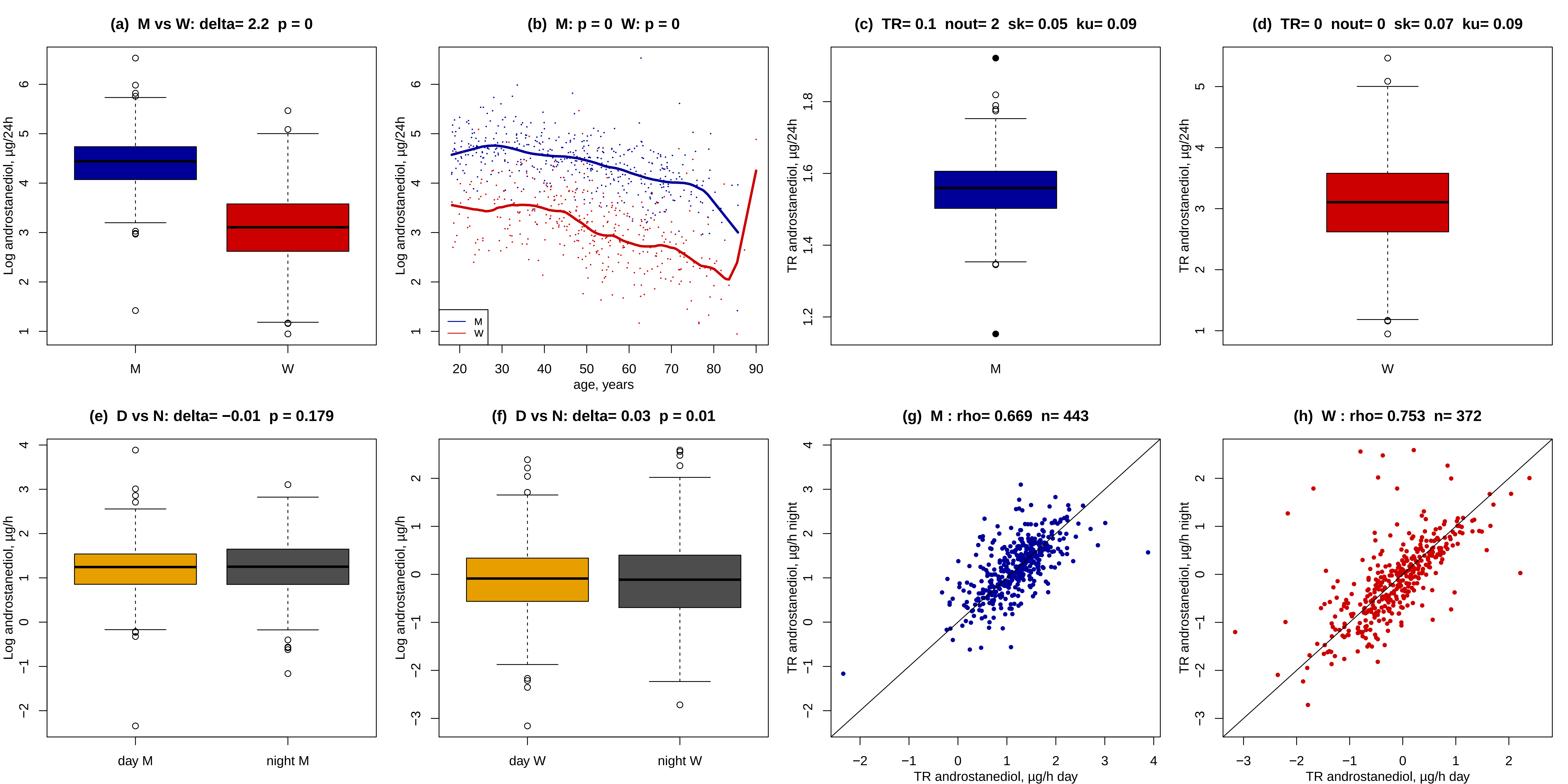

(a) M vs W: delta= 2.2 p = 0
(b) M: p = 0 W: p = 0
(c) TR= 0.1 nout= 2 sk= 0.05 ku= 0.09
(d) TR= 0 nout= 0 sk= 0.07 ku= 0.09
Log androstanediol, µg/24h
TR androstanediol, µg/24h
M
W
age, years
(e) D vs N: delta= −0.01 p = 0.179
(f) D vs N: delta= 0.03 p = 0.01
(g) M : rho= 0.669 n= 443
(h) W : rho= 0.753 n= 372
Log androstanediol, µg/h
TR androstanediol, µg/h night
TR androstanediol, µg/h day
day M
night M
day W
night W

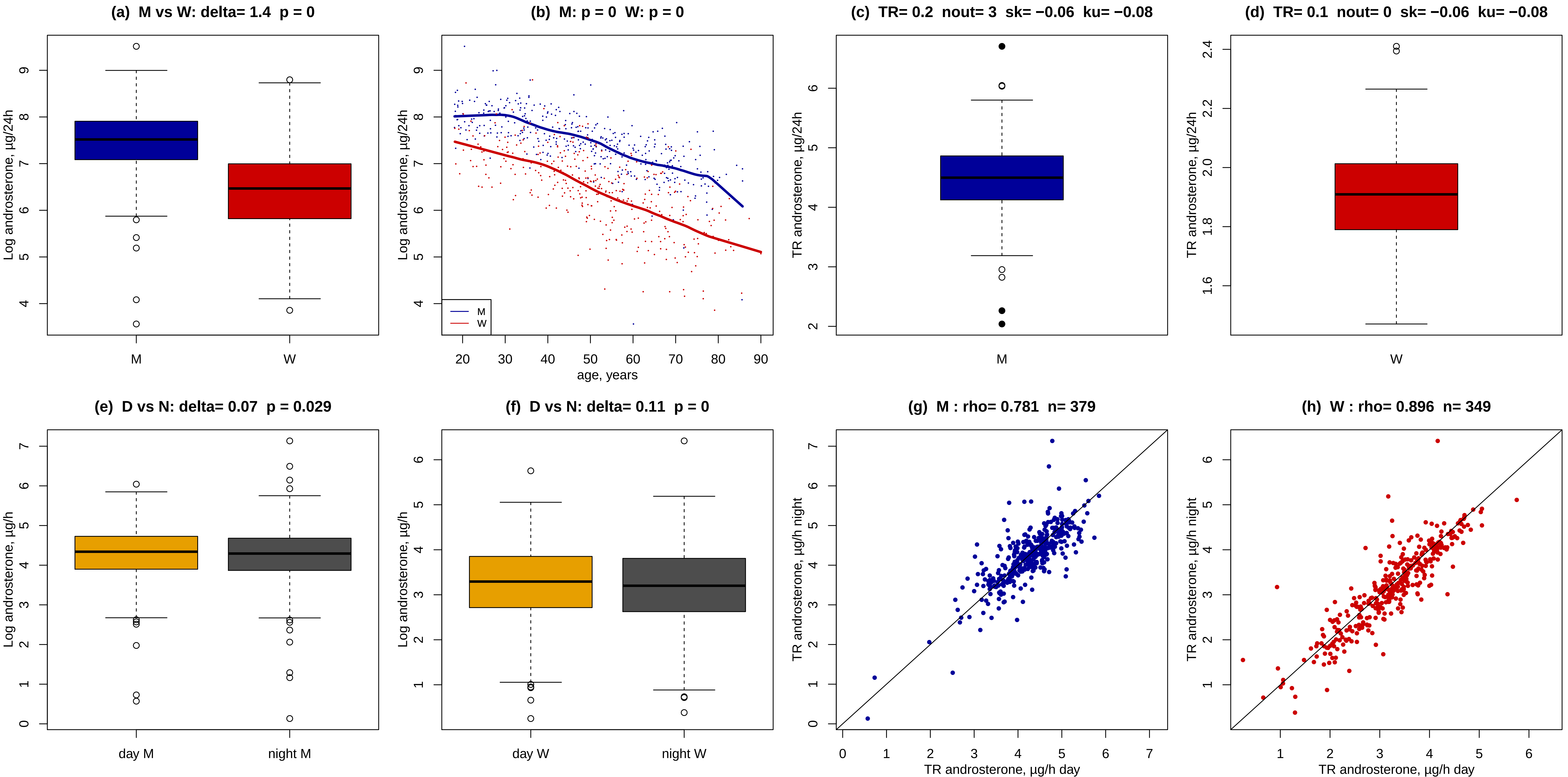

(a) M vs W: delta= 1.4 p = 0
Log androsterone, µg/24h
M
W
(b) M: p = 0 W: p = 0
Log androsterone, µg/24h
age, years
M
W
(c) TR= 0.2 nout= 3 sk= −0.06 ku= −0.08
TR androsterone, µg/24h
M
(d) TR= 0.1 nout= 0 sk= −0.06 ku= −0.08
TR androsterone, µg/24h
W
(e) D vs N: delta= 0.07 p = 0.029
Log androsterone, µg/h
day M
night M
(f) D vs N: delta= 0.11 p = 0
Log androsterone, µg/h
day W
night W
(g) M : rho= 0.781 n= 379
TR androsterone, µg/h night
TR androsterone, µg/h day
(h) W : rho= 0.896 n= 349
TR androsterone, µg/h night
TR androsterone, µg/h day

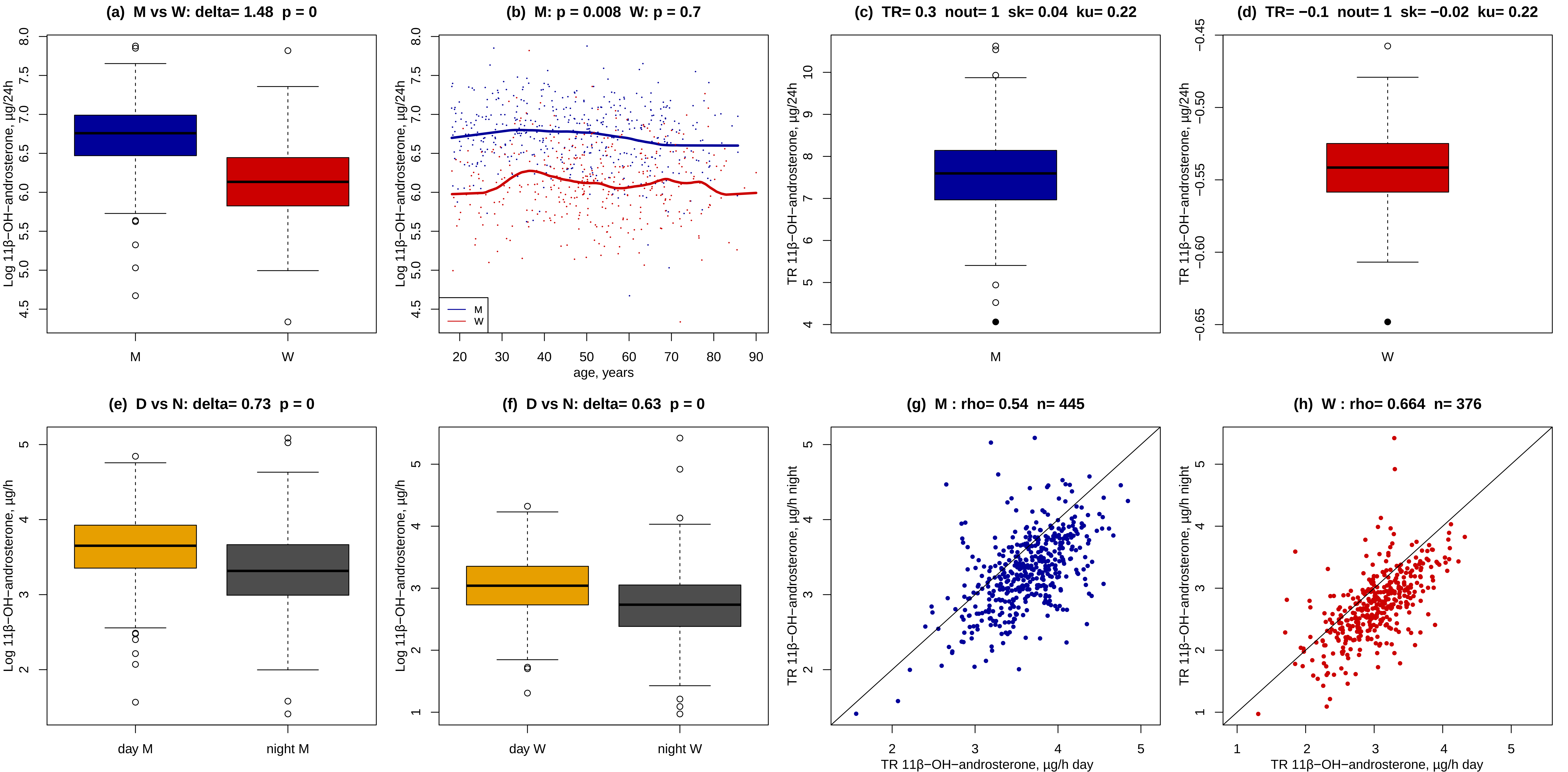

(a) M vs W: delta= 1.48 p = 0
(b) M: p = 0.008 W: p = 0.7
(c) TR= 0.3 nout= 1 sk= 0.04 ku= 0.22
(d) TR= −0.1 nout= 1 sk= −0.02 ku= 0.22
Log 11β−OH−androsterone, μg/24h
TR 11β−OH−androsterone, μg/24h
age, years
M
W
(e) D vs N: delta= 0.73 p = 0
(f) D vs N: delta= 0.63 p = 0
(g) M : rho= 0.54 n= 445
(h) W : rho= 0.664 n= 376
Log 11β−OH−androsterone, μg/h
TR 11β−OH−androsterone, μg/h night
TR 11β−OH−androsterone, μg/h day
day M
night M
day W
night W

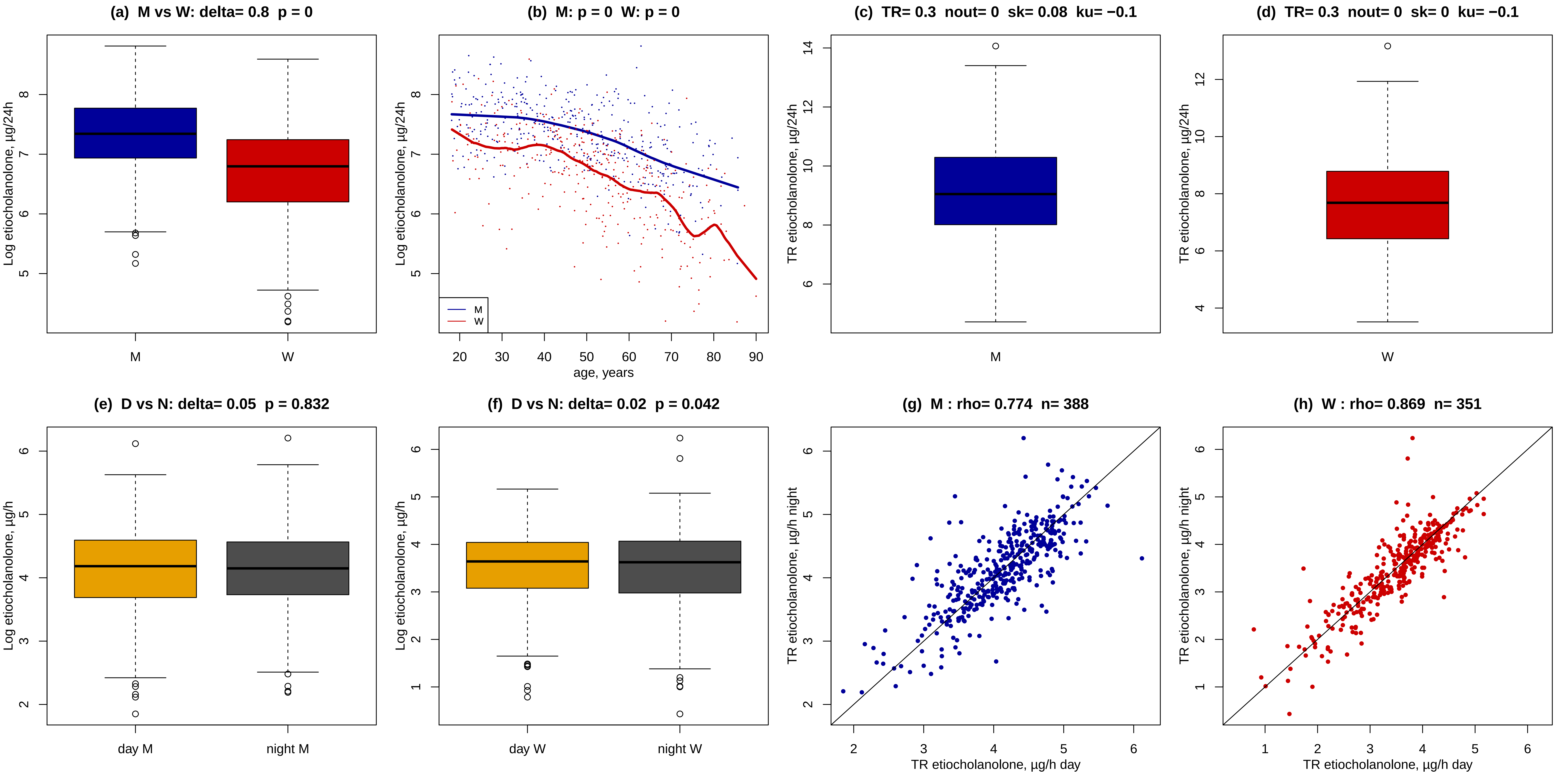

(a) M vs W: delta= 0.8 p = 0
(b) M: p = 0 W: p = 0
(c) TR= 0.3 nout= 0 sk= 0.08 ku= −0.1
(d) TR= 0.3 nout= 0 sk= 0 ku= −0.1
Log etiocholanolone, µg/24h
TR etiocholanolone, µg/24h
M
W
age, years
(e) D vs N: delta= 0.05 p = 0.832
(f) D vs N: delta= 0.02 p = 0.042
(g) M : rho= 0.774 n= 388
(h) W : rho= 0.869 n= 351
Log etiocholanolone, µg/h
day M
night M
day W
night W
TR etiocholanolone, µg/h night
TR etiocholanolone, µg/h day

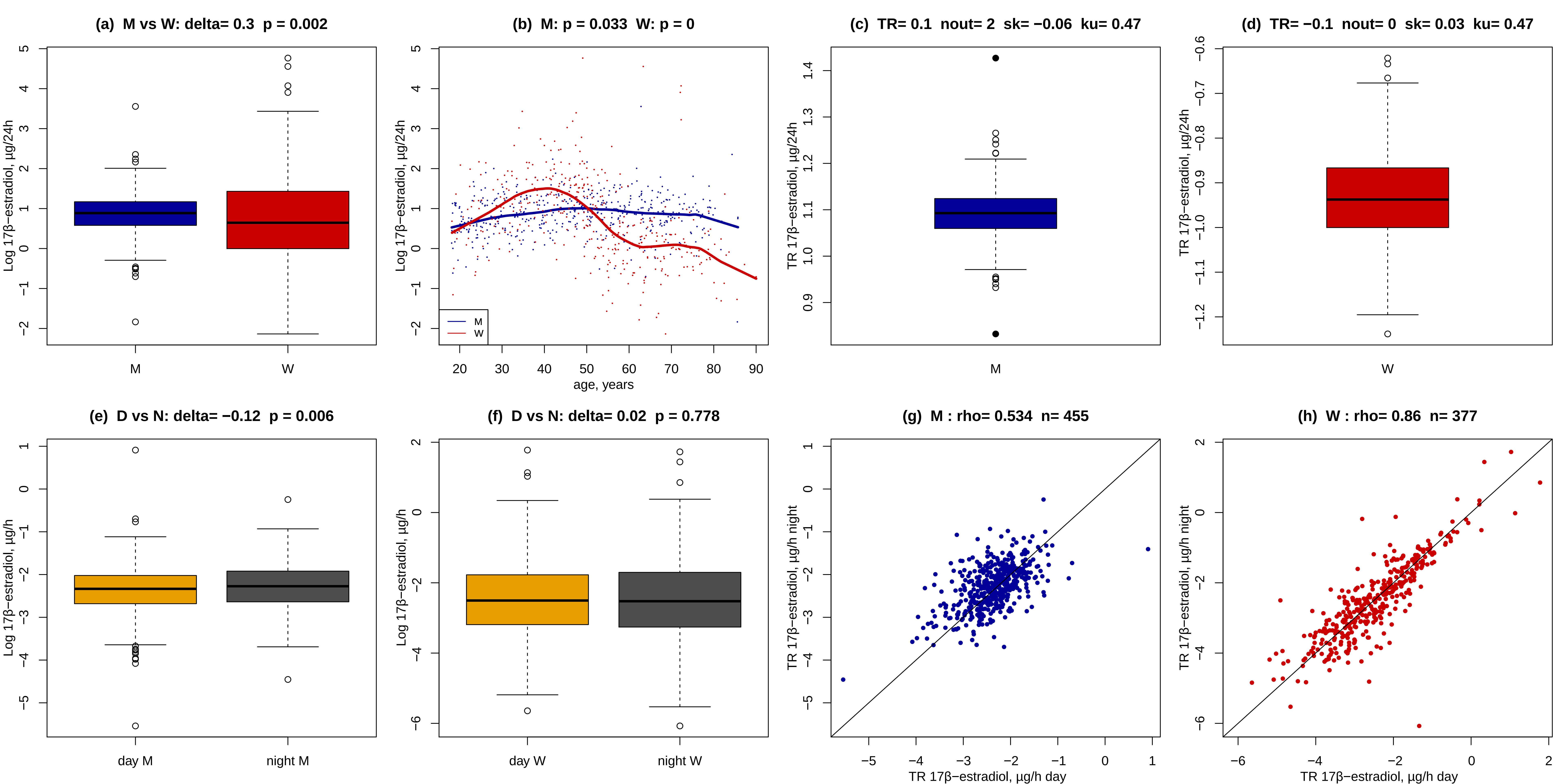
(a) M vs W: delta= 0.3 p = 0.002
Log 17β−estradiol, µg/24h
M
W
(b) M: p = 0.033 W: p = 0
Log 17β−estradiol, µg/24h
age, years
M
W
(c) TR= 0.1 nout= 2 sk= −0.06 ku= 0.47
TR 17β−estradiol, µg/24h
M
(d) TR= −0.1 nout= 0 sk= 0.03 ku= 0.47
TR 17β−estradiol, µg/24h
W
(e) D vs N: delta= −0.12 p = 0.006
Log 17β−estradiol, µg/h
day M
night M
(f) D vs N: delta= 0.02 p = 0.778
Log 17β−estradiol, µg/h
day W
night W
(g) M : rho= 0.534 n= 455
TR 17β−estradiol, µg/h night
TR 17β−estradiol, µg/h day
(h) W : rho= 0.86 n= 377
TR 17β−estradiol, µg/h night
TR 17β−estradiol, µg/h day

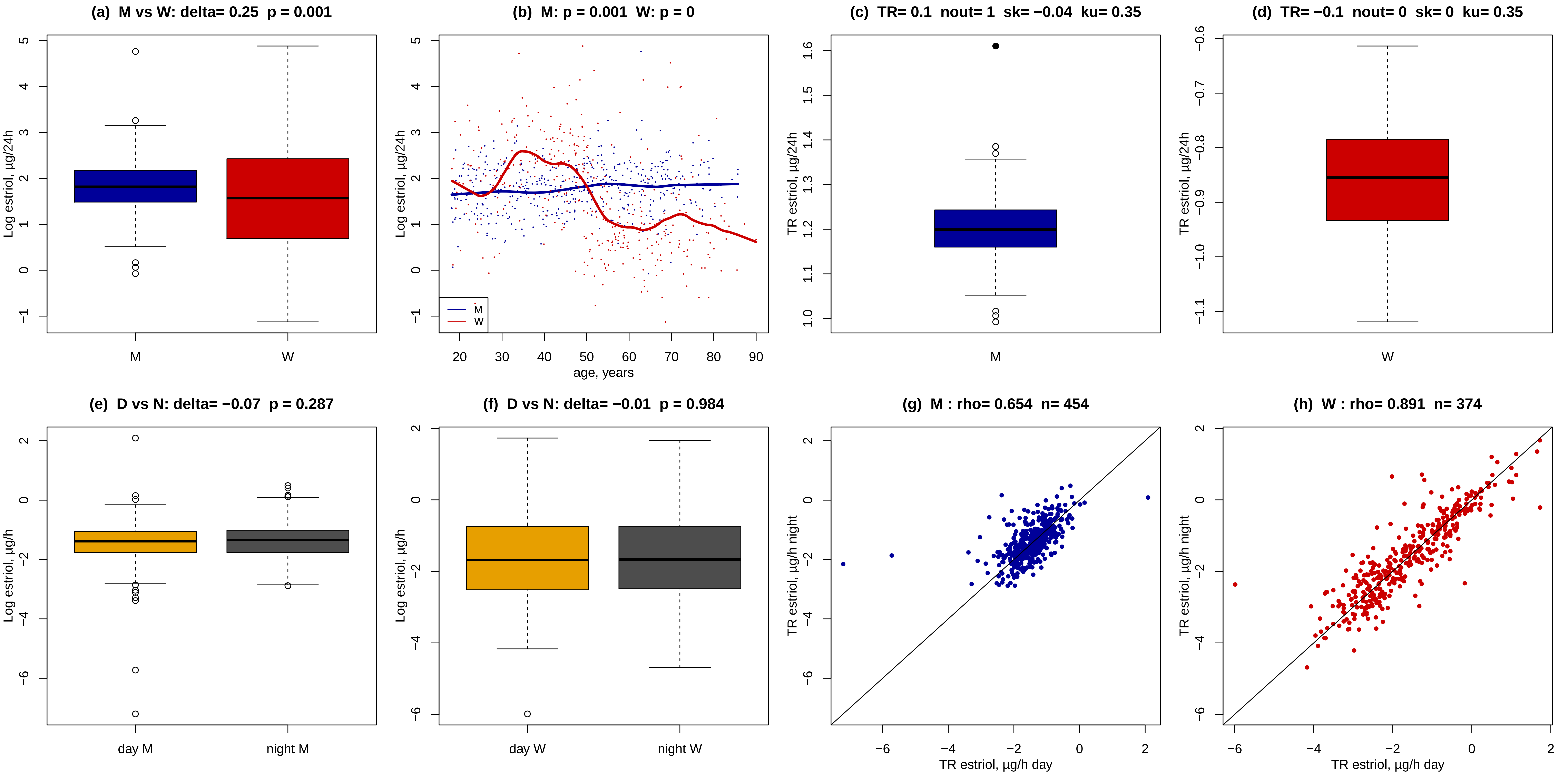

(a) M vs W: delta= 0.25 p = 0.001
Log estriol, µg/24h
M
W
(b) M: p = 0.001 W: p = 0
Log estriol, µg/24h
M
W
age, years
(c) TR= 0.1 nout= 1 sk= −0.04 ku= 0.35
TR estriol, µg/24h
M
(d) TR= −0.1 nout= 0 sk= 0 ku= 0.35
TR estriol, µg/24h
W
(e) D vs N: delta= −0.07 p = 0.287
Log estriol, µg/h
day M
night M
(f) D vs N: delta= −0.01 p = 0.984
Log estriol, µg/h
day W
night W
(g) M : rho= 0.654 n= 454
TR estriol, µg/h night
TR estriol, µg/h day
(h) W : rho= 0.891 n= 374
TR estriol, µg/h night
TR estriol, µg/h day

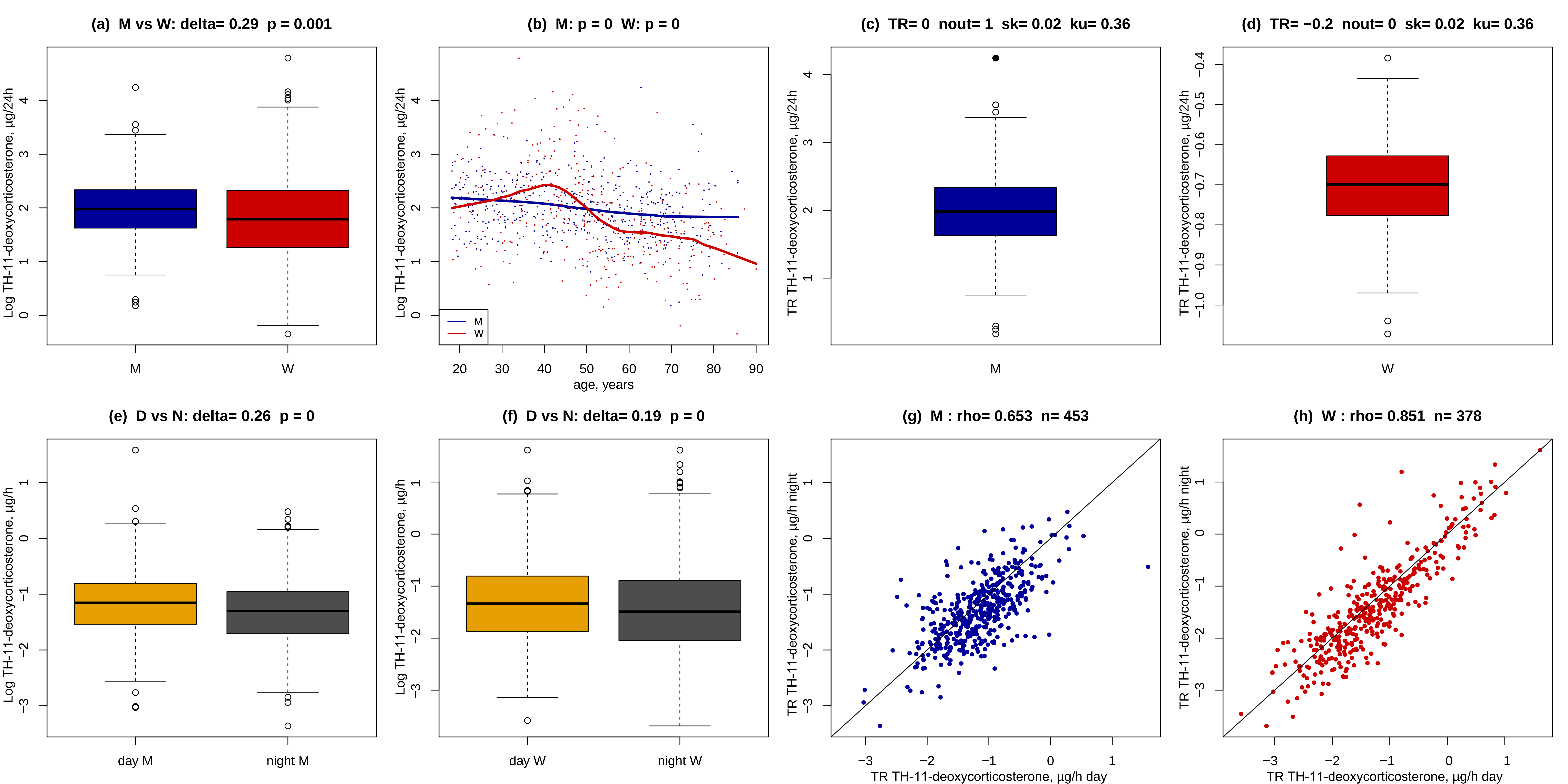

(a) M vs W: delta= 0.29 p = 0.001
(b) M: p = 0 W: p = 0
(c) TR= 0 nout= 1 sk= 0.02 ku= 0.36
(d) TR= −0.2 nout= 0 sk= 0.02 ku= 0.36
Log TH-11-deoxycorticosterone, µg/24h
TR TH-11-deoxycorticosterone, µg/24h
age, years
M
W
(e) D vs N: delta= 0.26 p = 0
(f) D vs N: delta= 0.19 p = 0
(g) M : rho= 0.653 n= 453
(h) W : rho= 0.851 n= 378
Log TH-11-deoxycorticosterone, µg/h
TR TH-11-deoxycorticosterone, µg/h night
TR TH-11-deoxycorticosterone, µg/h day
day M
night M
day W
night W

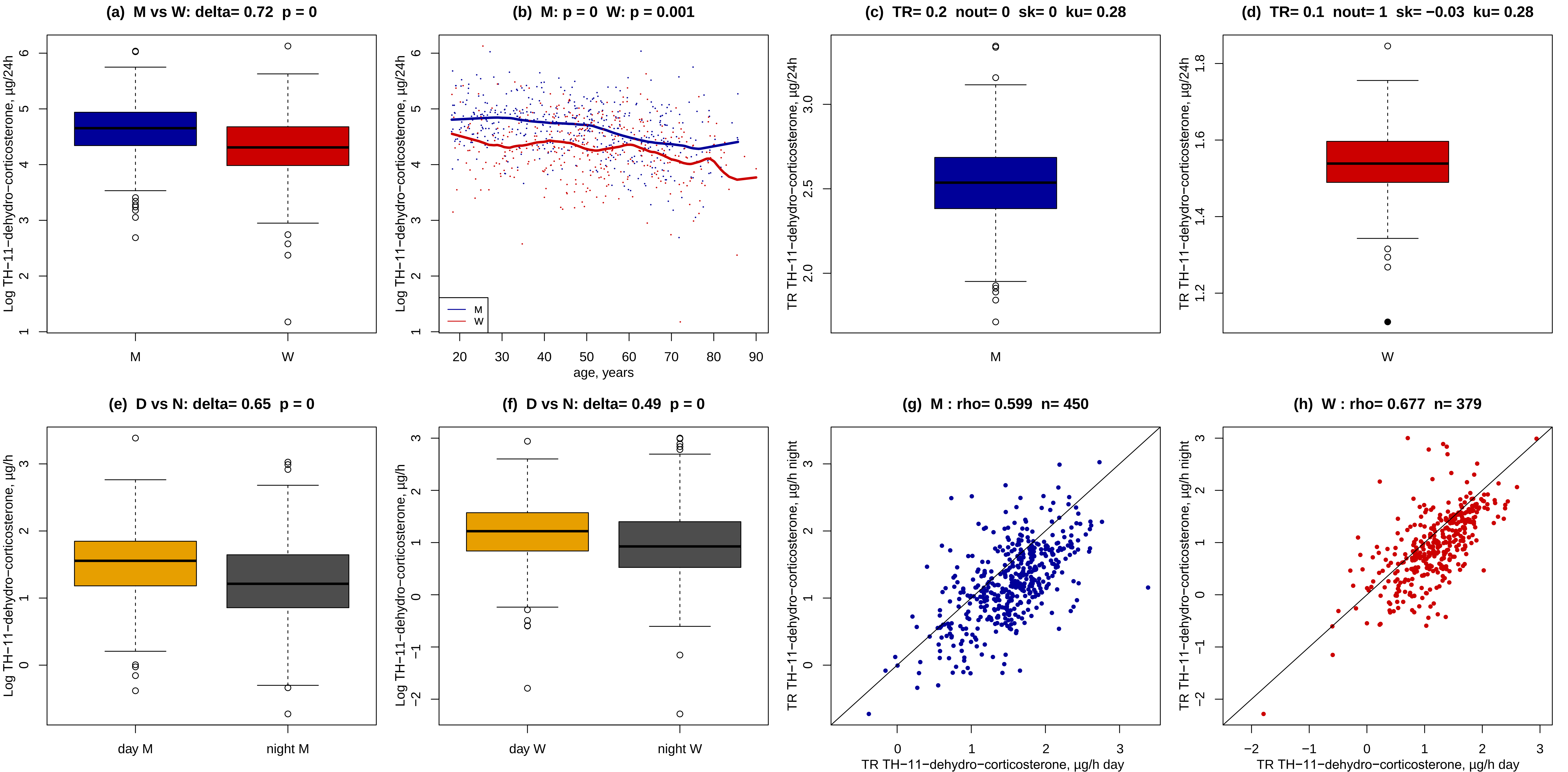

(a) M vs W: delta= 0.72 p = 0
(b) M: p = 0 W: p = 0.001
(c) TR= 0.2 nout= 0 sk= 0 ku= 0.28
(d) TR= 0.1 nout= 1 sk= −0.03 ku= 0.28
Log TH−11−dehydro−corticosterone, µg/24h
TR TH−11−dehydro−corticosterone, µg/24h
age, years
M
W
(e) D vs N: delta= 0.65 p = 0
(f) D vs N: delta= 0.49 p = 0
(g) M : rho= 0.599 n= 450
(h) W : rho= 0.677 n= 379
Log TH−11−dehydro−corticosterone, µg/h
TR TH−11−dehydro−corticosterone, µg/h night
TR TH−11−dehydro−corticosterone, µg/h day
day M
night M
day W
night W

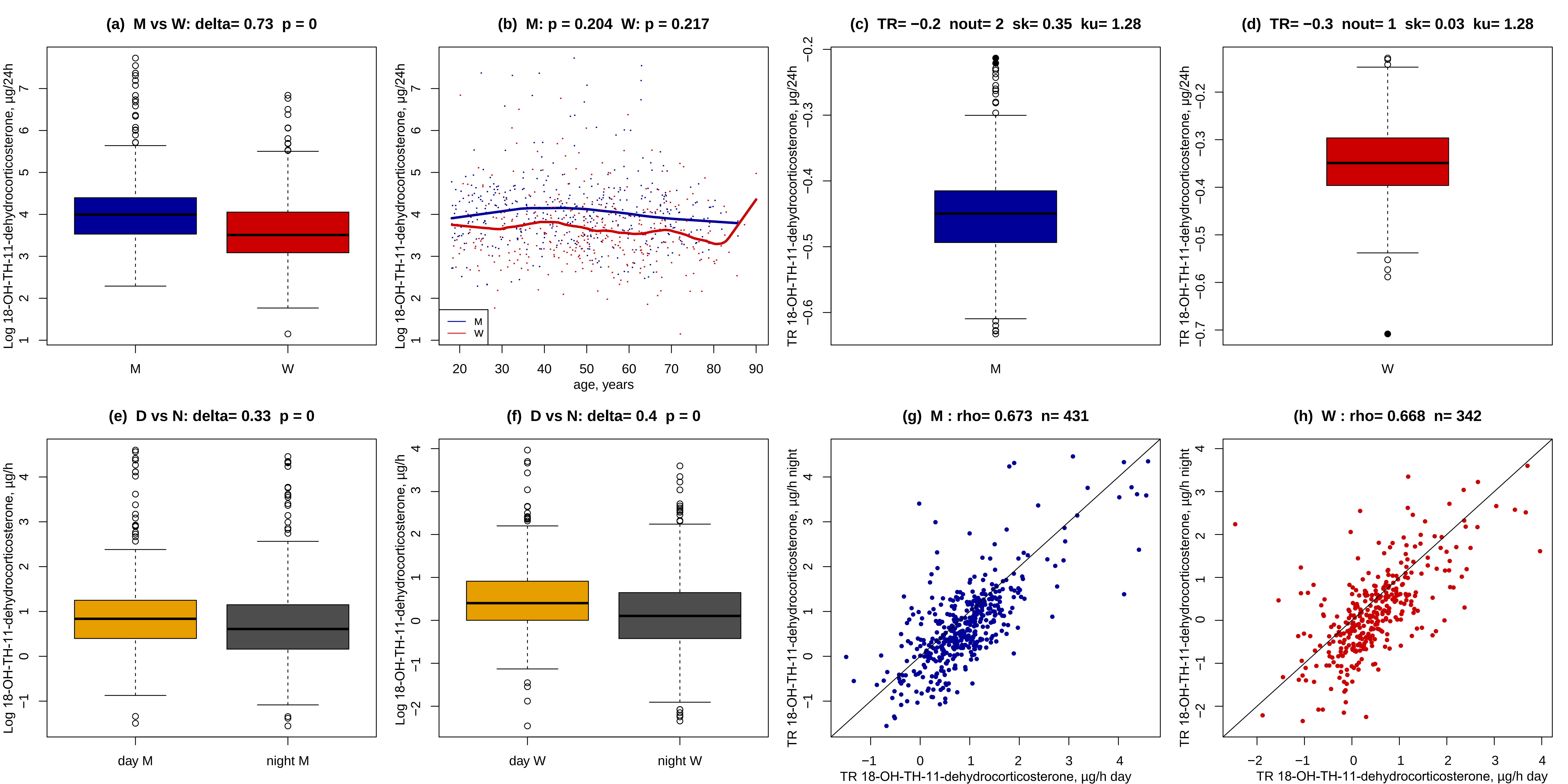
(a) M vs W: delta= 0.73 p = 0
Log 18-OH-TH-11-dehydrocorticosterone, µg/24h
M
W
(b) M: p = 0.204 W: p = 0.217
Log 18-OH-TH-11-dehydrocorticosterone, µg/24h
M
W
age, years
(c) TR= −0.2 nout= 2 sk= 0.35 ku= 1.28
TR 18-OH-TH-11-dehydrocorticosterone, µg/24h
M
(d) TR= −0.3 nout= 1 sk= 0.03 ku= 1.28
TR 18-OH-TH-11-dehydrocorticosterone, µg/24h
W
(e) D vs N: delta= 0.33 p = 0
Log 18-OH-TH-11-dehydrocorticosterone, µg/h
day M
night M
(f) D vs N: delta= 0.4 p = 0
Log 18-OH-TH-11-dehydrocorticosterone, µg/h
day W
night W
(g) M : rho= 0.673 n= 431
TR 18-OH-TH-11-dehydrocorticosterone, µg/h night
TR 18-OH-TH-11-dehydrocorticosterone, µg/h day
(h) W : rho= 0.668 n= 342
TR 18-OH-TH-11-dehydrocorticosterone, µg/h night
TR 18-OH-TH-11-dehydrocorticosterone, µg/h day

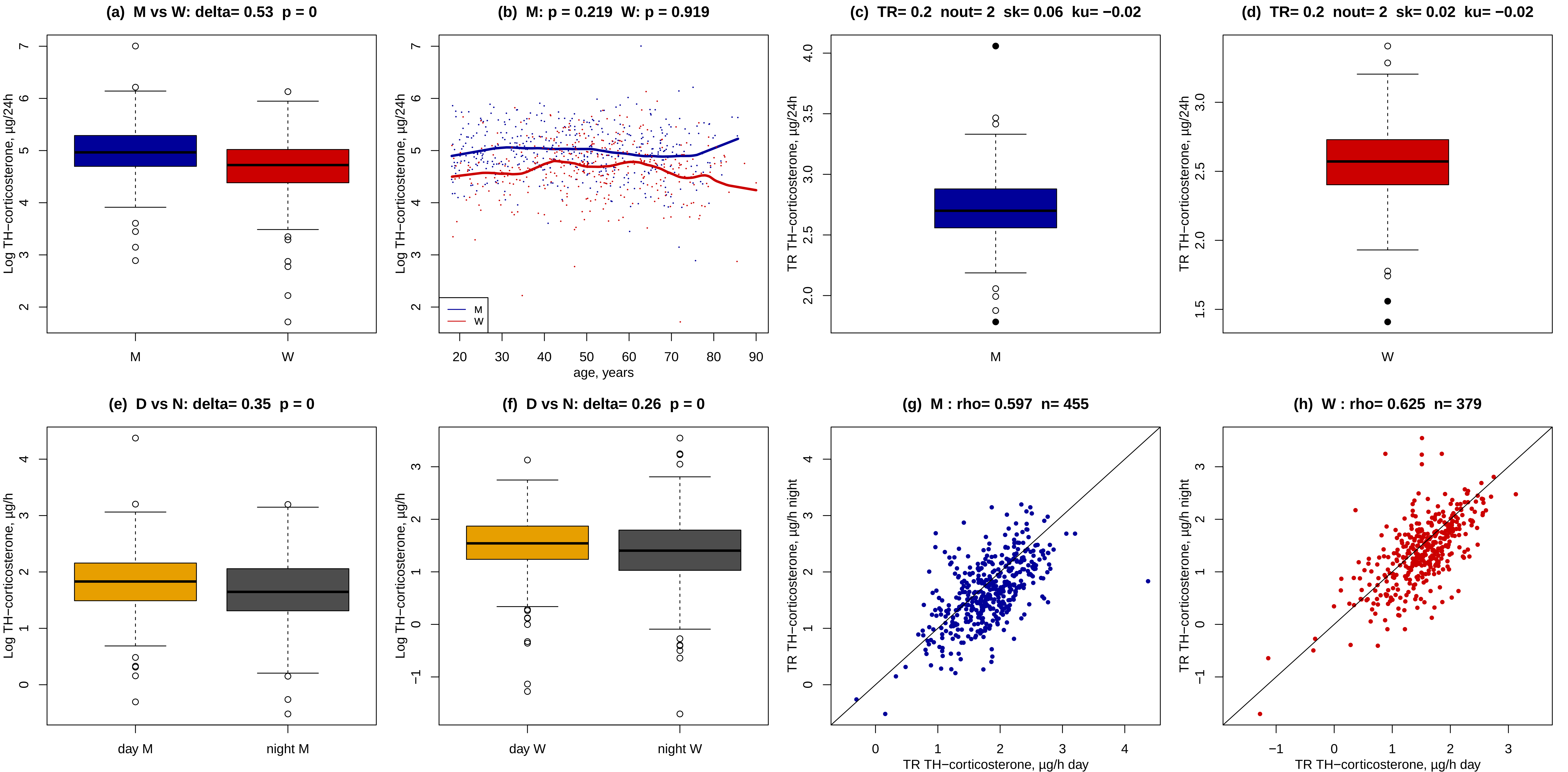

(a) M vs W: delta= 0.53 p = 0
Log TH−corticosterone, µg/24h
M
W
(b) M: p = 0.219 W: p = 0.919
Log TH−corticosterone, µg/24h
M
W
age, years
(c) TR= 0.2 nout= 2 sk= 0.06 ku= −0.02
TR TH−corticosterone, µg/24h
M
(d) TR= 0.2 nout= 2 sk= 0.02 ku= −0.02
TR TH−corticosterone, µg/24h
W
(e) D vs N: delta= 0.35 p = 0
Log TH−corticosterone, µg/h
day M
night M
(f) D vs N: delta= 0.26 p = 0
Log TH−corticosterone, µg/h
day W
night W
(g) M : rho= 0.597 n= 455
TR TH−corticosterone, µg/h night
TR TH−corticosterone, µg/h day
(h) W : rho= 0.625 n= 379
TR TH−corticosterone, µg/h night
TR TH−corticosterone, µg/h day

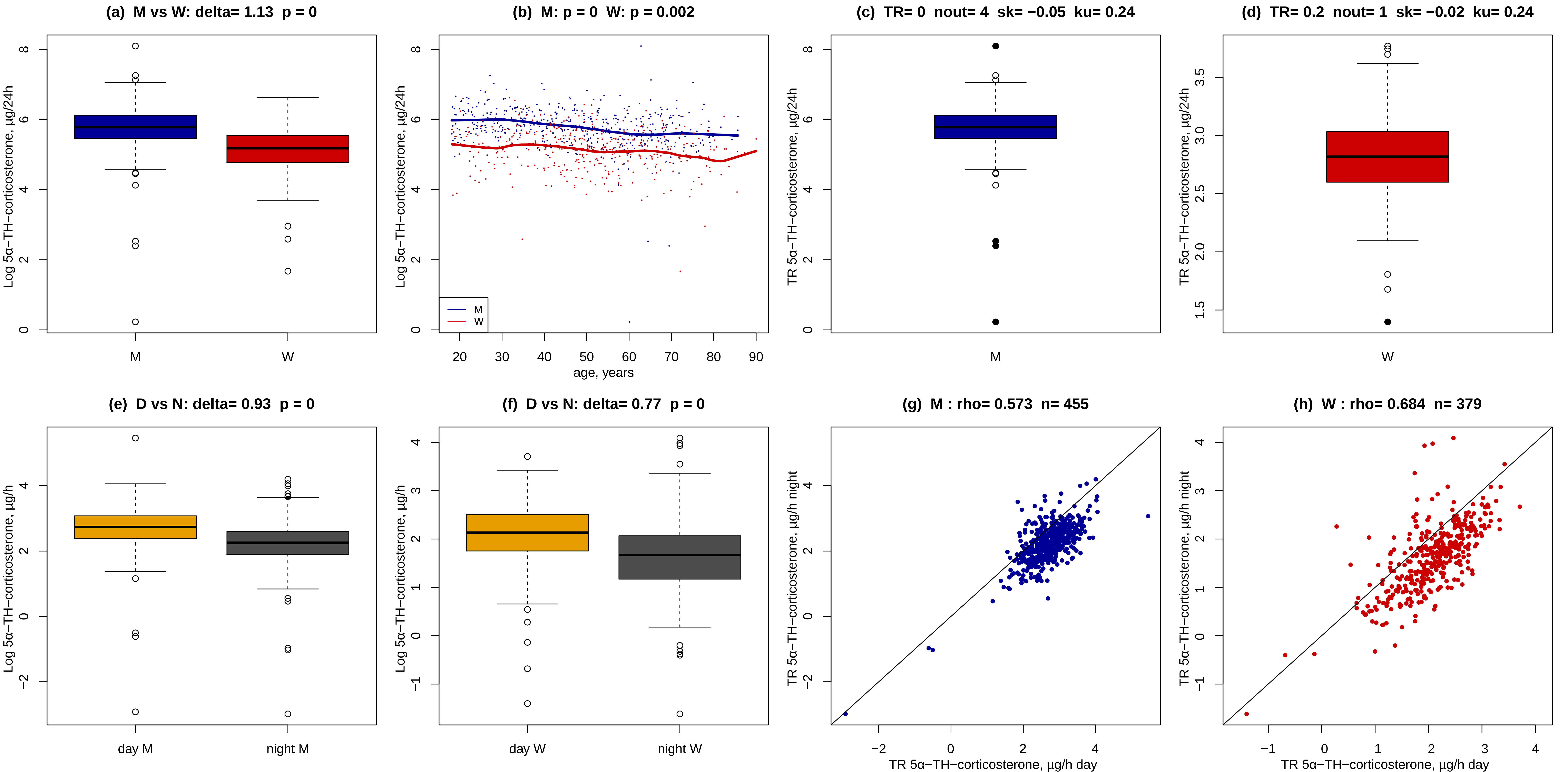
(a) M vs W: delta= 1.13 p = 0
(b) M: p = 0 W: p = 0.002
(c) TR= 0 nout= 4 sk= −0.05 ku= 0.24
(d) TR= 0.2 nout= 1 sk= −0.02 ku= 0.24
Log 5α−TH−corticosterone, µg/24h
TR 5α−TH−corticosterone, µg/24h
M
W
age, years
(e) D vs N: delta= 0.93 p = 0
(f) D vs N: delta= 0.77 p = 0
(g) M : rho= 0.573 n= 455
(h) W : rho= 0.684 n= 379
Log 5α−TH−corticosterone, µg/h
TR 5α−TH−corticosterone, µg/h night
TR 5α−TH−corticosterone, µg/h day
day M
night M
day W
night W

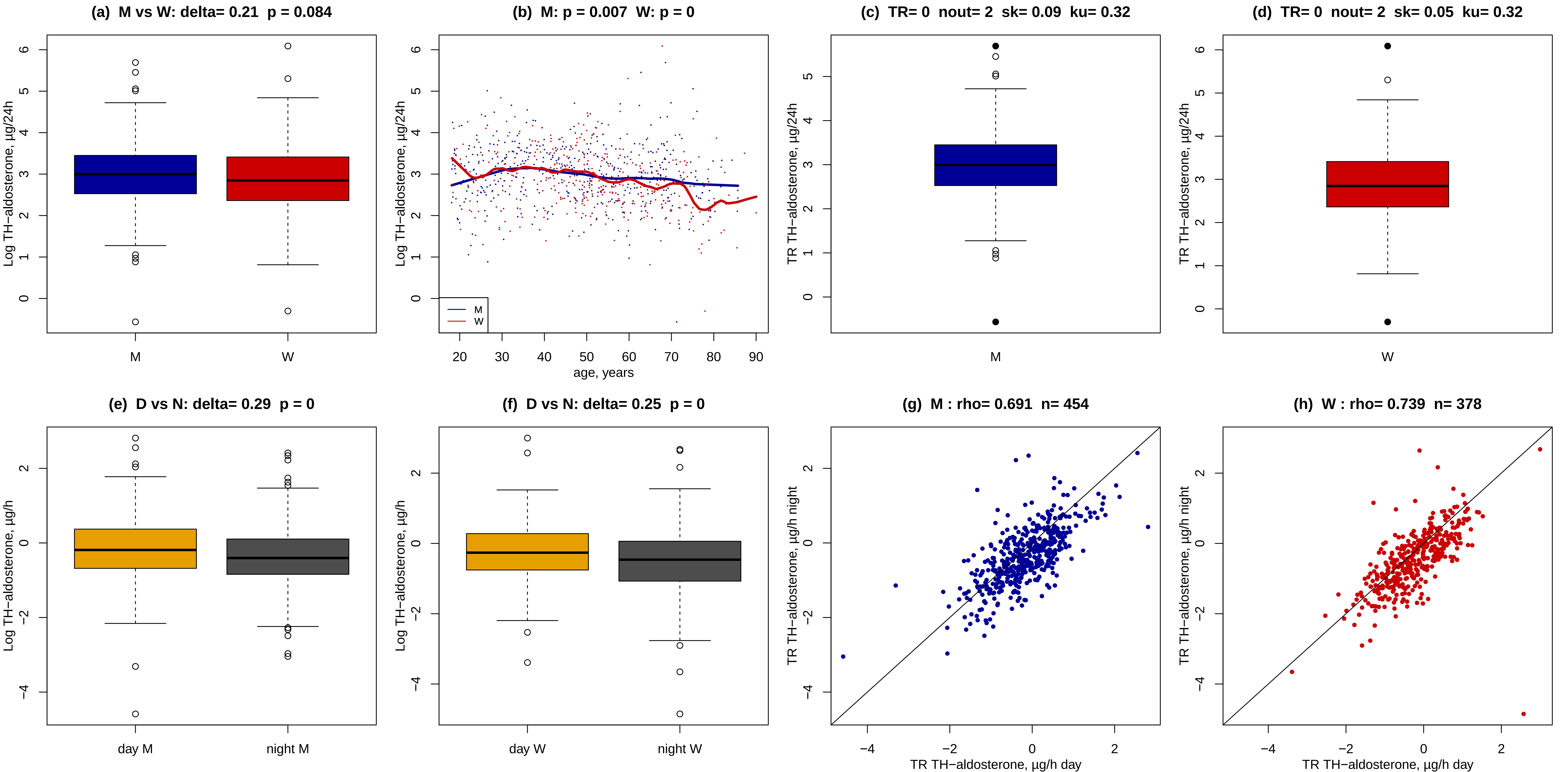

(a) M vs W: delta= 0.21 p = 0.084
(b) M: p = 0.007 W: p = 0
(c) TR= 0 nout= 2 sk= 0.09 ku= 0.32
(d) TR= 0 nout= 2 sk= 0.05 ku= 0.32
Log TH−aldosterone, µg/24h
TR TH−aldosterone, µg/24h
M
W
age, years
(e) D vs N: delta= 0.29 p = 0
(f) D vs N: delta= 0.25 p = 0
(g) M : rho= 0.691 n= 454
(h) W : rho= 0.739 n= 378
Log TH−aldosterone, µg/h
TR TH−aldosterone, µg/h night
TR TH−aldosterone, µg/h day
day M
night M
day W
night W

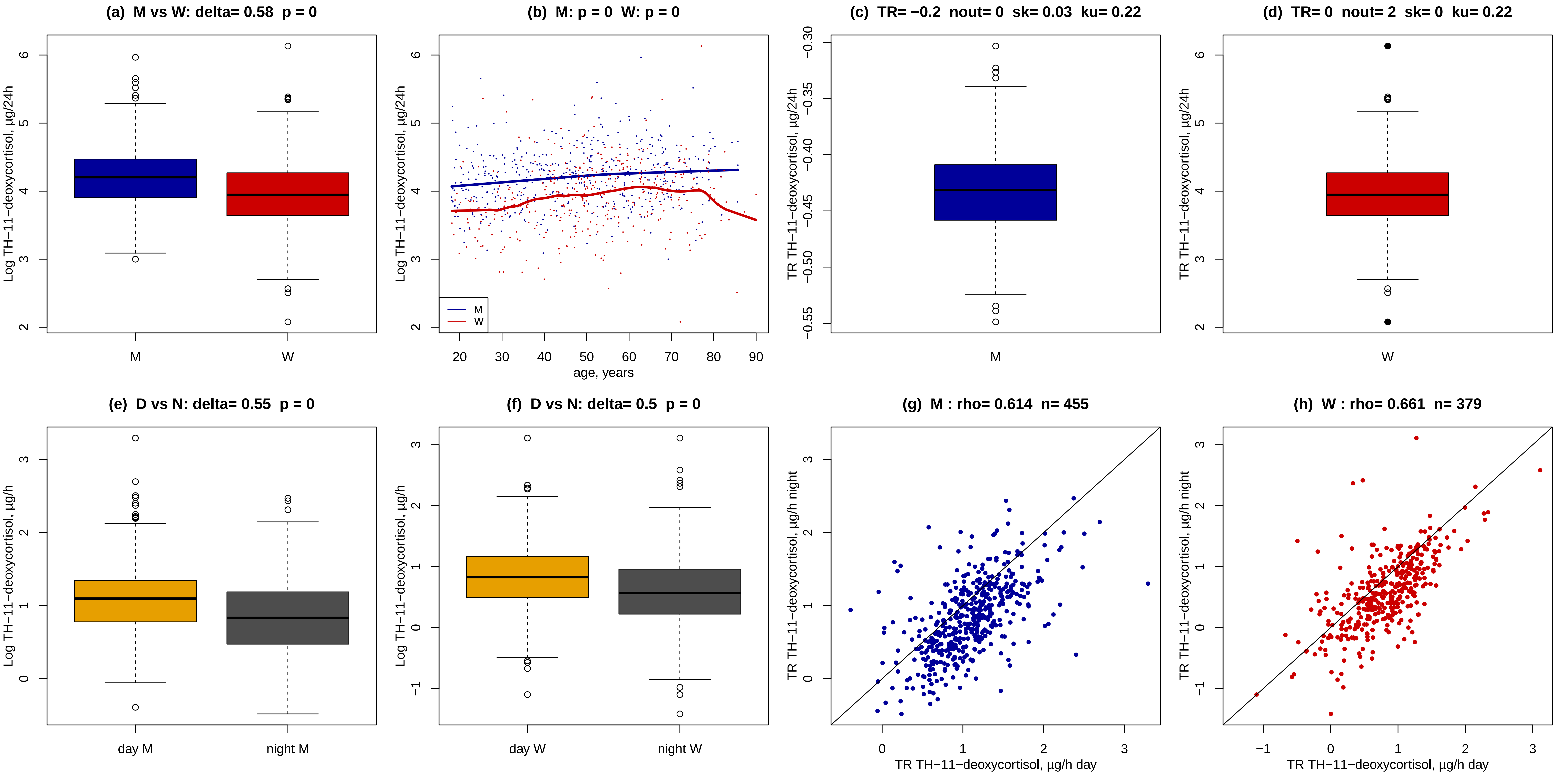
(a) M vs W: delta= 0.58 p = 0
Log TH−11−deoxycortisol, µg/24h
M
W
(b) M: p = 0 W: p = 0
Log TH−11−deoxycortisol, µg/24h
age, years
M
W
(c) TR= −0.2 nout= 0 sk= 0.03 ku= 0.22
TR TH−11−deoxycortisol, µg/24h
M
(d) TR= 0 nout= 2 sk= 0 ku= 0.22
TR TH−11−deoxycortisol, µg/24h
W
(e) D vs N: delta= 0.55 p = 0
Log TH−11−deoxycortisol, µg/h
day M
night M
(f) D vs N: delta= 0.5 p = 0
Log TH−11−deoxycortisol, µg/h
day W
night W
(g) M : rho= 0.614 n= 455
TR TH−11−deoxycortisol, µg/h night
TR TH−11−deoxycortisol, µg/h day
(h) W : rho= 0.661 n= 379
TR TH−11−deoxycortisol, µg/h night
TR TH−11−deoxycortisol, µg/h day

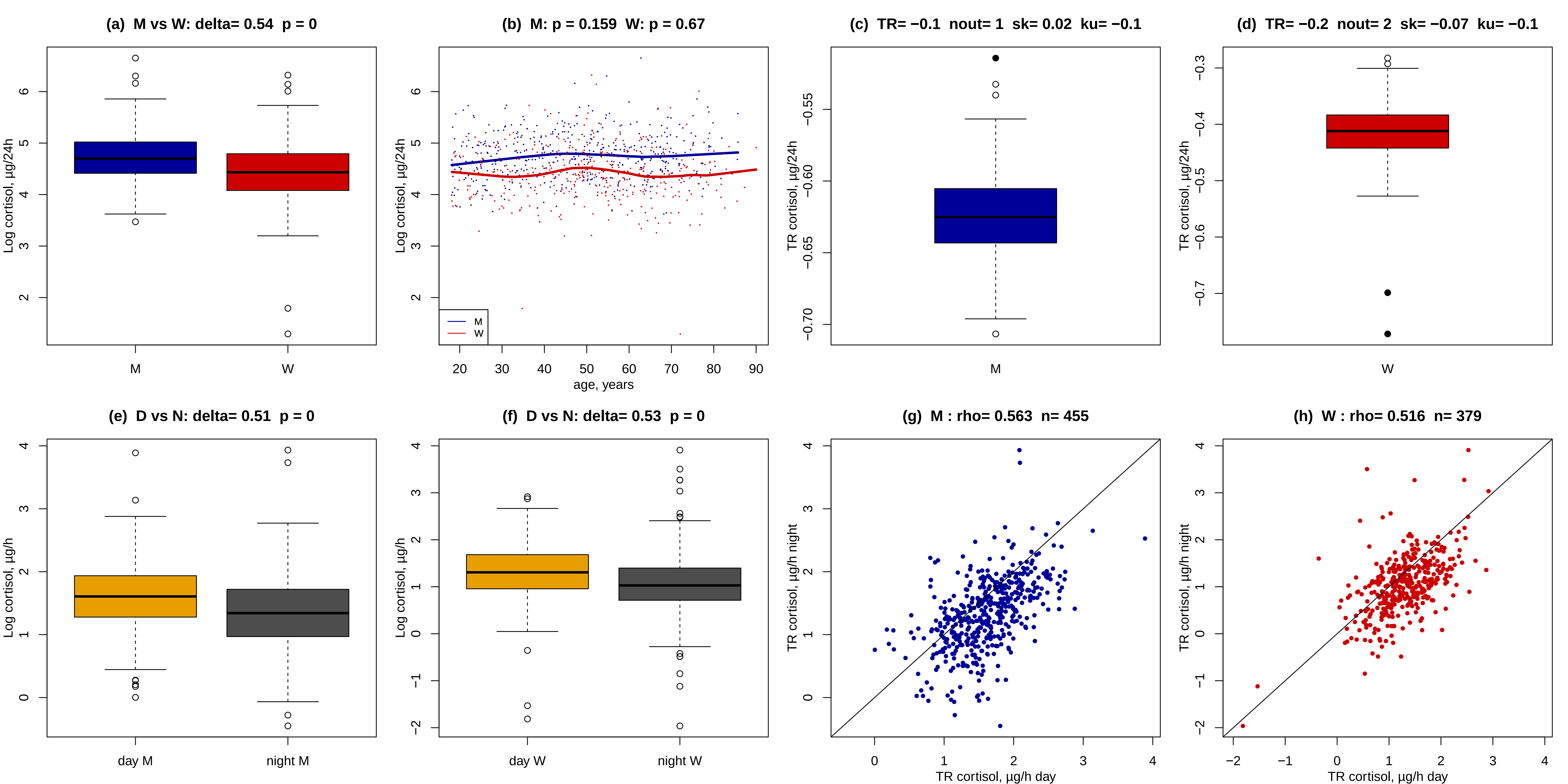
(a) M vs W: delta= 0.54 p = 0
Log cortisol, µg/24h
M
W
(b) M: p = 0.159 W: p = 0.67
Log cortisol, µg/24h
M
W
age, years
(c) TR= −0.1 nout= 1 sk= 0.02 ku= −0.1
TR cortisol, µg/24h
M
(d) TR= −0.2 nout= 2 sk= −0.07 ku= −0.1
TR cortisol, µg/24h
W
(e) D vs N: delta= 0.51 p = 0
Log cortisol, µg/h
day M
night M
(f) D vs N: delta= 0.53 p = 0
Log cortisol, µg/h
day W
night W
(g) M : rho= 0.563 n= 455
TR cortisol, µg/h night
TR cortisol, µg/h day
(h) W : rho= 0.516 n= 379
TR cortisol, µg/h night
TR cortisol, µg/h day

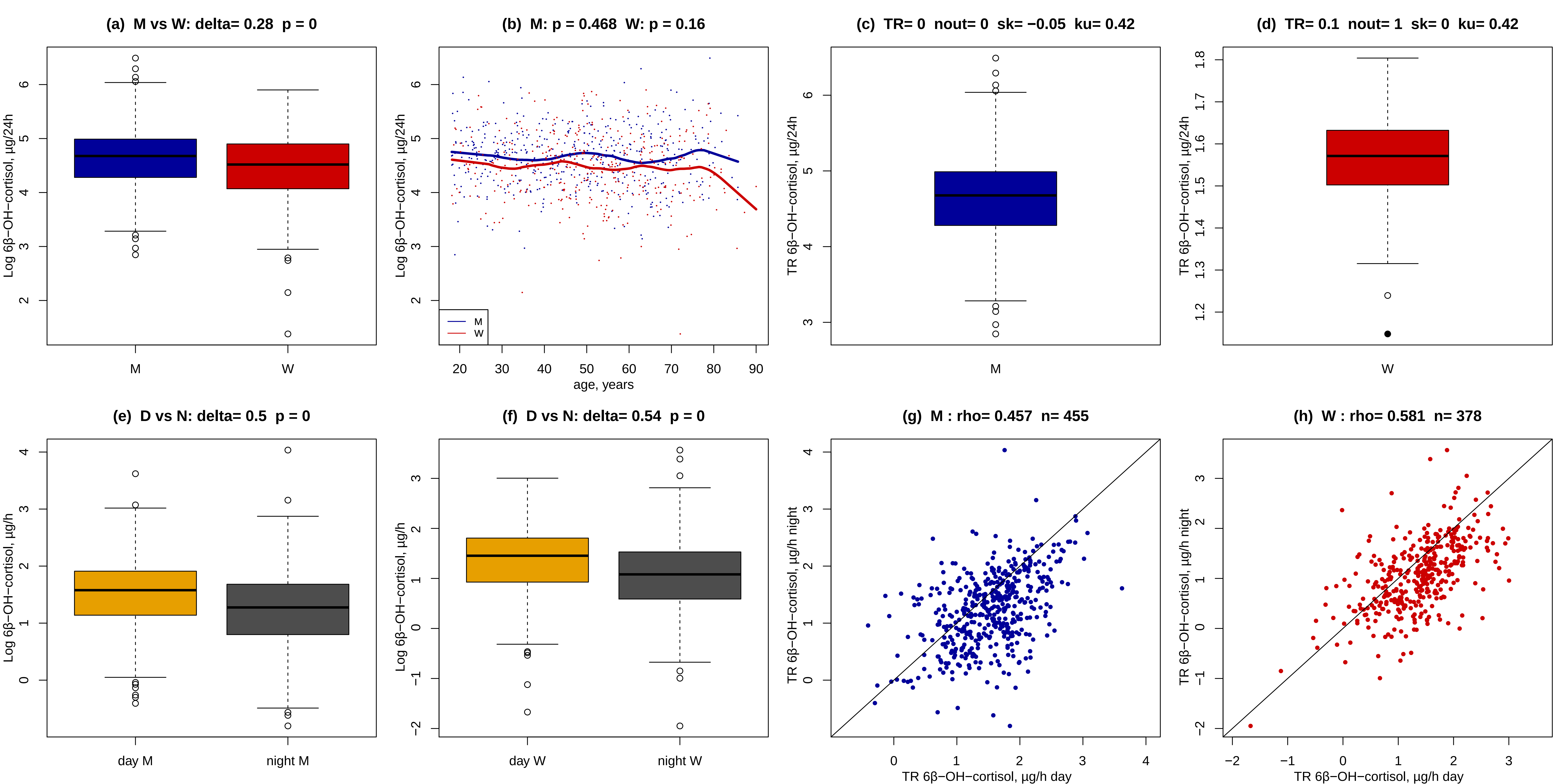

(a) M vs W: delta= 0.28 p = 0
Log 6β−OH−cortisol, µg/24h
M
W
(b) M: p = 0.468 W: p = 0.16
Log 6β−OH−cortisol, µg/24h
M
W
age, years
(c) TR= 0 nout= 0 sk= −0.05 ku= 0.42
TR 6β−OH−cortisol, µg/24h
M
(d) TR= 0.1 nout= 1 sk= 0 ku= 0.42
TR 6β−OH−cortisol, µg/24h
W
(e) D vs N: delta= 0.5 p = 0
Log 6β−OH−cortisol, µg/h
day M
night M
(f) D vs N: delta= 0.54 p = 0
Log 6β−OH−cortisol, µg/h
day W
night W
(g) M : rho= 0.457 n= 455
TR 6β−OH−cortisol, µg/h night
TR 6β−OH−cortisol, µg/h day
(h) W : rho= 0.581 n= 378
TR 6β−OH−cortisol, µg/h night
TR 6β−OH−cortisol, µg/h day

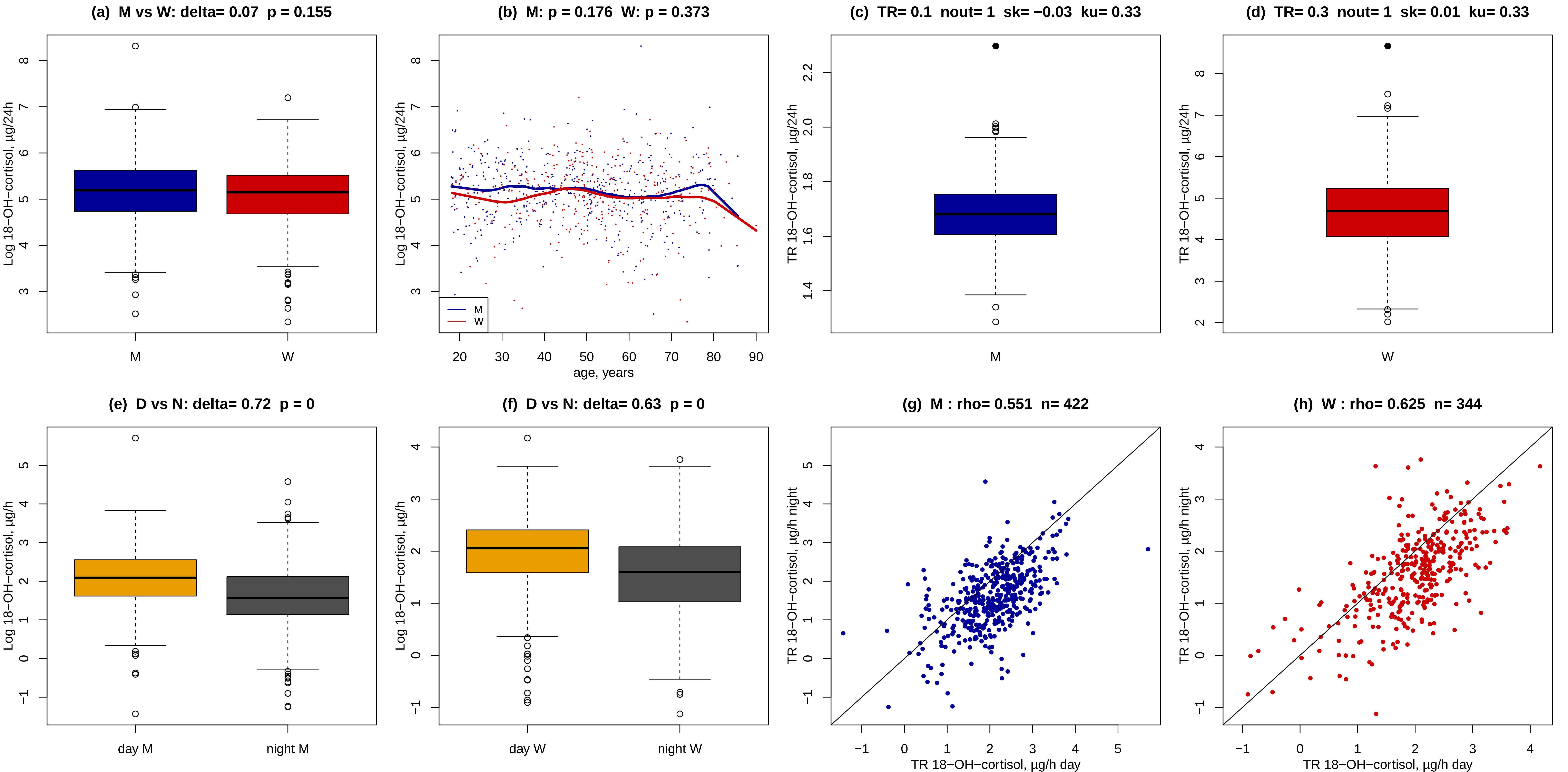

(a) M vs W: delta= 0.07 p = 0.155
Log 18−OH−cortisol, µg/24h
M
W
(b) M: p = 0.176 W: p = 0.373
Log 18−OH−cortisol, µg/24h
M
W
age, years
(c) TR= 0.1 nout= 1 sk= −0.03 ku= 0.33
TR 18−OH−cortisol, µg/24h
M
(d) TR= 0.3 nout= 1 sk= 0.01 ku= 0.33
TR 18−OH−cortisol, µg/24h
W
(e) D vs N: delta= 0.72 p = 0
Log 18−OH−cortisol, µg/h
day M
night M
(f) D vs N: delta= 0.63 p = 0
Log 18−OH−cortisol, µg/h
day W
night W
(g) M : rho= 0.551 n= 422
TR 18−OH−cortisol, µg/h night
TR 18−OH−cortisol, µg/h day
(h) W : rho= 0.625 n= 344
TR 18−OH−cortisol, µg/h night
TR 18−OH−cortisol, µg/h day

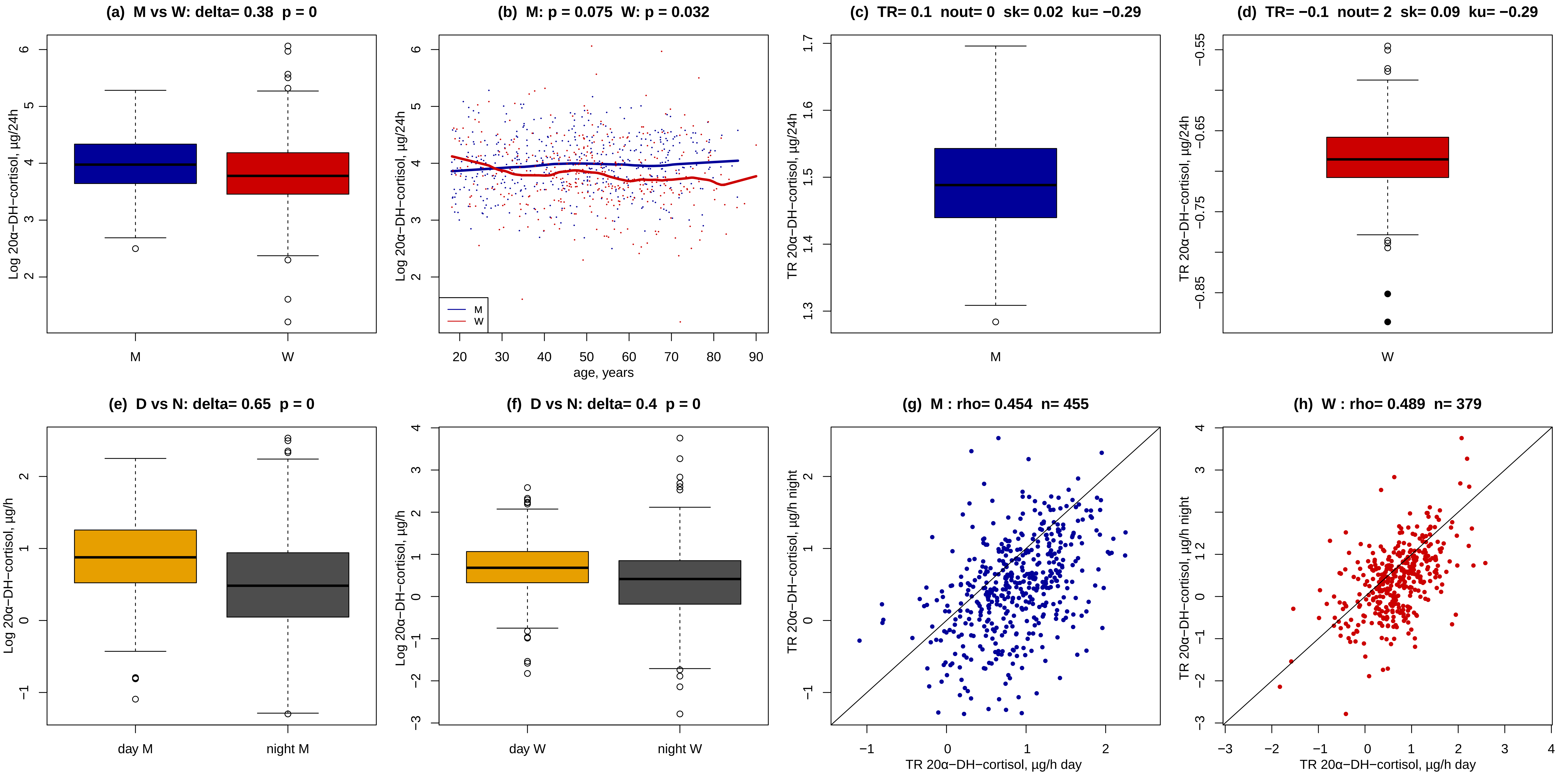
(a) M vs W: delta= 0.38 p = 0
Log 20α−DH−cortisol, µg/24h
M
W
(b) M: p = 0.075 W: p = 0.032
Log 20α−DH−cortisol, µg/24h
M
W
age, years
(c) TR= 0.1 nout= 0 sk= 0.02 ku= −0.29
TR 20α−DH−cortisol, µg/24h
M
(d) TR= −0.1 nout= 2 sk= 0.09 ku= −0.29
TR 20α−DH−cortisol, µg/24h
W
(e) D vs N: delta= 0.65 p = 0
Log 20α−DH−cortisol, µg/h
day M
night M
(f) D vs N: delta= 0.4 p = 0
Log 20α−DH−cortisol, µg/h
day W
night W
(g) M : rho= 0.454 n= 455
TR 20α−DH−cortisol, µg/h night
TR 20α−DH−cortisol, µg/h day
(h) W : rho= 0.489 n= 379
TR 20α−DH−cortisol, µg/h night
TR 20α−DH−cortisol, µg/h day

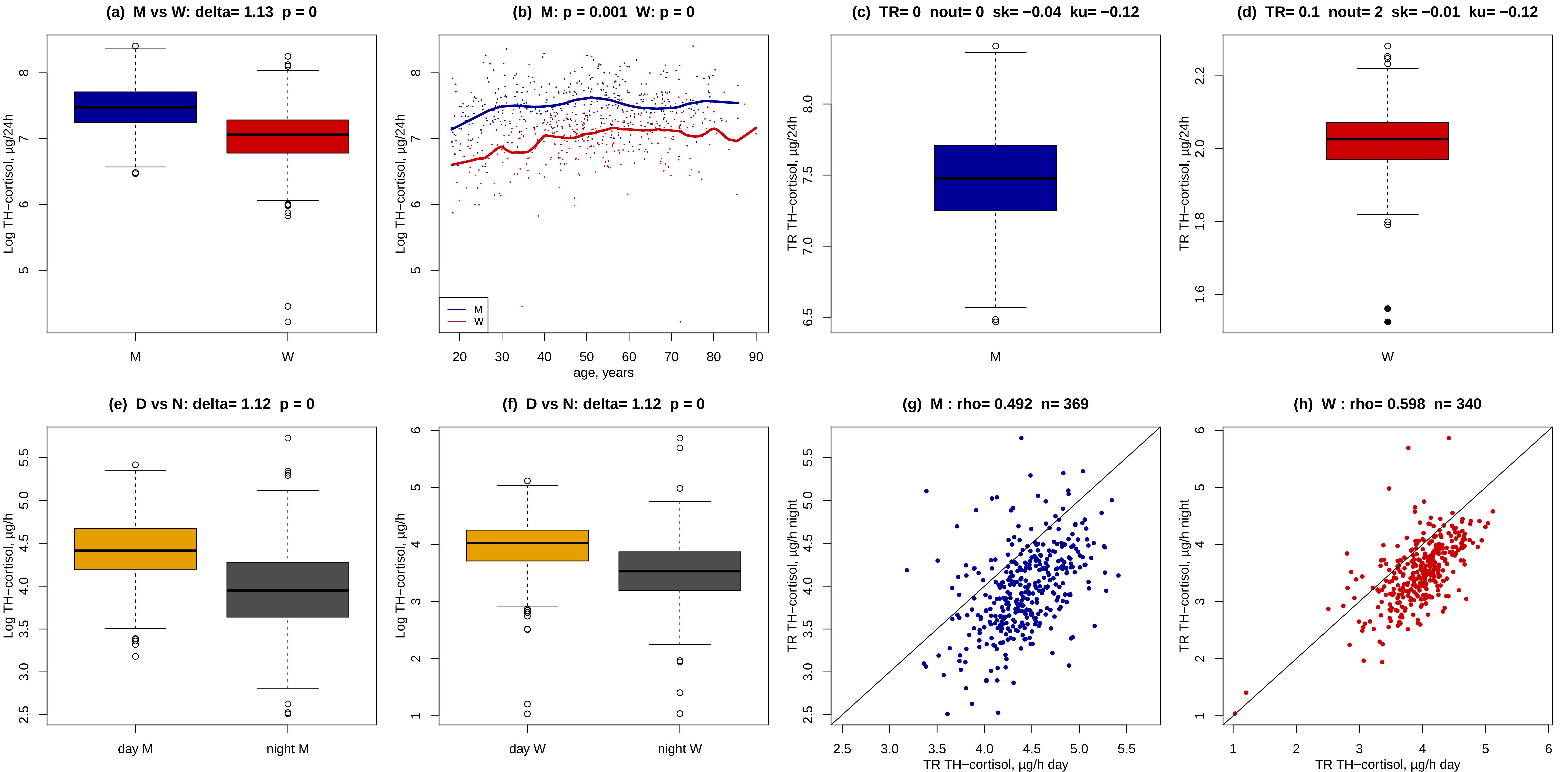

(a) M vs W: delta= 1.13 p = 0
Log TH−cortisol, µg/24h
M
W
(b) M: p = 0.001 W: p = 0
Log TH−cortisol, µg/24h
age, years
M
W
(c) TR= 0 nout= 0 sk= −0.04 ku= −0.12
TR TH−cortisol, µg/24h
M
(d) TR= 0.1 nout= 2 sk= −0.01 ku= −0.12
TR TH−cortisol, µg/24h
W
(e) D vs N: delta= 1.12 p = 0
Log TH−cortisol, µg/h
day M
night M
(f) D vs N: delta= 1.12 p = 0
Log TH−cortisol, µg/h
day W
night W
(g) M : rho= 0.492 n= 369
TR TH−cortisol, µg/h night
TR TH−cortisol, µg/h day
(h) W : rho= 0.598 n= 340
TR TH−cortisol, µg/h night
TR TH−cortisol, µg/h day

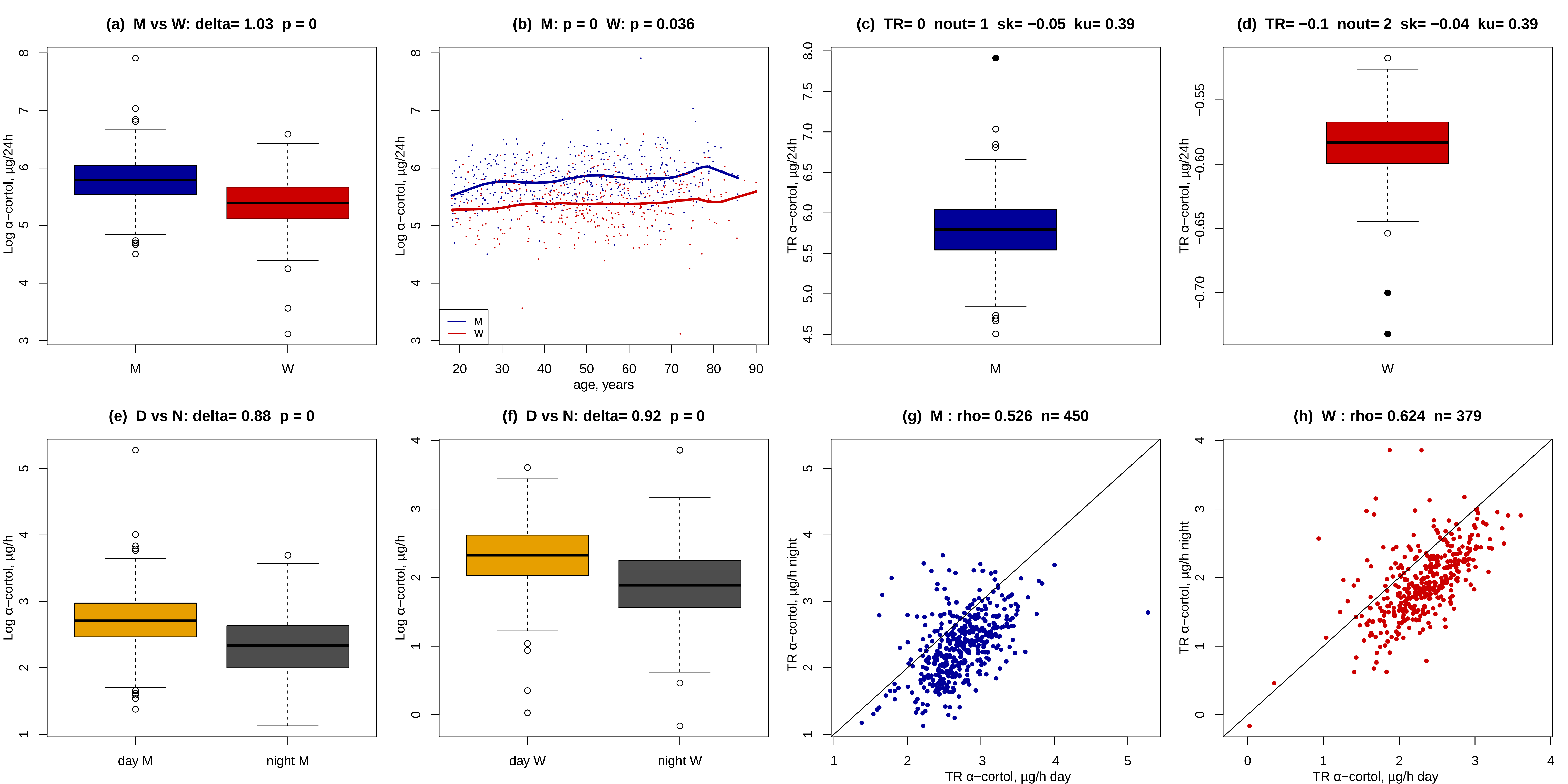
(a) M vs W: delta= 1.03 p = 0
Log α−cortol, μg/24h
M
W
(b) M: p = 0 W: p = 0.036
Log α−cortol, μg/24h
age, years
M
W
(c) TR= 0 nout= 1 sk= −0.05 ku= 0.39
TR α−cortol, μg/24h
M
(d) TR= −0.1 nout= 2 sk= −0.04 ku= 0.39
TR α−cortol, μg/24h
W
(e) D vs N: delta= 0.88 p = 0
Log α−cortol, μg/h
day M
night M
(f) D vs N: delta= 0.92 p = 0
Log α−cortol, μg/h
day W
night W
(g) M : rho= 0.526 n= 450
TR α−cortol, μg/h night
TR α−cortol, μg/h day
(h) W : rho= 0.624 n= 379
TR α−cortol, μg/h night
TR α−cortol, μg/h day

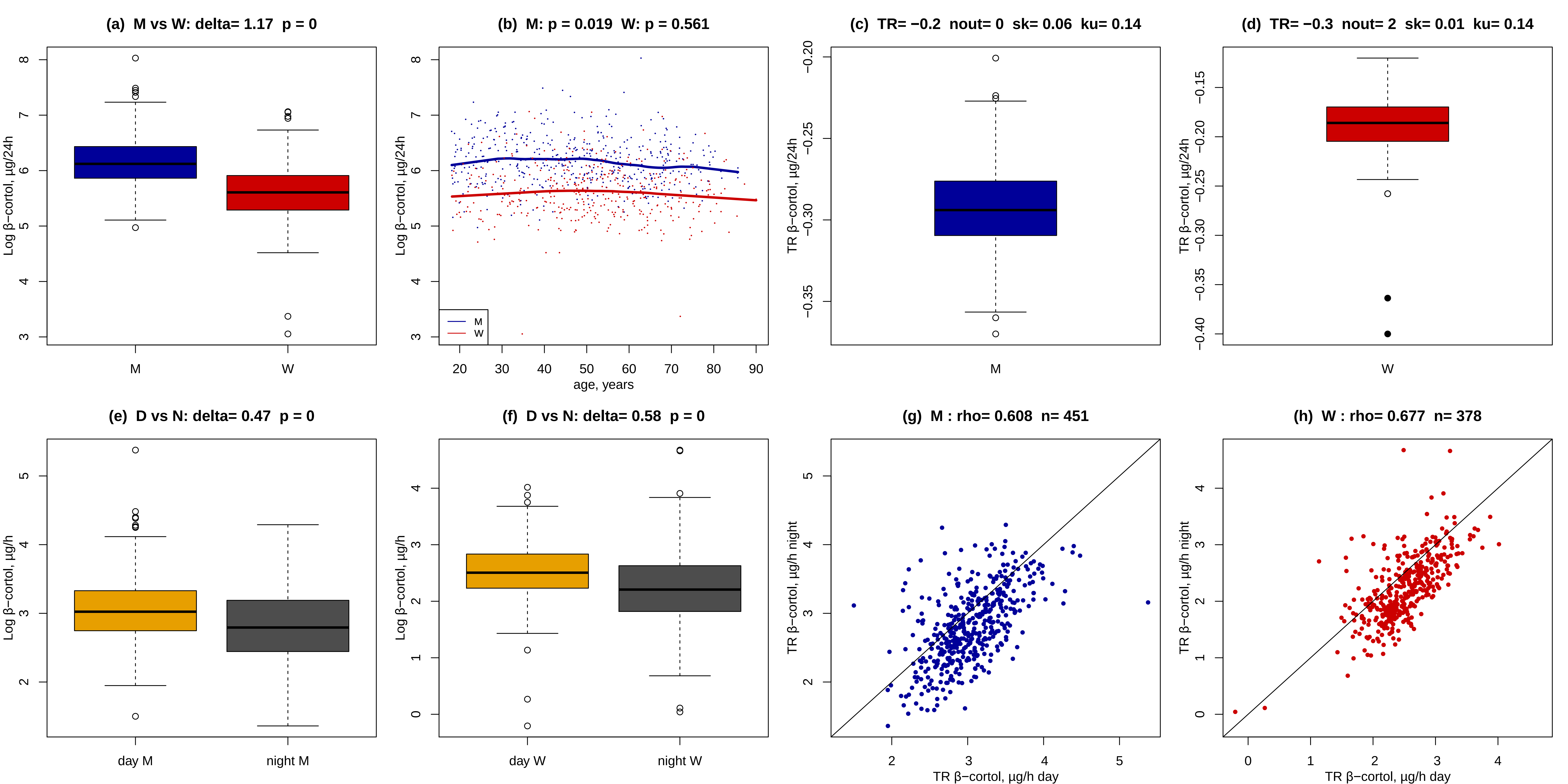
(a) M vs W: delta= 1.17 p = 0
Log β−cortol, μg/24h
M
W
(b) M: p = 0.019 W: p = 0.561
Log β−cortol, μg/24h
M
W
age, years
(c) TR= −0.2 nout= 0 sk= 0.06 ku= 0.14
TR β−cortol, μg/24h
M
(d) TR= −0.3 nout= 2 sk= 0.01 ku= 0.14
TR β−cortol, μg/24h
W
(e) D vs N: delta= 0.47 p = 0
Log β−cortol, μg/h
day M
night M
(f) D vs N: delta= 0.58 p = 0
Log β−cortol, μg/h
day W
night W
(g) M : rho= 0.608 n= 451
TR β−cortol, μg/h night
TR β−cortol, μg/h day
(h) W : rho= 0.677 n= 378
TR β−cortol, μg/h night
TR β−cortol, μg/h day

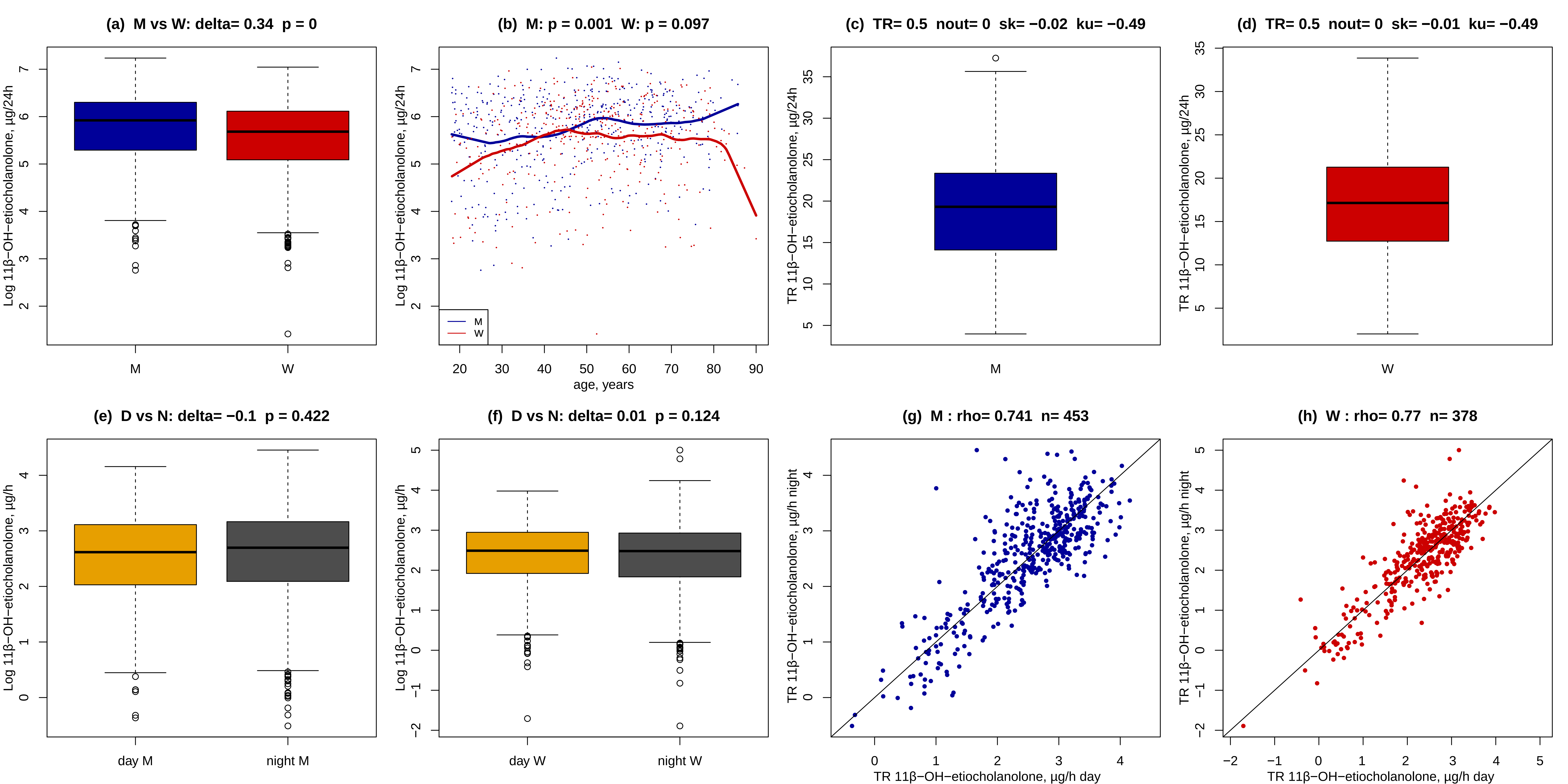

(a) M vs W: delta= 0.34 p = 0
Log 11β−OH−etiocholanolone, µg/24h
M
W
(b) M: p = 0.001 W: p = 0.097
Log 11β−OH−etiocholanolone, µg/24h
age, years
M
W
(c) TR= 0.5 nout= 0 sk= −0.02 ku= −0.49
TR 11β−OH−etiocholanolone, µg/24h
M
(d) TR= 0.5 nout= 0 sk= −0.01 ku= −0.49
TR 11β−OH−etiocholanolone, µg/24h
W
(e) D vs N: delta= −0.1 p = 0.422
Log 11β−OH−etiocholanolone, µg/h
day M
night M
(f) D vs N: delta= 0.01 p = 0.124
Log 11β−OH−etiocholanolone, µg/h
day W
night W
(g) M : rho= 0.741 n= 453
TR 11β−OH−etiocholanolone, µg/h night
TR 11β−OH−etiocholanolone, µg/h day
(h) W : rho= 0.77 n= 378
TR 11β−OH−etiocholanolone, µg/h night
TR 11β−OH−etiocholanolone, µg/h day

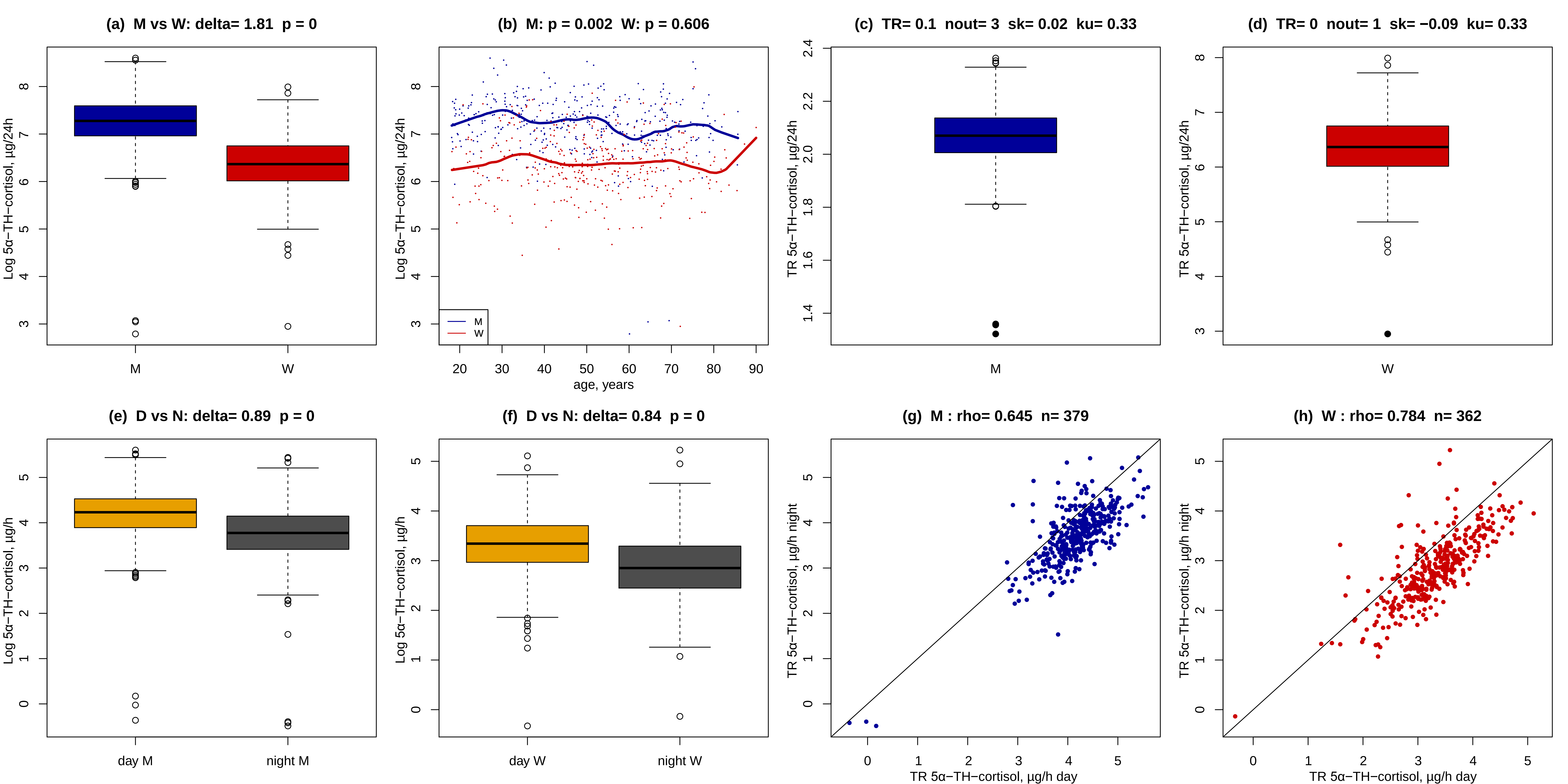

(a) M vs W: delta= 1.81 p = 0
Log 5α−TH−cortisol, µg/24h
M
W
(b) M: p = 0.002 W: p = 0.606
Log 5α−TH−cortisol, µg/24h
M
W
age, years
(c) TR= 0.1 nout= 3 sk= 0.02 ku= 0.33
TR 5α−TH−cortisol, µg/24h
M
(d) TR= 0 nout= 1 sk= −0.09 ku= 0.33
TR 5α−TH−cortisol, µg/24h
W
(e) D vs N: delta= 0.89 p = 0
Log 5α−TH−cortisol, µg/h
day M
night M
(f) D vs N: delta= 0.84 p = 0
Log 5α−TH−cortisol, µg/h
day W
night W
(g) M : rho= 0.645 n= 379
TR 5α−TH−cortisol, µg/h night
TR 5α−TH−cortisol, µg/h day
(h) W : rho= 0.784 n= 362
TR 5α−TH−cortisol, µg/h night
TR 5α−TH−cortisol, µg/h day

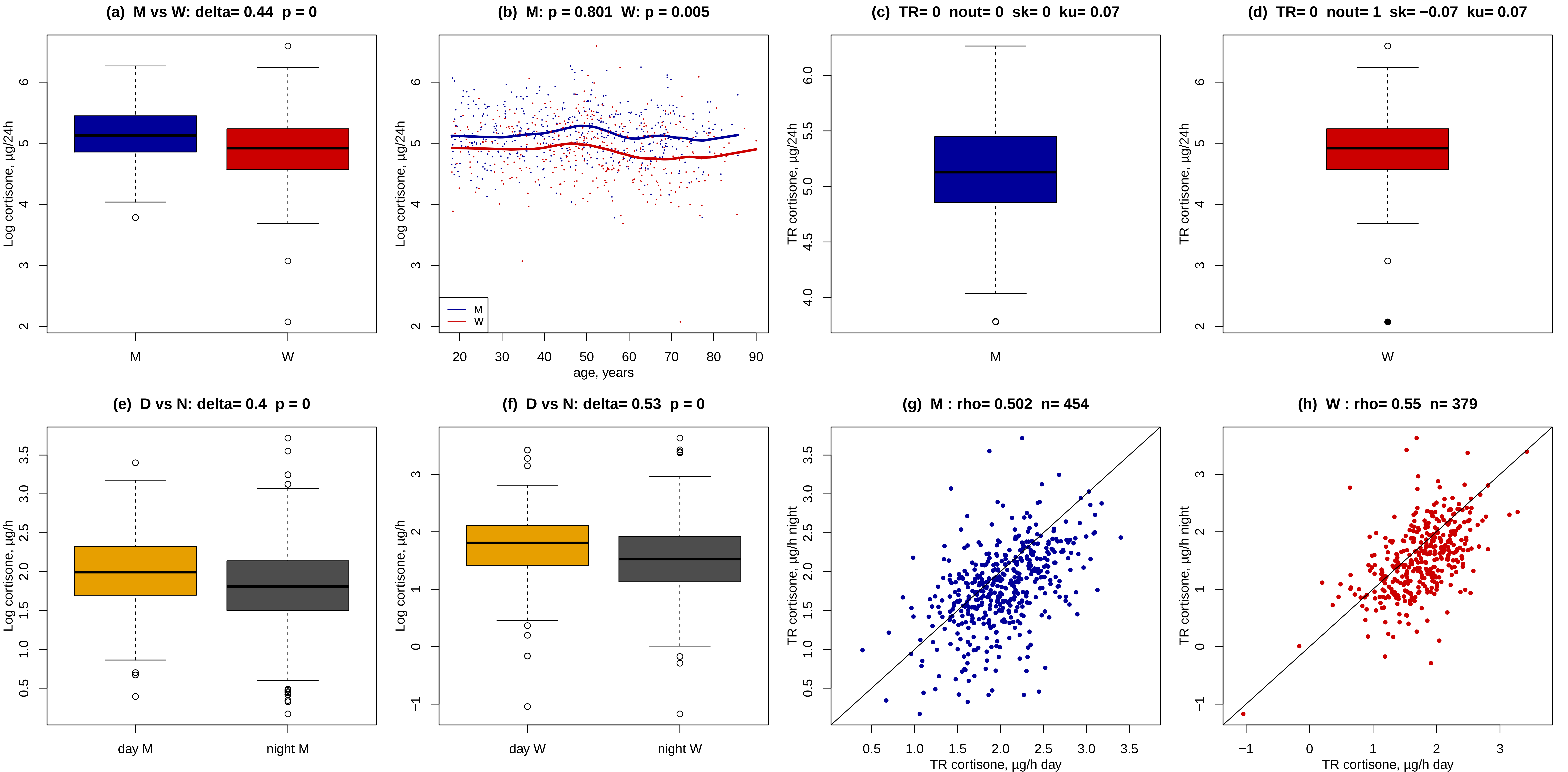

(a) M vs W: delta= 0.44 p = 0
Log cortisone, µg/24h
M
W
(b) M: p = 0.801 W: p = 0.005
Log cortisone, µg/24h
age, years
M
W
(c) TR= 0 nout= 0 sk= 0 ku= 0.07
TR cortisone, µg/24h
M
(d) TR= 0 nout= 1 sk= −0.07 ku= 0.07
TR cortisone, µg/24h
W
(e) D vs N: delta= 0.4 p = 0
Log cortisone, µg/h
day M
night M
(f) D vs N: delta= 0.53 p = 0
Log cortisone, µg/h
day W
night W
(g) M : rho= 0.502 n= 454
TR cortisone, µg/h night
TR cortisone, µg/h day
(h) W : rho= 0.55 n= 379
TR cortisone, µg/h night
TR cortisone, µg/h day

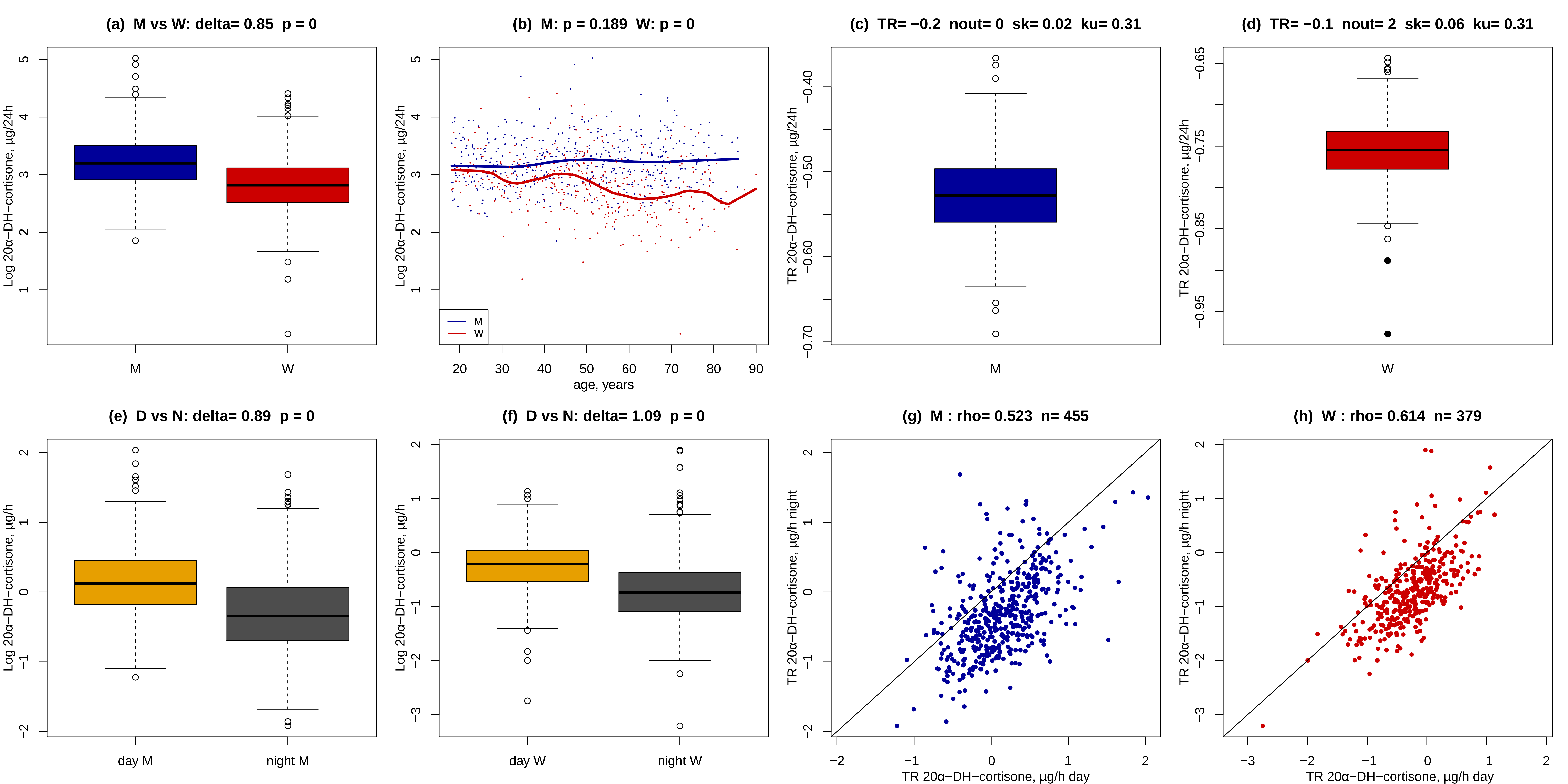
(a) M vs W: delta= 0.85 p = 0
Log 20α−DH−cortisone, µg/24h
M
W
(b) M: p = 0.189 W: p = 0
Log 20α−DH−cortisone, µg/24h
M
W
age, years
(c) TR= −0.2 nout= 0 sk= 0.02 ku= 0.31
TR 20α−DH−cortisone, µg/24h
M
(d) TR= −0.1 nout= 2 sk= 0.06 ku= 0.31
TR 20α−DH−cortisone, µg/24h
W
(e) D vs N: delta= 0.89 p = 0
Log 20α−DH−cortisone, µg/h
day M
night M
(f) D vs N: delta= 1.09 p = 0
Log 20α−DH−cortisone, µg/h
day W
night W
(g) M : rho= 0.523 n= 455
TR 20α−DH−cortisone, µg/h night
TR 20α−DH−cortisone, µg/h day
(h) W : rho= 0.614 n= 379
TR 20α−DH−cortisone, µg/h night
TR 20α−DH−cortisone, µg/h day

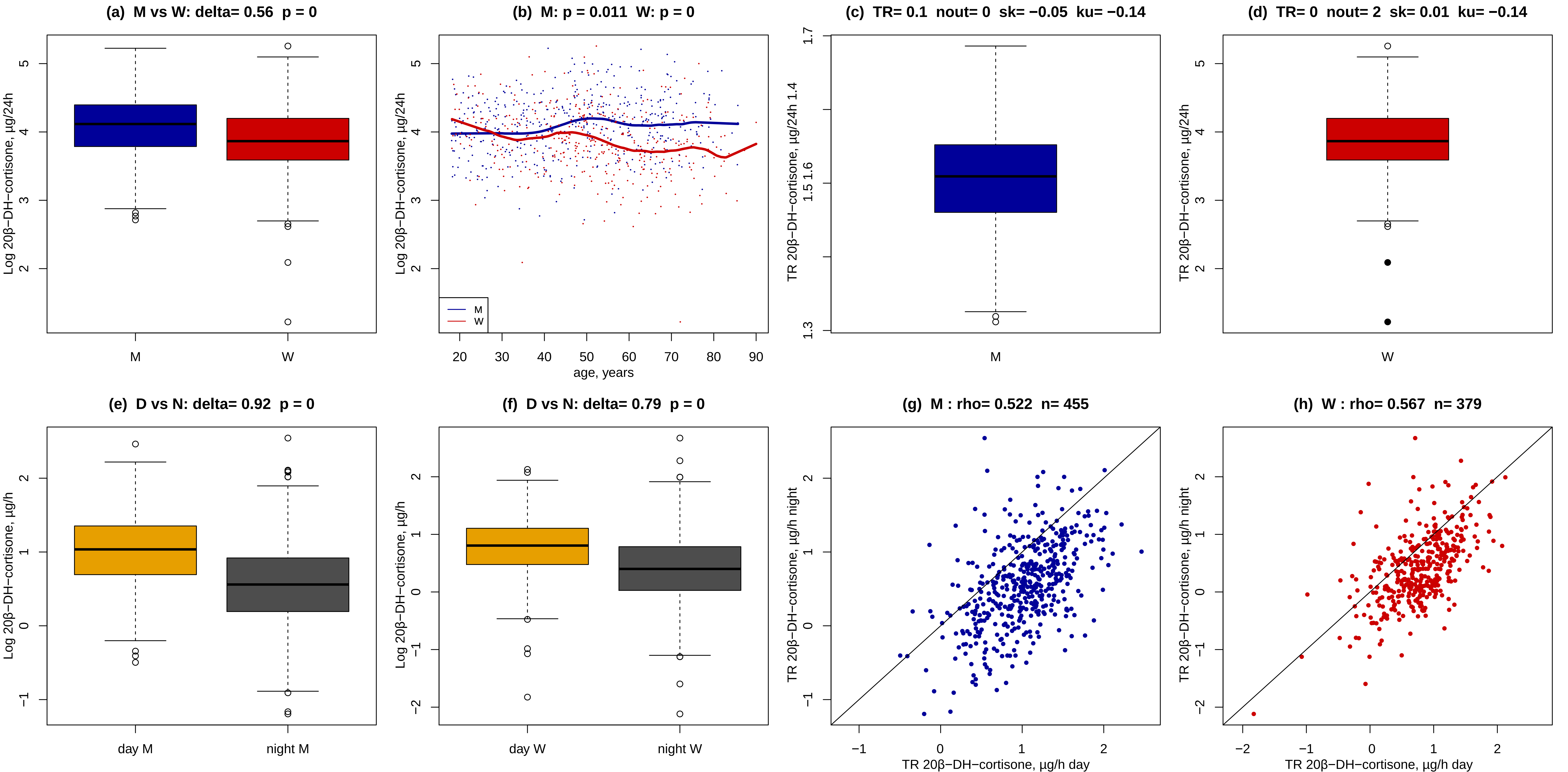

(a) M vs W: delta= 0.56 p = 0
(b) M: p = 0.011 W: p = 0
(c) TR= 0.1 nout= 0 sk= −0.05 ku= −0.14
(d) TR= 0 nout= 2 sk= 0.01 ku= −0.14
(e) D vs N: delta= 0.92 p = 0
(f) D vs N: delta= 0.79 p = 0
(g) M : rho= 0.522 n= 455
(h) W : rho= 0.567 n= 379
Log 20β−DH−cortisone, μg/24h
TR 20β−DH−cortisone, μg/24h
Log 20β−DH−cortisone, μg/h
TR 20β−DH−cortisone, μg/h night
TR 20β−DH−cortisone, μg/h day
age, years
M
W
day M
night M
day W
night W

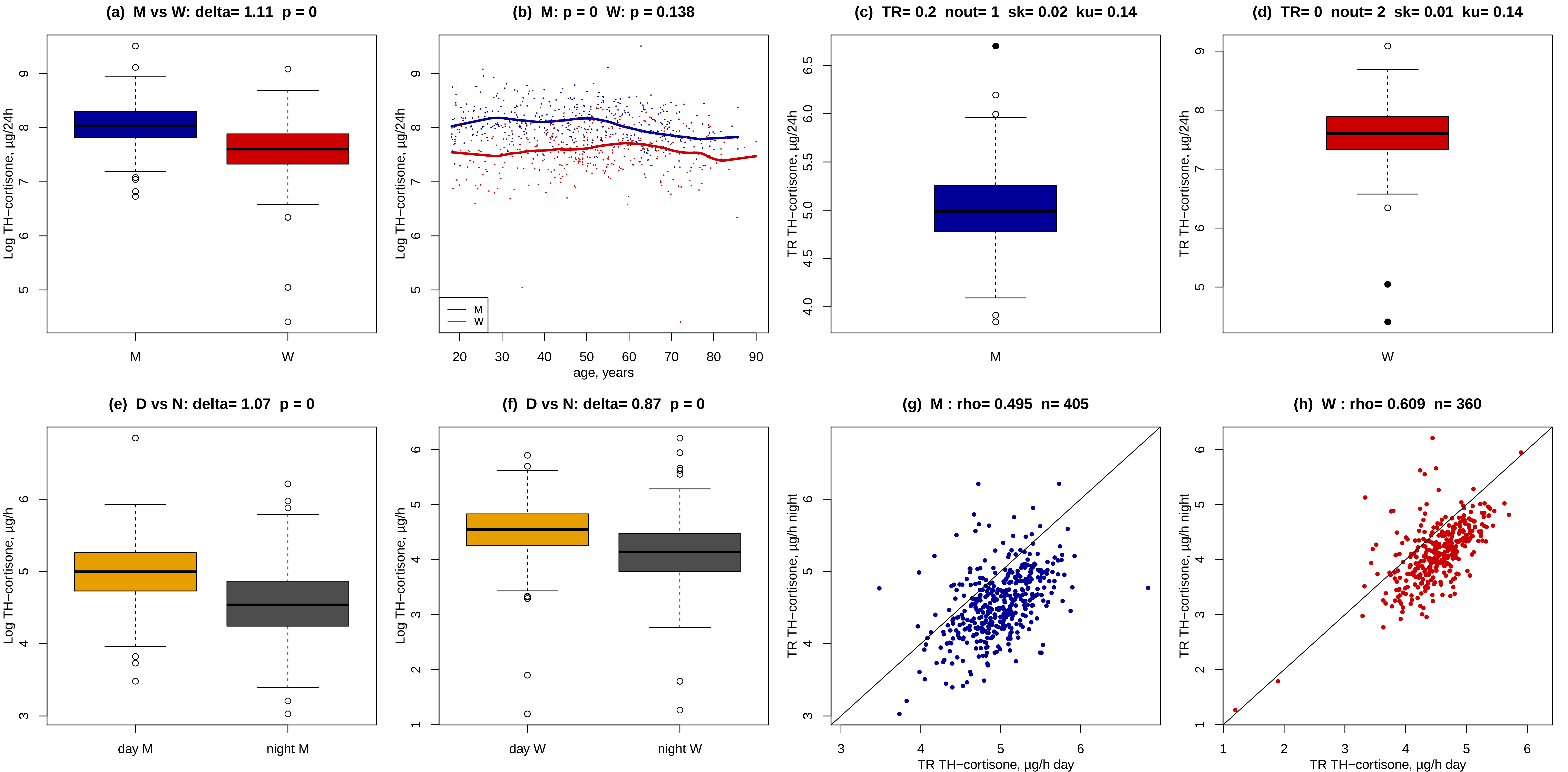
(a) M vs W: delta= 1.11 p = 0
Log TH−cortisone, µg/24h
M
W
(b) M: p = 0 W: p = 0.138
Log TH−cortisone, µg/24h
M
W
age, years
(c) TR= 0.2 nout= 1 sk= 0.02 ku= 0.14
TR TH−cortisone, µg/24h
M
(d) TR= 0 nout= 2 sk= 0.01 ku= 0.14
TR TH−cortisone, µg/24h
W
(e) D vs N: delta= 1.07 p = 0
Log TH−cortisone, µg/h
day M
night M
(f) D vs N: delta= 0.87 p = 0
Log TH−cortisone, µg/h
day W
night W
(g) M : rho= 0.495 n= 405
TR TH−cortisone, µg/h night
TR TH−cortisone, µg/h day
(h) W : rho= 0.609 n= 360
TR TH−cortisone, µg/h night
TR TH−cortisone, µg/h day

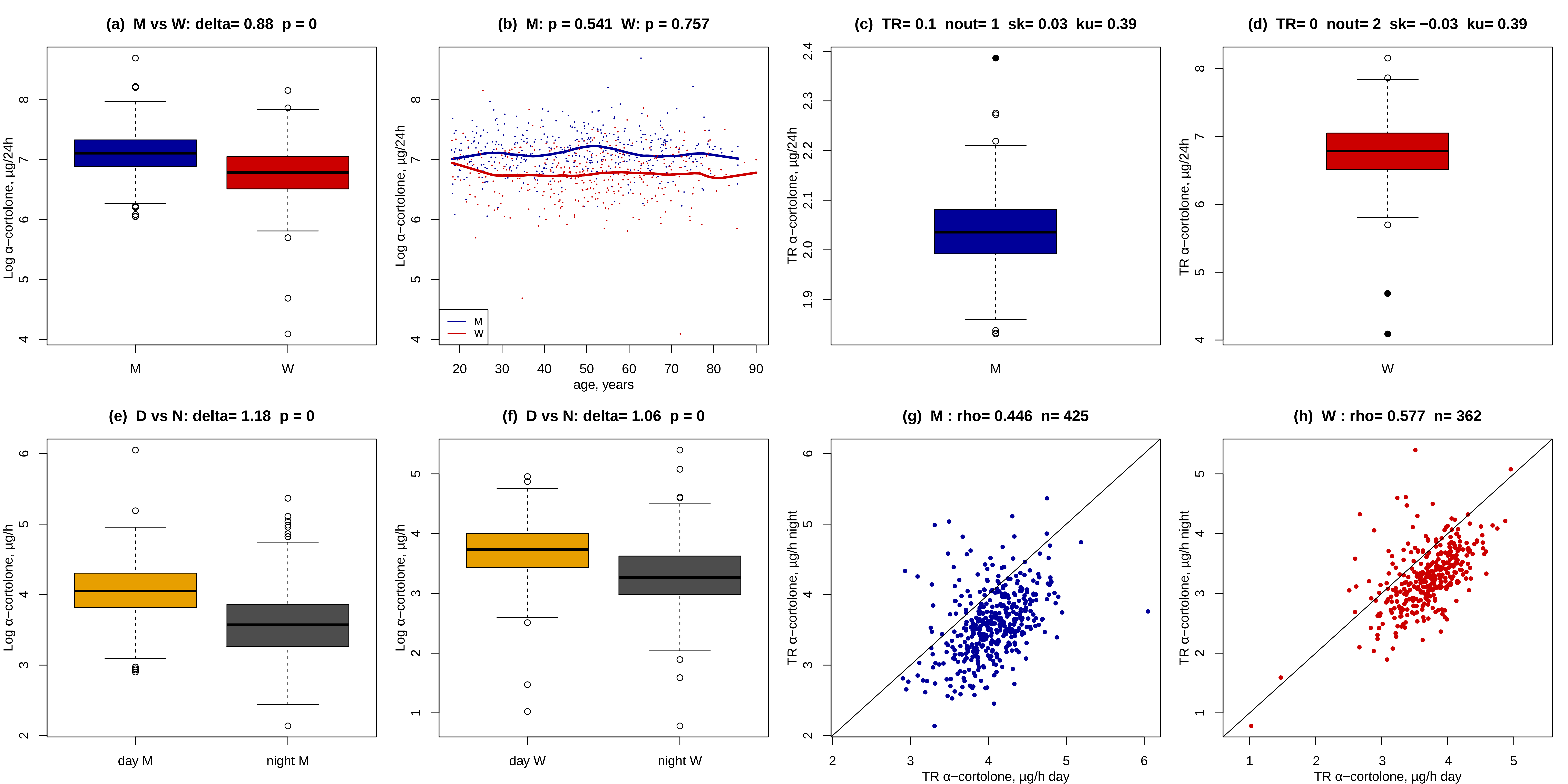
(a) M vs W: delta= 0.88 p = 0
Log α−cortolone, µg/24h
M
W
(b) M: p = 0.541 W: p = 0.757
Log α−cortolone, µg/24h
M
W
age, years
(c) TR= 0.1 nout= 1 sk= 0.03 ku= 0.39
TR α−cortolone, µg/24h
M
(d) TR= 0 nout= 2 sk= −0.03 ku= 0.39
TR α−cortolone, µg/24h
W
(e) D vs N: delta= 1.18 p = 0
Log α−cortolone, µg/h
day M
night M
(f) D vs N: delta= 1.06 p = 0
Log α−cortolone, µg/h
day W
night W
(g) M : rho= 0.446 n= 425
TR α−cortolone, µg/h night
TR α−cortolone, µg/h day
(h) W : rho= 0.577 n= 362
TR α−cortolone, µg/h night
TR α−cortolone, µg/h day

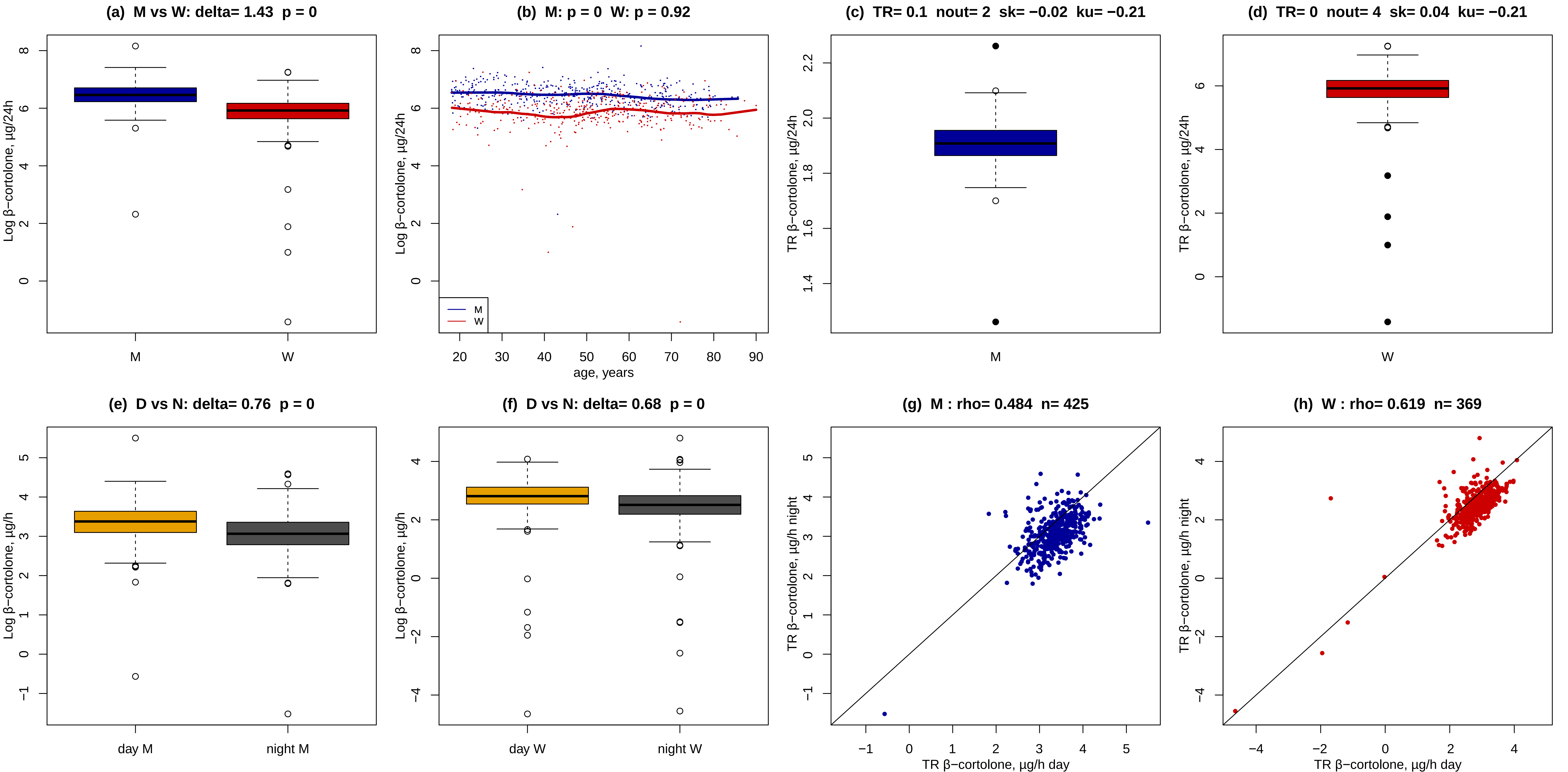

(a) M vs W: delta= 1.43 p = 0
Log β−cortolone, μg/24h
M
W
(b) M: p = 0 W: p = 0.92
Log β−cortolone, μg/24h
M
W
age, years
(c) TR= 0.1 nout= 2 sk= −0.02 ku= −0.21
TR β−cortolone, μg/24h
M
(d) TR= 0 nout= 4 sk= 0.04 ku= −0.21
TR β−cortolone, μg/24h
W
(e) D vs N: delta= 0.76 p = 0
Log β−cortolone, μg/h
day M
night M
(f) D vs N: delta= 0.68 p = 0
Log β−cortolone, μg/h
day W
night W
(g) M : rho= 0.484 n= 425
TR β−cortolone, μg/h night
TR β−cortolone, μg/h day
(h) W : rho= 0.619 n= 369
TR β−cortolone, μg/h night
TR β−cortolone, μg/h day

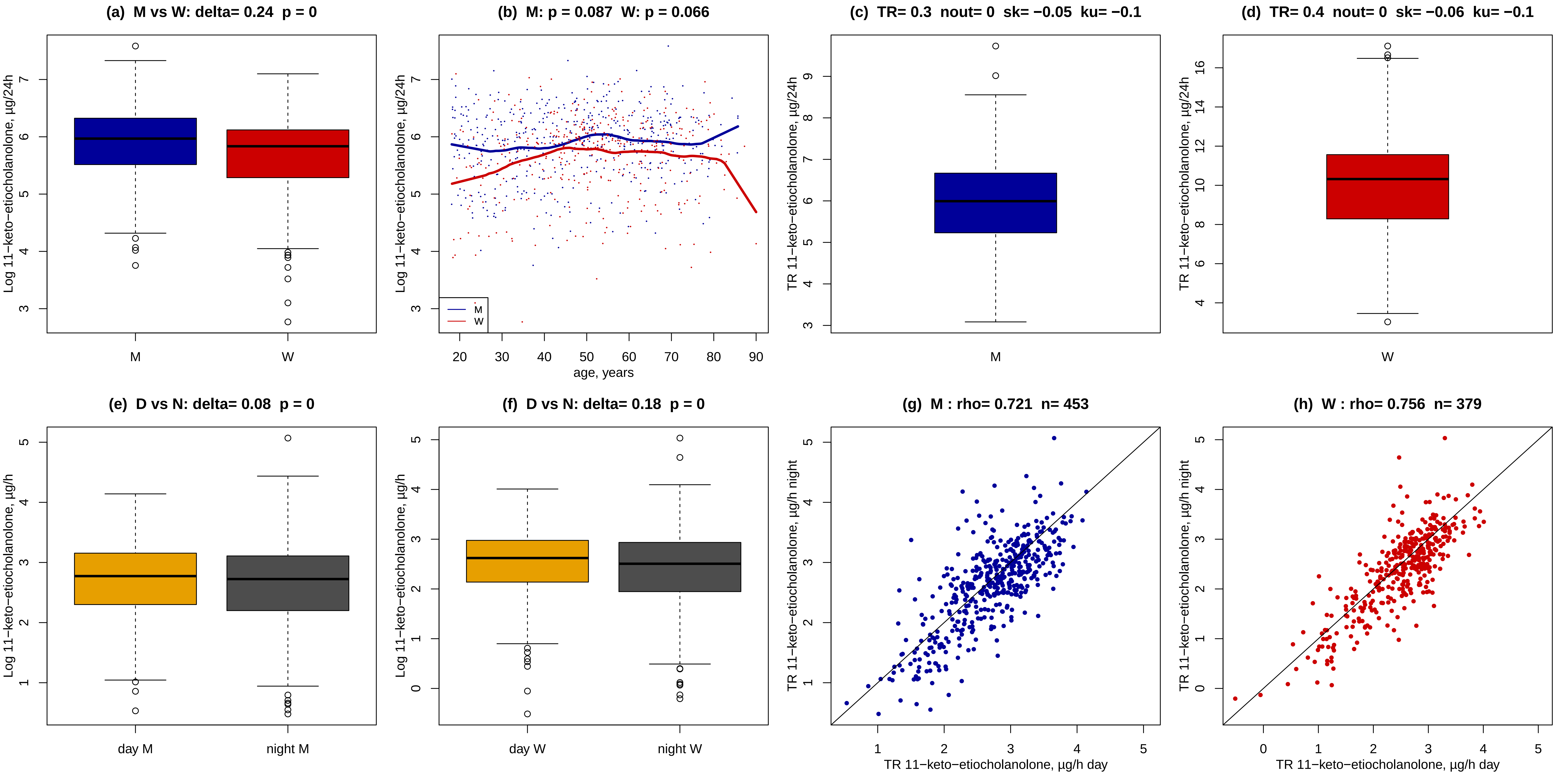

(a) M vs W: delta= 0.24 p = 0
Log 11−keto−etiocholanolone, µg/24h
M
W
(b) M: p = 0.087 W: p = 0.066
age, years
(c) TR= 0.3 nout= 0 sk= −0.05 ku= −0.1
TR 11−keto−etiocholanolone, µg/24h
(d) TR= 0.4 nout= 0 sk= −0.06 ku= −0.1
(e) D vs N: delta= 0.08 p = 0
Log 11−keto−etiocholanolone, µg/h
day M
night M
(f) D vs N: delta= 0.18 p = 0
day W
night W
(g) M : rho= 0.721 n= 453
TR 11−keto−etiocholanolone, µg/h night
TR 11−keto−etiocholanolone, µg/h day
(h) W : rho= 0.756 n= 379
