# Supplementary material for: Reference intervals for the urinary steroid metabolome: The impact of sex, age, day and night time on human adult steroidogenesis
Source: PLoS One. 2019 Mar 29;14(3):e0214549. doi: 10.1371/journal.pone.0214549 (PMC6440635; doi:10.1371/journal.pone.0214549)
Supplement: S4 Fig — Reference curves of the 40 steroid compounds measured in urine are shown including one steroid compound per page. The percentiles 2.5, 10, 25, 50, 75, 90 and 97.5 of the steroid compounds in function of age and sex are shown on a log-scale. To improve comparison the same scale has been used for men and women. (PDF) [file pone.0214549.s004.pdf]

Supporting Figure 4. Reference curves of steroid compounds. Reference curves of the 40 steroid compounds measured in urine are shown including one steroid compound per page. The percentiles 2.5, 10, 25, 50, 75, 90 and 97.5 of the steroid compounds in function of age and sex are shown on a log-scale. To improve comparison the same scale has been used for men and women.

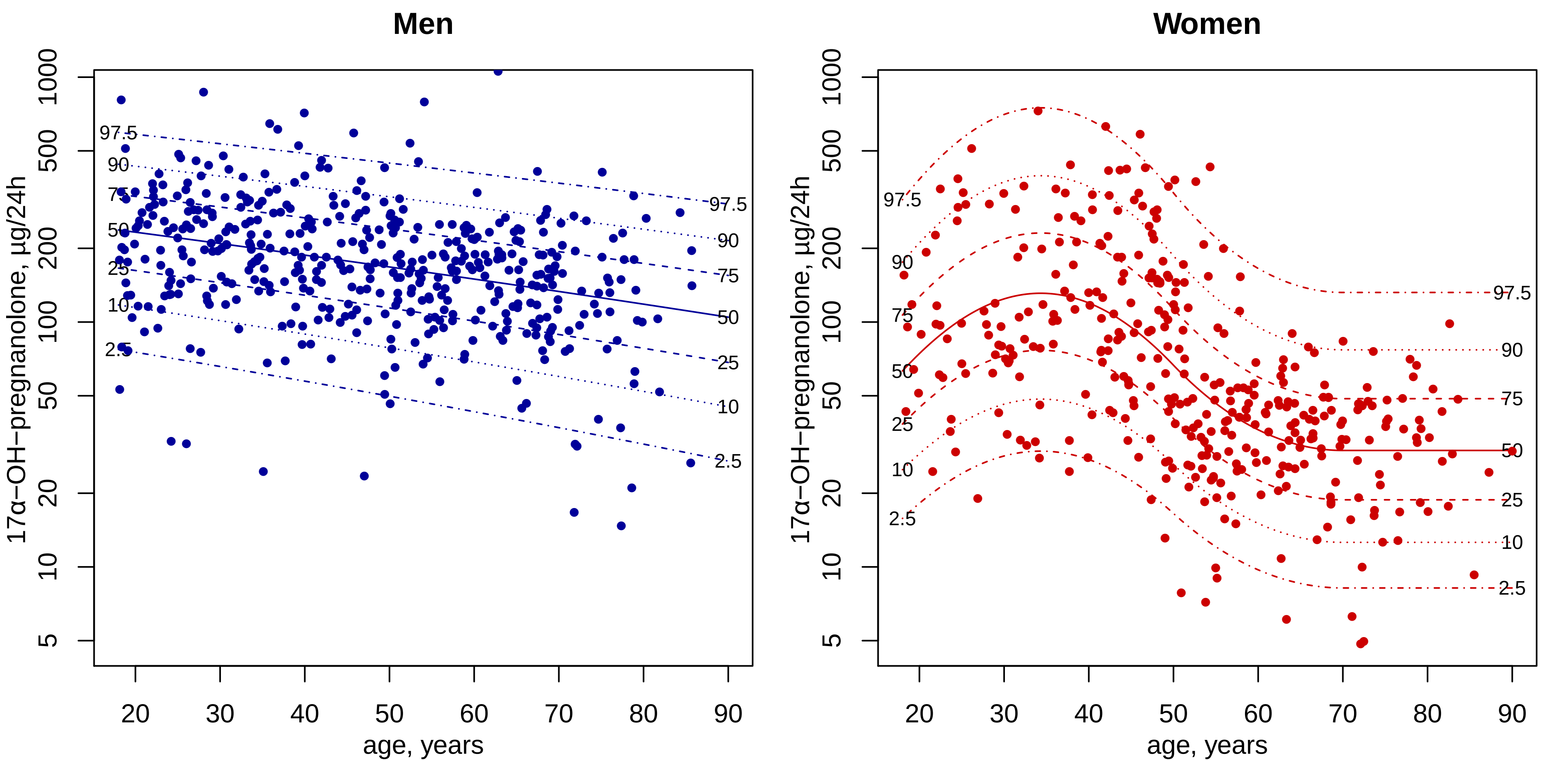

Men
Women
17α−OH−pregnanolone, µg/24h
age, years
1000
500
200
100
50
20
10
5
20
30
40
50
60
70
80
90
97.5
90
75
50
25
10
2.5

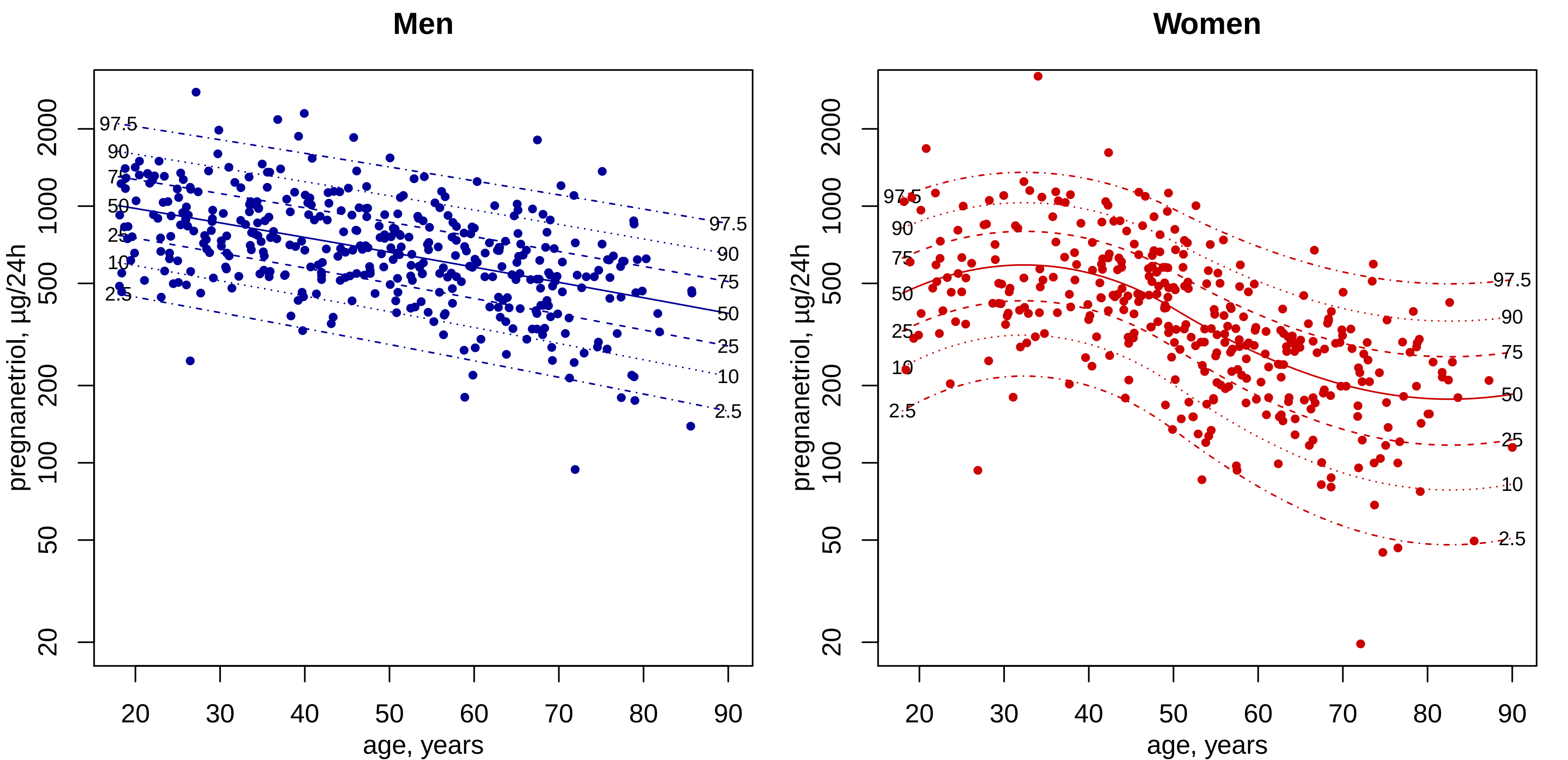

Men
Women
pregnanetriol, µg/24h
age, years
20
50
100
200
500
1000
2000
20
30
40
50
60
70
80
90
97.5
90
75
50
25
10
2.5

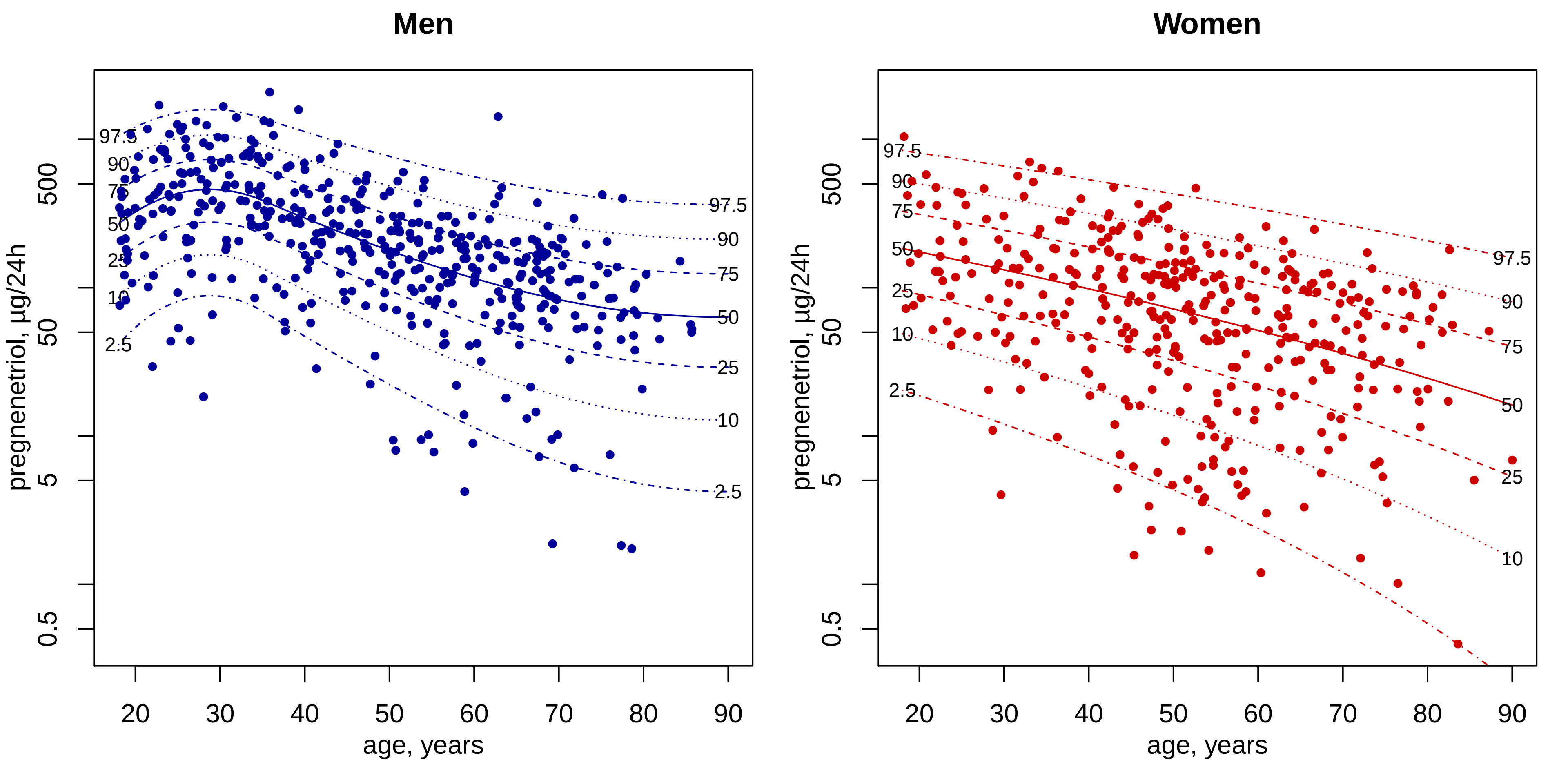
Men
Women
pregnenetriol, µg/24h
age, years
0.5
5
50
500
20
30
40
50
60
70
80
90
97.5
90
75
50
25
10
2.5

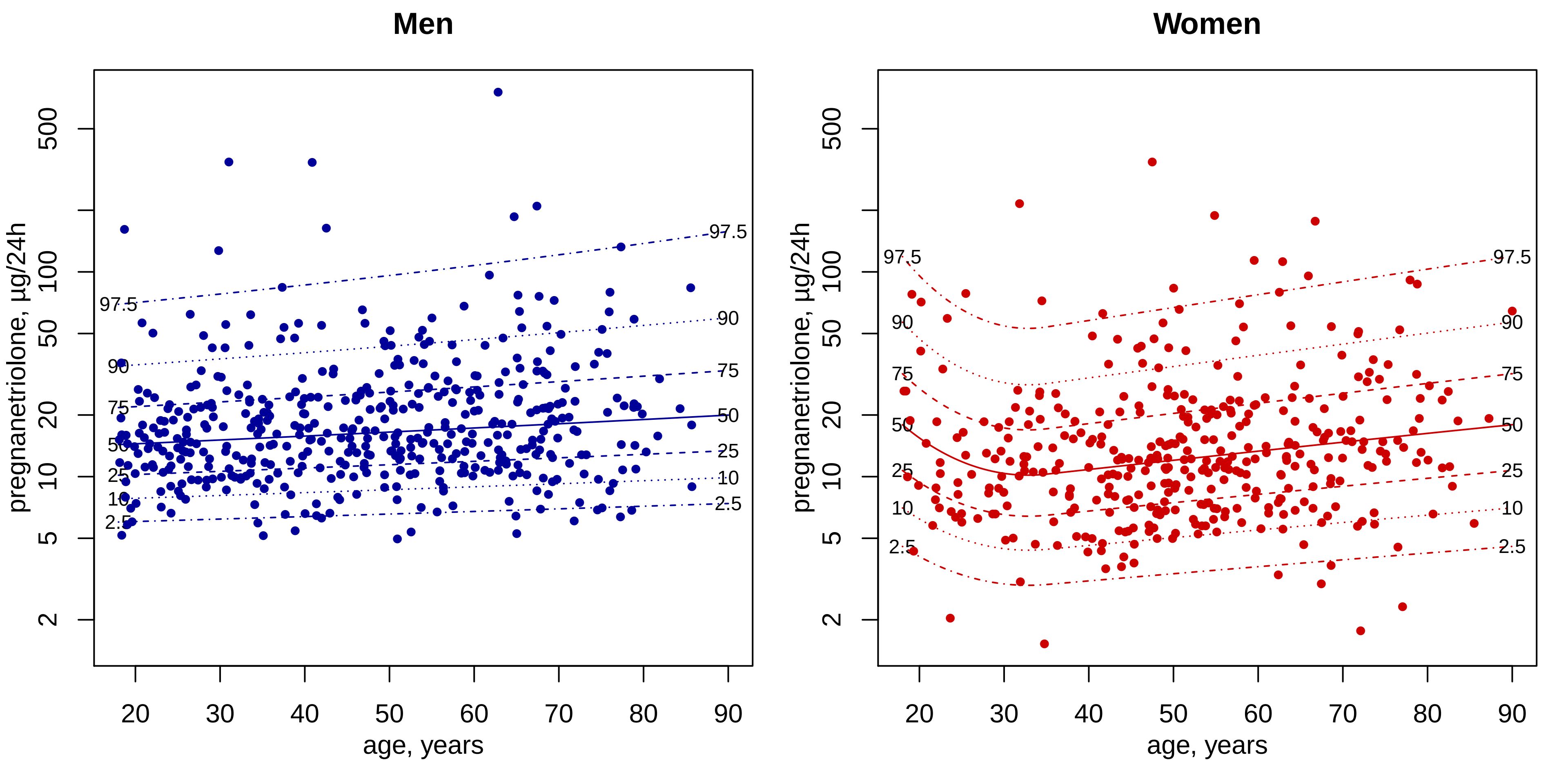

Men
Women
pregnanetriolone, µg/24h
age, years
500
100
50
20
10
5
2
20
30
40
50
60
70
80
90
97.5
90
75
50
25
10
2.5

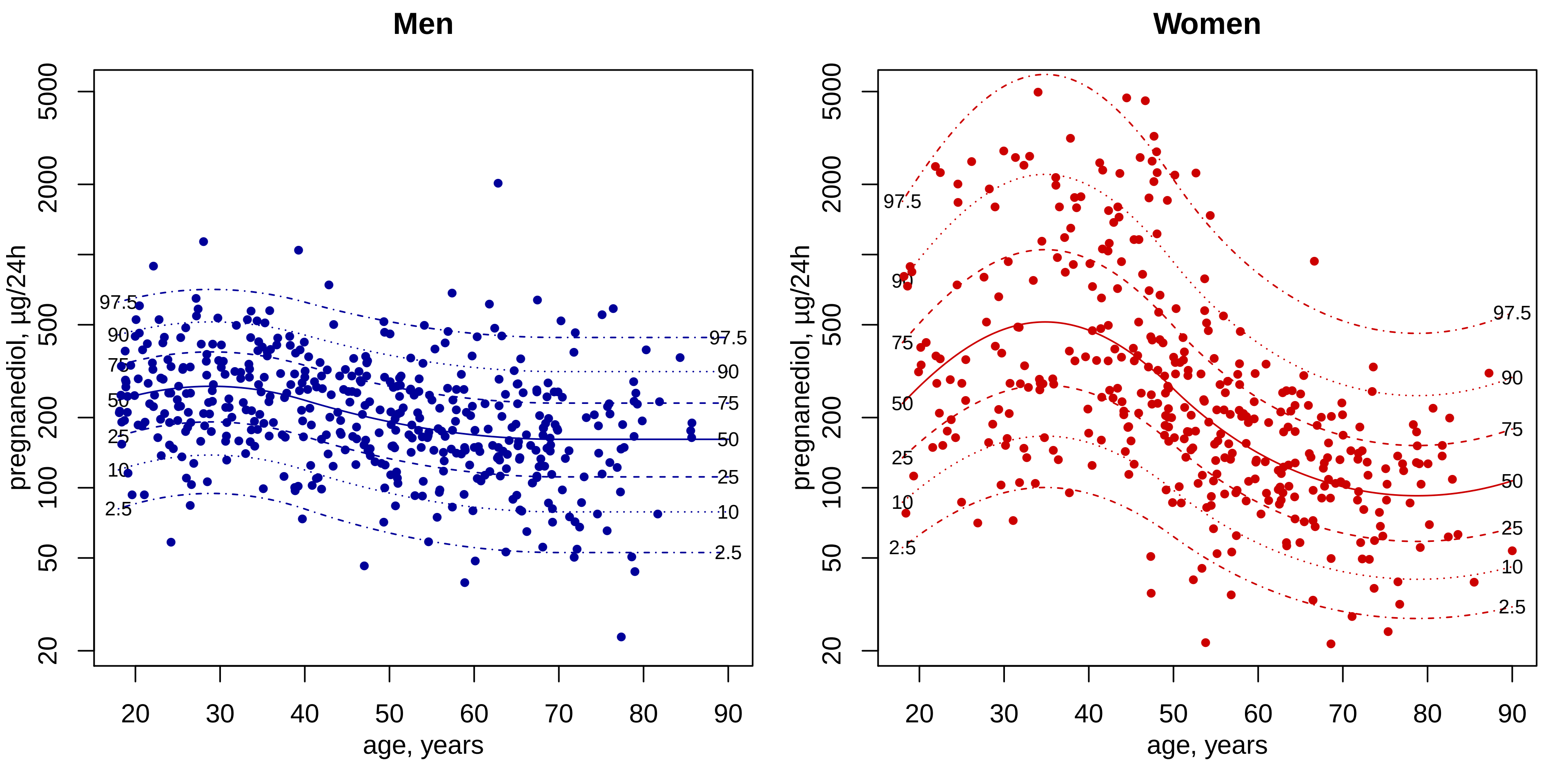

Men
Women
pregnanediol, µg/24h
age, years
5000
2000
500
200
100
50
20
20
30
40
50
60
70
80
90
97.5
90
75
50
25
10
2.5

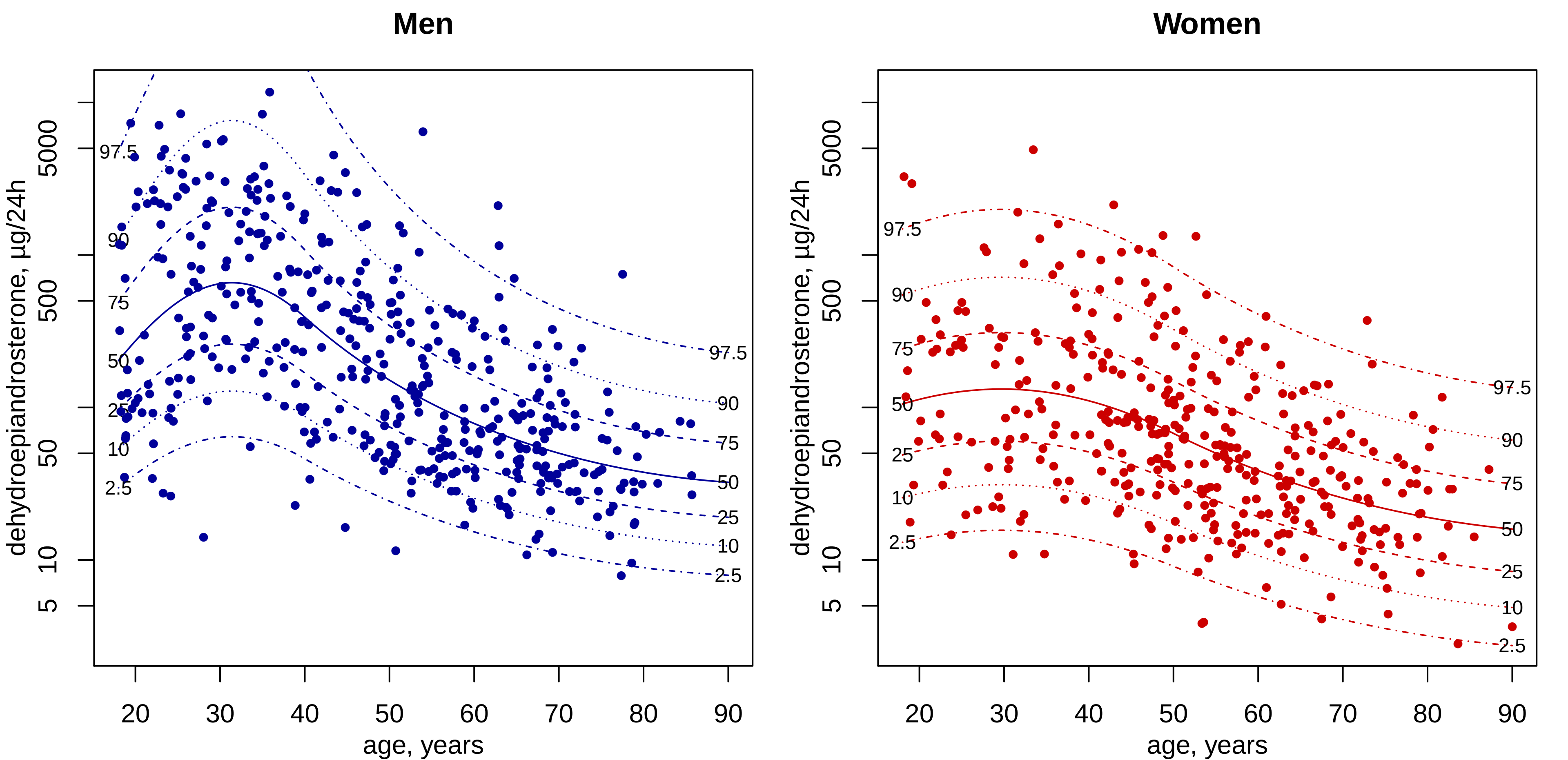

Men
Women
dehydroepiandrosterone, µg/24h
5
10
50
500
5000
20
30
40
50
60
70
80
90
age, years
97.5
90
75
50
25
10
2.5

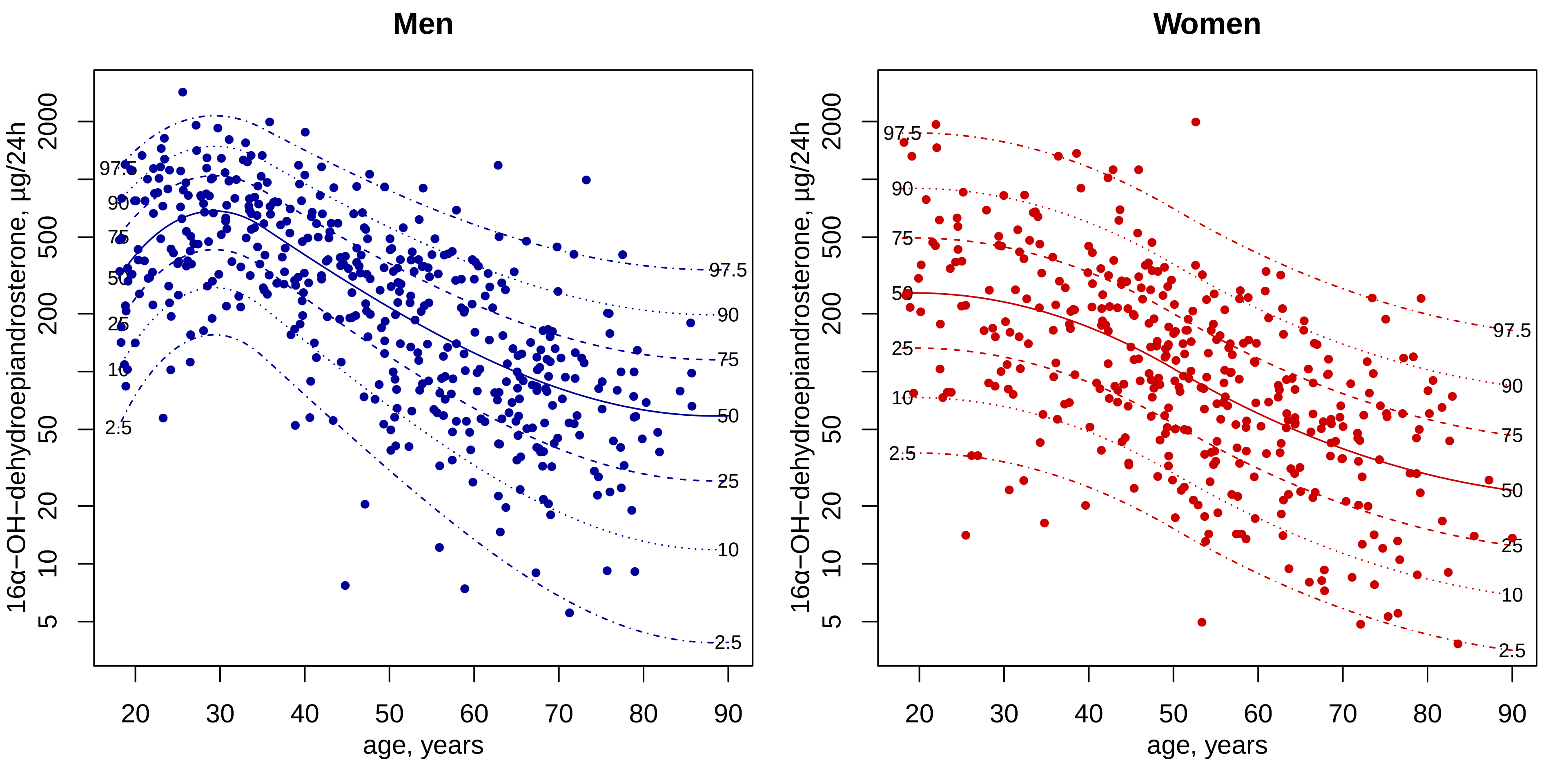

Men
Women
16α−OH−dehydroepiandrosterone, µg/24h
age, years
97.5
90
75
50
25
10
2.5
2000
500
200
50
20
10
5
20
30
40
50
60
70
80
90

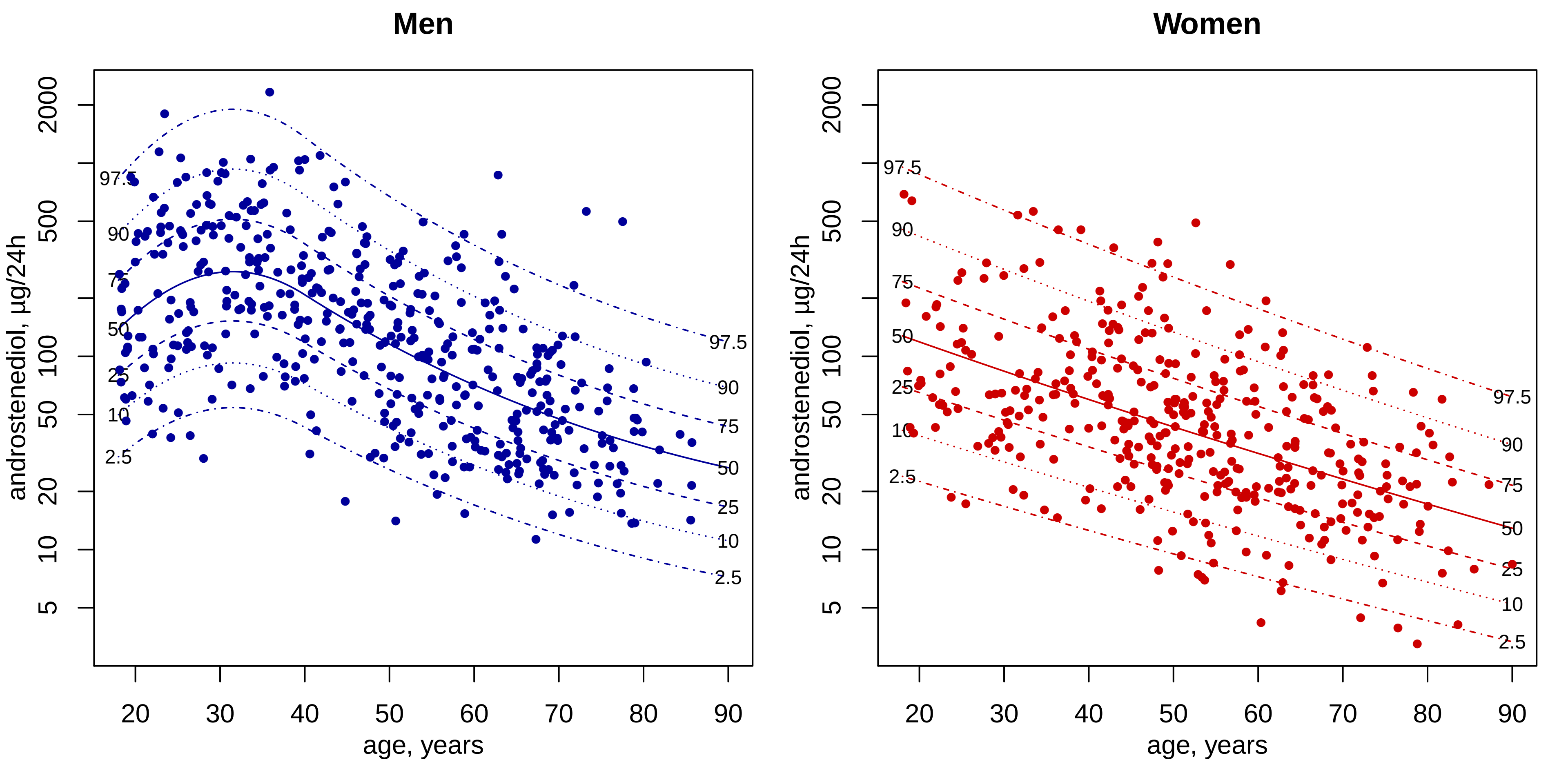

Men
Women
androstenediol, µg/24h
age, years
2000
500
100
50
20
10
5
20
30
40
50
60
70
80
90
97.5
90
75
50
25
10
2.5

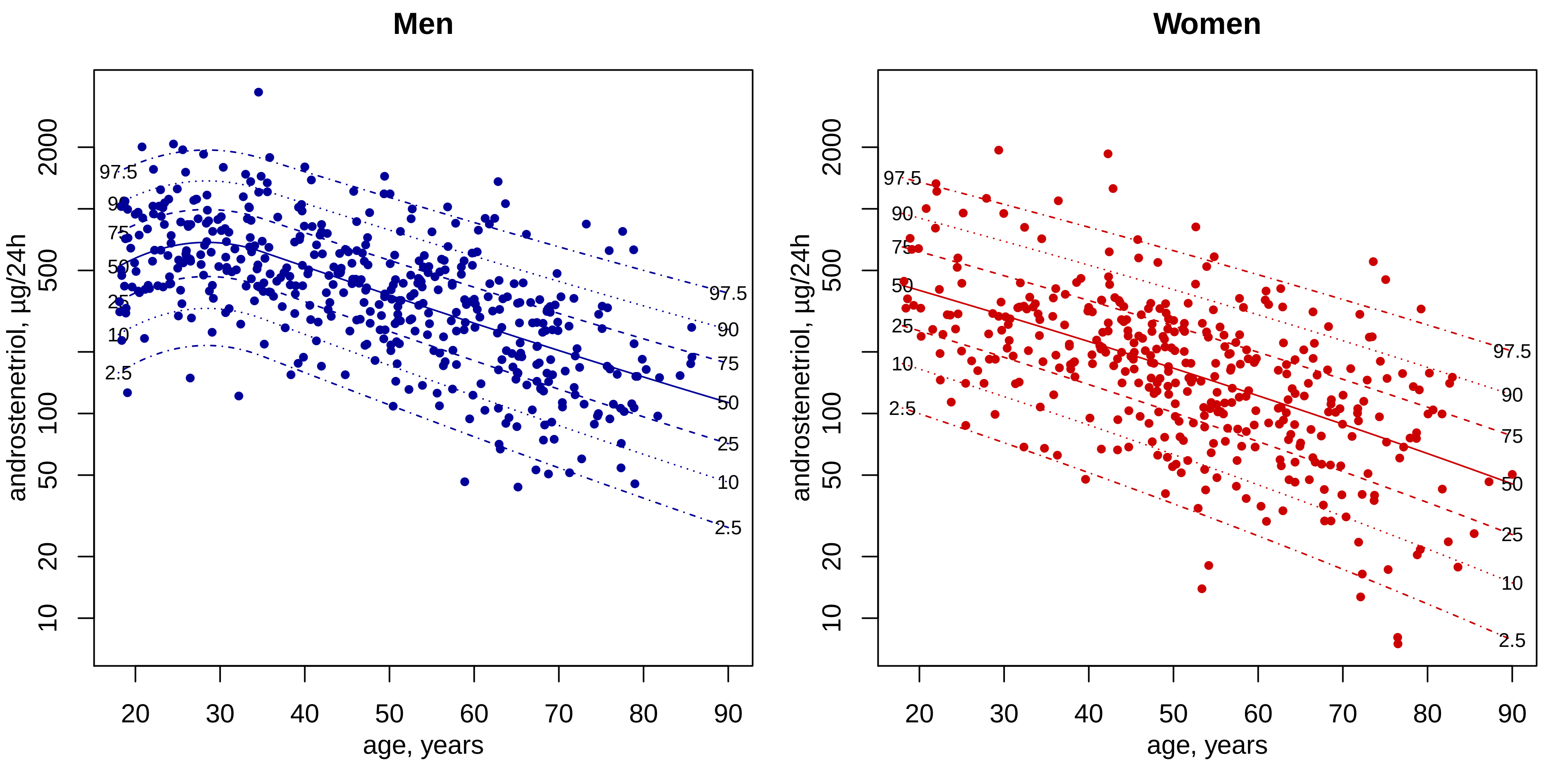
Men
Women
androstenetriol, µg/24h
age, years
2000
500
100
50
20
10
20
30
40
50
60
70
80
90
97.5
90
75
50
25
10
2.5

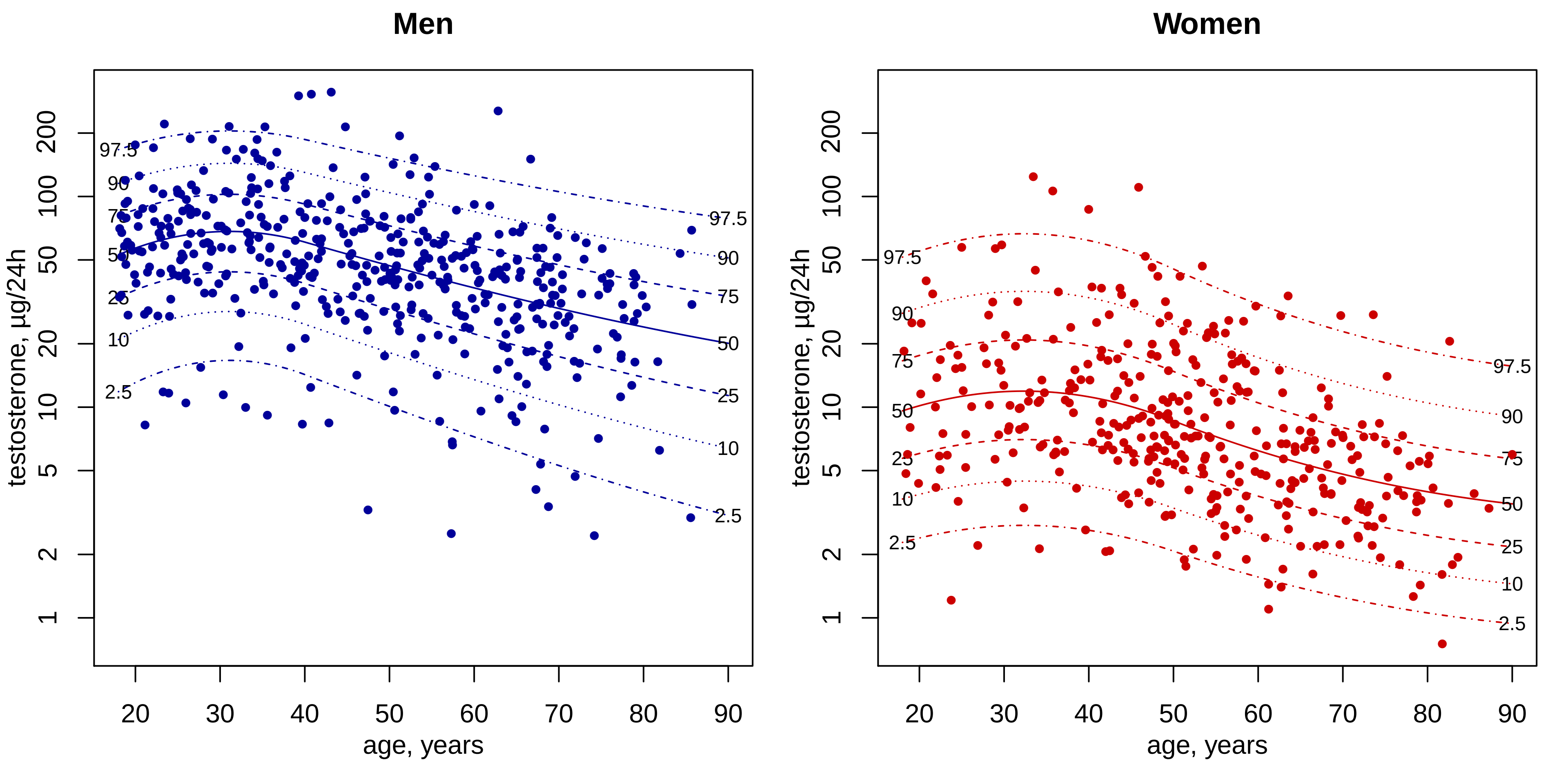
Men
Women
testosterone, µg/24h
age, years
1
2
5
10
20
50
100
200
20
30
40
50
60
70
80
90
97.5
90
75
50
25
10
2.5

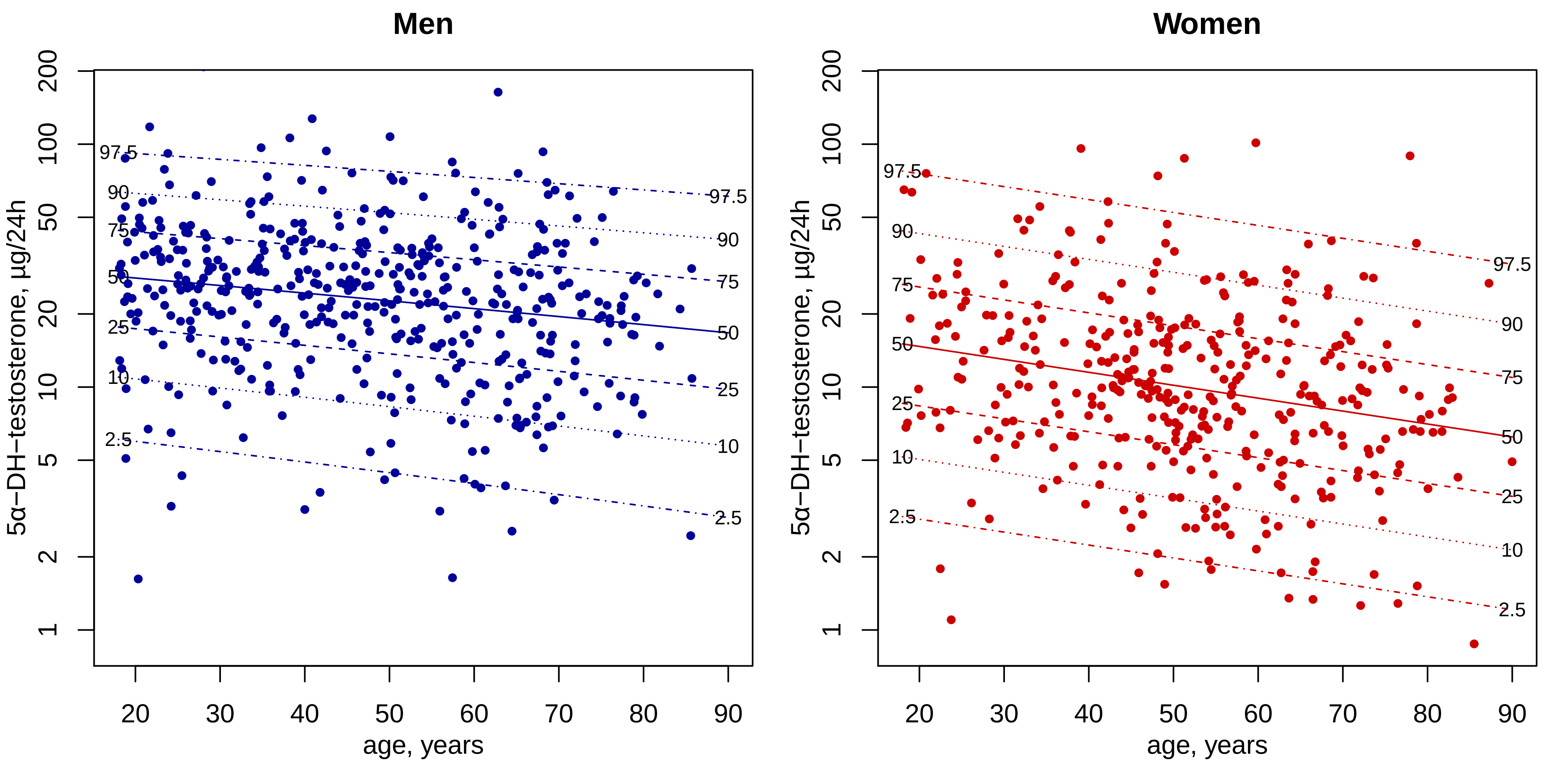
Men
Women
5α−DH−testosterone, µg/24h
age, years
1
2
5
10
20
50
100
200
20
30
40
50
60
70
80
90
97.5
90
75
50
25
10
2.5

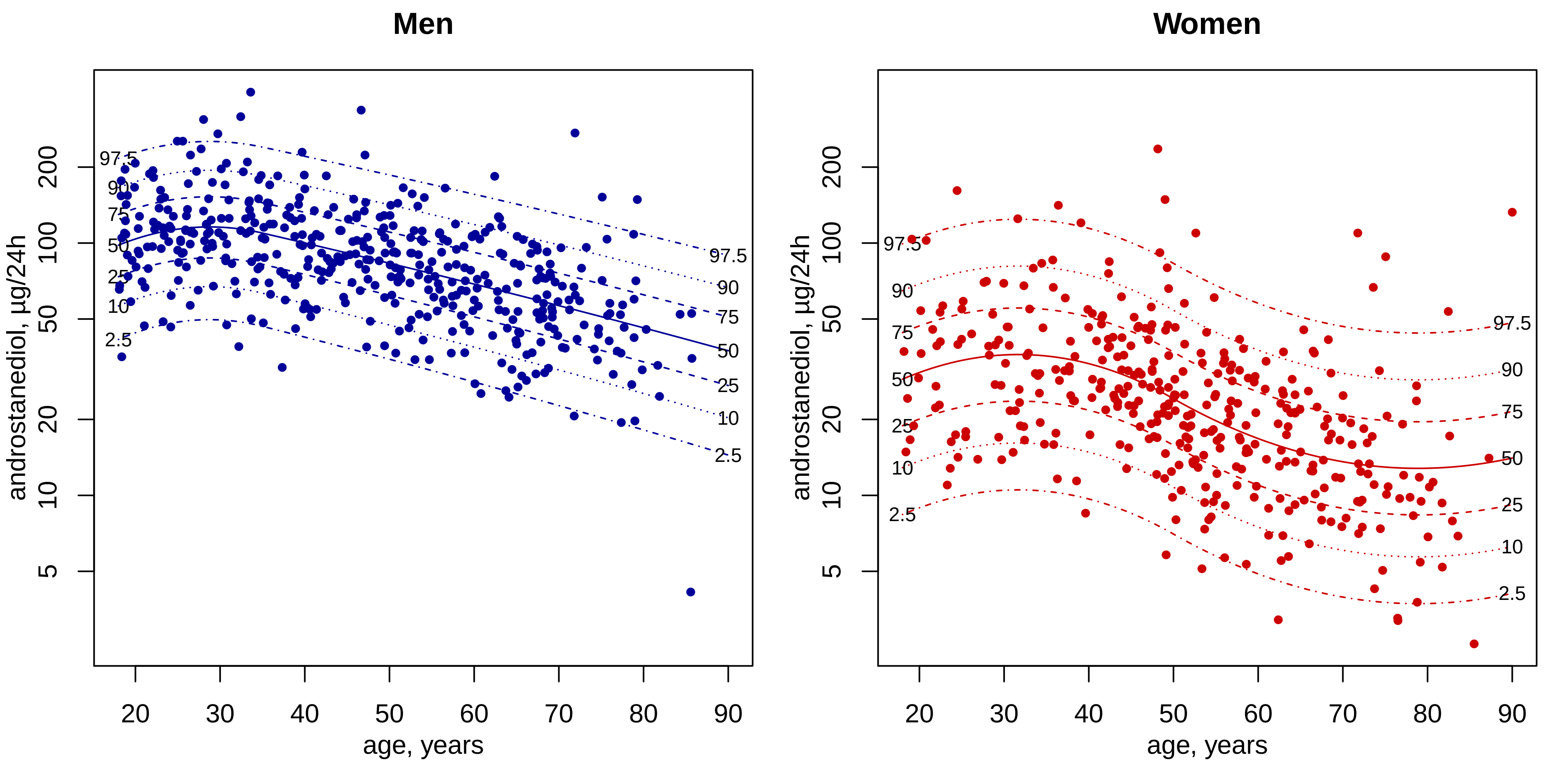
Men
Women
androstanediol, µg/24h
age, years
97.5
90
75
50
25
10
2.5
5
10
20
50
100
200
20
30
40
50
60
70
80
90

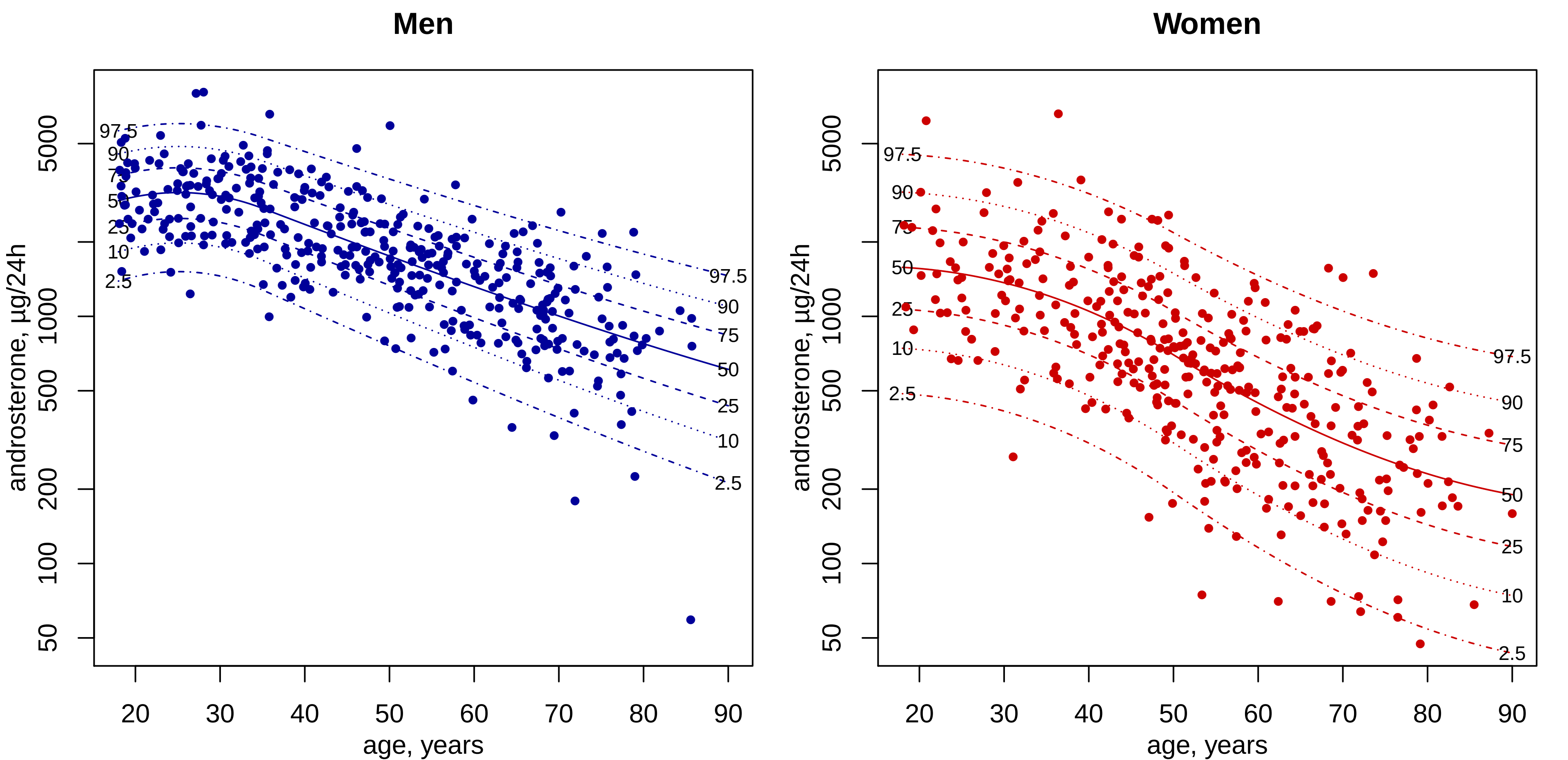

Men
Women
androsterone, µg/24h
age, years
5000
1000
500
200
100
50
20
30
40
50
60
70
80
90
97.5
90
75
50
25
10
2.5

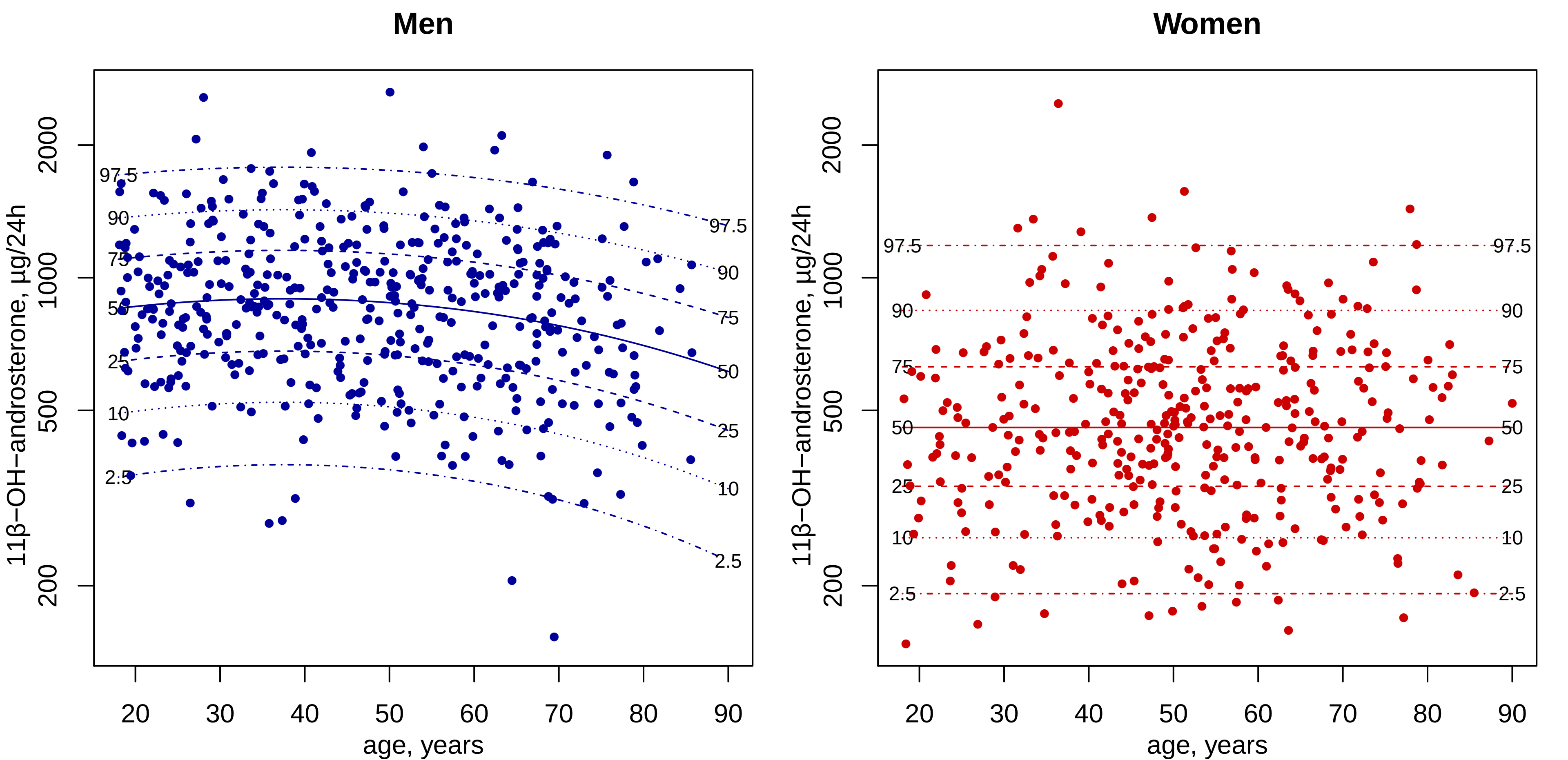

Men
Women
11β−OH−androsterone, µg/24h
2000
1000
500
200
97.5
90
75
50
25
10
2.5
20
30
40
50
60
70
80
90
age, years

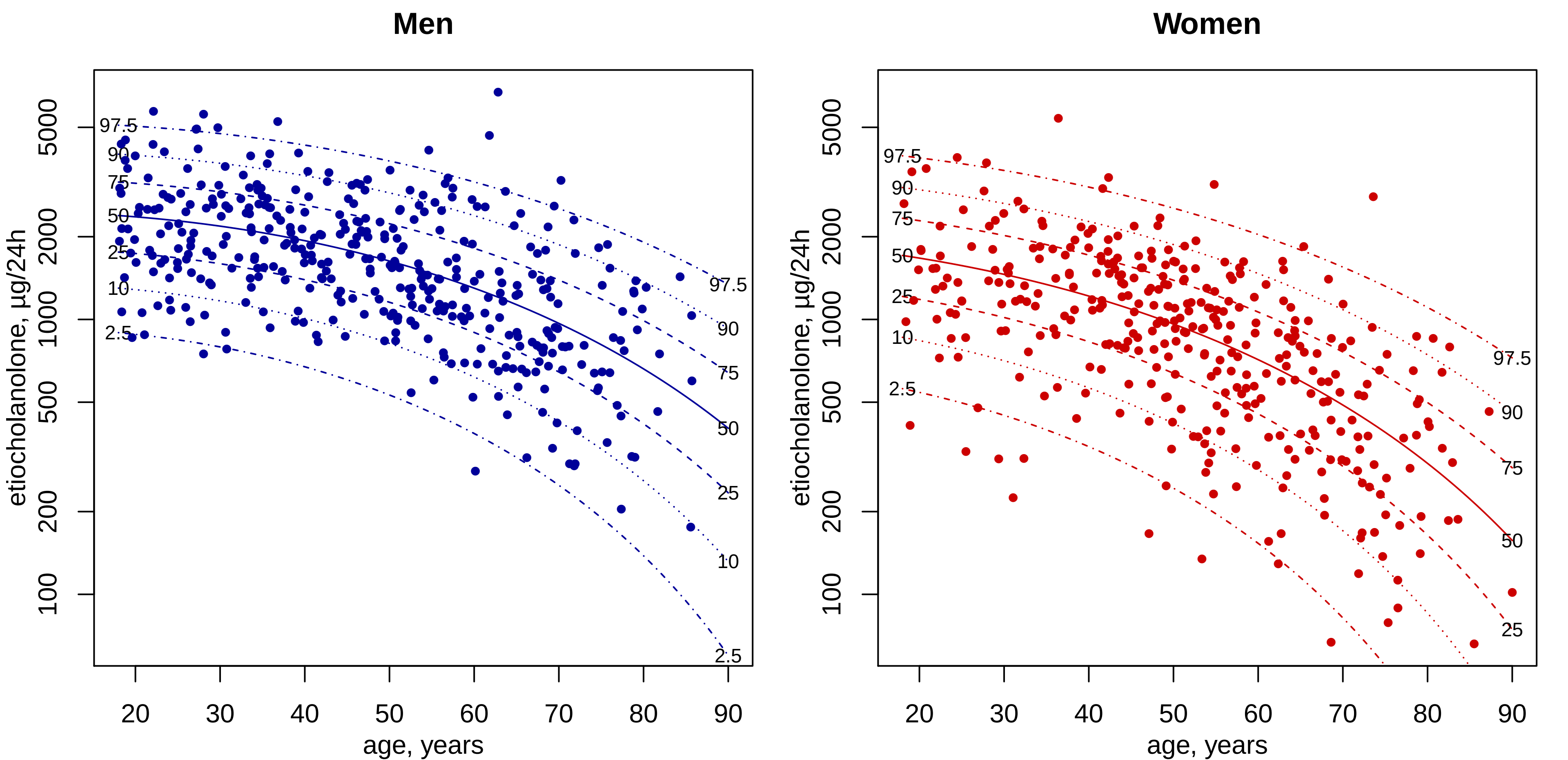

Men
Women
97.5
90
75
50
25
10
2.5
etiocholanolone, µg/24h
age, years
5000
2000
1000
500
200
100
20
30
40
50
60
70
80
90

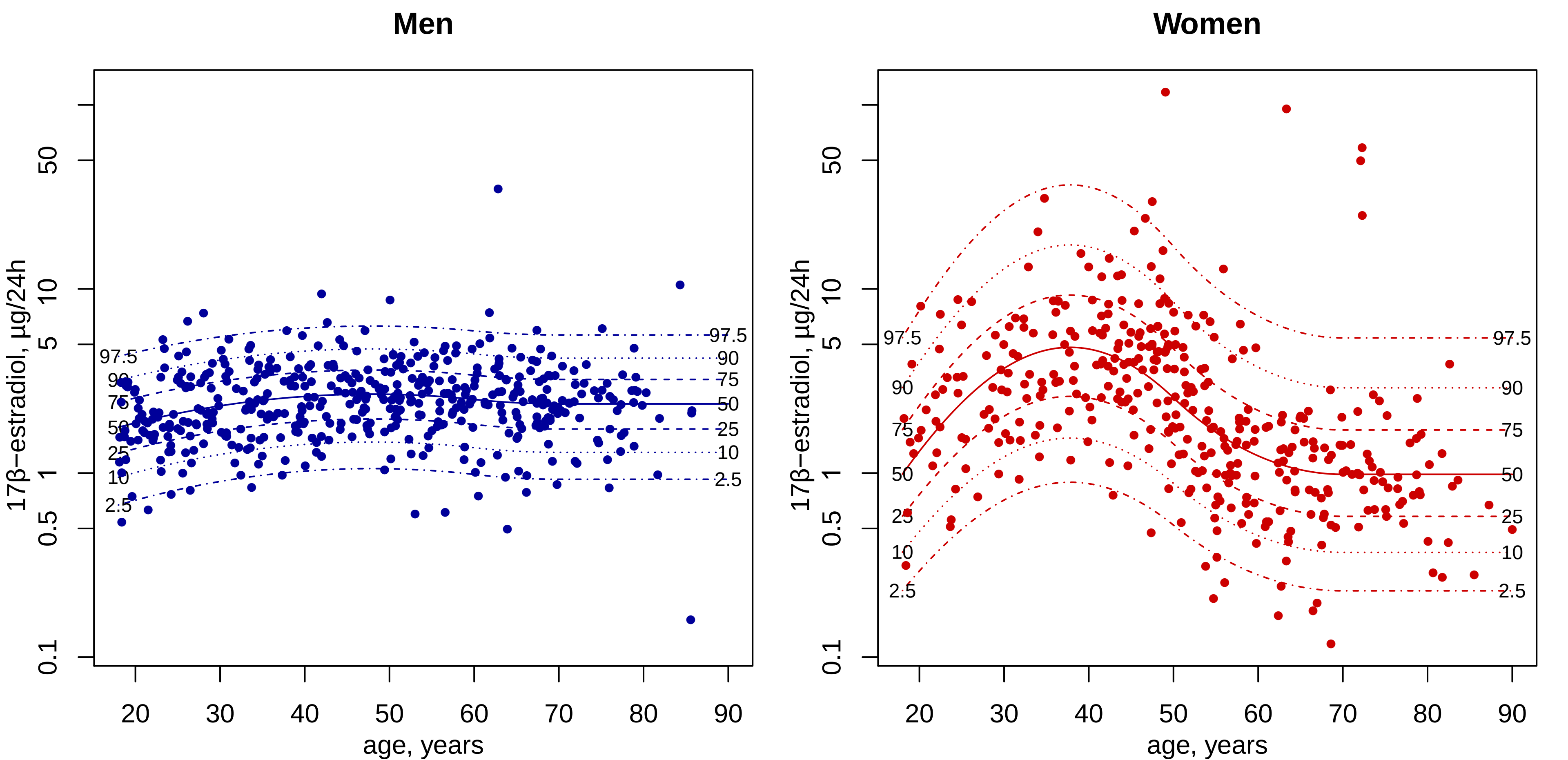

Men
Women
17β−estradiol, μg/24h
age, years
0.1
0.5
1
5
10
50
20
30
40
50
60
70
80
90
97.5
90
75
50
25
10
2.5

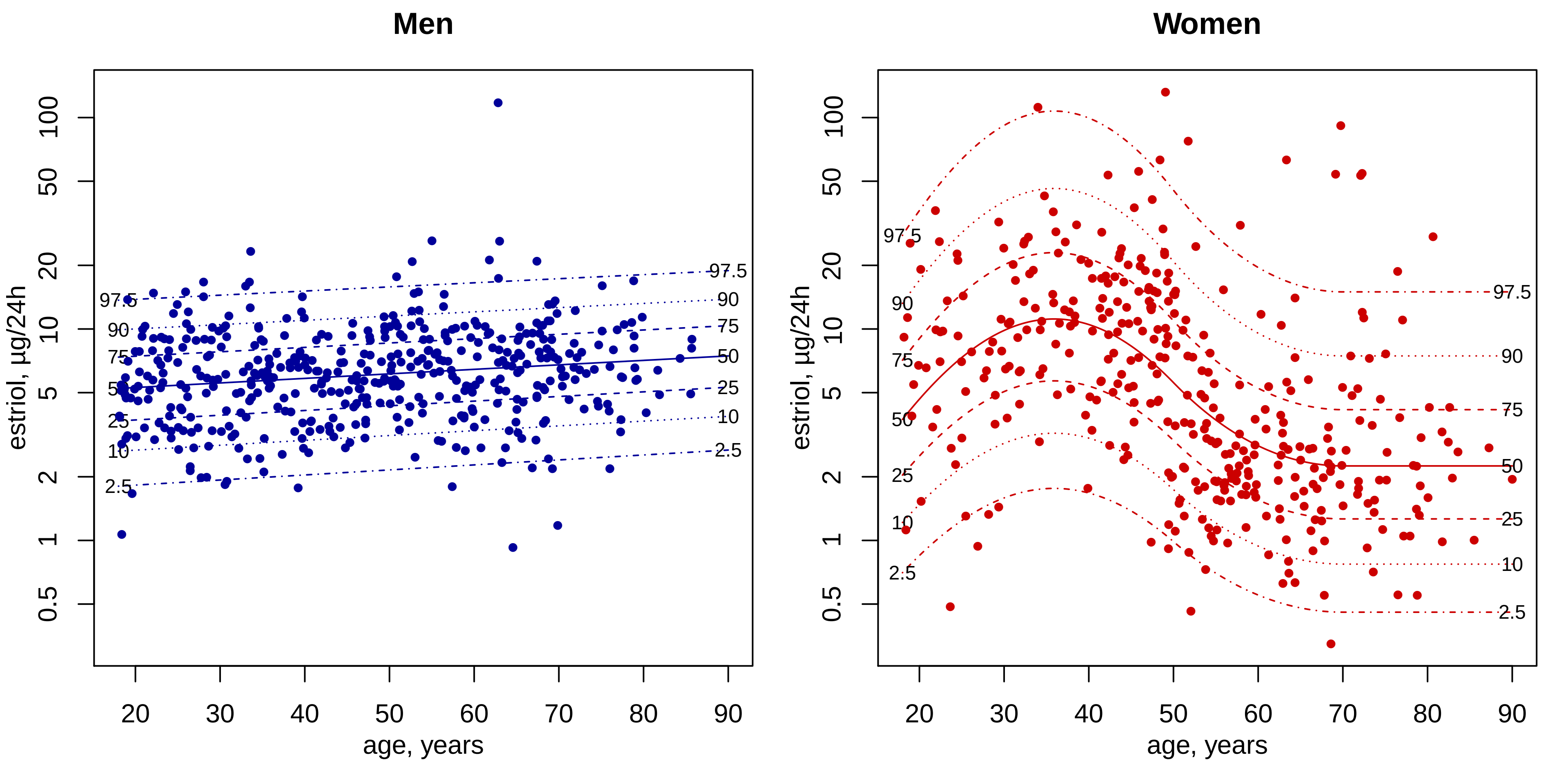

Men
Women
estriol, µg/24h
age, years
0.5
1
2
5
10
20
50
100
20
30
40
50
60
70
80
90
97.5
90
75
50
25
10
2.5

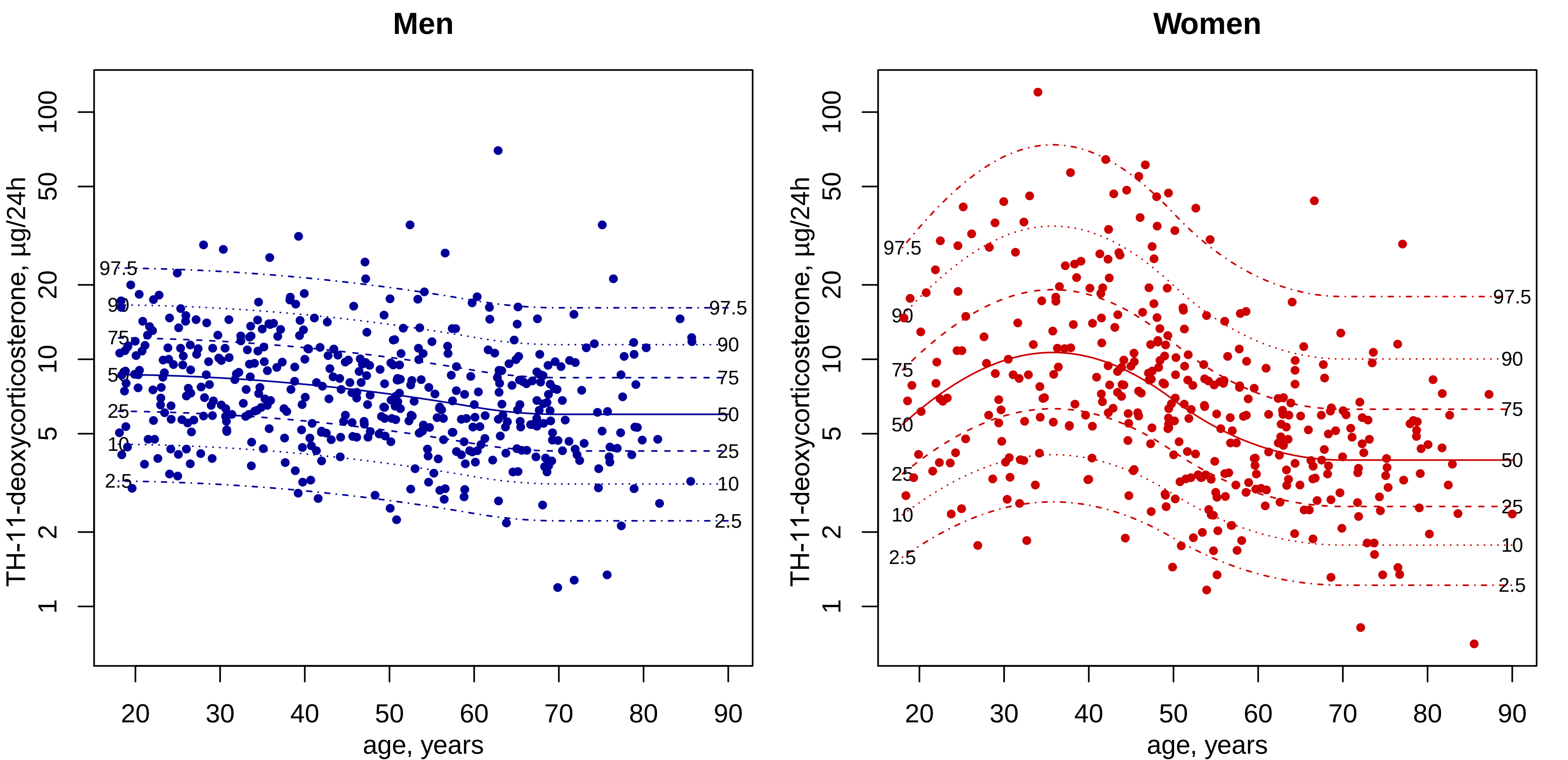

Men
Women
TH-11-deoxycorticosterone, µg/24h
age, years
97.5
90
75
50
25
10
2.5

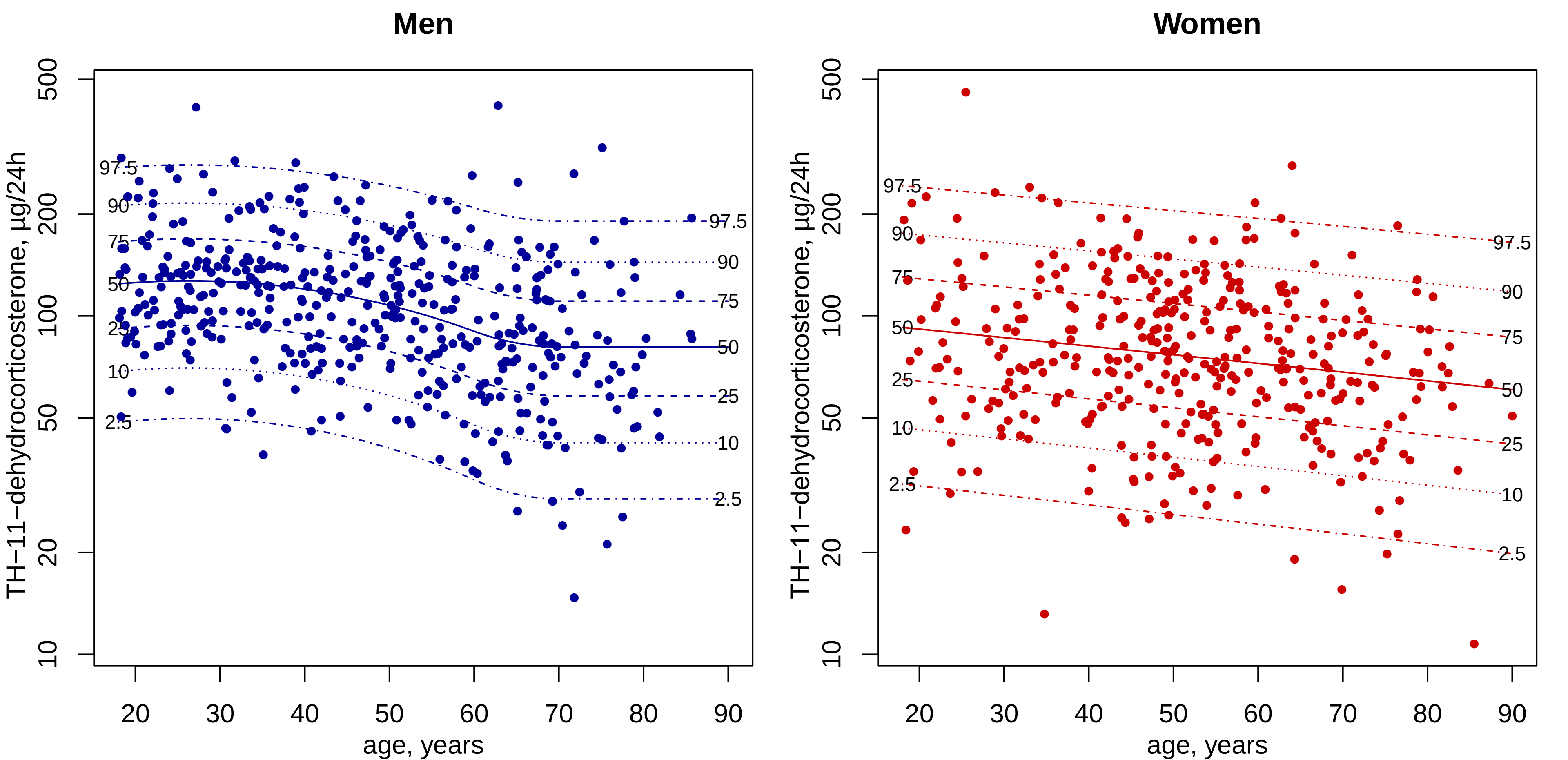

Men
Women
TH−11−dehydrocorticosterone, µg/24h
age, years
97.5
90
75
50
25
10
2.5
500
200
100
50
20
10
20
30
40
50
60
70
80
90

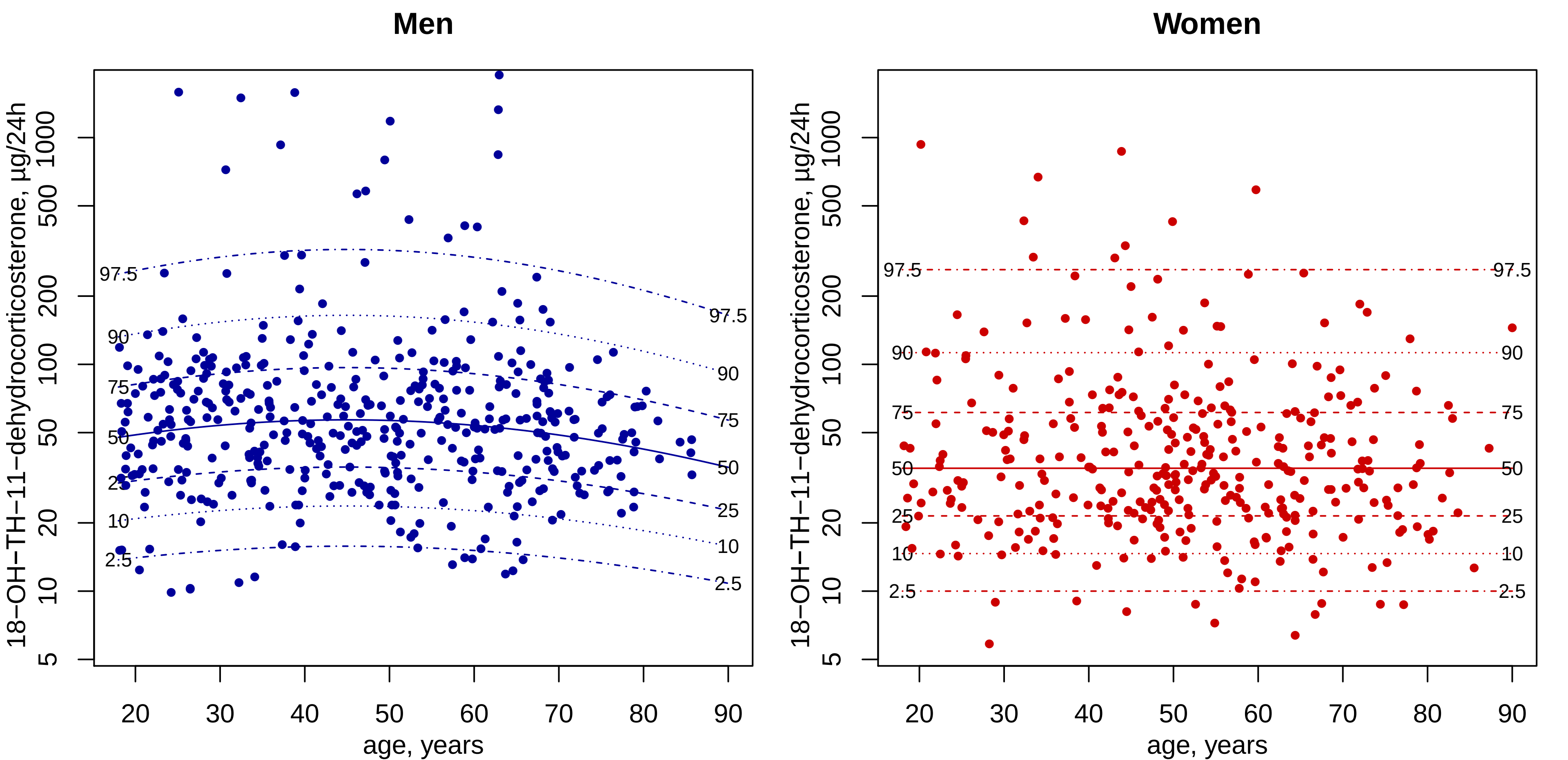

Men
Women
18−OH−TH−11−dehydrocorticosterone, µg/24h
5
10
20
50
100
200
500
1000
97.5
90
75
50
25
10
2.5
20
30
40
50
60
70
80
90
age, years

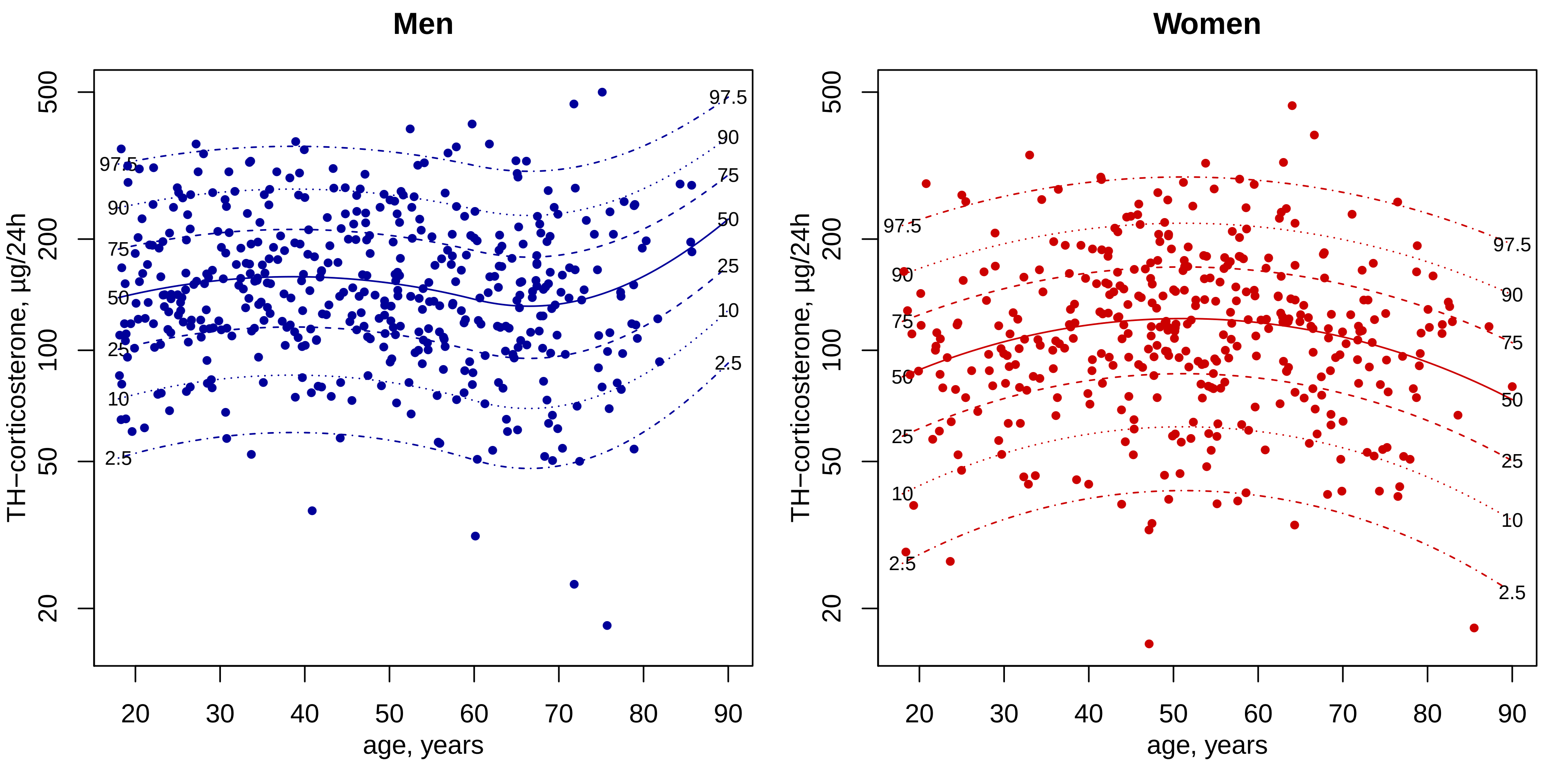

Men
Women
TH−corticosterone, µg/24h
age, years
500
200
100
50
20
20
30
40
50
60
70
80
90
97.5
90
75
50
25
10
2.5

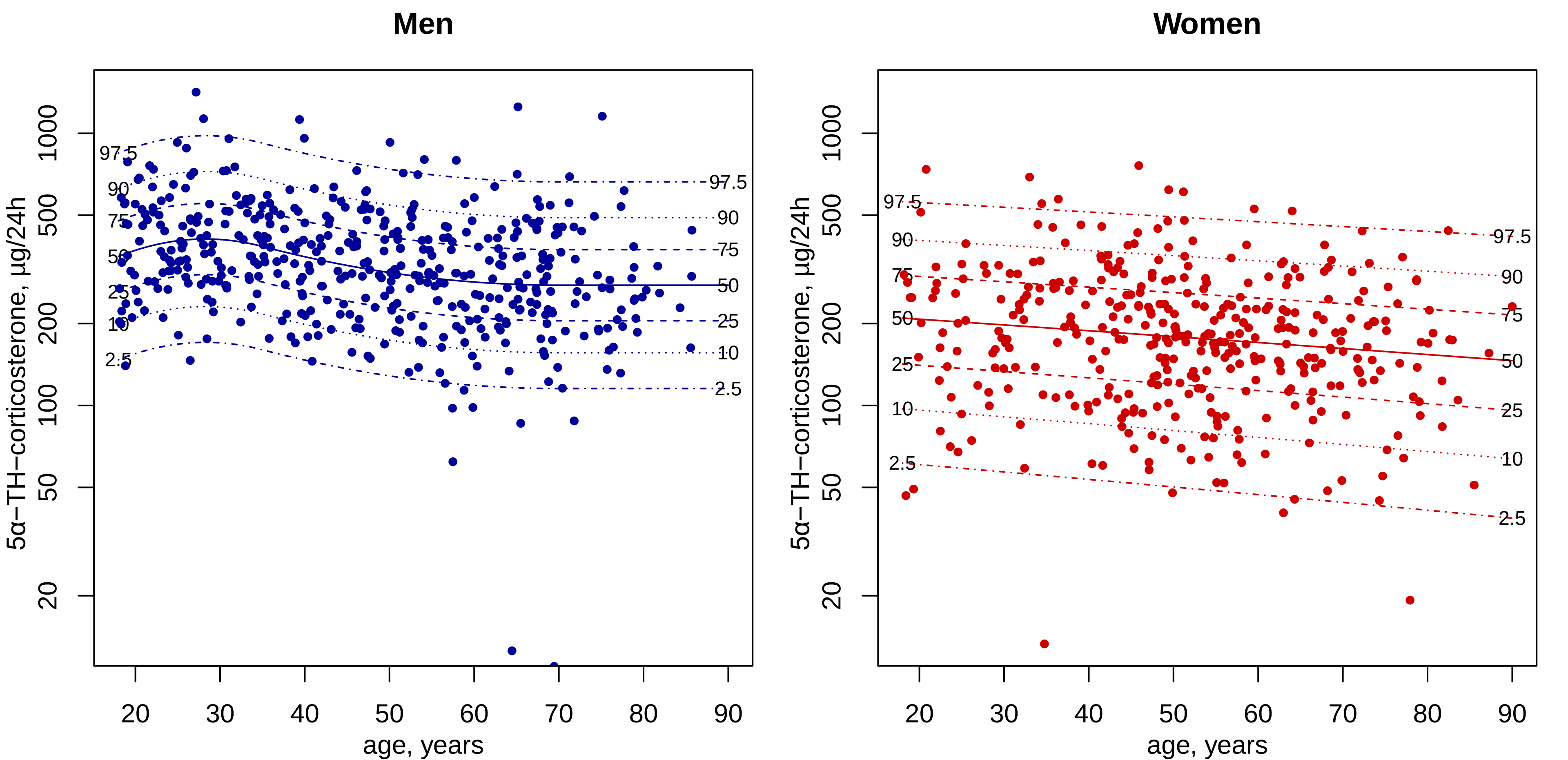

Men
Women
5α−TH−corticosterone, µg/24h
age, years
20
50
100
200
500
1000
20 30 40 50 60 70 80 90
97.5
90
75
50
25
10
2.5

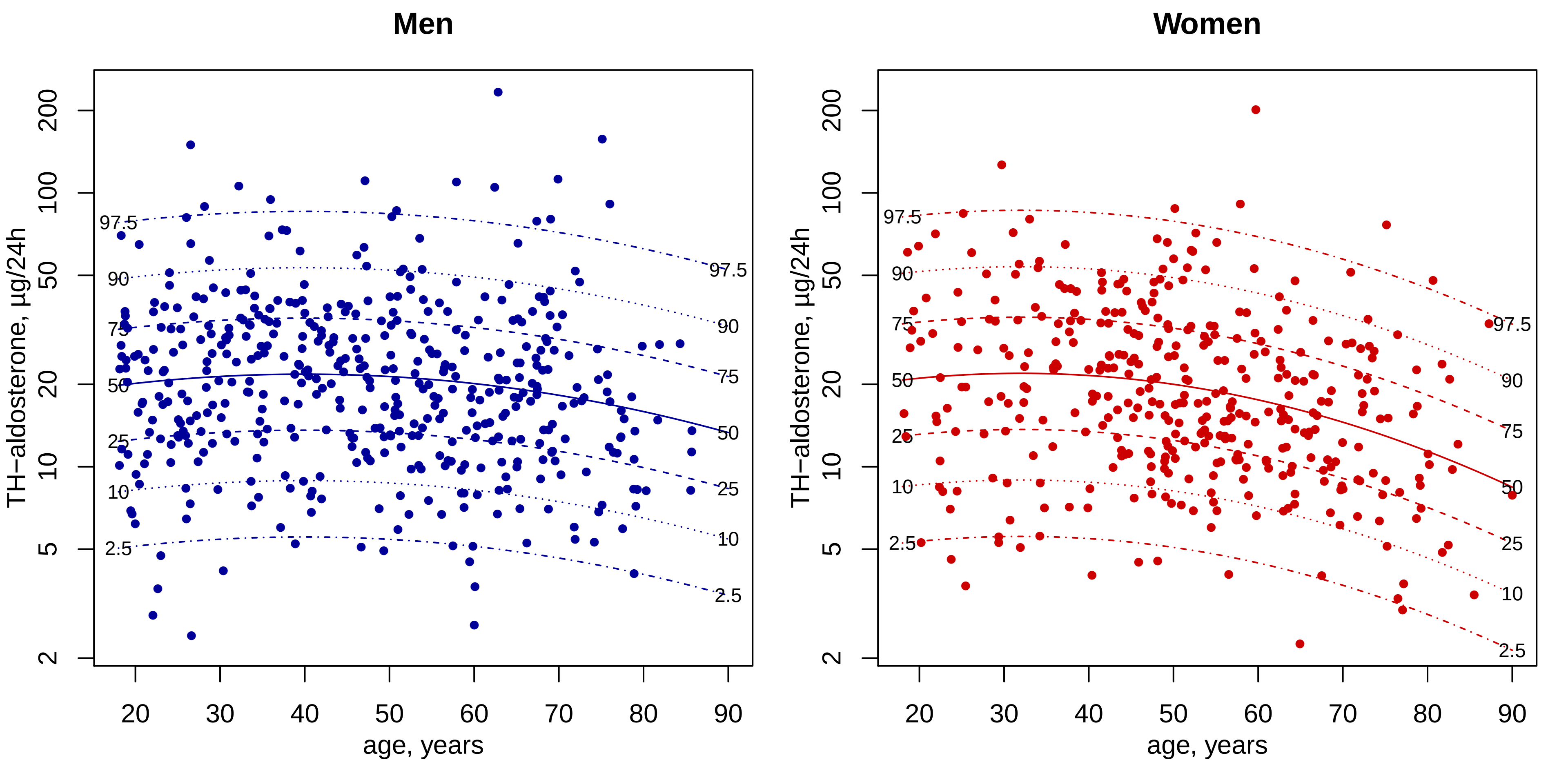

Men
Women
TH−aldosterone, µg/24h
age, years
200
100
50
20
10
5
2
20
30
40
50
60
70
80
90
97.5
90
75
50
25
10
2.5

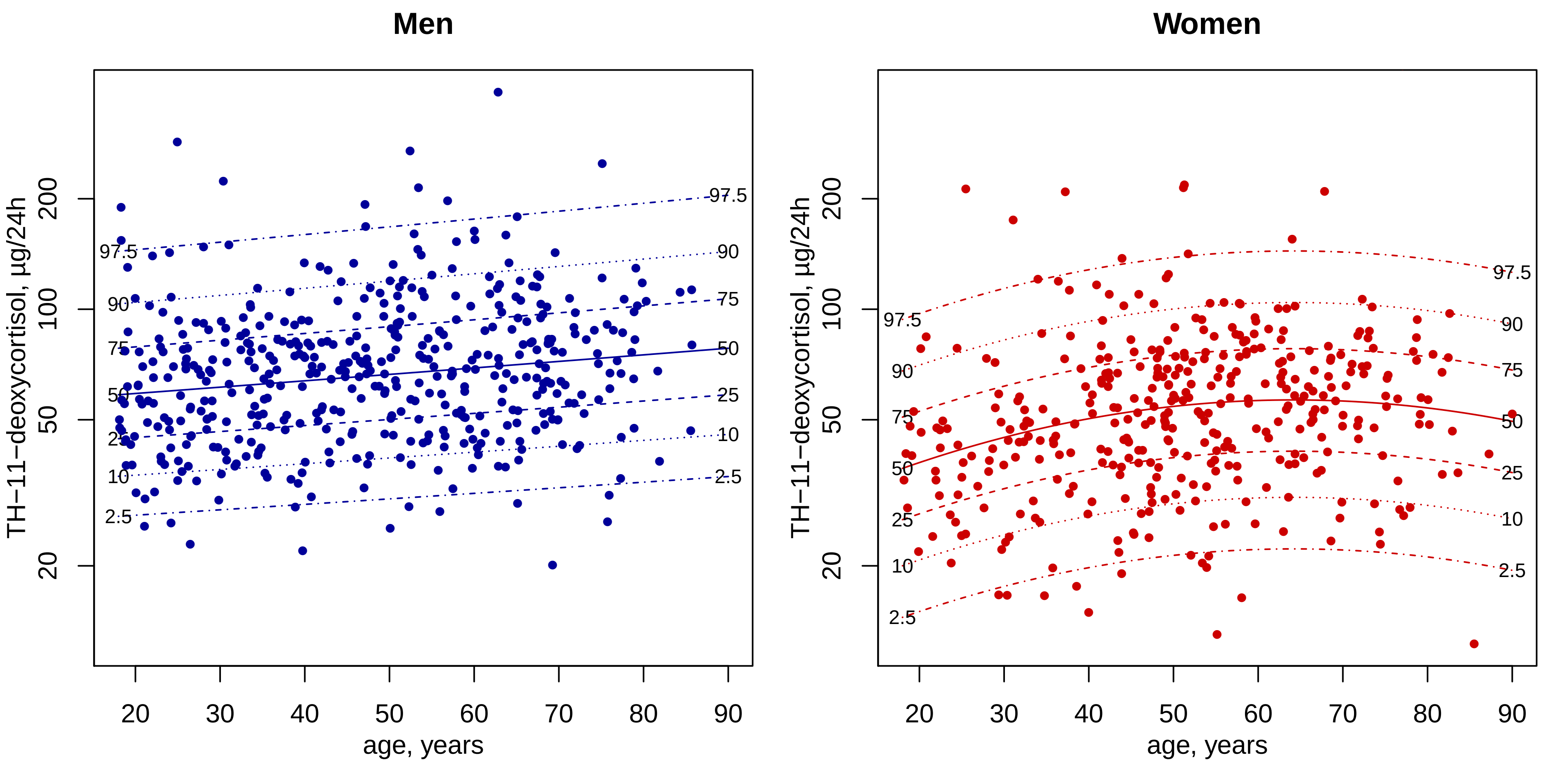

Men
Women
TH−11−deoxycortisol, µg/24h
200
100
50
20
97.5
90
75
50
25
10
2.5
20
30
40
50
60
70
80
90
age, years

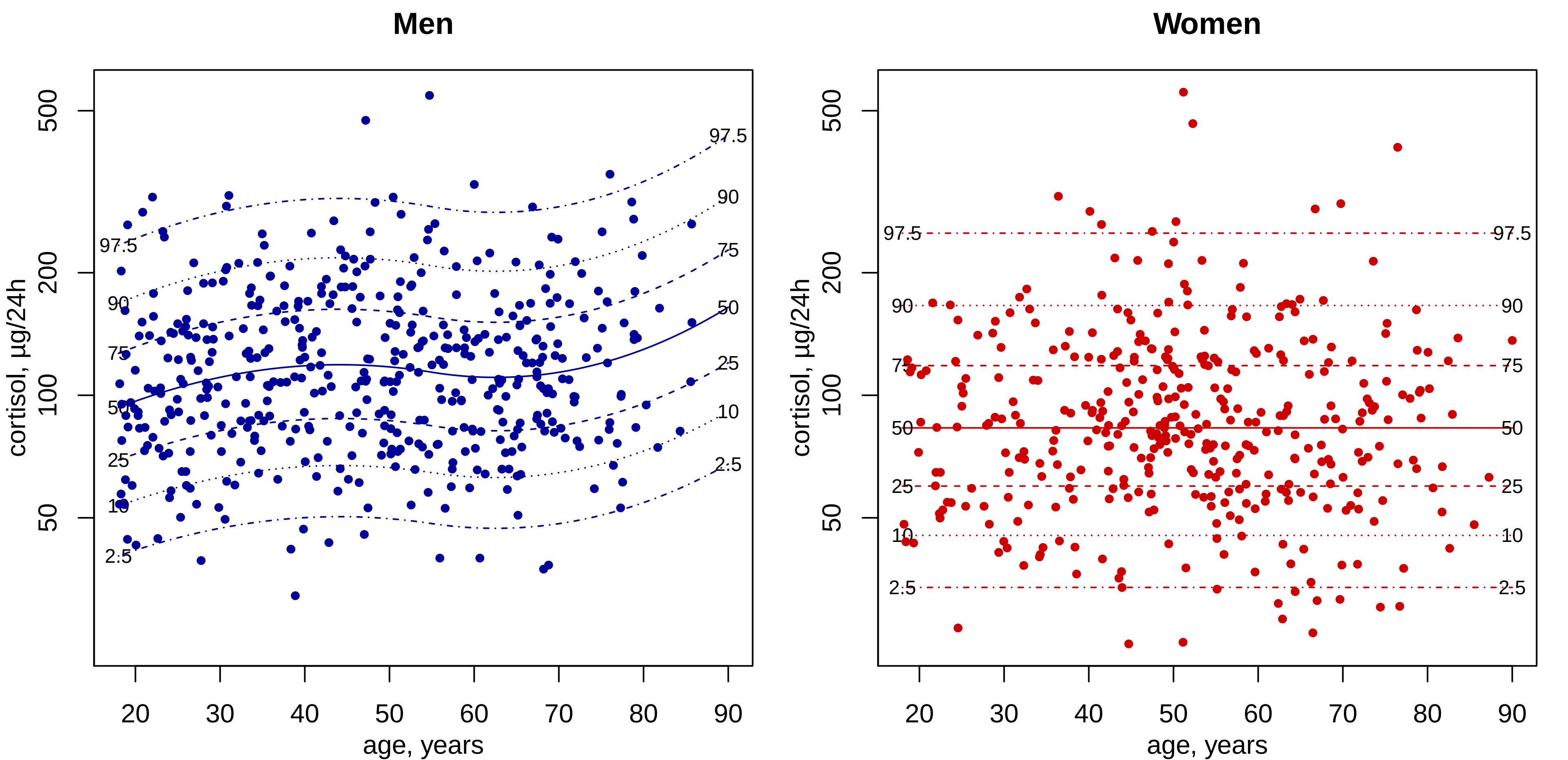

Men
Women
cortisol, µg/24h
age, years
500
200
100
50
20
30
40
50
60
70
80
90
97.5
90
75
50
25
10
2.5

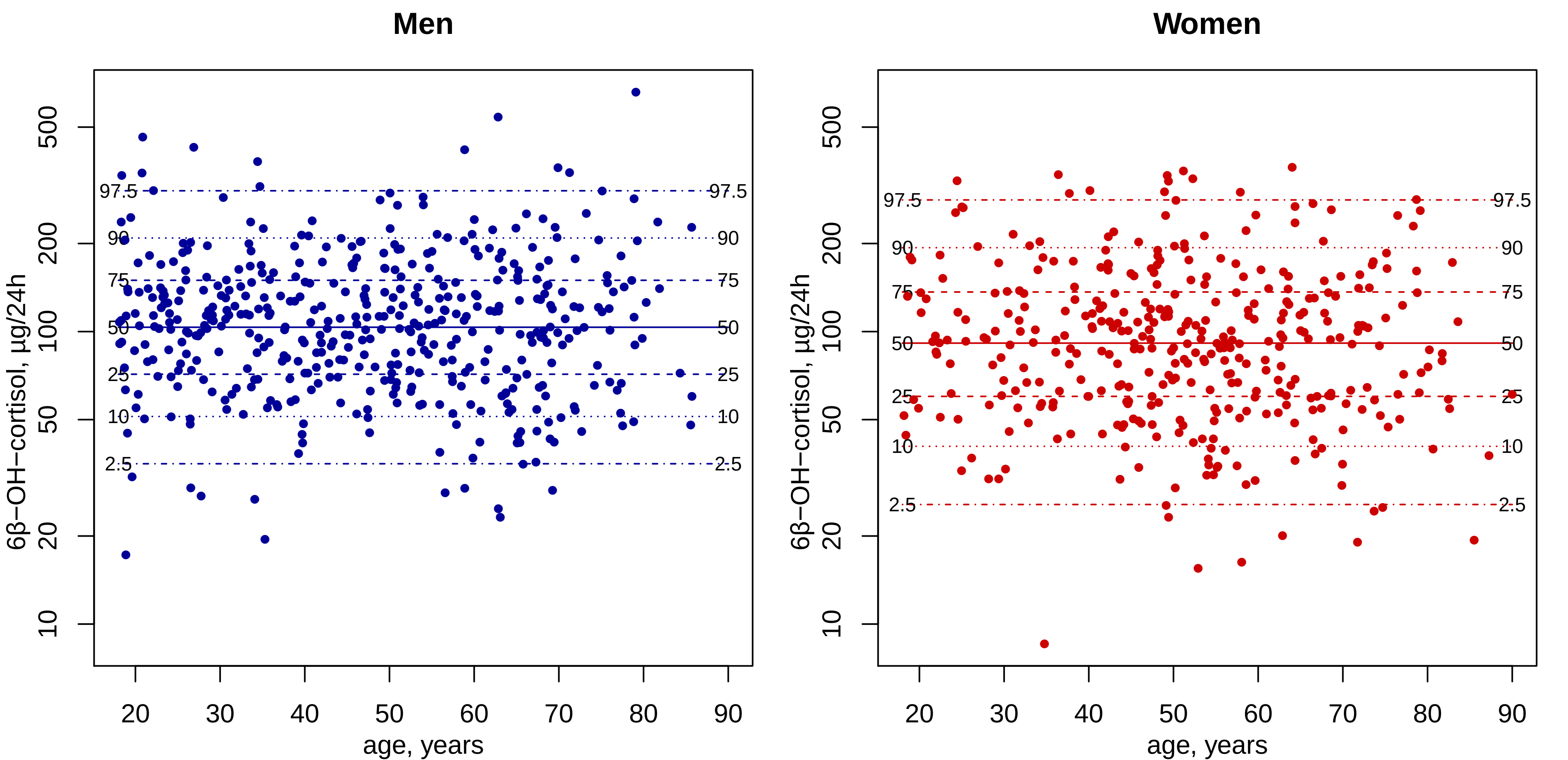
Men
Women
6β−OH−cortisol, µg/24h
age, years
10
20
50
100
200
500
20
30
40
50
60
70
80
90
97.5
90
75
50
25
10
2.5

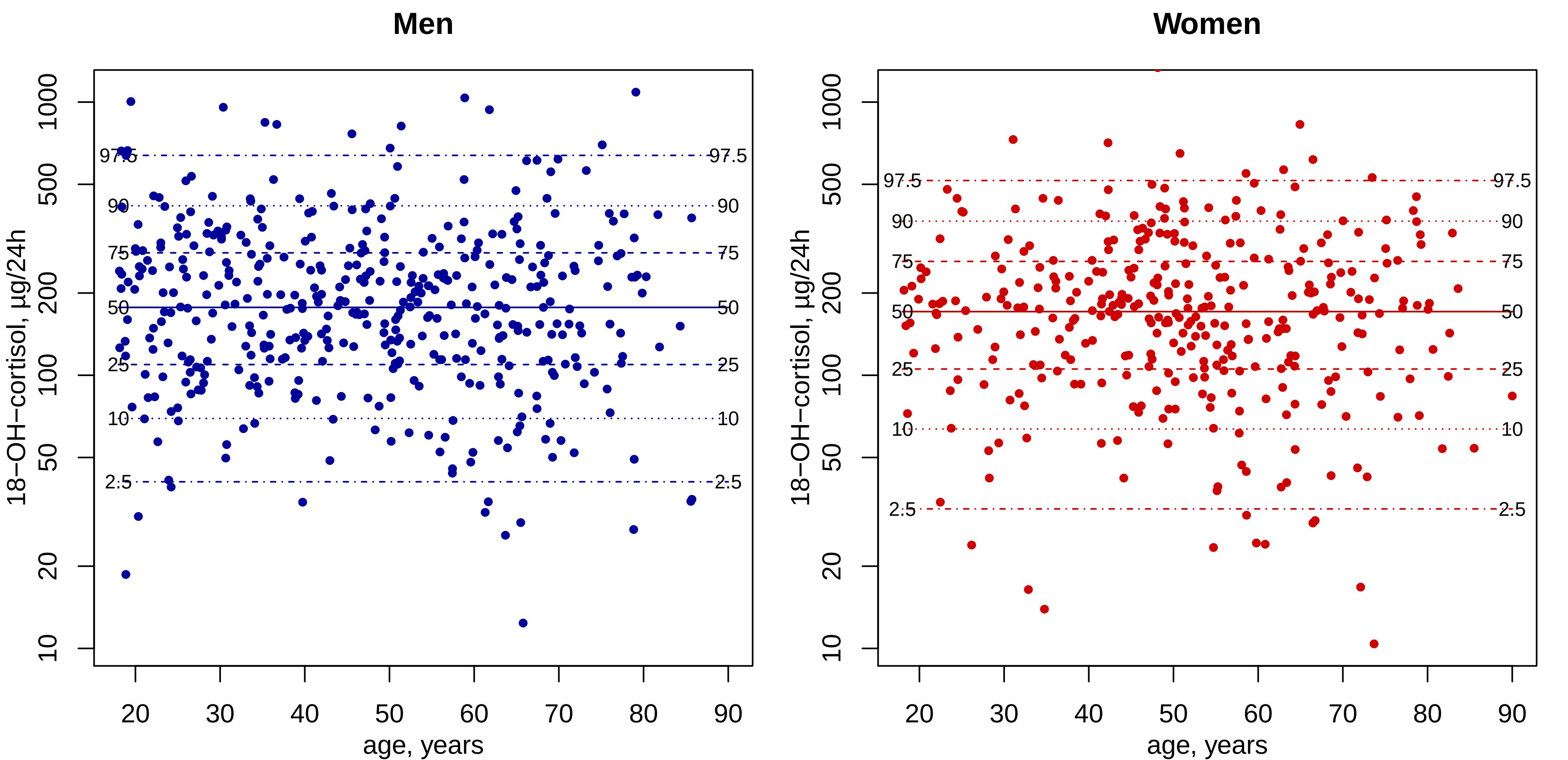
Men
Women
18−OH−cortisol, µg/24h
10
20
50
100
200
500
1000
97.5
90
75
50
25
10
2.5
20
30
40
50
60
70
80
90
age, years

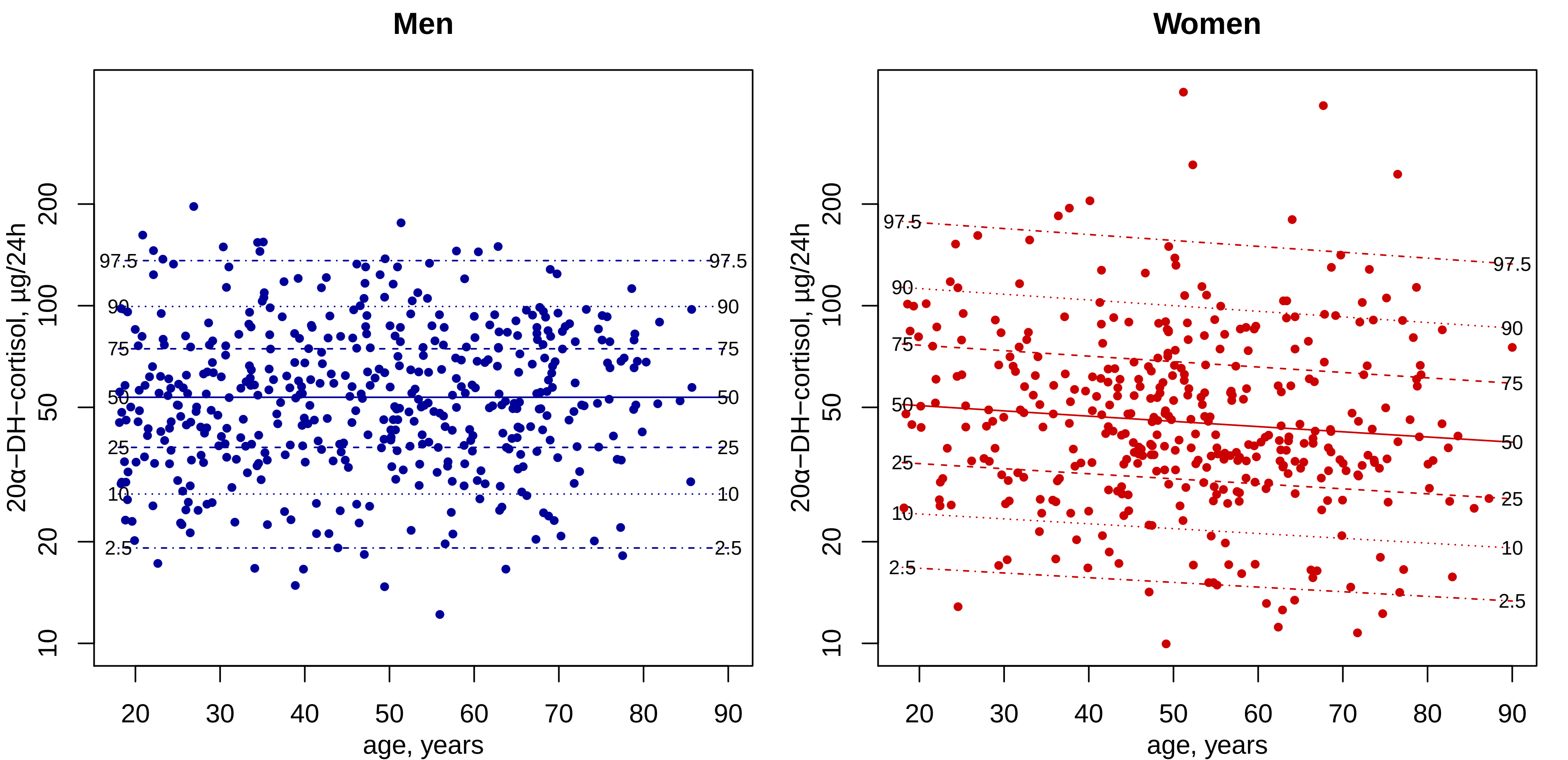

Men
Women
20α−DH−cortisol, µg/24h
age, years
97.5
90
75
50
25
10
2.5
10
20
50
100
200
20
30
40
50
60
70
80
90

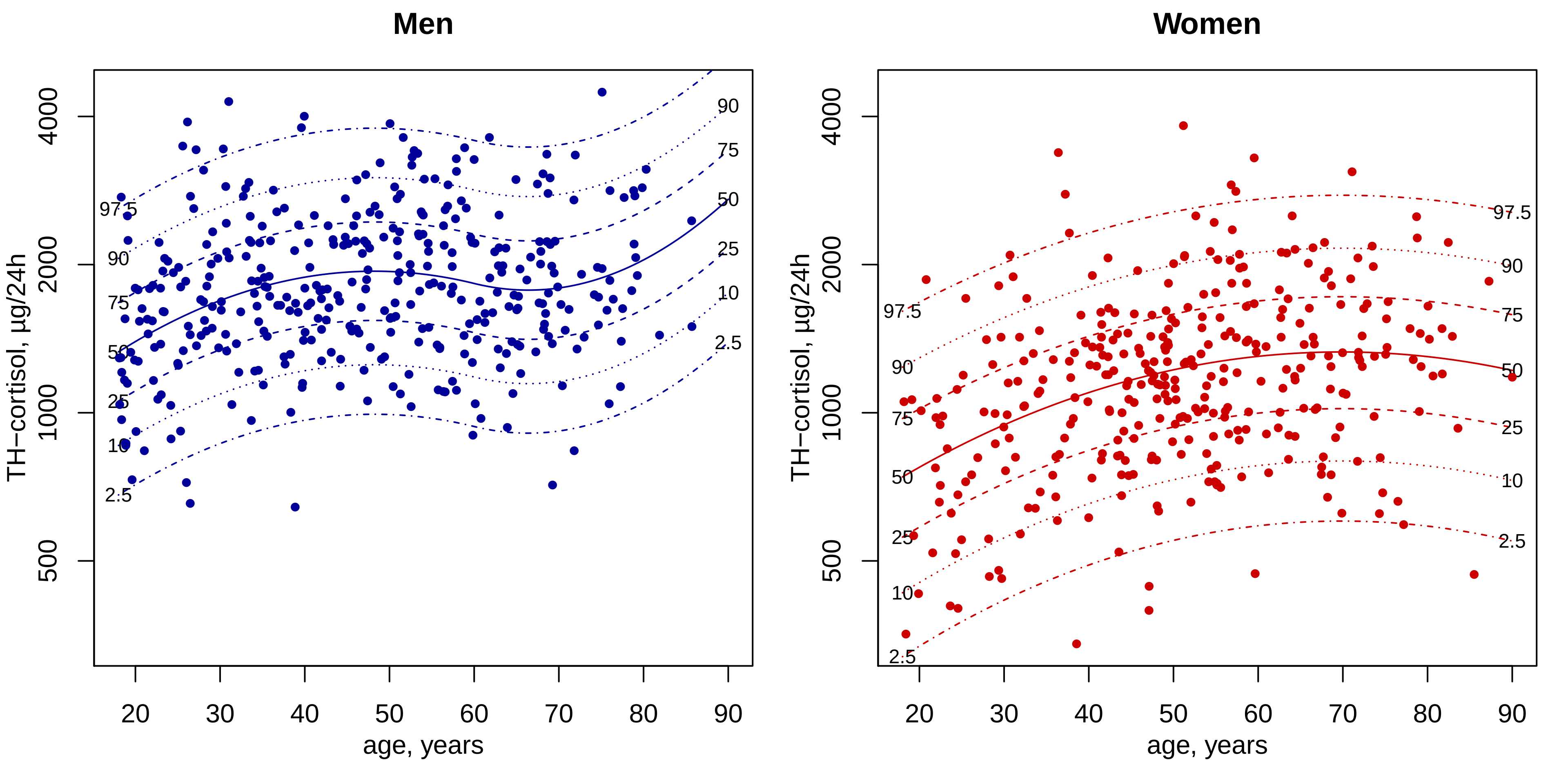

Men
Women
TH−cortisol, µg/24h
4000
2000
1000
500
age, years
20
30
40
50
60
70
80
90
97.5
90
75
50
25
10
2.5

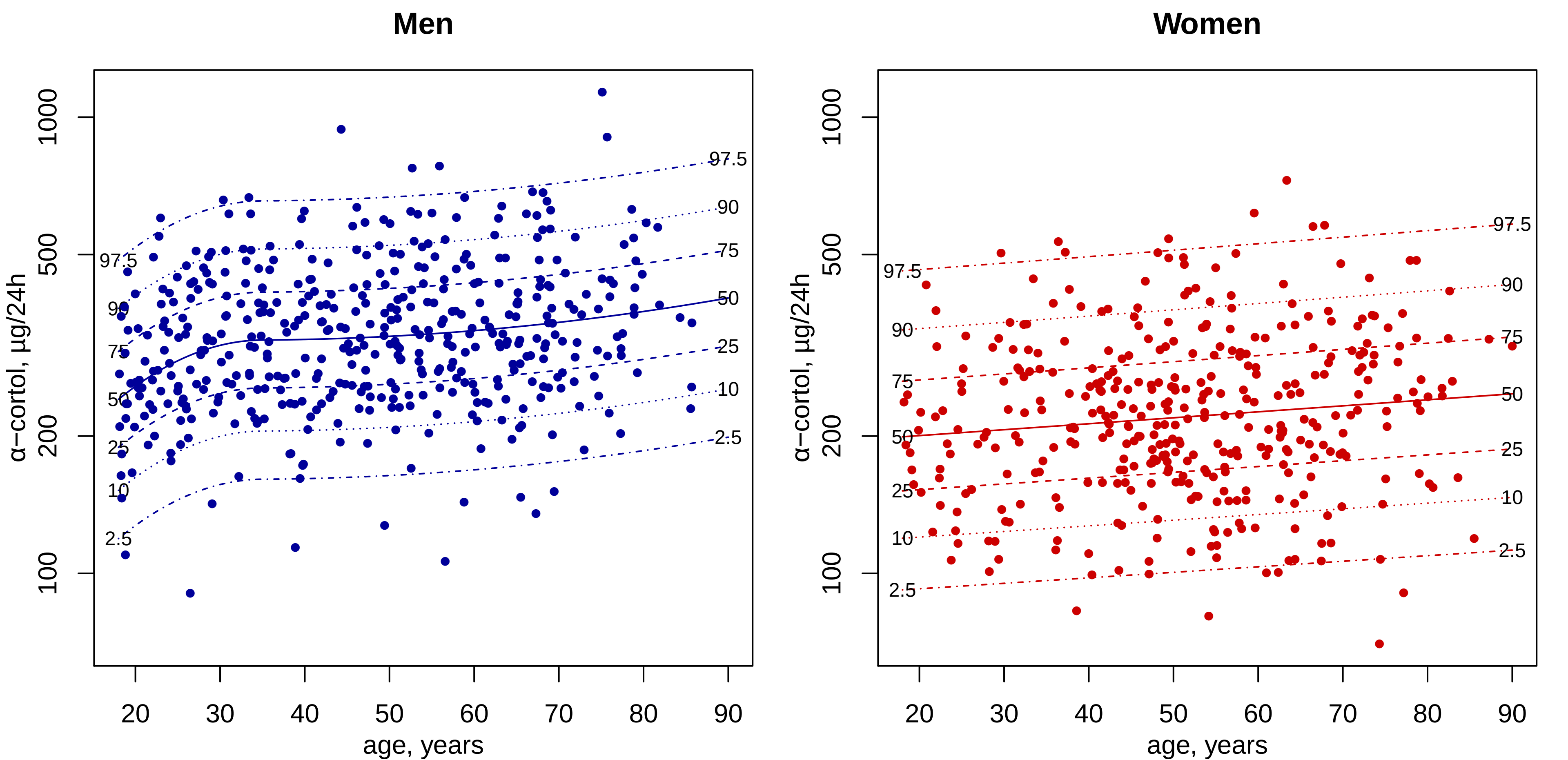

Men
Women
1000
500
200
100
α−cortol, µg/24h
20
30
40
50
60
70
80
90
age, years
97.5
90
75
50
25
10
2.5

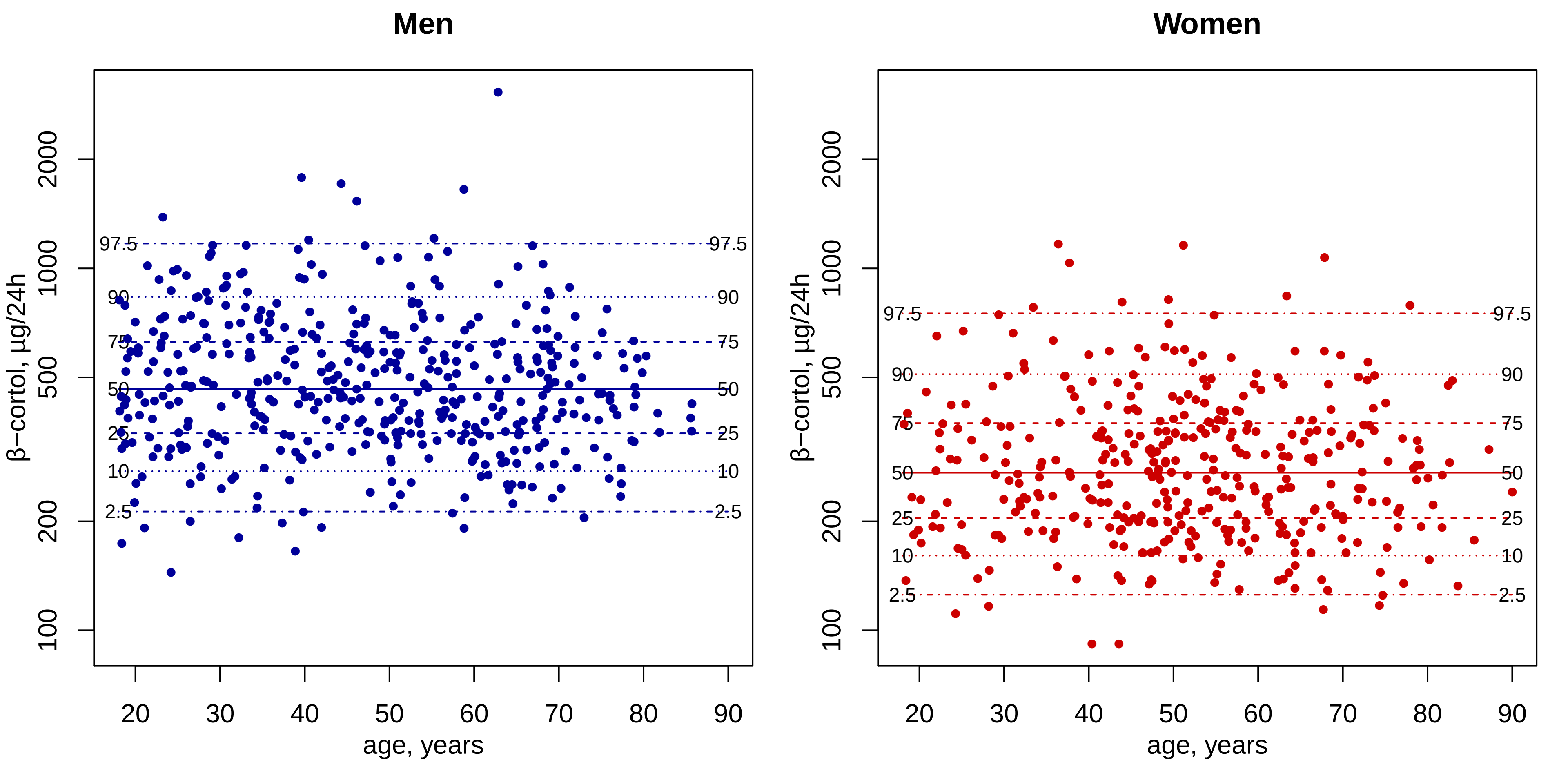

Men
Women
β−cortol, μg/24h
age, years
97.5
90
75
50
25
10
2.5

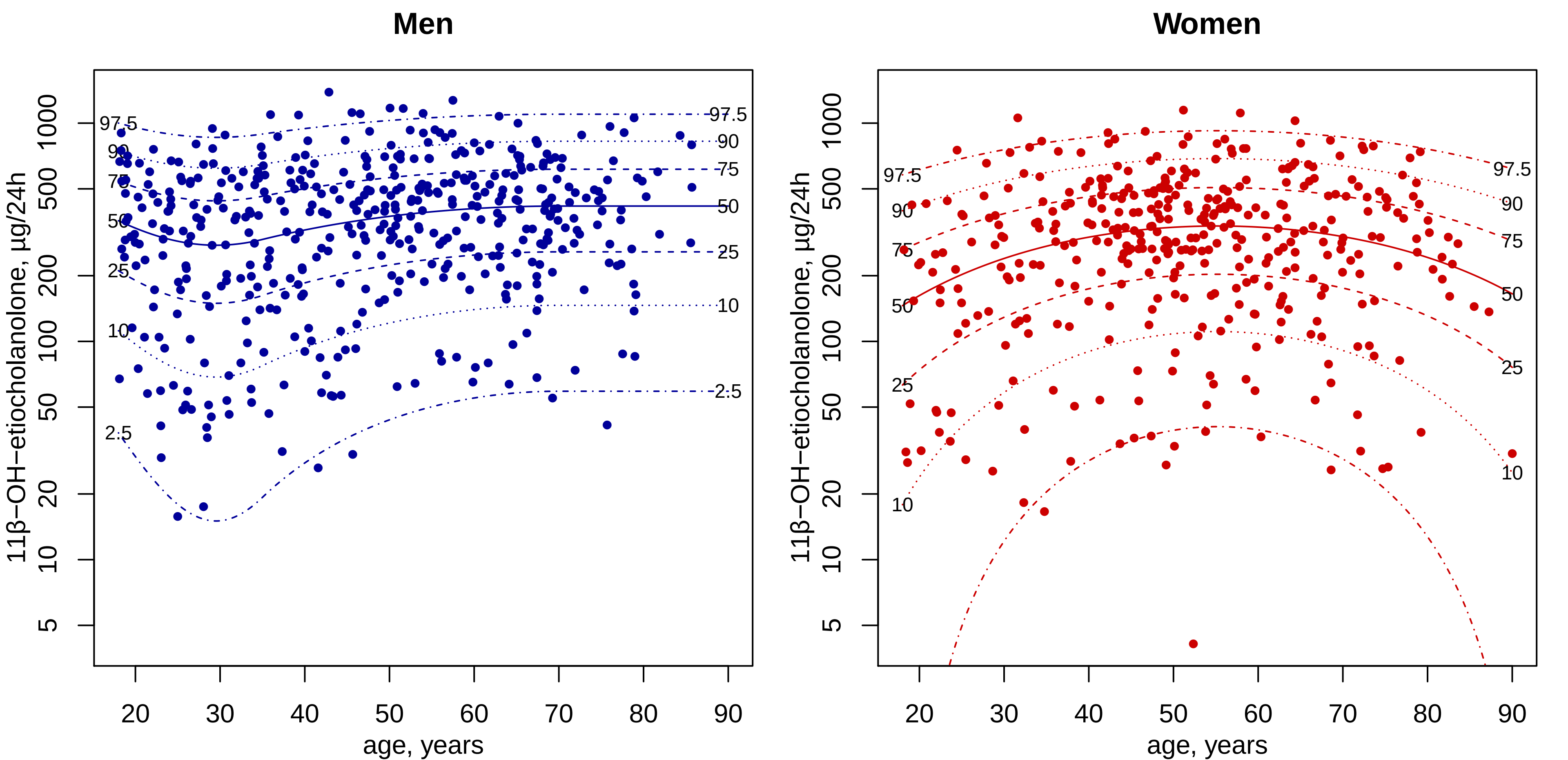
Men
Women
11β−OH−etiocholanolone, μg/24h
age, years
97.5
90
75
50
25
10
2.5
1000
500
200
100
50
20
10
5
20
30
40
50
60
70
80
90

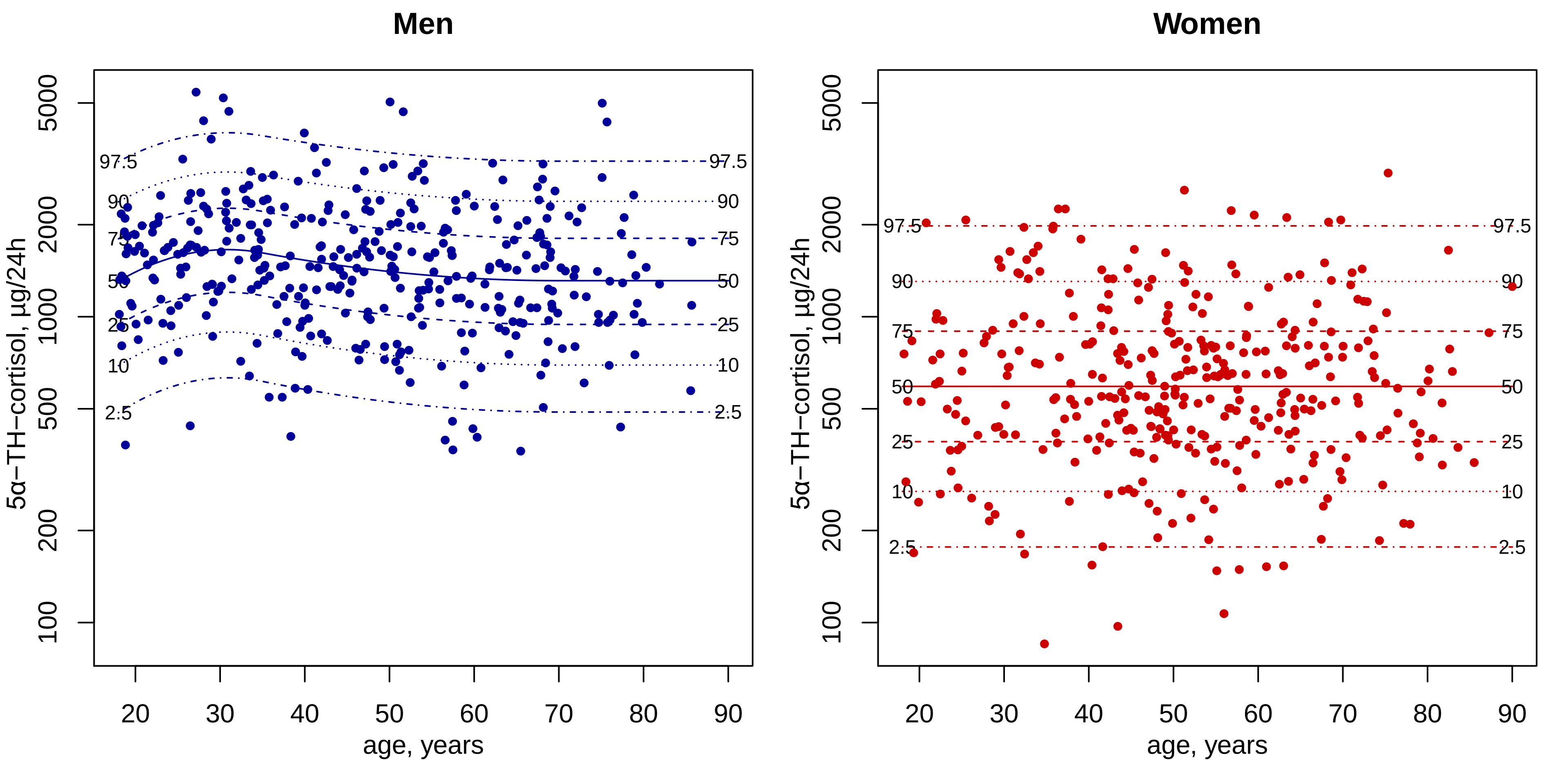

Men
Women
5000
2000
1000
500
200
100
5α−TH−cortisol, µg/24h
97.5
90
75
50
25
10
2.5
20
30
40
50
60
70
80
90
age, years

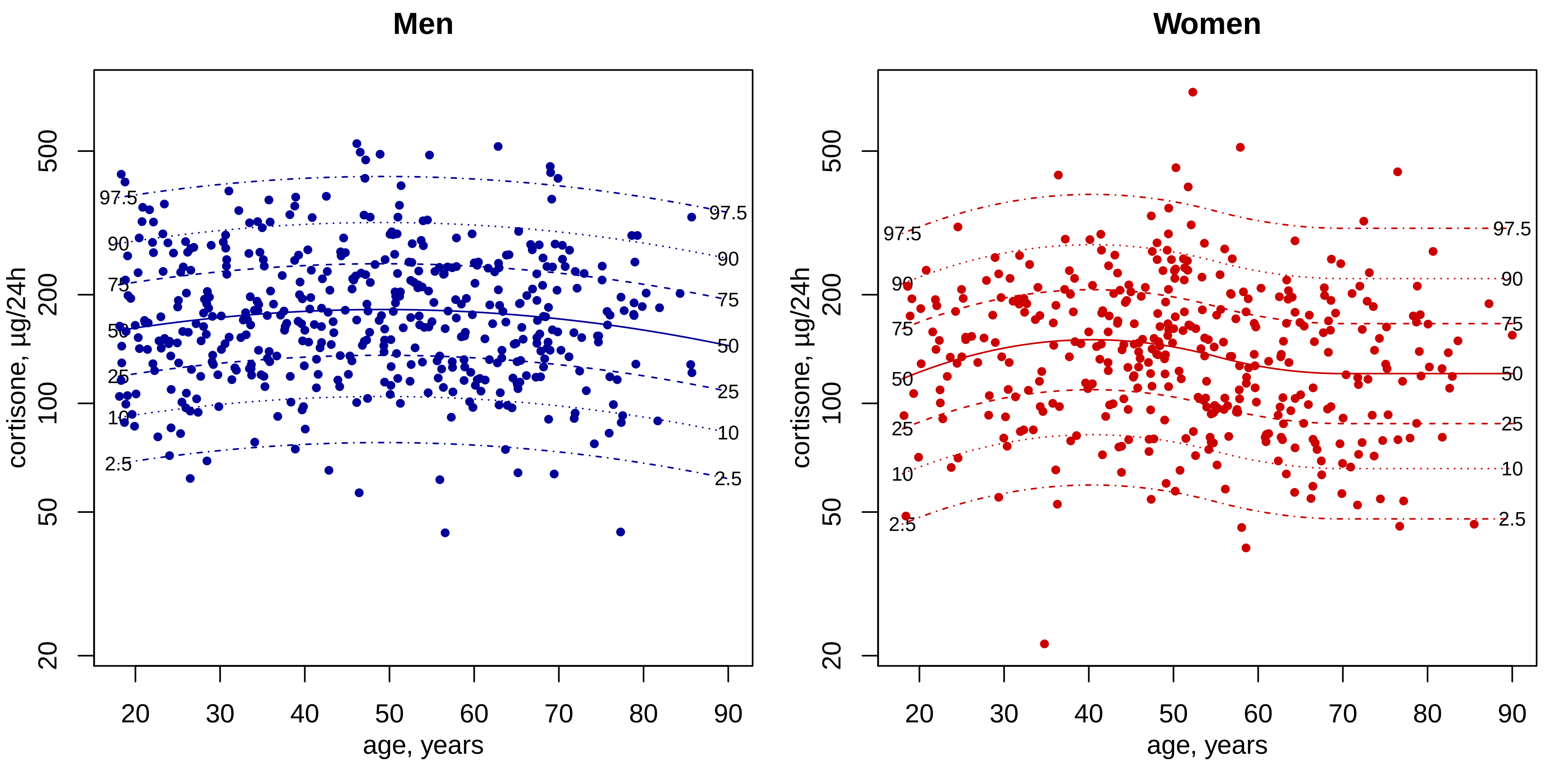
Men
Women
cortisone, µg/24h
age, years
500
200
100
50
20
20
30
40
50
60
70
80
90
97.5
90
75
50
25
10
2.5

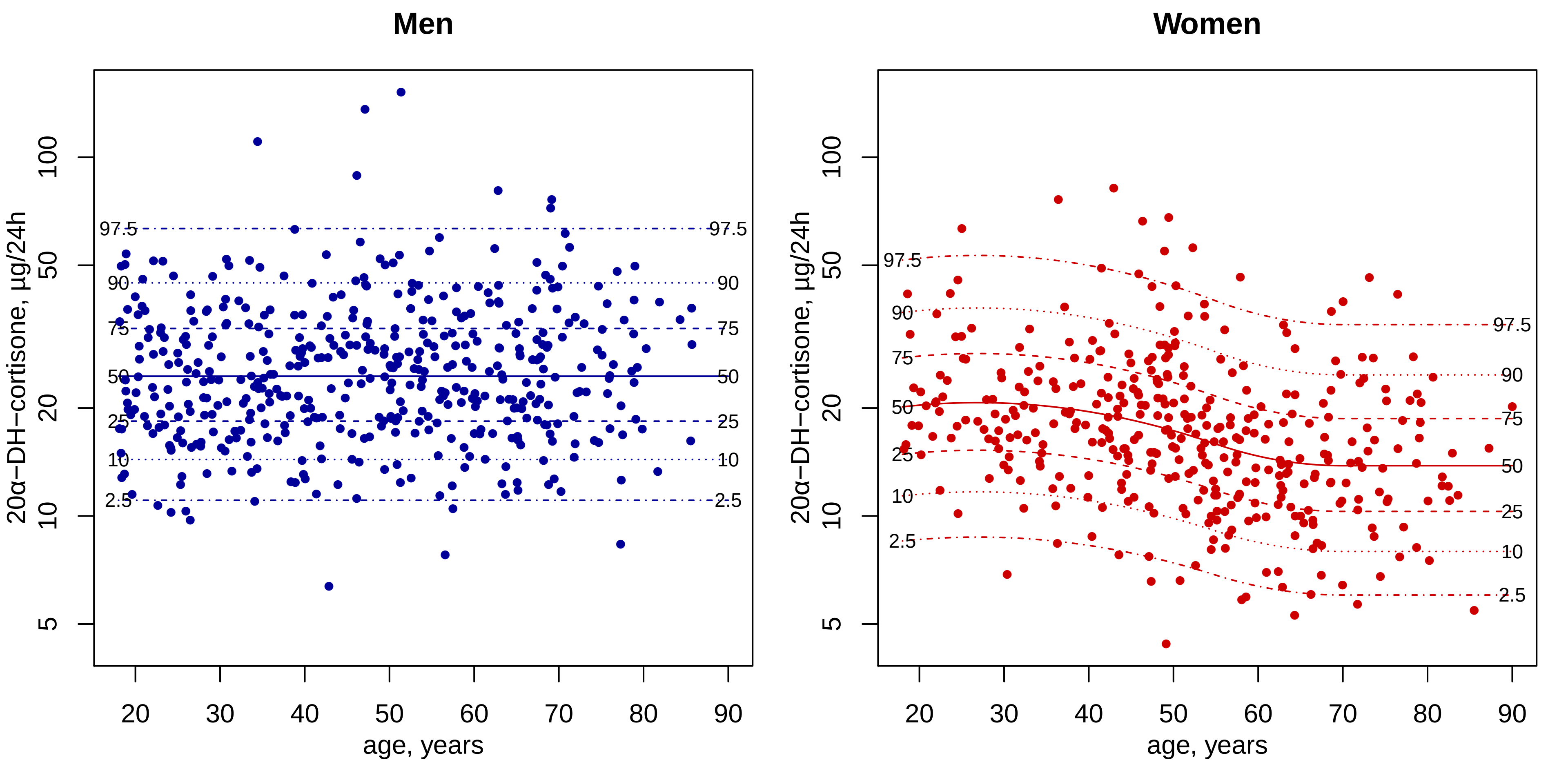

Men
Women
20α−DH−cortisone, µg/24h
age, years
97.5
90
75
50
25
10
2.5
5
10
20
50
100
20
30
40
50
60
70
80
90

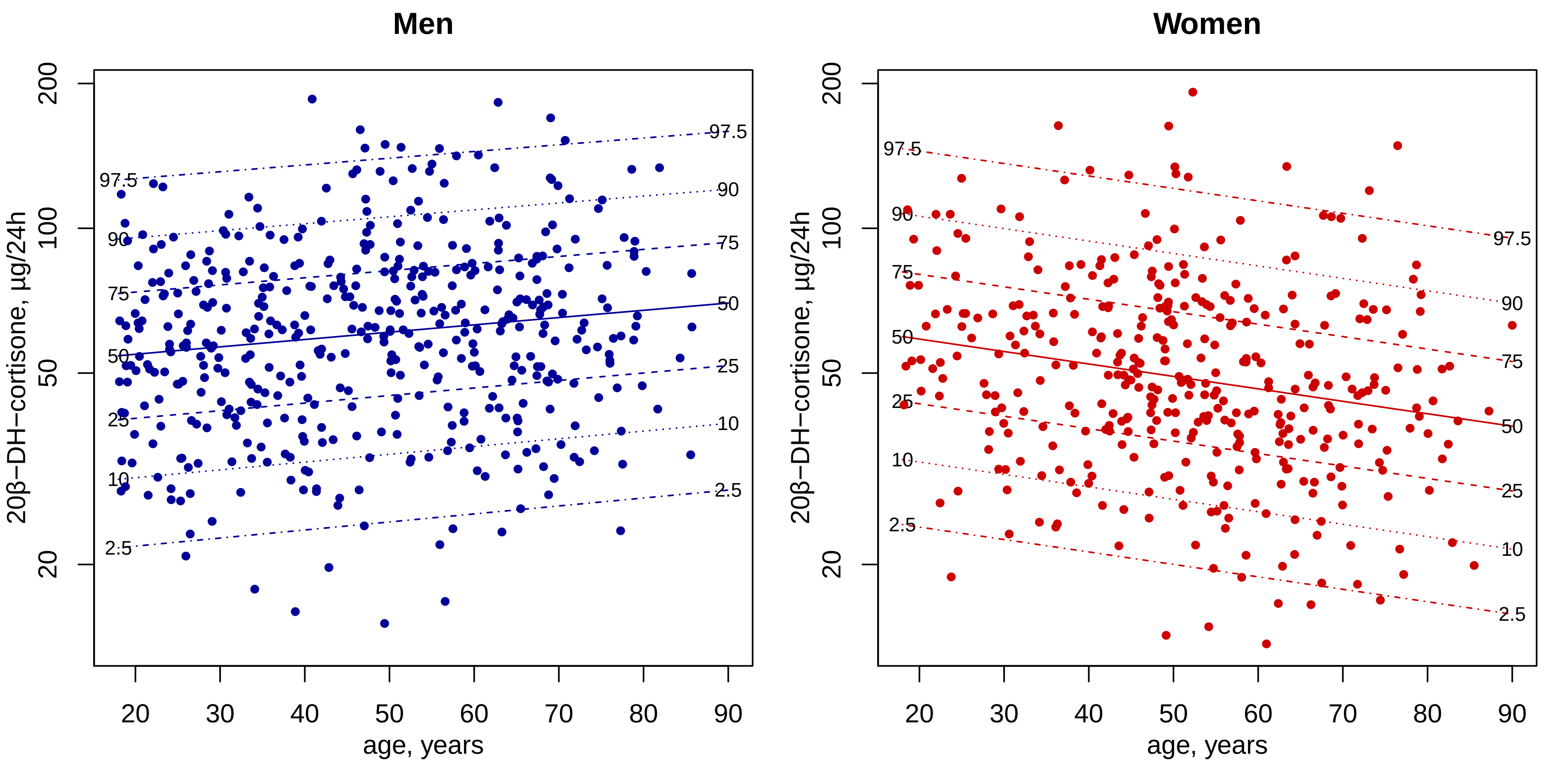
Men
Women
20β−DH−cortisone, µg/24h
200
100
50
20
age, years
20
30
40
50
60
70
80
90
97.5
90
75
50
25
10
2.5

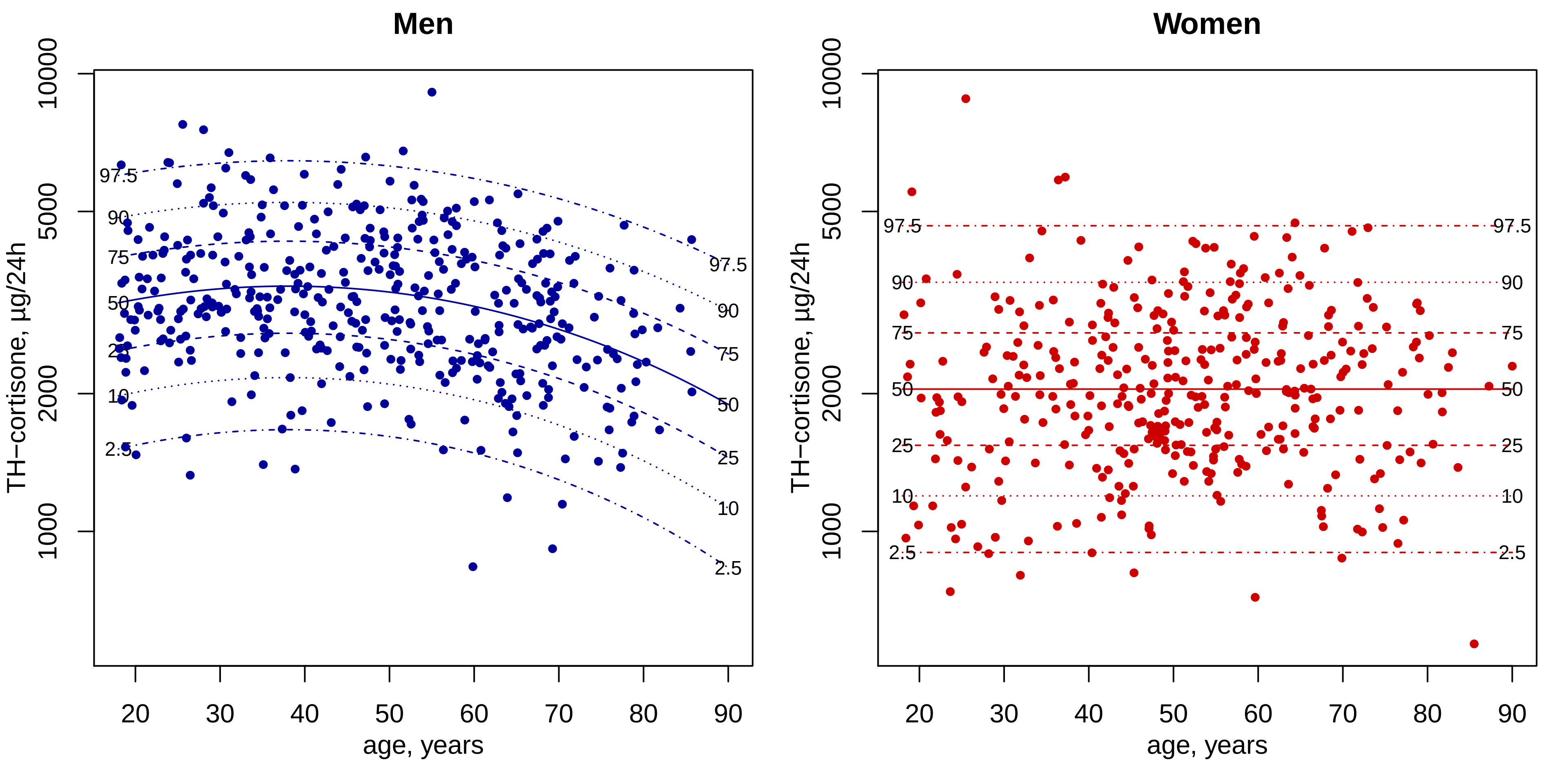
Men
Women
TH−cortisone, µg/24h
age, years
97.5
90
75
50
25
10
2.5
10000
5000
2000
1000
20
30
40
50
60
70
80
90

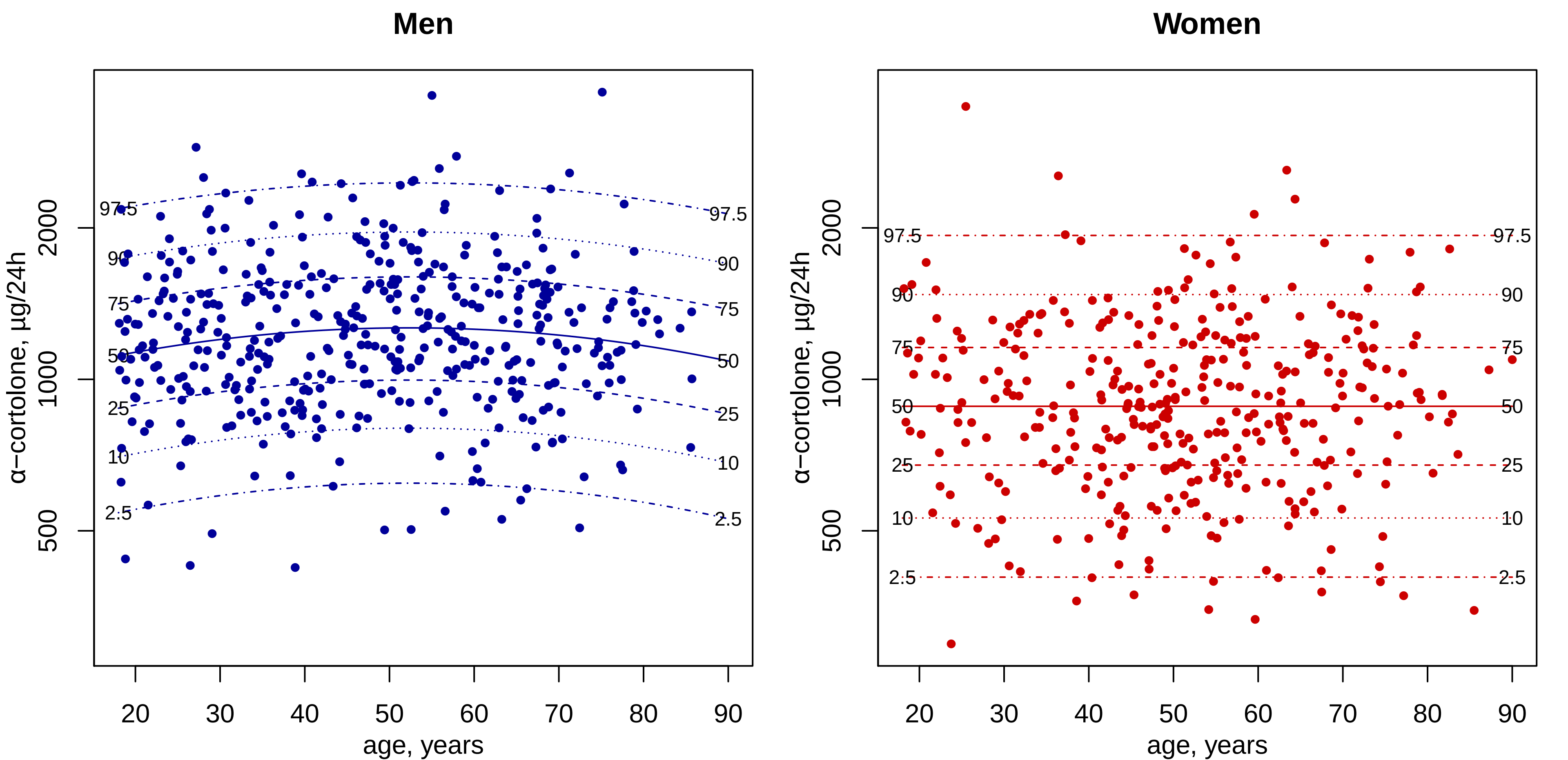
Men
Women
α−cortolone, µg/24h
age, years
97.5
90
75
50
25
10
2.5
500
1000
2000
20
30
40
50
60
70
80
90

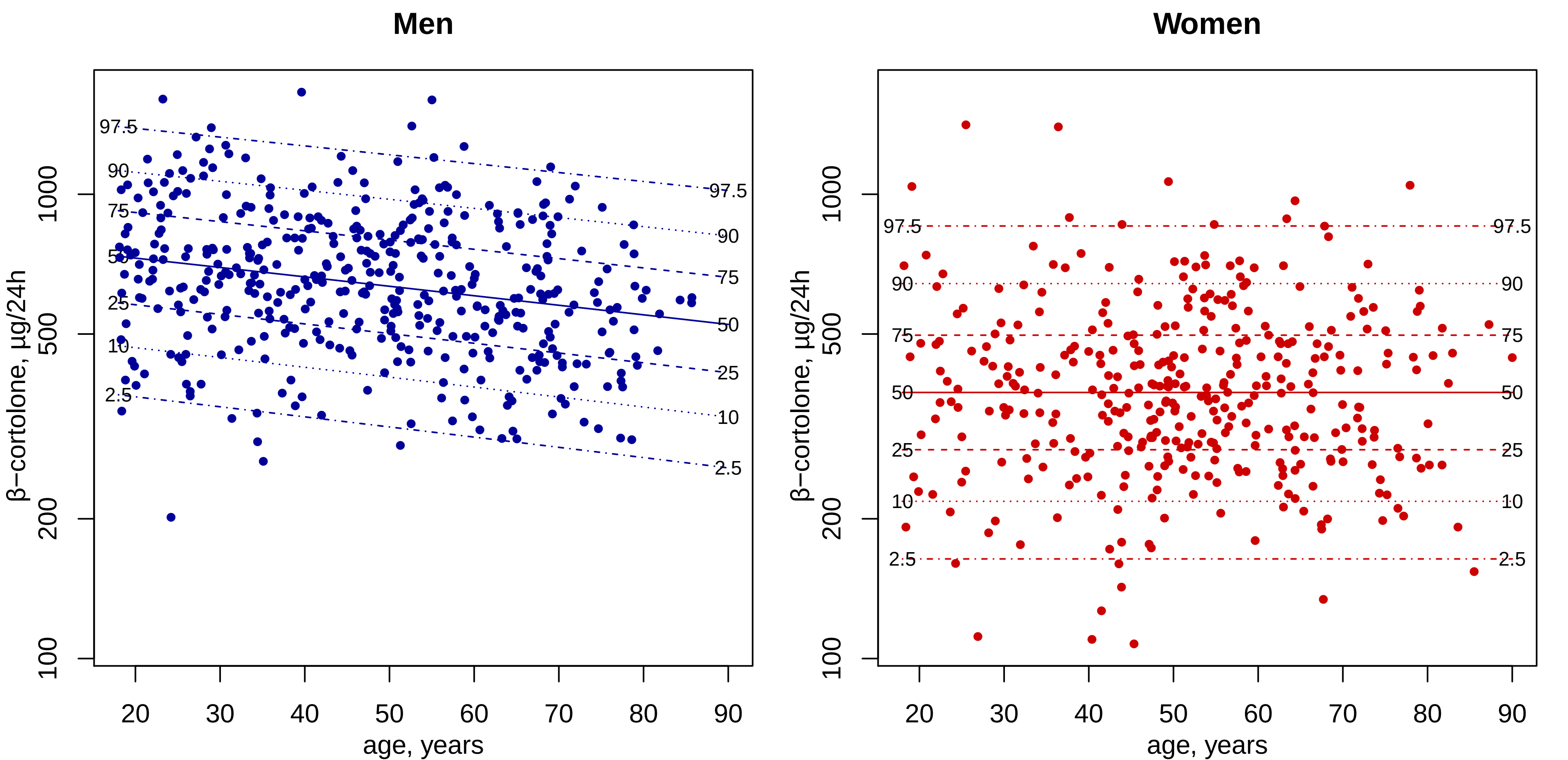

Men
Women
β−cortolone, µg/24h
age, years
97.5
90
75
50
25
10
2.5
1000
500
200
100
20
30
40
50
60
70
80
90

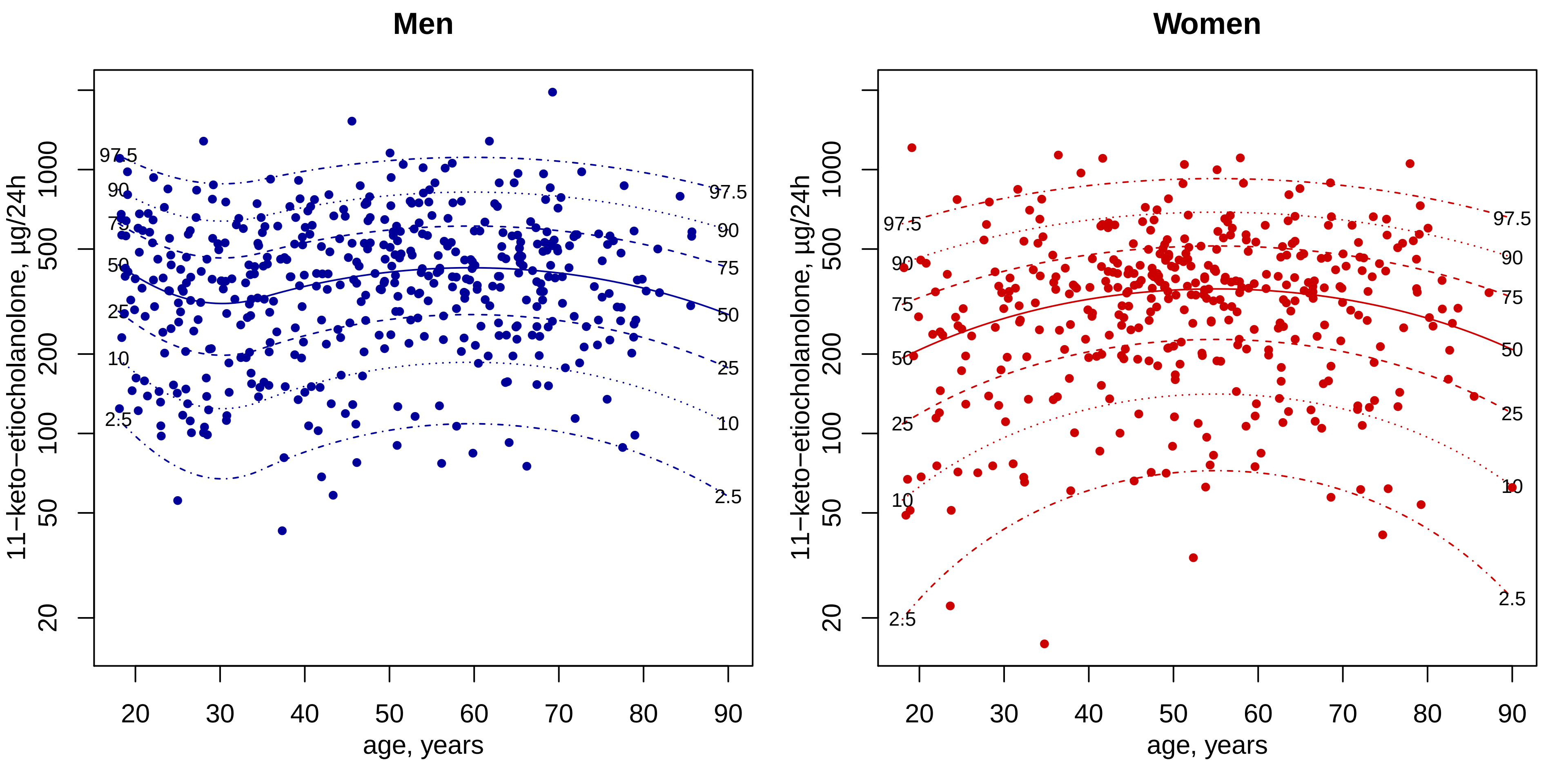

Men
Women
11−keto−etiocholanolone, μg/24h
age, years
20
50
100
200
500
1000
20
30
40
50
60
70
80
90
97.5
90
75
50
25
10
2.5
