# Supplementary material for: Reference intervals for the urinary steroid metabolome: The impact of sex, age, day and night time on human adult steroidogenesis
Source: PLoS One. 2019 Mar 29;14(3):e0214549. doi: 10.1371/journal.pone.0214549 (PMC6440635; doi:10.1371/journal.pone.0214549)
Supplement: S1 Table — Quantitative urinary steroid excretion values measured by GC-MS and published since 1986 in adults are shown. Abbreviations: GC: gas chromatography, MS: mass spectrometry, F: female, M: male, d: days, y: years. (PDF) [file pone.0214549.s005.pdf]

Supporting Table 1. Quantitative urinary steroid excretion values measured by GC-MS published since 1986 in adults.

Author, y, reference	Shackleton, 1986, (1)		Bevan et al., 1986, (2)	Weykamp et al., 1989, (3)					
**Sex**	F	M	F	F			M		
**Age (y)**	22-50	22-50	reproductive age	17-50	51-70	> 70	17-50	51-70	> 70
**Number of subjects**	13	17	NA	24	24	24	24	24	24
**Type of data**	NA		NA	2.5th-97.5th percentile					
**Measuring unit**	µg/24h	µg/24h	µmol/24h	µmol/24h					
**Analysis method**	GC-MS		NA	GC without MS					
**Population**	random sample corrected for mean daily creatinine excretion		non-pregnant women of reproductive age	good health, no medication, female: neither pregnancy nor using oral contraceptives, at 24 institutions in The Netherland and Belgium (1 subject from each sex and age stratum per institution)					
**Subgroups**	-	-	-	-	-	-	-	-	-
**Progesterones**									
17α-OH-pregnanolone									
pregnanetriol	362	555	1.0-4.2	1.0-3.9	0.6-2.5	0.3-1.3	1.4-6.2	1.3-4.7	0.8-3.1
pregnenetriol	421	522							
pregnanetriolone									
pregnanediol			0.4-4.8	0.7-7.1	0.3-4.2	0.0-2.2	0.5-3.3	0.5-2.7	0.4-1.8
**Androgens**									
dehydroepiandrosterone	512	2243	0.2-1.0	0.2-1.6	0.2-0.9	0.1-0.5	0.0-2.8	0.0-3.0	0.0-1.8
16α-OH-dehydroepiandrosterone			0.6-1.3						
androstenediol									
androstenetriol			0.3-1.9						
testosterone									
5α-DH-testosterone									
androstanediol									
androsterone	1847	2430	2.1-6.5	2.3-9.7	0.6-5.5	0.2-3.1	3.8-14.1	2.3-11.6	0.5-6.8
11β-OH-androsterone	675	1031	1.2-3.8	1.3-4.8	1.3-4.1	1.0-3.1	2.0-7.5	2.0-7.5	1.5-5.6
etiocholanolone	2471	3018	2.7-6.9	2.4-9.4	1.2-6.1	0.3-3.3	3.0-15.8	2.5-12.7	0.8-7.3
**Estrogens**									
17β-estradiol									
estriol									
**Corticosterones**									
TH-11-deoxycorticosterone									
TH-11-dehydrocorticosterone	526	553	0.1-0.4						
18-OH-TH-11-dehydrocorticosterone									
TH-corticosterone	245	253	0.2-0.7						
5α-TH-corticosterone	504	440	0.4-0.8						
**Mineralocorticoids**									
TH-aldosterone									
TH-11-deoxycortisol				0.2-1.2	0.2-1.0	0.1-0.5	0.1-1.2	0.0-1.2	0.0-0.8
**Glucocorticoids**									
cortisol									
6β-OH-cortisol									
18-OH-cortisol									
20α-DH-cortisol									
TH-cortisol	1552	1956	1.3-2.7	2.4-7.0	2.4-7.1	2.2-6.7	3.4-12.4	3.9-13.1	3.6-11.7
α-cortol	235	194	0.4-0.7						
α-cortolone	524	479	0.7-1.9						
β-cortol<br>β-cortolone	506#	600#	0.9-3.7#						
11β-OH-etiocholanolone	283	300	0.3-0.9	0.5-2.7	0.5-2.6	0.4-2.4	0.5-3.1	0.5-2.4	0.2-1.8
allo-TH-cortisol	625	1254	0.9-2.3	1.1-5.6	0.7-4.9	0.5-3.3	1.8-10.4	1.5-10.3	0.8-8.6
cortisone									
20α-DH-cortisone									
20β-DH-cortisone									
TH-cortisone	3113	3107	3.9-7.7	4.6-11.6	3.8-11.2	3.4-10.8	6.7-20.2	6.1-17.0	4.3-13.8
11-keto-androsterone	343*	515*		0.3-1.9	0.3-1.9	0.3-1.4	0.3-2.7	0.1-2.4	0.0-1.9
11-keto-etiocholanolone			0.1-0.8	0.3-1.8	0.3-1.6	0.2-1.1	0.4-2.5	0.4-2.1	0.2-1.6
Creatinine, mmol/24h				7.1-12.4	4.7-11.6	4.1-10.3	9.2-19.4	7.2-18.5	5.3-15.8

Abbreviations: GC, gas chromatography; MS, mass spectrometry; F, female; M: male; d, days; y, years. #the value represents the sum of the two compounds β-cortol and β-cortolone. *the value represents the sum of the two compounds 11-keto-androsterone and 11-keto-androsterone

Supporting Table 1. (continued)

Author, year, reference	Shackleton, 1993, (4)		Finken et al., 1999, (5)			
**Sex**	F	M	F			M
**Age (y)**	18-50	18-50	20-40			22-35
**Number of subjects**	17	17	10	10	10	10
**Type of data**	Mean, Range		Median [25th-75th percentile]			
**Measuring unit**	µg/24h	µg/24h	µg/24h			
**Analysis method**	GC-MS		GC-MS			
**Population**	NA		white, 10 healthy men and 10 healthy women with regular endogenous menstrual cycles (between 24-34 days), all studies were completed during the months December to March			
**Subgroups**	-	-	Menstrual phase (2-5 d after starting menstruation)	Follicular phase (19-16 d before the next expected menstruation)	Luteal phase (9-5 d before next menstruation)	-
**Progesterones**						
17α-OH-pregnanolone	128, 22-570	243, 72-452				
pregnanetriol	559, 117-1742	618, 189-1737				
pregnenetriol	161, 80-352	307, 71-363				
pregnanetriolone	14, 3-46	16, 6-66				
pregnanediol	351, 66-2654	173, 110-446				
**Androgens**						
dehydroepiandrosterone	188, 34-1497	490. 71-2455				
16α-OH-dehydroepiandrosterone	310, 63-1377	520-110-1480				
androstenediol	97, 29-203	323, 38-390				
androstenetriol	321, 90-695	524, 87-1433				
testosterone						
5α-DH-testosterone						
androstanediol	95, 16-231	186, 67-649				
androsterone	1289, 457-3656	2109, 738-5204				
11β-OH-androsterone	463, 202-1189	992, 280-2821				
etiocholanolone	1504, 488-3684	1865, 652-3387	1881 [1384-2492]	1628 [1234-2571]	2122 [1209-2651]	2191 [1076-2566]
**Estrogens**						
17β-estradiol	9, 0.1-15	3, 0.1-6				
estriol	12, 1-30	5, 1-10				
**Corticosterones**						
TH-11-deoxycorticosterone	104, 50-253	150, 44-328				
TH-11-dehydrocorticosterone	19, 1-108	14, 2-38				
18-OH-TH-11-dehydrocorticosterone	75, 20-150	139, 32-431				
TH-corticosterone	95, 36-276	127, 14-393				
5α-TH-corticosterone	238, 79-525	323, 117-1275				
**Mineralocorticoids**						
TH-aldosterone	29, 7-51	31, 8-60				
TH-11-deoxycortisol	33, 18-72	53, 16-111				
**Glucocorticoids**						
cortisol	81, 19-212	130, 13-243	150 [84-204]	163 [115-178]	117 [71-256]	119 [85-139]
6β-OH-cortisol	171, 101-532	254, 48-666				
18-OH-cortisol	79, 6-153	134, 6-410				
20α-DH-cortisol	53, 22-92	62, 21-111				
TH-cortisol	1045, 321-1908	1722, 615-2997	1600 [1419-1968]	1770 [1412-1965]	2007 [1434-2259]	2197 [1748-2995]
α-cortol	166, 67-289	214, 110-704				
α-cortolone	888, 379-1316	1062, 376-2438				
β-cortol	285, 35-759	431, 138-793				
β-cortolone	367, 84-802	657, 242-1180				
11β-OH-etiocholanolone	212, 24-844	345, 74-635				
allo-TH-cortisol	696, 164-1252	1459, 414-2599	1811 [1391-2300]	1950 [1698-2324]	1943 [1765-2245]	2723 [2454-3154]
cortisone			123 [85-145]	116 [111-146]	133 [122-195]	135 [115-150]
20α-DH-cortisone						
20β-DH-cortisone						
TH-cortisone	2066, 639-3528	3046, 1193-6760	1927 [1678-2860]	2238 [1789-2735]	1999 [1525-2515]	2393 [2082-2895]
11-keto-androsterone<br>11-keto-etiocholanolone	278, 123-991*	497, 148-1104*				
Creatinine, mmol/24h						

Abbreviations: GC, gas chromatography; MS, mass spectrometry; F, female; M: male; d, days; y, years. *the value represents the sum of the two compounds 11-keto-androsterone and 11-keto-androsterone

Supporting Table 1. (continued)

**Author, y, reference**	**Shamim et al., 2000, (6)**			**Shackleton et al., 2006, (7) and 2008, (8)**		**Taylor, 2006, (9) and 2013, (10)**	
**Sex**	F	M	Sex difference	F	M	F	M
**Age (y)**	31.1±4	30.5±3.9		NA		NA	
**Number of subjects**	14	16		17	17	NA	
**Type of data**	Median [25th-75th percentile]		Mann-Whitney U-test	Range		Range	
**Measuring unit**	µg/24h			µg/24h	µg/24h	µg/24h	µg/24h
**Analysis method**	GC-MS			GC-MS		GC-MS	
**Population**	normal haemoglobin, serum sodium, potassium and creatinine, biochemistry blood tests, chest X-ray, electrocardiogram and 24h ambulatory blood pressure monitoring to exclude borderline hypertension, no medication, no endocrine disorders		*p*-value	NA		NA	
**Subgroups**	-	-	-	-	-	-	-
**Progesterones**							
17α-OH-pregnanolone				32-657	41-728		
pregnanetriol				87-1311	186-1505	<890	<1563
pregnenetriol				44-342	28-1062		
pregnanetriolone				1-77	4-37		
pregnanediol						<2430	<1450
**Androgens**							
dehydroepiandrosterone	100 [75-180]	460 [290-750]	0.01	20-1138	5-1476	<800	<1750
16α-OH-dehydroepiandrosterone	200 [150-377]	? [220-380]	0.2	35-655	40-796	<515	<1480
androstenediol				28-201	45-954		
androstenetriol	320 [195-877]	460 [430-560]	0.4	40-540	42-710	<760	<1630
testosterone							
5α-DH-testosterone							
androstanediol				15-147	48-578		
androsterone	720 [522-820]	1200 [1020-1482]	0.0001	373-3414	798-4705	<1610	490-2570
11β-OH-androsterone	325 [220-410]	730 [520-740]	0.003	191-854	500-1733	80-980	370-1340
etiocholanolone	955 [600-1260]	1220 [825-1502]	0.01	450-2900	689-3252	<2180	180-2424
**Estrogens**							
17β-estradiol							
estriol				2-32	1-16		
**Corticosterones**							
TH-11-deoxycorticosterone				1-157	2-38		
TH-11-dehydrocorticosterone				76-596	104-554	40-410	<660
18-OH-TH-11-dehydrocorticosterone				25-207	45-184		
TH-corticosterone				26-262	32-238	<360	10-410
5α-TH-corticosterone				49-447	135-588	<422	80-570
**Mineralocorticoids**							
TH-aldosterone				6-63	10-58		
TH-11-deoxycortisol				17-117	10-109		
**Glucocorticoids**							
cortisol				25-115	35-168		
6β-OH-cortisol				53-416	122-487		
18-OH-cortisol							
20α-DH-cortisol							
TH-cortisol	830 [480-950]	1080 [1025-1350]	0.0007	458-1907	942-2800	250-1510	310-2240
α-cortol	200 [147-262]	265 [215-365]	0.05	122-365	96-509	70-340	60-700
α-cortolone	730 [350-910]	770 [717-917]	0.1	457-1564	449-2044	140-1210	<1520
β-cortol	765 [490-820]#	1040 [890-1102]#	0.002	124-690	196-880		
β-cortolone				216-814	231-1534	<1100	150-1590
11β-OH-etiocholanolone	160 [112-207]	140 [97-332]	0.39	14-687	18-1034	20-650	<1540
allo-TH-cortisol	400 [270-530]	830 [607-925]	0.0002	142-1589	796-2456	90-920	190-2220
cortisone				49-215	92-366		
20α-DH-cortisone							
20β-DH-cortisone							
TH-cortisone	1485 [1140-1990]	2290 [2115-2715]	0.001	727-3815	1365-5788	370-3510	570-5700
11-keto-androsterone							
11-keto-etiocholanolone				57-916	79-1026	30-610	<1280
Creatinine, mmol/24h							

Abbreviations: GC, gas chromatography; MS, mass spectrometry; F, female; M: male; d, days; y, years.

Supporting Table 1. (continued)

**Author, y, reference**	**Chan et al., 2008, (11)**							
**Sex**	F				M			
**Age (y)**	44.7 ±13.1 (mean±SD), range 20-85				41.6 ±12.9 (mean±SD), range 23-78			
**Number of subjects**	89				83			
**Type of data**	Median [2.5th-97.5th percentile] (in the original publication also the 25th and 75th percentile are provided)							
**Measuring unit**	µg/24h							
**Analysis method**	GC-MS							
**Population**	Staff of the Department of Pathology, Queen Elizabeth Hospital, Hong Kong, their families, relatives and friends. Exclusion criteria included tobacco smoking, alcohol consumption (>14 units/week in female, >21 units/week in male), acute or chronic medical or mental illnesses, known malignancies, use of chronic medications, herbs or vitamin supplements, pregnant or lactatingwomen and miscarriage in the last 3 months.							
**Subgroups**	All n=84-89	< 40 y n=29	40-59 y n=29	≥ 60 y n=29	All n=81-83	< 40 y n=41-43	40-59 y n=29-30	≥ 60 y n=10
**Progesterones**								
17α-OH-pregnanolone	61 [16-295]				146 [54-365]	165 [67-372]	134 [50-289]	107 [38-267]
pregnanetriol	272 [81-1152]				540 [228-1297]	665 [284-1471]	476 [219-970]	346 [185-718]
pregnenetriol	82 [15-344]				283 [38-925]	363 [107-976]	347 [54-764]	118 [25-256]
pregnanetriolone	6 [<1-44]				7 [<1-39]			
pregnanediol	173 [48-2899]				182 [58-457]	222 [75-476]	180 [57-331]	106 [41-348]
**Androgens**								
dehydroepiandrosterone	114 [7-1672]				885 [32-3657]	1152 [164-3657]	889 [49-3105]	167 [15-1214]
16α-OH-dehydroepiandrosterone	213 [28-1114]				483 [67-1376]	535 [253-379]	486 [91-670]	626 [17-322]
androstenediol	38 [7-211]				173 [14-626]	233 [51-833]	151 [20-496]	25 [10-99]
androstenetriol	199 [41-475]				392 [143-954]			
testosterone								
5α-DH-testosterone								
androstanediol								
androsterone	738 [127-1898]	1083 [431-2037]	684 [143-1765]	533 [100-919]	1805 [410-3498]	2312 [792-3514]	2195 [589-3105]	957 [343-2372]
11β-OH-androsterone	453 [136-944]				763 [233-1822]	776 [233-1618]	904 [318-1898]	527 [311-1059]
etiocholanolone	745 [184-1466]				1215 [437-2802]	1374 [577-2802]	1169 [586-2637]	828 [235-1362]
**Estrogens**								
17β-estradiol								
estriol								
**Corticosterones**								
TH-11-deoxycorticosterone								
TH-11-dehydrocorticosterone	59 [19-141]				97 [31-255]	115 [41-255]	95 [29-167]	72 [31-228]
18-OH-TH-11-dehydrocorticosterone								
TH-corticosterone	58 [19-123]				99 [36-248]	108 [41-251]	100 [36-161]	75 [31-198]
5α-TH-corticosterone	102 [36-242]				216 [58-610]	251 [58-653]	204 [49-402]	170 [79-311]
**Mineralocorticoids**								
TH-aldosterone								
TH-11-deoxycortisol	19 [9-59]				31 [10-90]			
**Glucocorticoids**								
cortisol	17 [6-38]				28 [10-72]			
6β-OH-cortisol								
18-OH-cortisol								
20α-DH-cortisol								
TH-cortisol	719 [307-1445]				1161 [527-2350]			
α-cortol	154 [63-326]				217 [79-486]			
α-cortolone	766 [255-1364]				1097 [549-2484]			
β-cortol	223 [61-561]				315 [107-841]			
β-cortolone	320 [93-693]				468 [207-1146]	520 [220-1391]	493 [237-771]	312 [204-573]
11β-OH-etiocholanolone	66 [9-357]				117 [21-503]	142 [17-572]	213 [26-482]	152 [24-321]
allo-TH-cortisol	654 [145-1620]				1625 [413-4353]	1834 [422-4959]	1848 [299-3283]	1223 [465-1855]
cortisone								
20α-DH-cortisone								
20β-DH-cortisone								
TH-cortisone	1753 [547-3117]				2825 [1239-6367]	3051 [1253-6409]	3117 [1301-4917]	1740 [1202-3293]
11-keto-androsterone								
11-keto-etiocholanolone	97 [6-437]				168 [37-541]	177 [41-622]	240 [51-486]	79 [36-295]

Abbreviations: GC, gas chromatography; MS, mass spectrometry; F, female; M: male; d, days; y, years.

Supporting Table 1. (continued)

Author, y, reference	de Jong et al., 2017, (12)											
**Sex**	F						M					
**Age (y)**	20-29	30-39	40-49	50-59	60-69	70-79	20-29	30-39	40-49	50-59	60-69	70-79
**Number of subjects**	20	20	20	20	20	20	20	20	20	20	20	20
**Type of data**	Mean or *Median [2.5^{th}-97.5^{th} percentile]											
**Measuring unit**	µmol/24h											
**Analysis method**	GC-MS/MS											
**Population**	healthy, no medication, body mass index 21-30 kg/m^2, selected from the LifeLines Cohort Study, a large population based cohort study in which inhabitants of the northern part of The Netherlands and their families were invited to participate, (13)											
**Subgroups**	-	-	-	-	-	-	-	-	-	-	-	-
**Progesterones**												
17α-OH-pregnanolone												
pregnanetriol	2.1* [0.6-5.7]	2.7* [0.7-6.5]	2.6 [0.1-5.3]	1.1* [0.5-2.6]	1.0* [0.5-2.7]	1 [0.2-1.8]	4.3* [2.2-8.4]	3.3 [0.6-6.0]	3.3 [1.2-5.3]	2.8* [1.4-6.4]	2.2* [1.0-6.5]	2.6* [1.3-5.2]
pregnenetriol	0.8* [0.1-2.2]	0.5* [0.0-2.8]	0.3* [0.0-1.1]	0.2* [0.0-0.7]	0.2* [0.0-0.4]	0.1* [0.0-0.4]	2 [0.1-4.0]	1.1* [0.2-3.9]	1.2 [0.2-2.3]	0.8 [0.1-1.7]	0.5* [0.1-1.3]	0.8 [0.1-1.5]
pregnanetriolone	0.04* [0.0-0.2]	0.03* [0.0-0.2]	<0.1 [<0.1]	<0.1 [<0.1]	0.06* [0.0-0.4]	0.05* [0.0-0.3]	<0.1 [<0.1]	<0.1 [<0.1]	0.05* [0.0-0.2]	0.06* [0.0-0.2]	0.06* [0.0-0.4]	0.07* [0.0-0.2]
pregnanediol	1.5* [0.3-14.2]	2.2* [0.4-21.9]	1.6* [0.2-25.6]	0.6* [0.2-1.4]	0.7* [0.2-1.7]	0.6 [0.1-1.2]	1.3* [0.6-3.7]	1.0* [0.3-2.4]	1.0* [0.5-2.2]	0.8* [0.3-2.6]	0.8* [0.2-2.4]	1.1 [0.1-2.0]
**Androgens**												
dehydroepiandrosterone	1.2* [0.2-11.8]	0.8* [0.1-12.2]	0.7* [0.1-5.7]	0.3* [0.2-1.0]	0.3* [0.1-0.8]	0.2* [0.0-0.6]	6.6* [0.5-41.2]	1.5* [0.2-17.3]	2.6* [0.6-12.9]	1.1* [0.2-7.0]	0.6* [0.2-3.2]	0.9* [0.1-7.8]
16α-OH-dehydroepiandrosterone	1.7 [0.1-3.4]	1.0* [0.1-5.0]	0.6* [0.2-3.3]	0.3* [0.0-1.5]	0.2* [0.0-1.1]	0.2* [0.0-0.5]	4.2* [1.2-11.3]	2.0* [0.2-12.6]	1.9 [0.4-3.4]	1.2* [0.1-4.2]	0.7* [0.1-3.2]	1.0* [0.2-4.6]
androstenediol												
androstenetriol	1.1* [0.3-5.8]	1.1* [0.2-2.8]	0.9* [0.3-2.4]	0.6 [0.1-1.1]	0.5* [0.1-1.9]	0.4 [0.0-0.8]	2.5* [1.0-9.6]	2.2* [0.5-9.9]	2.1* [0.5-5.1]	1.4* [0.5-4.4]	1.3 [0.1-3.0]	1.2* [0.5-3.4]
testosterone												
5α-DH-testosterone												
androstanediol												
androsterone	6.7* [2.8-20.8]	8 [0.1-17.3]	5.2* [2.1-15.4]	2.7* [1.1-7.0]	2.5* [0.5-8.8]	1.9* [0.3-6.0]	21.6 [6.8-36.4]	13.7* [6.4-29.0]	14 [5.5-22.7]	10.4 [3.2-17.6]	6.8* [3.5-14.7]	6.8 [2.6-11.0]
11β-OH-androsterone	1.7* [0.6-6.3]	2.2* [0.6-6.0]	2.9 [0.6-5.2]	2.3* [1.0-5.3]	2.5 [0.3-4.8]	2.8 [0.3-5.0]	5.3 [1.1-9.5]	4 [1.1-6.9]	4.8 [2.3-7.2]	4.2* [2.2-8.1]	4.5 [0.9-8.2]	4.4 [1.6-7.3]
etiocholanolone	8.1 [1.8-14.4]	7.3 [1.4-13.2]	6.2* [2.6-17.7]	4.2 [0.9-7.4]	3.3* [1.2-9.7]	2.5* [0.8-7.6]	13.5* [5.0-39.9]	8.6 [0.3-16.9]	9.6 [3.1-16.1]	8.6 [1.2-16.1]	7.3 [1.5-13.0]	7 [2.1-12.1]
**Estrogens**												
17β-estradiol												
estriol	<0.1 [<0.1]	<0.1 [<0.1]	<0.1 [<0.1]	<0.1 [<0.1]	<0.1 [<0.1]	<0.1 [<0.1]	<0.1 [<0.1]	<0.1 [<0.1]	<0.1 [<0.1]	<0.1 [<0.1]	<0.1 [<0.1]	<0.1 [<0.1]
**Corticosterones**												
TH-11-deoxycorticosterone	<0.1 [<0.1]	<0.1 [<0.1]	<0.1 [<0.1]	<0.1 [<0.1]	<0.1 [<0.1]	<0.1 [<0.1]	<0.1 [≤0.1]	<0.1 [<0.1]	<0.1 [<0.1]	<0.1 [<0.1]	<0.1 [≤0.1]	<0.1 [<0.1]
TH-11-dehydrocorticosterone	0.3* [0.1-1.3]	0.3 [0.0-0.5]	0.3* [0.2-0.6]	0.3* [0.1-0.8]	0.3* [0.1-0.9]	0.3* [0.0-0.8]	0.6 [0.1-1.1]	0.4* [0.1-1.1]	0.4 [0.1-0.8]	0.5 [0.1-0.9]	0.4* [0.0-1.1]	0.5 [0.1-0.9]
18-OH-TH-11-dehydrocorticosterone												
TH-corticosterone	0.4* [0.1-1.3]	0.4* [0.1-0.8]	0.4 [0.2-0.7]	0.4* [0.2-0.9]	0.4* [0.1-1.3]	0.4* [0.2-1.0]	0.6* [0.3-1.4]	0.5 [0.1-1.1]	0.5* [0.2-1.1]	0.5* [0.2-1.3]	0.56* [0.1-1.7]	0.7 [0.2-1.3]
5α-TH-corticosterone	0.8* [0.2-3.5]	0.8 [0.1-1.5]	0.8 [0.2-1.3]	0.5* [0.2-1.5]	0.6 [0.2-1.0]	0.6* [0.2-1.7]	1.8 [0.3-3.3]	1.7 [0.1-3.4]	1.5 [0.3-2.7]	1.3* [0.6-3.2]	1.0* [0.3-3.4]	1.6 [0.1-3.0]
**Mineralocorticoids**												
TH-aldosterone												
TH-11-deoxycortisol	0.1* [0.0-0.3]	0.2* [0.0-0.3]	0.2* [0.1-0.4]	0.2* [0.0-0.6]	0.2* [0.1-0.7]	0.2* [0.0-0.6]	0.2* [0.0-0.6]	0.2* [0.0-0.4]	0.2 [0.0-0.3]	0.2 [0.0-0.5]	0.3* [0.1-0.7]	0.3* [0.1-0.7]
**Glucocorticoids**												
cortisol												
6β-OH-cortisol												
18-OH-cortisol												
20α-DH-cortisol												
TH-cortisol	4.1 [1.0-7.1]	4.2* [2.2-8.7]	4.5* [2.8-8.0]	4.7 [1.8-7.7]	5.4* [2.6-12.7]	6.1 [2.2-10.0]	6.9* [3.5-13.5]	6.5 [3.1-10.0]	7.2 [3.8-10.6]	7.9 [2.5-13.4]	8.2* [3.9-16.0]	9.7 [4.0-15.3]
α-cortol	0.6 [0.1-1.1]	0.5 [0.3-1.3]	0.6 [0.3-1.0]	0.5* [0.3-1.2]	0.7* [0.3-1.4]	0.9 [0.3-1.4]	0.8* [0.5-1.7]	0.7* [0.5-1.2]	0.9 [0.5-1.2]	0.8* [0.4-1.6]	1 [0.2-1.8]	1.2 [0.3-2.1]
α-cortolone	4.1 [0.9-7.4]	3.3* [1.5-7.4]	3.9 [1.3-6.6]	3.7 [0.9-6.4]	3.5* [2.1-7.1]	4.7 [1.8-7.5]	5.4 [2.2-8.5]	4.3* [2.7-6.7]	4.8 [2.8-6.7]	4.6 [1.9-7.3]	5 [1.5-8.6]	5.2 [2.1-8.3]
β-cortol												
β-cortolone	1.7 [0.1-3.3]	1.6 [0.4-2.8]	1.6* [0.7-4.0]	1.5* [0.8-3.2]	1.6* [0.8-3.1]	2.1 [0.7-3.5]	2.7* [1.4-6.1]	2.7 [0.9-4.5]	2.7 [1.0-4.5]	2.7 [0.9-4.5]	2.5* [1.3-4.8]	2.5 [0.9-4.1]
11β-OH-etiocholanolone	1.2 [0.1-2.3]	1.3* [0.3-3.8]	1.6 [0.2-3.1]	1.6 [0.2-3.0]	1 [0.1-2.1]	1.4* [0.4-4.7]	1.9 [0.1-4.0]	1.6* [0.0-4.7]	1.9 [0.1-4.1]	2 [0.1-4.6]	1.4* [0.2-6.3]	1.9* [0.5-7.6]
allo-TH-cortisol	3.2* [0.4-10.4]	3.6 [0.1-7.6]	3.1 [0.9-5.4]	2.4* [1.1-6.3]	2.8 [0.7-4.8]	2.3* [0.5-9.0]	7.4* [2.5-21.6]	8.5 [1.8-15.3]	8.3 [3.9-12.8]	7.1* [3.3-14.7]	5.9* [3.0-13.2]	7 [1.6-12.4]
cortisone												
20α-DH-cortisone												
20β-DH-cortisone												
TH-cortisone	7.8* [3.1-23.4]	8.5 [2.6-14.4]	9.2* [4.7-18.2]	8.3* [4.5-18.4]	9.1* [4.8-19.4]	11.1 [3.4-18.8]	15.2 [5.0-25.3]	12.9* [6.5-24.7]	14 [7.1-20.9]	14.7 [5.1-24.2]	14.2 [5.4-23.0]	14.1 [5.6-22.6]
11-keto-androsterone												
11-keto-etiocholanolone	1.0* [0.4-3.1]	1.1* [0.5-2.8]	1.6 [0.1-3.1]	1.4* [0.6-3.9]	1.5* [0.4-3.6]	1.9 [0.5-3.8]	1.8 [0.1-3.6]	1.6 [0.1-3.5]	1.7 [0.1-3.4]	1.8* [0.4-5.2]	1.9* [0.5-4.8]	1.7* [0.4-4.6]
Creatinine, mmol/24h												

*Median

Abbreviations: GC, gas chromatography; MS, mass spectrometry; F, female; M: male; d, days; y, years.

References

1. Shackleton CH. Profiling steroid hormones and urinary steroids. Journal of chromatography. 1986;379:91-156.
2. Bevan BR, Savvas M, Jenkins JM, Baker K, Pennington GW, Taylor NF. Abnormal steroid excretion in gestational trophoblastic disease complicated by ovarian theca-lutein cysts. Journal of clinical pathology. 1986;39(6):627-34.
3. Weykamp CW, Penders TJ, Schmidt NA, Borburgh AJ, van de Calseyde JF, Wolthers BJ. Steroid profile for urine: reference values. Clinical chemistry. 1989;35(12):2281-4.
4. Shackleton CH. Mass spectrometry in the diagnosis of steroid-related disorders and in hypertension research. The Journal of steroid biochemistry and molecular biology. 1993;45(1-3):127-40.
5. Finken MJ, Andrews RC, Andrew R, Walker BR. Cortisol metabolism in healthy young adults: sexual dimorphism in activities of A-ring reductases, but not 11beta-hydroxysteroid dehydrogenases. The Journal of clinical endocrinology and metabolism. 1999;84(9):3316-21.
6. Shamim W, Yousufuddin M, Bakhai A, Coats AJ, Honour JW. Gender differences in the urinary excretion rates of cortisol and androgen metabolites. Annals of clinical biochemistry. 2000;37 (Pt 6):770-4.
7. Shackleton CHJM. Hyphenated Methods. The Encyclopedia of Mass Spectrometry. 82006.
8. Shackleton C. Genetic Disorders of Steroid Metabolism Diagnosed by Mass Spectrometry. In: Blau N, Duran M, Gibson KM, editors. Laboratory Guide to the Methods in Biochemical Genetics: Springer Berlin Heidelberg; 2008. p. 549-605.
9. Taylor NF. Urinary Steroid Profiling. In: Wheeler MJ, Hutchinson JSM, editors. Methods in Molecular Biology: Hormone Assays in Biological Fluids. Totowa, NJ: Humana Press Inc.; 2006. p. 159-75.
10. Taylor NF. Urinary steroid profiling. Methods in molecular biology (Clifton, NJ). 2013;1065:259-76.
11. Chan AO, Taylor NF, Tiu SC, Shek CC. Reference intervals of urinary steroid metabolites using gas chromatography-mass spectrometry in Chinese adults. Steroids. 2008;73(8):828-37.
12. de Jong WHA, Buitenwerf E, Pranger AT, Riphagen IJ, Wolffenbuttel BHR, Kerstens MN, et al. Determination of reference intervals for urinary steroid profiling using a newly validated GC-MS/MS method. Clinical chemistry and laboratory medicine : CCLM / FESCC. 2017;56(1):103-12.
13. Scholtens S, Smidt N, Swertz MA, Bakker SJ, Dotinga A, Vonk JM, et al. Cohort Profile: LifeLines, a three-generation cohort study and biobank. International journal of epidemiology. 2015;44(4):1172-80.
