# Supplementary material for: Reference intervals for the urinary steroid metabolome: The impact of sex, age, day and night time on human adult steroidogenesis
Source: PLoS One. 2019 Mar 29;14(3):e0214549. doi: 10.1371/journal.pone.0214549 (PMC6440635; doi:10.1371/journal.pone.0214549)
Supplement: S2 Table — The number of participants is indicated for each characteristic and sex group. Categorical variables are described by % and continuous variables by their mean±standard deviation or by their median;25th-75th percentiles. Sex-specific differences were determined by chi squared test or Mann–Whitney U test, and the corresponding p values are indicated. (PDF) [file pone.0214549.s006.pdf]

Supporting Table 2. Baseline characteristics of the reference sample group.

Characteristics	Reference sample group				
	N	Men	N	Women	*p*
Male	459	54.8%	379	45.2%	-
Age, y	459	47.7±17.6	379	51.2±16.1	0.0054
Body mass index, kg/m^2	459	25.4;22.9-28.1	378	23.8;21.4-26.5	<0.001
Current smoker	130	28.3%	71	18.7%	0.001
Hypertension	126	27.5%	75	19.8%	0.01
Diabetes	29	6.3%	10	2.6%	0.012
**Blood values**					
Sodium, mmol/L	454	141;139-142	378	141;139-143	0.10
Potassium, mmol/L	454	4.1;3.9-4.3	378	4;3.8-4.2	0.0039
Chloride, mmol/L	453	105;103-107	378	105;103-107	0.10
Calcium total, mmol/L	454	2.29;2.24-2.34	377	2.29;2.23-2.35	0.65
Phosphate, mmol/L	453	0.99±0.16	377	1.09±0.16	<0.001
Creatinine, µmol/L	454	81.8±13.8	378	66.2±10.4	<0.001
eGFR CKD-EPI, mL/min per 1.73 m^2	454	98.2;85.8-110	378	93.5;82.4-103	<0.001
Urea, mmol/L	454	5.15;4.2-6.2	378	4.5;3.73-5.5	<0.001
Uric acid, mmol/L	454	351;308-391	378	260;224-302	<0.001
Glucose fasting, mmol/L	454	5.2;4.9-5.7	378	5;4.61-5.3	<0.001
Albumine, g/L	454	40;37-44	376	40;36-44	0.16
Aspartate aminotransferase, U/L	454	24;20-29	378	22;18-25	<0.001
Alanine aminotransferase, U/L	452	24;18-32	378	17;13.3-23	<0.001
Gamma-glutamyltransferase, U/L	452	24;16-35	377	15;9-23	<0.001
C-reactive protein, mg/L	451	0.5;0.5-2.05	376	0.5;0.5-1.9	0.53
Hemoglobin, g/L	451	148;141-154	375	134;129-140	<0.001
Thrombocytes, ×10^9/L	449	212;181-243	374	238;201-269	<0.001
Leukocytes, ×10^9/L	451	5.6;4.7-6.55	374	5.6;4.7-6.4	1.00
**Urine values**					
Volume day+night-time collection, mL	459	1520;1108-2079	379	1700;1247-2242	0.024
Volume day collection, mL	459	1000;703-1500	379	1135;800-1580	0.023
Volume nighttime collection, mL	459	450;300-632	379	496;300-700	0.43
Collection time day+nighttime, min	458	1440;1395-1480	379	1440;1390-1480	0.71
Collection time day, min	458	960;915-1010	379	950;885-990	<0.001
Collection time nighttime, min	457	480;435-525	379	495;450-540	<0.001
Creatinine, µmol/(day+nighttime)	458	15465;13234-17514	378	9663;8413-11067	<0.001
Creatinine, µmol/day	459	9900;8332-11574	378	6263;5096-7304	<0.001
Creatinine, µmol/nighttime	458	5200;4444-6253	379	3350;2796-4069	<0.001

The number of participants is indicated for each characteristic and sex group. Categorical variables are described by % and continuous variables by their mean±standard deviation or by their median;25th-75th percentiles. Sex-specific differences were determined by chi squared test or Mann–Whitney U test, and the corresponding *p* values are indicated.
