# Supplementary material for: Reference intervals for the urinary steroid metabolome: The impact of sex, age, day and night time on human adult steroidogenesis
Source: PLoS One. 2019 Mar 29;14(3):e0214549. doi: 10.1371/journal.pone.0214549 (PMC6440635; doi:10.1371/journal.pone.0214549)
Supplement: S3 Table — The available number of participants is indicated for each metabolite stratified for sex. Metabolites in the unit μg/24 hours are described by their median;25th-75th percentile. Between-group differences were determined by Mann–Whitney U test, and the corresponding p values are indicated. (PDF) [file pone.0214549.s007.pdf]

Supporting Table 3. Sex specific differences in 24 hours urinary excretion of steroid hormone metabolites.

Metabolite, µg/24 hours	Men		Women		
	N	Median;25th-75th	N	Median;25th-75th	*p*
17α-OH-pregnanolone	451	173;118-247	375	51.1;32.9-111	<0.001
pregnanetriol	395	676;518-920	360	340;226-526	<0.001
pregnenetriol	448	193;96.2-371	378	71.2;30.4-134	<0.001
pregnanetriolone	457	16.2;11.4-24.6	379	12.2;7.7-20.2	<0.001
pregnanediol	457	206;145-294	376	204;118-388	0.57
dehydroepiandrosterone	439	148;54.6-572	377	55;25.1-141	<0.001
16α-OH-dehydroepiandrosterone	448	258;89.4-495	379	92;44-214	<0.001
androstenediol	453	118;53.3-273	378	42.9;21.8-76.2	<0.001
androstenetriol	456	406;236-626	378	167;90.5-277	<0.001
testosterone	451	46.3;29.9-70.4	367	7.2;4.4-13.5	<0.001
5α-DH-testosterone	456	24.4;15.1-36.3	377	9.8;6.1-16.8	<0.001
androstanediol	445	85.1;58.7-114	372	22.3;13.8-35.8	<0.001
androsterone	381	1840;1194-2719	349	644;337-1092	<0.001
11β-OH-androsterone	447	861;646-1086	376	461;339-630	<0.001
etiocholanolone	390	1544;1029-2362	351	895;493-1399	<0.001
17β-estradiol	457	2.4;1.8-3.2	377	1.9;1-4.2	0.0016
estriol	456	6.2;4.4-8.8	374	4.8;2-11.3	0.0012
TH-11-deoxycorticosterone	455	7.3;5.1-10.4	378	6;3.5-10.2	<0.001
TH-11-dehydrocorticosterone	452	105;76.9-140	379	74.3;53.7-108	<0.001
18-OH-TH-11-dehydrocorticosterone	433	54.4;34.1-81.2	342	33.4;21.9-57.6	<0.001
TH-corticosterone	457	143;110-198	379	112;80-151	<0.001
5α-TH-corticosterone	457	325;236-456	379	178;119-257	<0.001
TH-aldosterone	456	20;12.6-31.5	378	17.2;10.6-30.3	0.0842
TH-11-deoxycortisol	457	67;49.5-87.5	379	51.7;38-71.4	<0.001
cortisol	457	110;82.5-152	379	84.2;59.1-121	<0.001
6β-OH-cortisol	457	107;72.2-147	378	91.7;58.6-134	<0.001
18-OH-cortisol	424	180;114-275	344	173;108-249	0.16
20α-DH-cortisol	457	53.3;38.2-76.5	379	43.7;31.7-65.7	<0.001
TH-cortisol	371	1766;1407-2231	340	1167;881-1455	<0.001
α-cortol	452	328;255-421	379	219;166-290	<0.001
β-cortol	453	456;352-622	378	272;198-369	<0.001
11β-OH-etiocholanolone	455	373;199-546	378	294;162-452	<0.001
allo-TH-cortisol	381	1445;1055-1986	362	582;409-851	<0.001
cortisone	456	169;129-232	379	137;96.1-188	<0.001
20α-DH-cortisone	457	24.4;18.3-33.1	379	16.7;12.3-22.5	<0.001
20β-DH-cortisone	457	61.3;44.1-81.1	379	47.7;36.2-66.6	<0.001
TH-cortisone	407	3091;2492-4016	360	2006;1528-2661	<0.001
α-cortolone	426	1221;984-1525	362	885;673-1153	<0.001
β-cortolone	427	639;507-818	369	373;280-479	<0.001
11-keto-etiocholanolone	455	391;248-558	379	342;198-455	<0.001

The available number of participants is indicated for each metabolite stratified for sex. Metabolites in the unit µg/24 hours are described by their median;25th-75th percentile. Between-group differences were determined by Mann–Whitney U test, and the corresponding *p* values are indicated.
