# Supplementary material for: Reference intervals for the urinary steroid metabolome: The impact of sex, age, day and night time on human adult steroidogenesis
Source: PLoS One. 2019 Mar 29;14(3):e0214549. doi: 10.1371/journal.pone.0214549 (PMC6440635; doi:10.1371/journal.pone.0214549)
Supplement: S4 Table — The available number of participants is indicated for each metabolite stratified for sex. Metabolites in the unit μg/hour are described by their median;25th-75th percentile. Within-sex differences were determined by Wilcoxon signed-rank test, and the corresponding p values are indicated. (PDF) [file pone.0214549.s008.pdf]

Supporting Table 4. Day and nighttime specific differences in urinary excretion of steroid hormone metabolites in men and women.

Metabolite, µg/hour	Men					Women				
	Day		Night			Day		Night		
	N	Median;25th-75th	N	Median;25th-75th	*p*	N	Median;25th-75th	N	Median;25th-75th	*p*
17α-OH-pregnanolone	455	7.33;4.88-10.6	451	6.91;4.7-10.2	0.013	379	2.41;1.44-5	375	1.83;1.12-4.03	<0.001
pregnanetriol	422	27.7;20.6-39.3	410	28.4;19.5-39.3	0.51	370	14.7;9.38-22.7	365	14.1;9.39-21.3	0.015
pregnenetriol	453	8.31;4.1-16.3	449	7.24;3.61-13	<0.001	379	3.21;1.23-6.05	378	2.51;1.05-5.08	<0.001
pregnanetriolone	456	0.766;0.518-1.12	456	0.509;0.358-0.847	<0.001	379	0.548;0.368-0.908	379	0.373;0.234-0.685	<0.001
pregnanediol	456	8.31;5.9-12.3	456	8.16;5.49-12.8	0.13	379	8.77;4.89-17.7	376	8.01;4.76-15.4	0.0018
dehydroepiandrosterone	443	6.78;2.32-28.9	443	4.71;1.94-16.5	<0.001	378	2.37;1.05-6.47	378	1.85;0.83-4.45	<0.001
16α-OH-dehydroepiandrosterone	449	10.8;3.71-21.6	449	8.97;3.04-20.7	<0.001	379	4.03;1.74-9.33	379	3.71;1.69-7.67	<0.001
androstenediol	455	5.05;2.16-11.7	452	4.84;2.13-10.1	<0.001	379	1.8;0.897-3.35	378	1.6;0.884-2.94	<0.001
androstenetriol	456	16.8;9.78-25.8	455	16.3;9.28-26.3	0.61	379	6.8;3.63-11.3	378	7.07;3.77-11.5	0.30
testosterone	453	1.85;1.17-2.86	451	1.96;1.26-3.08	0.0087	374	0.3;0.173-0.558	369	0.291;0.173-0.526	0.055
5α-DH-testosterone	455	1.03;0.61-1.5	456	0.871;0.548-1.45	<0.001	378	0.43;0.261-0.744	378	0.338;0.195-0.621	<0.001
androstanediol	449	3.47;2.35-4.66	447	3.5;2.34-5.2	0.18	375	0.917;0.57-1.4	374	0.896;0.502-1.49	0.0097
androsterone	404	76.7;49.4-113	400	73.2;47.9-108	0.029	362	26.9;15.2-47	359	24.6;13.8-45.1	<0.001
11β-OH-androsterone	450	38.5;28.6-50.6	450	27.6;19.9-39	<0.001	376	20.9;15.3-28.5	378	15.4;10.8-21.1	<0.001
etiocholanolone	407	65.6;40-98.9	413	63.3;41.8-96.1	0.83	369	38.1;21.7-56.9	359	37.5;19.6-58.4	0.042
17β-estradiol	456	0.097;0.069-0.132	456	0.103;0.071-0.147	0.0056	377	0.082;0.041-0.17	379	0.08;0.038-0.182	0.78
estriol	455	0.251;0.172-0.348	456	0.261;0.172-0.363	0.29	377	0.186;0.081-0.473	375	0.189;0.083-0.479	0.98
TH-11-deoxycorticosterone	455	0.315;0.215-0.447	455	0.272;0.181-0.385	<0.001	379	0.263;0.154-0.446	378	0.225;0.13-0.408	<0.001
TH-11-dehydrocorticosterone	451	4.74;3.26-6.34	455	3.36;2.35-5.18	<0.001	379	3.38;2.31-4.82	379	2.52;1.69-4.05	<0.001
18-OH-TH-11-dehydrocorticosterone	447	2.31;1.49-3.49	437	1.84;1.17-3.16	<0.001	359	1.49;1-2.49	351	1.11;0.655-1.91	<0.001
TH-corticosterone	456	6.24;4.44-8.65	456	5.19;3.72-7.83	<0.001	379	4.68;3.45-6.49	379	4.07;2.8-6.02	<0.001
5α-TH-corticosterone	456	15.4;10.9-21.7	456	9.53;6.64-13.4	<0.001	379	8.43;5.77-12.3	379	5.31;3.22-7.89	<0.001
TH-aldosterone	456	0.828;0.506-1.44	455	0.667;0.431-1.11	<0.001	379	0.769;0.47-1.32	378	0.63;0.342-1.06	<0.001
TH-11-deoxycortisol	456	2.99;2.18-3.82	456	2.3;1.61-3.28	<0.001	379	2.29;1.64-3.22	379	1.76;1.25-2.61	<0.001
cortisol	456	4.99;3.6-6.93	456	3.82;2.64-5.58	<0.001	379	3.69;2.6-5.39	379	2.8;2.04-4.05	<0.001
6β-OH-cortisol	456	4.84;3.13-6.75	456	3.58;2.22-5.37	<0.001	379	4.28;2.53-6.09	378	2.95;1.8-4.61	<0.001
18-OH-cortisol	446	8.08;5.05-12.8	429	4.78;3.13-8.32	<0.001	367	7.84;4.88-11.1	349	4.96;2.79-8.03	<0.001
20α-DH-cortisol	456	2.4;1.69-3.51	456	1.62;1.05-2.56	<0.001	379	1.98;1.39-2.9	379	1.51;0.833-2.34	<0.001
TH-cortisol	386	82.6;66.5-107	411	51.9;38-72.2	<0.001	354	56;41-70.2	355	34.2;24.5-48	<0.001
α-cortol	453	15;11.8-19.6	453	10.4;7.38-13.9	<0.001	379	10.2;7.61-13.7	379	6.6;4.76-9.48	<0.001
β-cortol	453	20.6;15.6-27.9	454	16.3;11.5-24.3	<0.001	379	12.2;9.31-17	378	9.09;6.17-13.8	<0.001
11β-OH-etiocholanolone	454	13.7;7.62-22.5	455	14.8;8.09-23.7	0.42	378	12;6.82-19	379	11.9;6.26-18.7	0.12
allo-TH-cortisol	393	68.8;49-92.6	419	43.6;30.3-63.2	<0.001	370	28.3;19.5-40.6	369	17.3;11.6-26.9	<0.001
cortisone	456	7.33;5.46-10.2	455	6.09;4.49-8.49	<0.001	379	6.09;4.13-8.21	379	4.59;3.09-6.83	<0.001
20α-DH-cortisone	456	1.13;0.84-1.57	456	0.709;0.499-1.07	<0.001	379	0.81;0.584-1.04	379	0.477;0.336-0.69	<0.001
20β-DH-cortisone	456	2.82;2-3.86	456	1.75;1.21-2.5	<0.001	379	2.24;1.61-3.02	379	1.49;1.03-2.19	<0.001
TH-cortisone	423	148;113-193	432	93.5;69.7-130	<0.001	368	94.6;71-125	368	62.9;44.2-88	<0.001
α-cortolone	431	57.5;45.3-74	444	35.6;26.1-47.6	<0.001	368	41.9;30.9-54.7	370	26.2;19.6-37.5	<0.001
β-cortolone	433	29.3;22.1-38	442	21.4;16.2-28.7	<0.001	372	16.6;12.7-22.6	376	12.3;8.96-16.9	<0.001
11-keto-etiocholanolone	455	16;9.98-23.5	455	15.2;9.02-22.4	<0.001	379	13.8;8.48-19.6	379	12.3;7-18.8	<0.001

The available number of participants is indicated for each metabolite stratified for sex. Metabolites in the unit µg/hour are described by their median;25th-75th percentile. Within-sex differences were determined by Wilcoxon signed-rank test, and the corresponding *p* values are indicated.
