# Supplementary material for: Reference intervals for the urinary steroid metabolome: The impact of sex, age, day and night time on human adult steroidogenesis
Source: PLoS One. 2019 Mar 29;14(3):e0214549. doi: 10.1371/journal.pone.0214549 (PMC6440635; doi:10.1371/journal.pone.0214549)
Supplement: S5 Table — The available number of participants is indicated for each metabolite stratified for sex. The correlation between day and nighttime excretion values was assessed by Spearman’s rank correlation coefficient ρ (rho). Rho values were ranked in ascending order within each sex group. Metabolites were colored by steroid groups as indicated. (PDF) [file pone.0214549.s009.pdf]

Supporting Table 5. Correlation between day and nighttime urinary excretion (µg/hour) of steroid hormone metabolites in men and in women.

Men				Women			
N	Metabolite	Rho	Group	Group	Rho	Metabolite	N
425	α-cortolone	0.446	Glucocorticoids	Glucocorticoids	0.489	20α-DH-cortisol	379
455	20α-DH-cortisol	0.454	Glucocorticoids	Glucocorticoids	0.516	cortisol	379
455	6β-OH-cortisol	0.457	Glucocorticoids	Glucocorticoids	0.550	cortisone	379
425	β-cortolone	0.484	Glucocorticoids	Glucocorticoids	0.567	20β-DH-cortisone	379
369	TH-cortisol	0.492	Glucocorticoids	Glucocorticoids	0.577	α-cortolone	362
405	TH-cortisone	0.495	Glucocorticoids	Glucocorticoids	0.581	6β-OH-cortisol	378
454	cortisone	0.502	Glucocorticoids	Glucocorticoids	0.598	TH-cortisol	340
455	20β-DH-cortisone	0.522	Glucocorticoids	Glucocorticoids	0.609	TH-cortisone	360
455	20α-DH-cortisone	0.523	Glucocorticoids	Glucocorticoids	0.614	20α-DH-cortisone	379
450	α-cortol	0.526	Glucocorticoids	Glucocorticoids	0.619	β-cortolone	369
455	17β-estradiol	0.534	Estrogens	Glucocorticoids	0.624	α-cortol	379
445	11β-OH-androsterone	0.540	Androgens	Corticosterones	0.625	TH-corticosterone	379
422	18-OH-cortisol	0.551	Glucocorticoids	Glucocorticoids	0.625	18-OH-cortisol	344
455	cortisol	0.563	Glucocorticoids	Mineralocorticoids	0.661	TH-11-deoxycortisol	379
455	5α-TH-corticosterone	0.573	Corticosterones	Androgens	0.664	11β-OH-androsterone	376
455	TH-corticosterone	0.597	Corticosterones	Corticosterones	0.668	18-OH-TH-11-dehydrocorticosterone	342
450	TH-11-dehydrocorticosterone	0.599	Corticosterones	Corticosterones	0.677	TH-11-dehydrocorticosterone	379
451	β-cortol	0.608	Glucocorticoids	Glucocorticoids	0.677	β-cortol	378
455	TH-11-deoxycortisol	0.614	Mineralocorticoids	Corticosterones	0.684	5α-TH-corticosterone	379
379	5α-TH-cortisol	0.645	Glucocorticoids	Mineralocorticoids	0.739	TH-aldosterone	378
453	TH-11-deoxycorticosterone	0.653	Corticosterones	Androgens	0.753	androstanediol	372
454	estriol	0.654	Estrogens	Glucocorticoids	0.756	11-keto-etiocholanolone	379
443	androstanediol	0.669	Androgens	Glucocorticoids	0.770	11β-OH-etiocholanolone	378
431	18-OH-TH-11-dehydrocorticosterone	0.673	Corticosterones	Glucocorticoids	0.784	5α-TH-cortisol	632
394	pregnanetriol	0.677	Progesterones	Androgens	0.789	5α-DH-testosterone	377
454	TH-aldosterone	0.691	Mineralocorticoids	Androgens	0.795	testosterone	367
453	11-keto-etiocholanolone	0.721	Glucocorticoids	Progesterones	0.824	pregnanetriolone	379
455	pregnanediol	0.723	Progesterones	Progesterones	0.850	pregnanetriol	360
454	5α-DH-testosterone	0.733	Androgens	Corticosterones	0.851	TH-11-deoxycorticosterone	378
449	17α-OH-pregnanolone	0.741	Progesterones	Estrogens	0.860	17β-estradiol	377
453	11β-OH-etiocholanolone	0.741	Glucocorticoids	Androgens	0.861	androstenediol	378
455	pregnanetriolone	0.742	Progesterones	Androgens	0.869	etiocholanolone	351
449	testosterone	0.744	Androgens	Androgens	0.870	dehydroepiandrosterone	377
388	etiocholanolone	0.774	Androgens	Progesterones	0.879	pregnenetriol	378
379	androsterone	0.781	Androgens	Androgens	0.881	androstenetriol	378
454	androstenetriol	0.828	Androgens	Progesterones	0.887	17α-OH-pregnanolone	375
446	16α-OH-dehydroepiandrosterone	0.863	Androgens	Progesterones	0.889	pregnanediol	376
446	pregnenetriol	0.866	Progesterones	Androgens	0.891	16α-OH-dehydroepiandrosterone	379
451	androstenediol	0.893	Androgens	Estrogens	0.891	estriol	374
437	dehydroepiandrosterone	0.908	Androgens	Androgens	0.896	androsterone	349

The available number of participants is indicated for each metabolite stratified for sex. The correlation between day and nighttime exretion values was assessed by Spearman's rank correlation coefficient ρ (rho). Rho values were ranked in ascending order within each sex group. Metabolites were colored by steroid groups as indicated.

Color code for steroid groups:

- Progesterones (yellow)
- Androgens (green)
- Estrogens (pink)
- Corticosterones (blue)
- Mineralocorticoids (light cyan)
- Glucocorticoids (red)
