# Supplementary material for: Reference intervals for the urinary steroid metabolome: The impact of sex, age, day and night time on human adult steroidogenesis
Source: PLoS One. 2019 Mar 29;14(3):e0214549. doi: 10.1371/journal.pone.0214549 (PMC6440635; doi:10.1371/journal.pone.0214549)
Supplement: S1 Text — The statistical methods applied are described in detail. (PDF) [file pone.0214549.s010.pdf]

Supporting Text 1. Statistical Methods. The statistical methods applied are described in detail.

Data for 40 steroid hormone metabolites were available from day and night-time urine collections for 459 men and 379 women finally included in the statistical analysis. The goal was to obtain reference intervals for the amount of each metabolite excreted during 24 hours in function of age (between 18 and 90 years) and sex. In a first descriptive analysis conducted on the log-scale, nonparametric fits revealed a different kind of relationship to age for men and for women for most steroids. We thus modeled separately the data for men and for women, leaving us with 80 models to develop, i.e. 40 models for men and 40 models for women.

To get reference intervals, one convenient approach is to look for a scale where near normality is achieved, allowing us to easily derive any percentile of the distribution, and enabling in turn the calculation of standard deviation scores (SDS) values. Since each steroid S was highly skewed, we started to correct for skewness via power transformation, yielding Y=sign(p)*S^p, where the optimal power p was selected on a fine grid ranging from -1 (corresponding to an inverse transformation) to 1 (corresponding to no transformation), and where the power 0 corresponded to a log-transformation (see G. E. P. Box, and D. R. Cox, 1964 [1]), to minimize the absolute value of skewness of Y (this was again done separately for men and women). To minimize their influence, the transformation was selected by removing the obvious outliers, which were detected sequentially using a boxplot rule, eliminating just one out of 10’000 observations in case of a perfect normality on the transformed scale.

The data Y hence obtained were then fitted in a linear mixed model with two nested random effects, a family effect (since individuals were nested into families) and a center effect (since families were nested into centers). Parameters to capture the age effect were included as fixed effects. Five such models have been considered: a constant function (i.e. no age effect with just an intercept), a linear function (2 parameters), a quadratic function (3 parameters), a quadratic spline function with one knot (5 parameters if we include the position of the knot) and a quadratic spline function with two knots and a levelling-off (4 parameters if the second knot is fixed), i.e. a function which becomes constant after a certain age, which can be constructed using B-splines (V. Rousson, 2008 [2]). The model minimizing the Akaike information criterion (AIC) has been ultimately selected. The first knot was chosen on a grid between 35 and 60 years (which were close to the 25th and the 75th percentile of the age distribution), whereas a levelling-off was obtained by adding a second knot at the late age of 75 (which was close to the 95th percentile of the age distribution).

SDS values were obtained for each measurement Y by subtracting the mean (which was thus age-dependent, noted MEAN(age)) and by dividing by the standard deviation provide SD by the fit (including all sources of variations, due to families, centers, as well as the random error), i.e. SDS=(Y-MEAN(age))/SD. SDS values were supposed to be approximately normally distributed. This was checked using a goodness of fit procedure, where we compared the number of observations falling below, between and above the 2.5^{th,} 10th, 25th, 50th, 75th, 90th and 97.5th percentile, assuming normality of SDS, with the theoretical values of a standardized normal distribution. Significant results were thus an indication that normality was not perfect. Note that while statistical models have been fitted without the outliers, goodness of fit procedures did include the outliers.

Let Zq the qth percentile of a standardized normal distribution. The qth percentile Sq for a steroid variable S at some given age measured on the original variable can be calculated as: Sq=sign(p)*(MEAN(age)+SD*Zq)^(1/p). If the selected power transformation is p=0, one calculates: Sq=exp(MEAN(age)+SD*Zq).

All calculations have been run using the free statistical package R version 3.3.3 [3], where linear mixed models have been fitted using the lmer function to be found in the lme4 library. B-splines have been implemented using the splines library.

Results

In our first descriptive analysis, we found significant differences between men and women for 37 out of the 40 steroids using a non-parametric Mann-Whitney test, with higher values for men than for women in each case. Even in case of a non-significant difference, the relationship to age might be different which motivated us to model the steroid variables separately for men and for women, as discussed above. We then compared the excretion rates of steroid analytes in μg/hour during the day and during the night using a signed-rank Wilcoxon test and we found significantly higher values on the day than on the night for 31 out of the 40 steroids for men, and for 35 out of the 40 steroids for women. Only for 2 steroids, we found significantly (although only slightly) higher values on the night than on the day in men.

The power transformation to correct for skewness was selected between -0.5 and +0.5 for all steroids and both genders, with most of them (47/80) between -0.1 and +0.1, i.e. close to a log-transformation. Out of the 32’507 available observations, 74 were detected as outliers and removed from subsequent analysis (apart from goodness of fit procedures).

A model without any age effect was selected for 15 out of the 80 models (5 steroids for men and 10 for women). Among the remaining 65 models, a linear age effect was selected in 17 cases, a quadratic effect in 14 cases, a quadratic spline in 21 cases and a quadratic spline with levelling-off in 13 cases, illustrating the variety of the possible relationships between the steroid variables and age.

Among the variance which was not explained by the factor age, the variance due to family ranged (depending on the steroid) between 6% and 41% (averaging 21%) for men, and between 0% and 54% (averaging 27%) for women. The variance due to the center ranged between 0% and 13% (averaging 2%) for men, and between 0% and 12% (averaging 3%) for women, the residual variance accounting on average for 78% (men) and for 70% (women) of the variability.

The goodness of fit statistics run at the 5% significance level did not reveal a contradiction between the model and the data for 74/80=92.5% of our models, which was in turn not significantly different from the expected 95%. Thus, our models were flexible enough to provide a good approximation of the reality in most cases.

References

[1] G.E.P. Box, D.R. Cox, An Analysis of Transformations, Journal of the Royal Statistical Society. Series B (Methodological), 26 (1964) 211-252.
[2] V. Rousson, Monotone fitting for developmental variables, Journal of Applied Statistics, 35 (2008) 659-670.
[3] R Development Core Team, R: A language and environment for statistical computing, R Foundation for Statistical Computing. Retrieved from https://www.R-project.org/, Vienna, Austria, 2017.
